# Open RGB imaging workflow for morphological and morphometric analysis of fruits using deep learning: a case study on almonds

**DOI:** 10.1093/gigascience/giaf157

**Published:** 2025-12-19

**Authors:** Jorge Mas-Gómez, Manuel Rubio, Federico Dicenta, Pedro José Martínez-García

**Affiliations:** Fruit Breeding Group, Department of Plant Breeding, Centro de Edafología y Biología Aplicada del Segura–Spanish National Research Council (CEBAS-CSIC), Campus Universitario Espinardo, E-30100 Murcia, Spain; Fruit Breeding Group, Department of Plant Breeding, Centro de Edafología y Biología Aplicada del Segura–Spanish National Research Council (CEBAS-CSIC), Campus Universitario Espinardo, E-30100 Murcia, Spain; Fruit Breeding Group, Department of Plant Breeding, Centro de Edafología y Biología Aplicada del Segura–Spanish National Research Council (CEBAS-CSIC), Campus Universitario Espinardo, E-30100 Murcia, Spain; Fruit Breeding Group, Department of Plant Breeding, Centro de Edafología y Biología Aplicada del Segura–Spanish National Research Council (CEBAS-CSIC), Campus Universitario Espinardo, E-30100 Murcia, Spain

**Keywords:** high-throughput phenotyping, almonds, breeding, computer vision, deep learning

## Abstract

**Background:**

High-throughput phenotyping is addressing the current bottleneck in phenotyping within breeding programs. Imaging tools are becoming the primary resource for improving the efficiency of phenotyping processes and providing large datasets for genomic selection approaches. The advent of artificial intelligence (AI) brings new advantages by enhancing phenotyping methods using imaging, making them more accessible to breeding programs. In this context, we have developed an open Python workflow for analyzing morphology, color, and morphometric traits using AI, which can be applied to fruits and other plant organs.

**Results:**

The workflow was implemented in almond (*Prunus dulcis* (Mill.) D. A. Webb), a species where breeding efficiency is critical due to its long breeding cycle. Over 25,000 kernels, more than 20,000 nuts, and over 600 individuals were phenotyped, making this the largest morphological study conducted in almond so far. The best segmentation and reconstruction approaches achieved error rates below 1%. Weight and area variables enabled accurate estimation of kernel thickness, with a root mean squared error of 0.47. Fifty-five heritable morphological, morphometric, and color traits were identified, highlighting their potential as target traits in breeding programs.

**Conclusion:**

The proposed workflow demonstrated robust performance across diverse datasets and was effective with limited training data for fine-tuning. Its compatibility with the output of AI-based labeling tools allows users to fully leverage the advantages of these technologies—reducing manual effort, accelerating dataset preparation, and streamlining the fine-tuning process of segmentation models. This flexibility enhances the scalability and practical applicability of the workflow in real-world phenotyping scenarios, especially in the context of breeding programs.

## Background

An observable performance of a plant (resulting from its genotype, environment, and their interaction) is defined as the phenotype. Phenotypes allow the portion attributable to genetic effects to be identified through quantitative genetics. The process of measuring and describing plant phenotypes is called phenotyping, and it remains the primary bottleneck in breeding programs [1]. To overcome this bottleneck, classical phenotyping tasks, which are often tedious, time-consuming, subjective, and/or expensive, are being replaced. Breeders are leveraging tools such as computer vision [2], artificial intelligence [3], and unmanned aerial platforms [4], among others, to meet the need for large-scale phenotyping and the generation of accurate, accessible big datasets [1]. Such high-throughput phenotyping enables the phenotyping of larger populations and the generation of quantitative measurements, which can improve prediction accuracy and response to selection in genomic selection schemes [5–8]. Clearly, high-throughput phenotyping is becoming a valuable tool for plant breeders and researchers to meet the challenges of future food demand [9, 10]. The use of high-throughput phenotyping platforms for fruit species such as apple, mango, vineyard or citrus has helped to study architecture parameters, pigment and nutrient contents, water stress, biochemical parameters of fruits, and disease detection [11]. A device for fast and accurate phenotyping of small fruit samples, called “FruitPhenoBox,” was developed for breeding programs [12], leading to the identification of key marker–trait associations in apple populations [13]. Additionally, by integrating genotype data, RGB imaging, and autoencoders, a framework was developed to reconstruct apple samples using only genotype data [14]. Morphometric approaches using machine learning have been applied in strawberries to extract quantitative shape features, enabling the identification of new heritable traits [15]. Furthermore, an automatic pipeline to phenotype morphological traits was developed for strawberries using computer vision techniques [16] and 3-dimensional (3D) imaging [17], with applicability to other fruits.

For stone fruit trees species, such as almond (*Prunus dulcis* (Mill.) D. A. Webb), the implementation of these tools is still scarce. Breeding almond trees and other *Prunus* species, such as apricot, peach, plum, or cherry, is a long and expensive task. Due to their long juvenility period, the timing to obtain a new, improved cultivar is around 12–15 years. Therefore, increasing efficiency is a key aspect for breeding these species, particularly in phenotyping processes of traits of interest, such as fruit morphology, shape, and color [18–20]. These traits directly influence market value and consumer acceptance [21, 22], and as have been commented, these traits are highly dimensional [16]; computing only a linear, univariate phenotype leads to a loss of information by extremely simplifying the features of a trait [23, 24], requiring the use of geometric-morphometric approaches [25].

Larger kernel sizes are generally more economically valuable [20], while smaller sizes are preferred for specific applications, such as chocolate bars [18]. Shape is also important for industrial processes: elongated almonds are preferred for slicing, whereas round almonds are generally more appreciated by consumers [18, 19]. Geometric parameters also affect the mechanical properties of almonds and other nuts, which are relevant for processing [26, 27]. For example, elongated shapes have been shown to result in lower breakage during blanching, while rounder shapes reduce breakage during shelling in Spanish almond industry machinery [28]. Preliminary efforts in imaging analysis and deep learning in almonds have demonstrated that the same quantitative trait loci (QTLs) for size and shape can be successfully identified using these approaches, as with traditional manual phenotyping methods [29]. Imaging has also been applied in the “Nonpareil” almond cultivar to measure length and predict kernel mass using machine learning for industrial applications [30]. Moreover, 3D imaging phenotyping methods have been developed for almonds. Software for measuring seed morphology has been developed, enabling the quantification of both shape and color traits [31–33]. However, none of these tools allow for detailed morphometric analyses of kernel shape, which can be an important commercial trait in almonds [21]. A common challenge is that elements (e.g., seeds or fruits) often touch each other [31], and algorithms such as watershed segmentation are not implemented in all available software. Among these tools, only SeedExtractor is open-source, providing a foundation for customization and further development, although it is implemented in MATLAB [31]. In contrast, widely adopted programming languages can facilitate faster and broader adoption. In this regard, PlantCV, written in Python, offers numerous tutorials for learning computer vision in plant phenotyping [34].

In most of the mentioned studies, segmentation—defined as the process of partitioning a digital image into meaningful regions of interest (ROIs) based on homogeneous visual features, such as color, texture, or intensity—is a core computer vision task, which its conventional use through binary thresholding often requires manual adjustments, limiting automation, especially as scene complexity increases [35, 36]. To enhance efficiency and automation, advanced tools capable of handling this complexity are essential for fruit morphology studies [37]. In this sense, deep learning techniques have gained significant relevance due to their high segmentation accuracy, even in complex scenarios, by leveraging large training and validation datasets [38]. Instance segmentation using deep learning can be implemented through 2 main approaches: 2-stage models (e.g., Mask R-CNN) and 1-stage models (e.g., You Look Only Once [YOLO], RetinaNet) [39]. While 2-stage models offer higher accuracy at the cost of lower speed and greater computational requirements, 1-stage models aim to balance accuracy and speed while using fewer resources [39]. Notably, recent advancements in YOLO models have significantly improved their accuracy while maintaining high-speed performance [36]. Nevertheless, training deep learning models from scratch requires large labeled datasets and substantial computational resources [40]. In this context, fine-tuning pretrained generalist models allows users to develop customized models without the need for extensive datasets [40, 41]. The integration of deep learning tools for labeling datasets has significantly accelerated this task [41, 42]. Moreover, new techniques such as slicing aided hyperinference (SAHI) have enhanced accuracy by reducing errors in the segmentation of small objects, making it particularly valuable for seed imagery [43]. The slicing process also reduces memory requirements for processing large images, maintaining high resolution without the need to resize them for model training and enabling the utilization of limited graphics processing unit (GPU) open resources (e.g., Google Colab).

In this work, we have developed a Python-based workflow to facilitate the development and deployment of custom deep learning models for measuring morphological and morphometric traits in fruit breeding programs. The main contribution of the workflow is the integration of preprocessing methods such as color and distortion correction, development and deployment of image segmentation fine-tuned generalist models, morphology measurements, and morphometric analyses. The workflow was successfully applied to phenotype multiple almond breeding populations, allowing for the estimation of broad-sense heritability values. Additionally, prediction models were developed to estimate kernel thickness based on area and weight. By implementing this workflow, the study has analyzed the largest number of individuals and data points ever recorded for the almond species to date.

## Material and Methods

### Plant material

The workflow was implemented to study breeding populations of the CEBAS-CSIC almond breeding program, located in the experimental field (Santomera, Murcia SE of Spain) during 2022 and 2023 seasons. In total, 665 unique genotypes from 6 F1 populations and a germplasm collection were studied (Table 1). Population parents were traditional cultivars (“Marcona” and “Desmayo Largueta”), released cultivars of CEBAS-CSIC (“Antoñeta,” “Penta,” “Tardona,” and “Florida”), and the breeding selections “R1000” (INRA, France) and “S4017” (CEBAS-CSIC). The target samples were shells and kernels of these almond descendants. For each genotype, 30 in-shell nuts and 30 kernels per year were sampled, photographed, and weighed, whenever available.

**Table 1:** Populations used in this study and the number of individuals studied per year

Year	2022		2023		Unique genotypes	
Family	Shell	Kernel	Shell	Kernel	Shell	Kernel
Germplasm collection	85	85	74	91	99	99
Antoñeta × Marcona	6	6	19	19	19	19
Antoñeta × Penta	161	161	142	142	183	183
Antoñeta × Tardona	57	57	56	53	71	70
Florida × Marcona	17	17	43	43	44	44
Desmayo × R1000	198	198	187	194	223	223
Marcona × S4017	0	0	26	27	26	27
Total	524	524	547	569	665	665

### Workflow description

A comprehensive workflow has been developed for preprocessing, segmentation model development, deployment, morphological measurements, and morphometric analyses, implemented through interactive Python notebooks (Supplementary Fig. S1) [44–47]. To optimize speed and performance, the use of a GPU is highly recommended. The workflow can be executed locally; however, it is also deployed on the Google Colab platform, enabling users to run it online and leverage free resources, such as GPUs. Although primarily written in Python, some functions integrate R for morphometric analyses, requiring R to be properly configured in the system’s path if executed locally.

### Color and distortion calibration

Color and distortion correction approaches are included in the preprocessing notebook (*1_Pre-processing_workflow.ipynb*). Color correction enables standardizing the dataset of the images using a color card as reference. Color correction functions implemented in the preprocessing notebook are based on the color correction module of the PlantCV Python library [34]. Distortion correction is implemented for possible radial and tangential distortion caused by the camera. Distortion correction functions are based on the OpenCV workflow for camera calibration [48]. It requires test patterns of chessboards with a known size of the squares to obtain a camera matrix that can be used subsequently with the pictures of the dataset. In addition, color and distortion correction functions are joined in the preprocessing notebook to deploy it in the whole dataset in 1 step.

### Auxiliary functions

Some optional auxiliary functions were included for the picture preprocessing, helping to separate the different samples in the pictures and getting the physical size of a pixel with reference objects in the picture (e.g., a coin with known diameter). These optional functions require training a model (next sections) to identify and segment the sample groups and the reference objects.

### Develop your segmentation model

The approach implemented in the present work intends to simplify the development of customized segmentation models. Four key steps are necessary for performing the development: slicing, labeling, “training,” and reconstruction (*2_Develop_your_segmentation_model_workflow.ipynb*). The slicing process consists of cropping pictures into smaller patches, with the size determined by the user. Using smaller patches of high-resolution images speeds up the training and prediction processes by reducing computational requirements [43]. Additionally, this approach improves the capture of fine details, as it avoids the loss of resolution and distortion caused by resizing the entire image [49, 50]. The slicing function defined in the workflow is divided into train, validation, and test datasets. The slices are obtained proportionally according to the user command to prepare for the next step. The second step, labeling, involves annotating which pixels correspond to the object of interest. For this task, the workflow was designed to accept as input the ZIP file generated by the image annotation platform CVAT in the YOLO Segmentation 1.0 format [42]. CVAT provides tools for semi-automatic segmentation using the Segment Anything Model (SAM) [41], which enables quick-instance segmentation with a single click. CVAT is available for free offline via a Docker container or online with a freemium model. The third step for developing the segmentation model is “training” the model. For that purpose, in the second notebook (*2_Develop_your_segmentation_model_workflow.ipynb*), the YOLO [51] pretrained algorithm series can be fine-tuned (although in their web mention, “training” really is a fine-tuning [52]) using our custom labeled dataset. The training process function enables controlling all the parameters of the training process (e.g., epochs, batch) and provides the results of the *YOLO.train* method. Result metrics include precision $( {{\mathrm{Precision}} = \frac{{{\mathrm{correctly}}\,{\mathrm{predicted}}\,{\mathrm{pixels}}}}{{{\mathrm{all}}\,{\mathrm{pixels}}\,{\mathrm{predicted}}\,{\mathrm{as}}\,{\mathrm{target}}}}} )$, which is the proportion of pixels predicted as belonging to the target class that are actually correct; recall $( {{\mathrm{Recall}} = \frac{{{\mathrm{correctly}}\,{\mathrm{predicted}}\,{\mathrm{pixels}}}}{{{\mathrm{all}}\,{\mathrm{pixels}}\,{\mathrm{belonging}}\,{\mathrm{to}}\,{\mathrm{the}}\,{\mathrm{targrt}}\,{\mathrm{class}}}}} )$, which is the proportion of pixels of the target class correctly identified by the model; F1-score $( {{\mathrm{F1}} = \frac{{2\,{\mathrm{Precision}}\,{\mathrm{Recall}}}}{{{\mathrm{Precision}}\,{\mathrm{ + }}\,{\mathrm{Recall}}}}} )$, which is the harmonic mean of precision and recall; intersection over union (IoU), which is the ratio between the area of overlap and the area of union of the predicted and true masks and is used internally to compute mean average precision but not reported directly; mean average precision @50 (mAP@50), defined as the average precision computed at an IoU threshold of 0.50; and mean average precision @50–95 (mAP@50–95), defined as the average precision averaged over IoU thresholds from 0.50 to 0.95 in steps of 0.05 [52, 53].

The last step for developing the segmentation model is to reconstruct the binary picture mask, for which 2 approaches were used. The first approach simply joins the patched binary masks after the prediction (*slice_predict_reconstruct*). The contours are detected using the *cv2.findcontours* OpenCV function [54] in the subsequent functions for morphology measurements. The watershed algorithm is implemented in such functions for separating touching objects of interest [55]. The second approach uses the SAHI pipeline (*predict_model_sahi*) [43], which is integrated in the YOLO segmentation process, and the instances segmented are identified specifically by providing their contours directly, solving also the problem of touching objects of interest. Here, the SAHI Python library is modified slightly to enable the *retina_mask* argument in the YOLO prediction function and provide fine details in the segmentations. A video tutorial describing the development of the model can be found in the workflow’s GitHub repository [44]. To assess the 2 reconstruction approaches, they were tested in the datasets studied, and the almonds with errors in the reconstruction were annotated and removed manually.

### Deploy your segmentation model for morphology and morphometric analyses

Once the segmentation model has been successfully “trained” and tested, it can be deployed for morphology and morphometric analyses. In the notebook for deployment (*3_Deploy_your_segmentation_model_worflow.ipynb*), 2 methods were prepared to measure.

The first method is general for any group of fruit, and the traits length (the longest axis of the fruit), width (the dimension perpendicular to the length), area (the surface enclosed by the fruit contour), perimeter (the length of the outer boundary), hull area (the area of the convex hull enclosing the shape), solidity (the ratio between area and hull area, reflecting compactness), aspect ratio (the ratio of length to width, indicating elongation), circularity (calculated as 4π × area/perimeter, with values closer to 1 indicating a more circular shape), ellipse ratio (the ratio between the minor and major axes of a fitted ellipse), and color (L*a*b* model) are measured for each shell/kernel. This general method can be used as a template and customized according to the user’s needs, for example, to study additional traits or different fruit shapes. The second method is specific for almonds adding more traits, such as width at 3 different heights (25%, 50%, and 75% of the length), vertical and horizontal symmetry, and symmetry in the top part of the almond (to measure the shoulder). In this specific method, almonds are aligned according to the angle of fitting an ellipse (*cv2.fitEllipse* function) and flipped vertically, placing the most distant point always in the lowest part (almond tip). Some extra traits derived from the results, together with the weight of the kernel/shell, such as the weight kernel/shell ratio, estimation of the thickness (only for kernels), and globosity (width/thickness), were also included. For the estimation of kernel thickness, a linear and quadratic model was fitted using 227 almond kernels, using the area and the weight as predictor variables. To validate the models, the dataset was split into 80% for training and 20% for validating, running it 100 times to assess the stability of the models.

Both methods export picture results, table results, and binary masks for morphometric analyses. Binary masks are flipped horizontally if the lowest pixel (tip) is the right part of the picture to avoid symmetric issues in morphometric analysis.

The morphometric analysis notebook (*4_Morphometrics_workflow.ipynb*) includes 2 morphometric approaches: elliptical Fourier analysis (EFA) and pixel-based principal component analysis (PCA). EFA is conducted using the Momocs v1.4.1 R package [52], but it is implemented in the notebook using Python, via a subprocess. Several functions are included for EFA: exploratory analysis, running the EFA, performing a PCA on the EFA results, and *kmeans* clustering. PCA performed on EFA coefficients generates as traits the components (EF-PCs) that explain the variability of the shape. Regarding the second pixel, pixel-based PCA, binary masks are flattened for performing the PCA and generating also PCs as traits (PB-PCs) [15]. Result plots of both approaches can be obtained to show the influence of each trait.

### RGB imaging system

For image acquisition, group pictures of various samples were captured over a black surface template, with the samples positioned inside painted rectangles. For the 2022 dataset, images were taken using a Canon EOS 70D camera, achieving a resolution of 6 px/mm. In 2023, the imaging system was redefined due to objectives beyond the scope of this work, utilizing an Arducam 8MP IMX219 camera, which resulted in images with a resolution of 4 px/mm. Both systems were illuminated using 2 white LED light sources.

### Heritability

Broad-sense heritability (H^2^) was estimated using the Python library *statsmodels* [53], fitting a linear mixed model using the genotype as random effects and the year as fixed effects (Equations 1 and 2).


(1) \begin{eqnarray*}
{{{\mathrm{Y}}}_{{\mathrm{ijk}}}} = {\mathrm{\mu }} + {{{\mathrm{G}}}_{\mathrm{i}}} + {{{\mathrm{Y}}}_{\mathrm{j}}} + {{{\mathrm{\varepsilon }}}_{{\mathrm{ijk}}}}
\end{eqnarray*}

where:

Y_ijk_: Observed phenotypic value for the *k*th observation of the *i*th genotype in the *j*th year

μ: Overall population mean

G_i_: Random effect of the *i*th genotype

Y_j_: Fixed effect of the *j*th year

ε_ijk_: Residual error term


(2) \begin{eqnarray*}
\frac{{{{\sigma }^2}G}}{{{{\sigma }^2}G + {{\sigma }^2}e}}
\end{eqnarray*}

Where:

σ^2^G: Genetic variance

σ^2^e: Residual variance

### Impact on almond breeding

To study the impact of this new tool on almond breeding, a comprehensive comparison with existing scientific articles related to quantitative almond morphology phenotyping was conducted. Several parameters, such as the number of individuals studied, number of shells/kernels phenotyped, and the number of data points obtained, were compared to evaluate the benefits of the new high-throughput phenotyping tool.

## Results

### YOLO fine-tuned segmentation models performance and reconstruction errors

**Figure 1:**
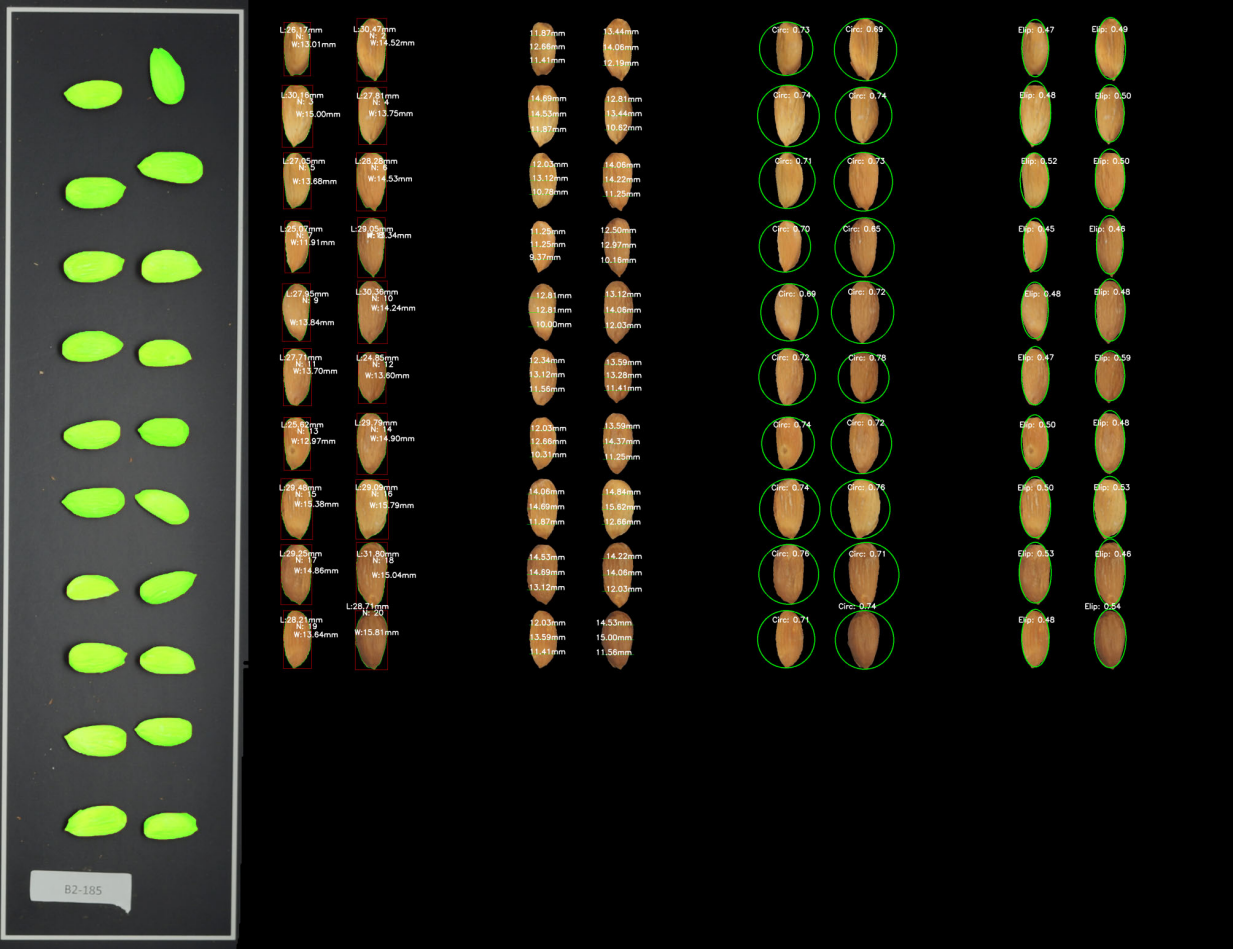
Images generated by the workflow, displayed from left to right: original image with masks, length and width measurements, widths at different lengths, circularity, and ellipse ratio.

A total of 46,729 elements (shell and kernels) were segmented (using the watershed approach for subsequent steps), and performance errors from both reconstruction approaches were annotated (Table 2). A standard image generated by the workflow is shown in Fig. 1. Four segmentation models were fine-tuned using datasets from the kernel and shell in 2022 and 2023. The pretrained model “yolov11s-seg.pt” [59] was selected, and fine-tuning was performed over 100 epochs (i.e., complete passes through the entire training dataset), using 500–1,000 image slices of 320 pixels each, split into 60% for training, 20% for validation, and 20% for testing. Metrics plots from the fine-tuning process of the datasets are shown as an example in Supplementary Fig. S2. In general, training and validation loss plots exhibited a consistent decrease in the process, with no evidence of overfitting. Precision and recall exceeded 0.95 early in training, and the F1-score was around 0.99 in all the models. The mAP@0.50 reached ∼1.0, while the more stringent mAP@50–95 stabilized later in training but consistently remained above 0.9. Validation masks were explored, showing precise results. All the combinations (dataset + reconstruction approach) showed errors lower than 2%, except for the shell 2022 dataset using SAHI (2.93%) (errors were considered when an almond was incorrectly reconstructed, not detected, or if it was something other than a target sample).

**Table 2:** Performance for different datasets and reconstruction methods, including reconstruction errors

Method	Dataset	Errors	Total elements	Total phenotyped elements	Error percentage
*Slice_predict_reconstruct* + Watershed	Kernel-2022	5	10,184	10,180	0.05%
	Kernel-2023	264	15,568	15,304	1.70%
	Shell-2022	4	10,364	10,360	0.04%
	Shell-2023	60	10,945	10,885	0.55%
SAHI	Kernel-2022	14	10,184	10,178	0.14%
	Kernel-2023	139	15,568	15,429	0.89%
	Shell-2022	304	10,364	10,288	2.93%
	Shell-2023	44	10,945	10,902	0.40%

### Thickness estimation

**Figure 2:**
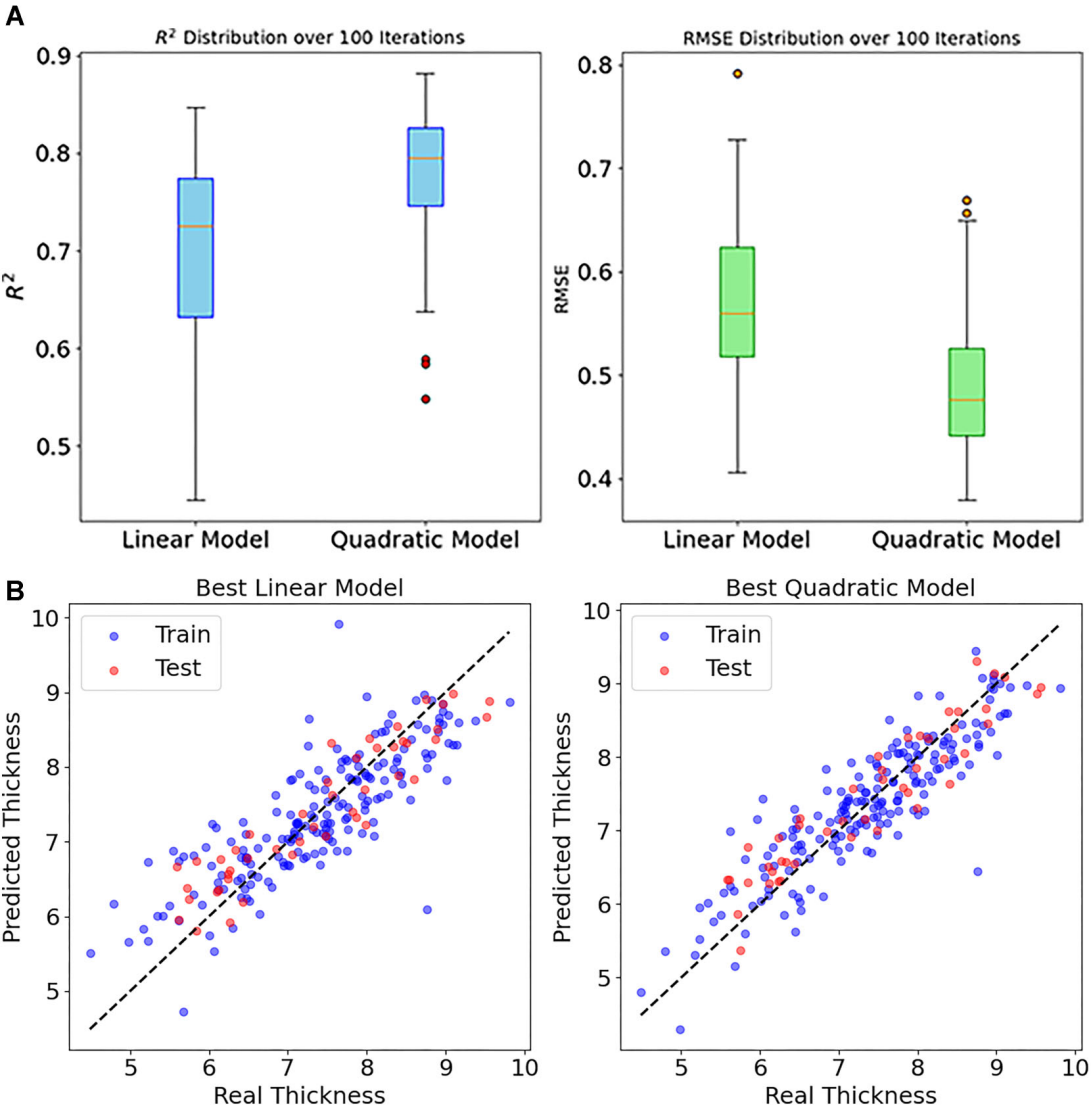
Results of the kernel thickness modeling, showing the *R*² (A, left) and RMSE (A, right) over 100 iterations, along with scatterplots for the best linear (B, left) and quadratic (B, right) models.

Kernel thickness was modeled using weight and area as predictor variables, employing both linear and quadratic models. The median *R*² in the test dataset for the linear model was 0.725, while for the quadratic model, it was 0.795. The corresponding median root mean squared error (RMSE) values were 0.56 for the linear model and 0.47 for the quadratic model (Fig. 2A). The best-performing linear and quadratic models were then plotted, achieving *R*² values of 0.847 and 0.8815, respectively, and RMSE values of 0.451 and 0.381, respectively (Fig. 2B). The quadratic model, trained with the complete dataset, was employed for estimating thickness across all samples in subsequent analysis.

### Morphometric results

**Figure 3:**
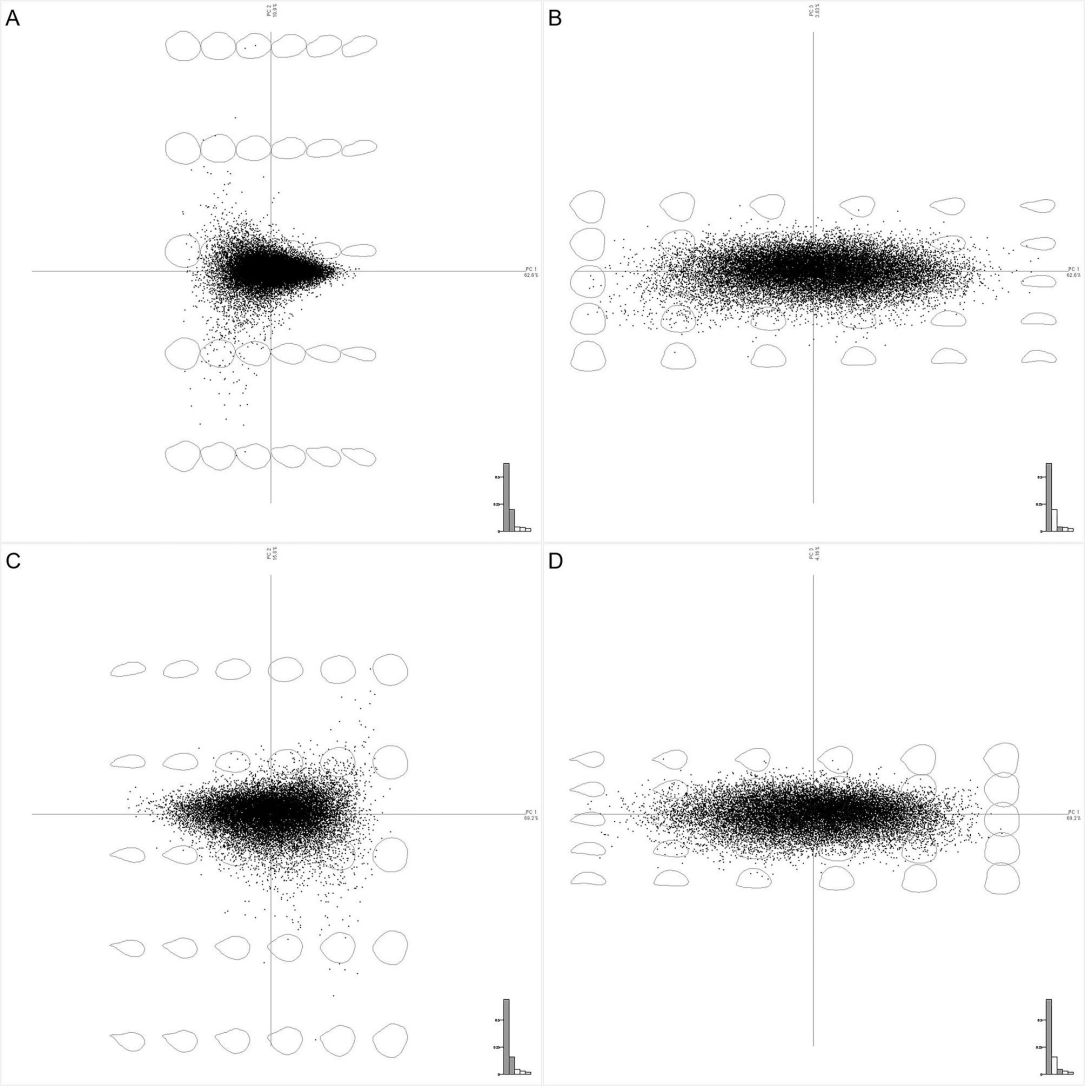
EFA-PCA scatterplot results for the kernel (A) EF-PC1/EF-PC2 and (B) EF-PC1/EF-PC3 and shell (C) EF-PC1/EF-PC2 and (D) EF-PC1/EF-PC3 datasets. The influence of the PCs on shape is represented in the plots.

**Figure 4:**
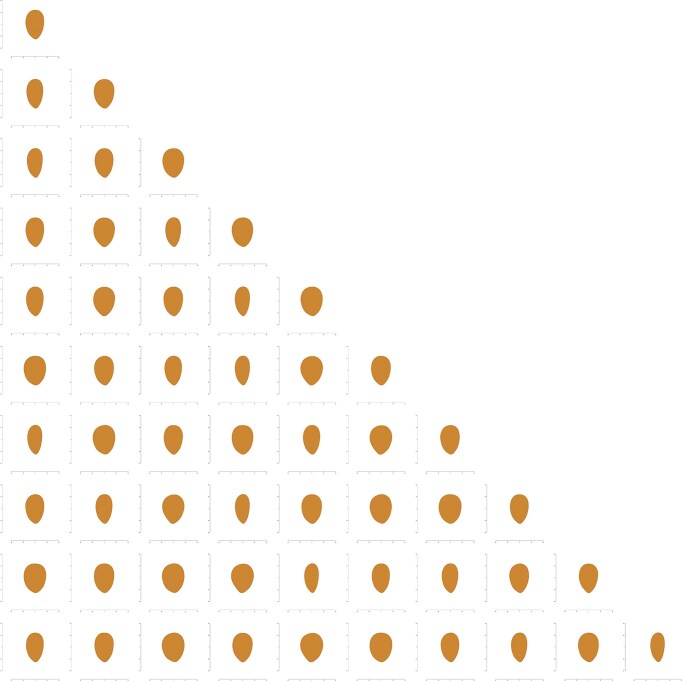
K-means clustering results using EFA-PCA in the kernel dataset, showing the shapes corresponding to each centroid for scenarios ranging from *k* = 1 to *k* = 10.

EFA was conducted using all the kernel and shell binary masks (separately), placing 10 harmonics in both datasets after a visual exploration analysis. PCA was performed on the EFA coefficients, with 62.63%, 19.93%, and 3.83% explained by the first 3 PCs for the kernel analysis, and 69.19%, 15.75%, and 4.15% explained by the first 3 PCs for the shell analysis (Fig. 3 and Supplementary Fig. S3). Only the first 6 components of each dataset were used for subsequent analysis because they explained at least 1% of the variability. K-means clustering from *k* = 1 to *k* = 10 was run to show the main groups of the datasets (Fig. 4 and Supplementary Fig. S4). The optimal number of clusters was studied, and no abrupt changes in the slope by the elbow method were observed in any dataset (Supplementary Fig. S5).

**Figure 5:**
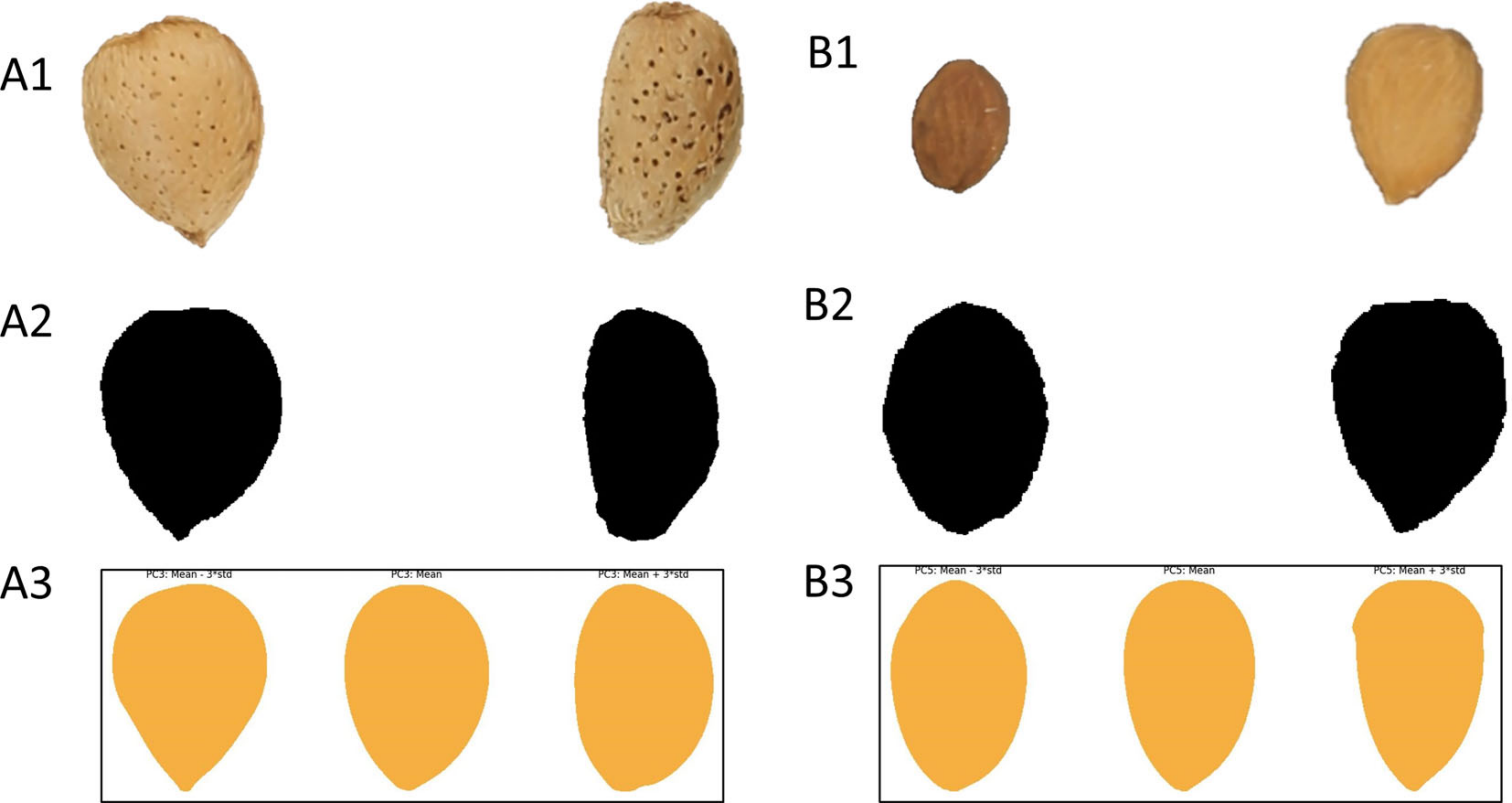
Example images for shell PB-PC3 (A) and kernel PB-PC5 (B) morphometric traits, showing 2 contrasting phenotypes for each trait. Panels A1 and B1 show the original almond images, A2 and B2 show the corresponding binary masks, and A3 and B3 display the pixel-based PCA traits from the mean shape to ±3 standard deviations.

**Figure 6:**
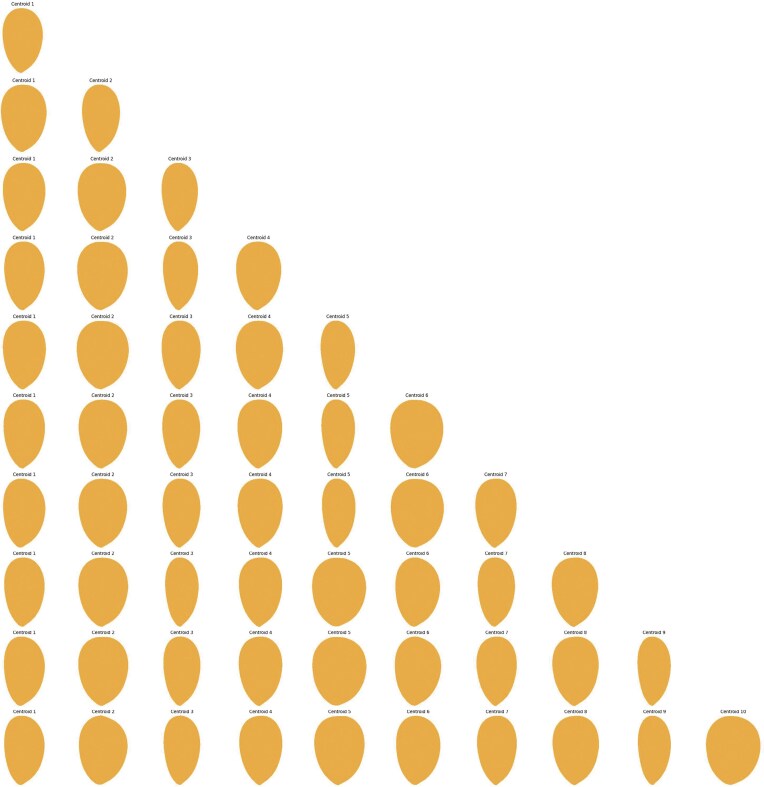
K-means clustering results using PB-PCA in the kernel dataset, showing the shapes corresponding to each centroid for scenarios ranging from *k* = 1 to *k* = 10.

Pixel-based PCA was performed in kernel and shell datasets, obtaining 34.96%, 8.78%, and 4.75% variance explained by the first 3 PCs in the kernel dataset and 37.72%, 9.19%, and 4.96% in the shell dataset (Supplementary Fig. S6). The PCs’ influence in the shape was studied by plotting the ±3× deviation from the mean shape (Fig. 5 and Supplementary Fig. S7). Ten PB-PCs were collected for each dataset, explaining at least 1% of the variance for each one. K-means clustering was also carried out from *k* = 1 to *k* = 10 (Fig. 6 and Supplementary Fig. S8), and no abrupt changes were observed by the elbow method to determine the optimal number of clusters (Supplementary Fig. S9).

### Correlations

**Figure 7:**
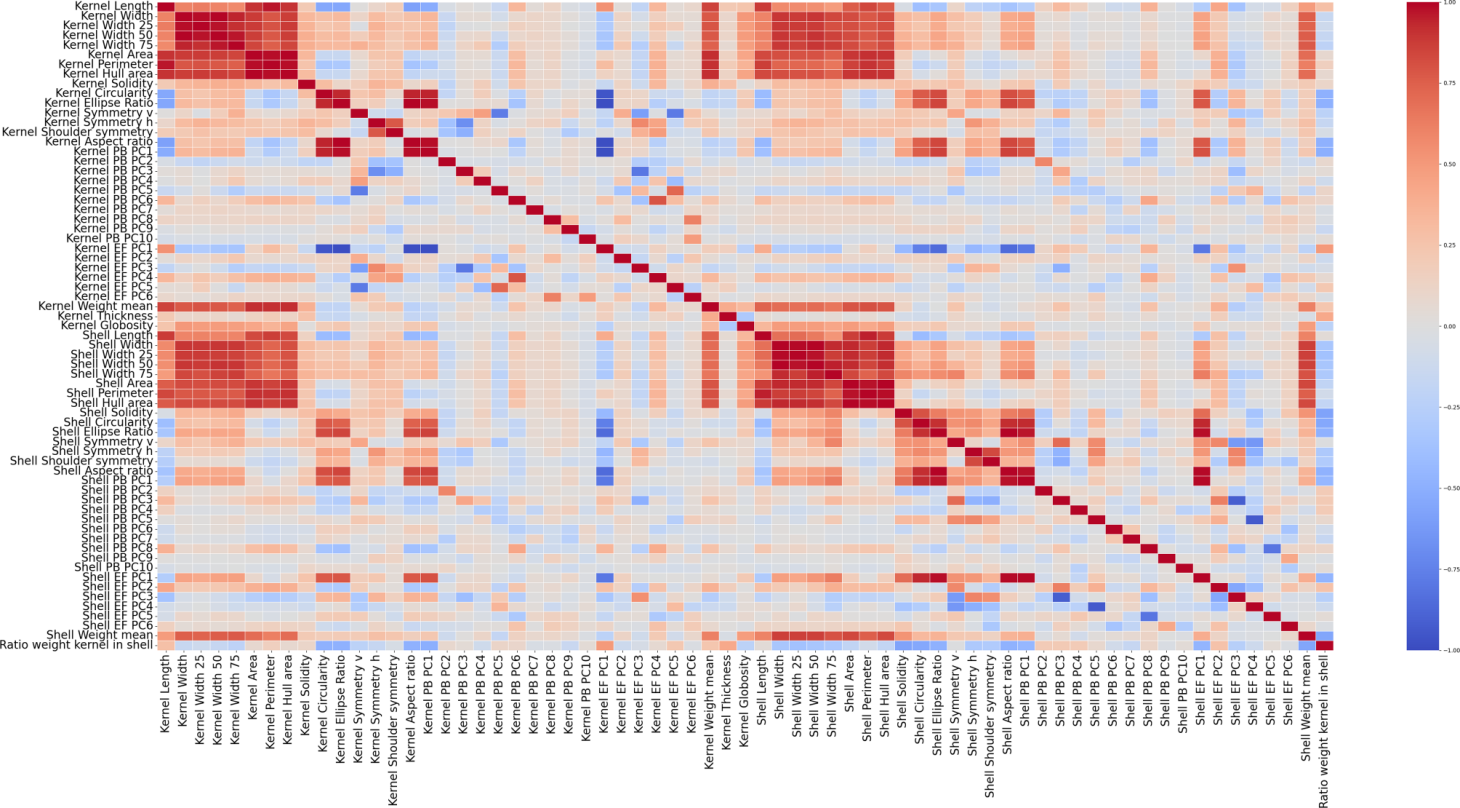
Heatmap of the Pearson correlations between all morphological and morphometric traits.

To analyze and interpret the phenotype data obtained, the Pearson correlation between all morphological and morphometric traits was calculated (Fig. 7). In absolute value, 340 pairwise correlations were higher than 0.75, with the traits of shell almond area, hull area, and perimeter exhibiting the highest number of strong pairwise correlations. High correlations (greater than 0.95) were observed between kernel and shell morphometric traits across different approaches, such as EF-PC1 with PB-PC1. Additionally, strong correlations were found between these 2 PC1 components and morphological traits such as aspect ratio and ellipse ratio.

### Heritability

**Figure 8:**
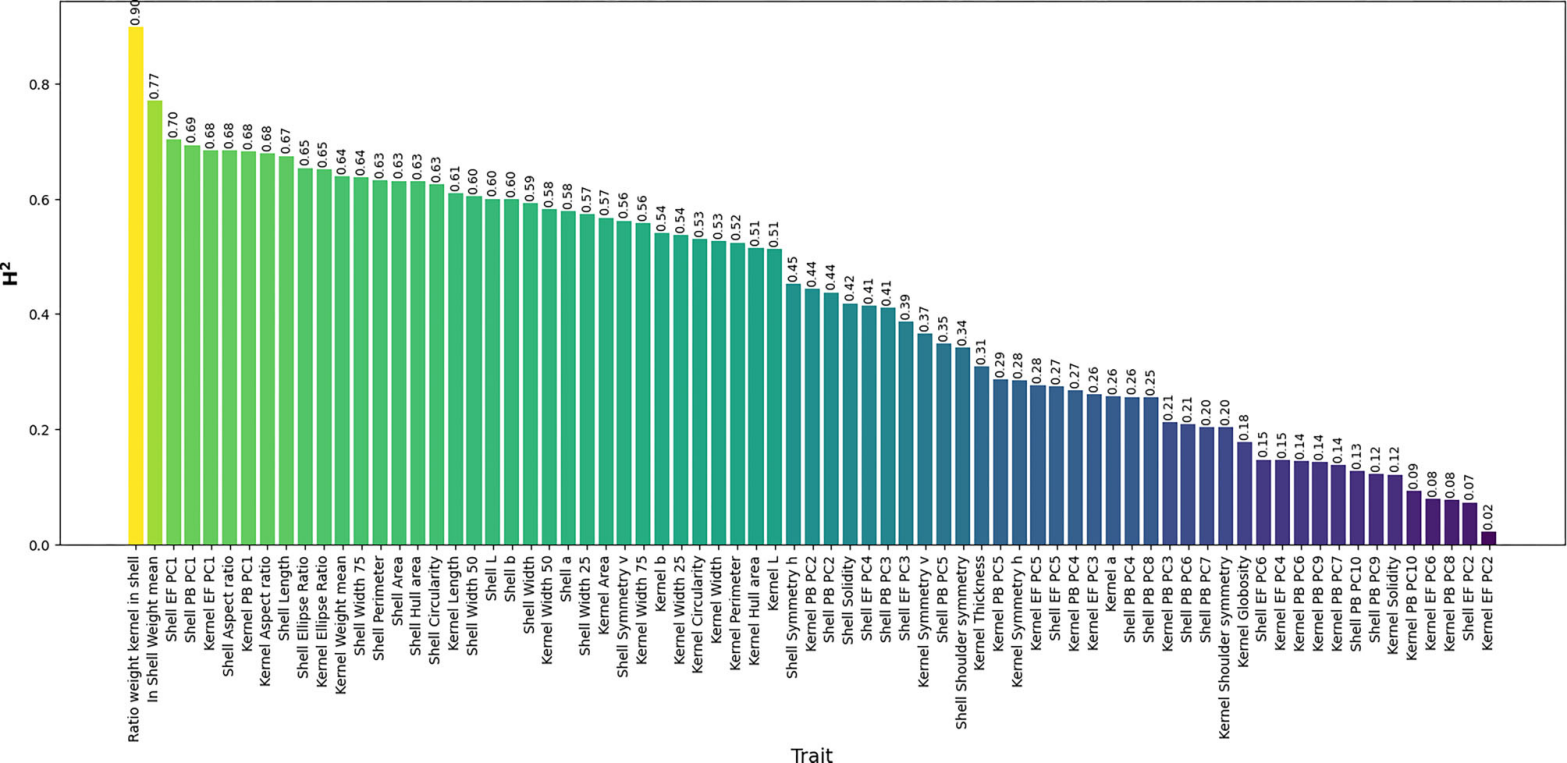
Broad-sense heritability of all traits studied in the almond populations.

Broad-sense heritability (H^2^) was estimated for all the traits studied here (Fig. 8). Fifty-five of the 73 traits showed heritabilities ≥0.25. Weight ratio kernel/shell, shell weight, and shell EF-PC1 showed the highest heritability values (0.9, 0.77, and 0.70, respectively). Between morphometric traits, those related to PC1 showed high heritability values (higher than 0.68), and others, such as kernel PB-PC2, shell PB-PC2, shell EF-PC4, and shell PB-PC3, showed medium values (higher than 0.3).

### Impact on almond breeding

**Figure 9:**
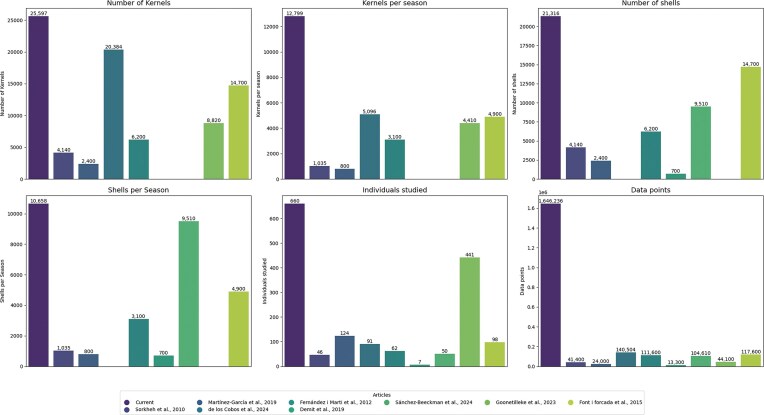
Bar plot for comparison among articles related to quantitative almond morphology traits [19, 29, 55–64].

Nine scientific articles related to almond morphology and breeding were collected to compare the performance of the high-throughput approach implemented in the current study (Supplementary Table S1 and Fig. 9). A total of 25,597 kernels were studied (an average of 12,799 per season), along with 21,316 shells (an average of 10,658 per season) (Fig. 9). The entire dataset comprised 660 individuals, and 1,579,443 data points were collected (Fig. 9).

## Discussion

The comprehensive workflow presented here introduces a new high-throughput phenotyping tool using almonds as a case of study for fruits. This approach has enabled the analysis of 665 genotypes—the largest dataset for almond morphological quantitatively measured traits reported to date, to the best of the authors’ knowledge. The development of this new phenotyping tool represents significant progress in addressing the phenotyping bottleneck in almond breeding programs. The developed methods were adjusted to the target samples used but can be adapted to phenotype other kinds of fruits and other types of organs, such as leaves or roots. Furthermore, as it is open source, it can be used by other plants breeding programs. In fact, the workflow was successfully applied to publicly available image datasets of apples and strawberries [13, 15,65–67]. Example results can be found in the workflow’s GitHub repository: https://github.com/jorgemasgomez/almondcv2.

Clearly, recent advancements in artificial intelligence (AI) segmentation models, such as YOLO [47] and SAM [41], enable breeding programs to develop fine-tuned models for specific applications, even without large datasets. Additionally, progress in labeling tools like CVAT [42], which integrate AI-assisted features, accelerates the tedious and time-consuming labeling process. The new workflow designed here uses Jupyter notebooks for ease of customization, eliminating the need for high-performance hardware (e.g., GPUs), allowing users to run the tool efficiently in cloud-based environments such as Google Colab. Segmentation model results demonstrated strong accuracy across all developed models, as measured by F1-score, precision, recall, and mAP metrics. Moreover, the reconstruction approaches employed showed good performance in general terms. A lower performance was noticed only in the shell 2022 using SAHI, which may be improved with SAHI parameter optimization.

The thickness estimation models demonstrated high accuracy, highlighting the usefulness of combining imaging phenotyping tools with traditional phenotyping processes, such as weighing [68]. This approach enhances the phenotyping process by offering an alternative to capturing transversal pictures and makes phenotyping more accessible without relying on more complex instruments like 3D cameras or approaches such as 3D reconstruction [17, 63]. However, the approach employed takes advantage of the similar density among kernels, but it would not be effective for shell nuts due to the variability in shell hardness and density.

Morphometric analyses provided new quantitative traits for almond shape breeding. Both EFA and pixel-based PCA showed a large variance explained by the PC1 in kernel and shell datasets. A high correlation between the different PCs1 and aspect ratio traits was identified and can also be observed graphically (Fig. 3 and Supplementary Fig. S7). Indeed, aspect ratio has been highly correlated with PCs1 in tomato and apple leaves [69, 70], pear fruit [71], walnut [59], and a shape discriminator almond nut [21]. Although the interpretation of some morphometric traits could be abstract, patterns in different parts of the shape could be observed, as in the tip (e.g., kernel PB-PC4), top (e.g., kernel PB-PC5), and side curvature (e.g., kernel EF-PC3). Further interpretation of these traits could be implemented by using visual guidance through text models [72]. In particular, a recent study highlighted the potential role of almond shape in breakage during processing [28], which indicates that morphology and morphometric analyses could play an important role in future research, with clear and easy interpretation being necessary. Moreover, some morphometric traits (apart from those related to aspect ratio) showed medium heritability values (>0.3), which could place them as breeding targets. In general, all the quantitative traits extracted can be incorporated into genomic selection schemes, achieving higher accuracy than with classical shape descriptors and allowing weighted importance in selection through a selection index [8]. This approach facilitates the development of breeding populations with target kernel size and shape tailored to the requirements of the intended end use [19–21].

The number of kernels and nuts analyzed in this comparative study is considerably higher than in previous research, and the difference becomes even more pronounced when comparing metrics per season. Data points collected were from 11 to 123 times higher than those reported in other works [19, 29, 59–64]. More importantly, this progress has been achieved while reducing both time and economic resources, as a single person was able to phenotype the entire dataset for 1 season in just 2 weeks. Currently, shell cracking remains the primary bottleneck due to the manual process, which is challenging to automate because of variability in shell hardness and size. This extensive dataset will facilitate future studies aimed at dissecting quantitative traits and implementing genomic selection approaches.

## Availability of Source Code and Requirements

Project name: AlmondCV

Project homepage: https://github.com/jorgemasgomez/almondcv2

Operating system(s): Platform independent

Programming language: Python, R

Other requirements: see public environment file released under GNU GPL v3

RRID: SCR_027064

WorkflowHub: https://workflowhub.eu/workflows/1731

Bio.tools: https://bio.tools/almondcv2

## Additional Files

**Supplementary Table S1**. Article metrics studied related to quantitative almond morphological traits.

**Supplementary Fig. S1**. Workflow description outlining the steps involved in developing the segmentation model (green) and deploying it (purple).

**Supplementary Fig. S2**. YOLO metrics obtained during the fine-tuning process for Kernel-2022 (A), Shell-2022 (B), Kernel-2023 (C), and Shell-2023 (D) datasets. A description of the metrics can be found in Ultralytics (2025).

**Supplementary Fig. S3**. Explained variance (%) per EF-PC in the kernel (left) and shell (right) datasets.

**Supplementary Fig. S4**. K-means clustering using EFA-PCA results in the shell dataset, showing the shapes corresponding to each centroid for scenarios ranging from *k* = 1 to *k* = 10.

**Supplementary Fig. S5**. Within-group sum of squares decay for K-means clustering using EFA-PCA results in the kernel (left) and shell (right) datasets.

**Supplementary Fig. S6**. Explained variance (%) per PB-PC in the kernel (left) and shell (right) datasets.

**Supplementary Fig. S7**. Representation of the influence of the PB-PCs on shape in the kernel (left) and shell (right) datasets, from the mean shape to ±3× standard deviation.

**Supplementary Fig. S8**. K-means clustering using PB-PCA results in the shell dataset, showing the shapes corresponding to each centroid for scenarios ranging from *k* = 1 to *k* = 10.

**Supplementary Fig. S9**. Within-group sum of squares decay for K-means clustering using PB-PCA results in the kernel (left) and shell (right) datasets.

## Abbreviations

AI: artificial intelligence; EFA: elliptical Fourier analysis; GPU: graphics processing unit; H2: broad sense heritability; IoU: intersection over union; PCA: principal component analysis; QTL: quantitative trait loci; RMSE: root mean squared error; ROI: region of interest; SAHI: slicing aided hyperinference; SAM: Segment Anything Model; YOLO: You Only Look Once.

## Supplementary Material

giaf157_Supplemental_Files

giaf157_Authors_Response_To_Reviewer_Comments_original_submission

giaf157_Authors_Response_To_Reviewer_Comments_revision_1

giaf157_GIGA-D-25-00218_Original_Submission

giaf157_GIGA-D-25-00218_Revision_1

giaf157_GIGA-D-25-00218_Revision_2

giaf157_Reviewer_1_Report_original_submission Yuvraj Chopra -- 7/1/2025

giaf157_Reviewer_2_Report_original_submission Qi Wang -- 7/1/2025

giaf157_Reviewer_2_Report_Revision_1 Qi Wang -- 8/29/2025

giaf157_Reviewer_3_Report_original_submission Yu Jiang -- 7/29/2025

## Data Availability

The data supporting the results of this article are available in the Zenodo repository [73]. All additional supporting data are available in the *GigaScience* repository, GigaDB [74] .

## References

[1] Yang W, Feng H, Zhang X, et al. Crop phenomics and high-throughput phenotyping: past decades, current challenges, and future perspectives. Mol Plant. 2020;13:187–214. 10.1016/j.molp.2020.01.008. 31981735

[2] Moore C R, Johnson L S, Kwak I-Y, et al. High-throughput computer vision introduces the time axis to a quantitative trait map of a plant growth response. Genetics. 2013;195:1077–86. 10.1534/genetics.113.153346. 23979570 PMC3813838

[3] Sheikh M, Iqra F, Ambreen H, et al. Integrating artificial intelligence and high-throughput phenotyping for crop improvement. J Integr Agric. 2024;23:1787–802. 10.1016/j.jia.2023.10.019.

[4] Condorelli G E, Maccaferri M, Newcomb M, et al. Comparative aerial and ground based high throughput phenotyping for the genetic dissection of NDVI as a proxy for drought adaptive traits in Durum wheat. Front Plant Sci. 2018;9:893. 10.3389/fpls.2018.00893. 29997645 PMC6028805

[5] Azevedo C F, Ferrão LFV, Benevenuto J, et al. Using visual scores for genomic prediction of complex traits in breeding programs. Theor Appl Genet. 2024;137:9. 10.1007/s00122-023-04512-w. 38102495

[6] Gorjanc G, Cleveland M A, Houston R D, et al. Potential of genotyping-by-sequencing for genomic selection in livestock populations. Genet Sel Evol. 2015;47:12. 10.1186/s12711-015-0102-z. 25887531 PMC4344748

[7] Gorjanc G, Dumasy J-F, Gonen S, et al. Potential of low-coverage genotyping-by-sequencing and imputation for cost-effective genomic selection in biparental segregating populations. Crop Sci. 2017;57:1404–20. 10.2135/cropsci2016.08.0675.

[8] Kizilkaya K, Fernando R L, Garrick D J. Reduction in accuracy of genomic prediction for ordered categorical data compared to continuous observations. Genet Sel Evol. 2014;46:37. 10.1186/1297-9686-46-37. 24912924 PMC4094927

[9] Singh A, Ganapathysubramanian B, Singh A K, et al. Machine learning for high-throughput stress phenotyping in plants. Trends Plant Sci. 2016;21:110–24. 10.1016/j.tplants.2015.10.015. 26651918

[10] Kim J Y . Roadmap to high throughput phenotyping for plant breeding. J Biosyst Eng. 2020;45:43–55. 10.1007/s42853-020-00043-0.

[11] Huang Y, Ren Z, Li D, et al. Phenotypic techniques and applications in fruit trees: a review. Plant Methods. 2020;16:107. 10.1186/s13007-020-00649-7. 32782454 PMC7412798

[12] Kirchgessner N, Hodel M, Studer B, et al. FruitPhenoBox—a device for rapid and automated fruit phenotyping of small sample sizes. Plant Methods. 2024;20:74. 10.1186/s13007-024-01206-2. 38783345 PMC11112871

[13] Keller B, Jung M, Bühlmann-Schütz S, et al. The genetic basis of apple shape and size unraveled by digital phenotyping. G3 (Bethesda). 2024;14:jkae045. 10.1093/g3journal/jkae045. PMC11075547 38441135

[14] Jurado-Ruiz F, Rousseau D, Botía J A, et al. GenoDrawing: an autoencoder framework for image prediction from SNP markers. Plant Phenomics. 2023;5:0113. 10.34133/plantphenomics.0113. 38239740 PMC10795539

[15] Feldmann M J, Hardigan M A, Famula R A, et al. Multi-dimensional machine learning approaches for fruit shape phenotyping in strawberry. Gigascience. 2020;9:giaa030. 10.1093/gigascience/giaa030. PMC7191992 32352533

[16] Zingaretti L M, Monfort A, Pérez-Enciso M. Automatic fruit morphology phenome and genetic analysis: an application in the octoploid strawberry. Plant Phenomics. 2021;2021:9812910. 10.34133/2021/9812910. 34056620 PMC8139333

[17] Feldmann M J, Tabb A. Cost-effective, high-throughput phenotyping system for 3D reconstruction of fruit form. Plant Phenome J. 2022;5:e20029. 10.1002/ppj2.20029.

[18] Janick J, Schirra M. Postharvest technology and utilization of almonds. Horticult Rev. 1997. 10.1002/9780470650646.

[19] Martínez-García P J, Rubio M, Cremades T, et al. Inheritance of shell and kernel shape in almond (Prunus dulcis). Sci Hortic. 2019;244:330–38. 10.1016/j.scienta.2018.09.041.

[20] Socias R, Kodad O, Alonso J, et al. Horticultural Reviews. In: Almond quality: a breeding perspective. John Wiley & Sons, Inc 2008:197.

[21] Demir B, Sayinci B, Çetin N, et al. Shape discrimination of almond cultivars by elliptic Fourier descriptors. Erwerbs Obstbau. 2019;61:245–56. 10.1007/s10341-019-00423-7.

[22] Verdú A, Izquierdo S, Socias i Company R. Processing and industrialization. In: Almonds: botany, production and uses. CABI. 2017:460–481.

[23] Klingenberg C P, Debat V, Roff D A. Quantitative genetics of shape in cricket wings: developmental integration in a functional structure. Evolution. 2010;64:2935–51. 10.1111/j.1558-5646.2010.01030.x. 20482613

[24] Claes P, Shriver M D. Establishing a multidisciplinary context for modeling 3D facial shape from DNA. PLoS Genet. 2014;10:e1004725. 10.1371/journal.pgen.1004725. 25375363 PMC4222671

[25] Dryden I L, Mardia K V. Statistical Shape Analysis, with Applications in R. Wiley: UK. 2016:1–470.

[26] Altuntas E, Gercekcioglu R, Kaya C. Selected mechanical and geometric properties of different almond cultivars. Int J Food Prop. 2010;13:282–93. 10.1080/10942910802331504.

[27] Koyuncu M A, Ekinci K, Gun A. The effects of altitude on fruit quality and compression load for cracking of walnuts (Juglans regia L.). J Food Qual. 2004;27:407–17. 10.1111/j.1745-4557.2004.00689.x.

[28] Lipan L, Miarnau X, Cutrone M, et al. Impact of industrial shelling and blanching on almond kernel integrity and color. LWT. 2025;218:117472. 10.1016/j.lwt.2025.117472.

[29] Pérez de Los Cobos F, Romero A, Lipan L, et al. QTL mapping of almond kernel quality traits in the F1 progeny of “Marcona” × “Marinada.” Front Plant Sci. 2024;15:1504198. 10.3389/fpls.2024.1504198. 39665108 PMC11631582

[30] Vidyarthi S K, Tiwari R, Singh S K. Stack ensembled model to measure size and mass of almond kernels. J Food Process Eng. 2020;43:e13374. 10.1111/jfpe.13374.

[31] Zhu F, Paul P, Hussain W, et al. SeedExtractor: an open-source GUI for seed image analysis. Front Plant Sci. 2021;11:581546. 10.3389/fpls.2020.581546. 33597957 PMC7882627

[32] Whan A P, Smith A B, Cavanagh C R, et al. GrainScan: a low cost, fast method for grain size and colour measurements. Plant Methods. 2014;10:23. 10.1186/1746-4811-10-23. 25050131 PMC4105244

[33] Tanabata T, Shibaya T, Hori K, et al. SmartGrain: high-throughput phenotyping software for measuring seed shape through image analysis. Plant Physiol. 2012;160:1871–80. 10.1104/pp.112.205120. 23054566 PMC3510117

[34] Gehan M A, Fahlgren N, Abbasi A, et al. PlantCV v2: image analysis software for high-throughput plant phenotyping. PeerJ PeerJ Inc. 2017;5:e4088. 10.7717/peerj.4088. PMC5713628 29209576

[35] Ngugi L C, Abelwahab M, Abo-Zahhad M. Recent advances in image processing techniques for automated leaf pest and disease recognition—a review. Inform Process Agricult. 2021;8:27–51. 10.1016/j.inpa.2020.04.004.

[36] Sapkota R, Ahmed D, Karkee M. Comparing YOLOv8 and Mask R-CNN for instance segmentation in complex orchard environments. Artif Intell Agricult. 2024;13:84–99. 10.1016/j.aiia.2024.07.001.

[37] Xue W, Ding H, Jin T, et al. CucumberAI: cucumber fruit morphology identification system based on artificial intelligence. Plant Phenomics. 2024;6:0193. 10.34133/plantphenomics.0193. 39144674 PMC11324094

[38] Katal N, Rzanny M, Mäder P, et al. Deep learning in plant phenological research: a systematic literature review. Front Plant Sci. 2022;13:805738. 10.3389/fpls.2022.805738. PMC8969581 35371160

[39] Gu W, Bai S, Kong L. A review on 2D instance segmentation based on deep neural networks. Image Vision Comput. 2022;120:104401. 10.1016/j.imavis.2022.104401.

[40] Bommasani R, Hudson D A, Adeli E, et al. On the opportunities and risks of foundation models. arXiv. 2021. 10.48550/arXiv.2108.07258. Accesed June 2025.

[41] Kirillov A, Mintun E, Ravi N, et al. Segment anything. In: IEEE/CVF International Conference on Computer Vision (ICCV). IEEE/CVF International Conference on Computer Vision: Paris, France. 2023. 10.1109/ICCV51070.2023.00371.

[42] Sekachev B, Manovich N, Zhiltsov M, et al. Opencv/cvat: v1.1.0. Zenodo. 2020. 10.5281/zenodo.3753255. Accesed June 2025.

[43] Akyon F C, Onur Altinuc S, Temizel A. Slicing aided hyper inference and fine-tuning for small object detection. In: IEEE International Conference on Image Processing (ICIP). IEEE International Conference on Image Processing (ICIP): Bordeaux, France. 2022.

[44] Mas-Gómez J, Rubio M, Dicenta F, et al. Open RGB imag- ing workflow for morphological and morphometric analysis. GitHub Repository. 2025. https://github.com/jorgemasgomez/almondcv2. 10.1093/gigascience/giaf157 PMC12970599 41416705

[45] Mas-Gómez J, Rubio M, Dicenta F, et al. almondcv2: open RGB imaging workflow for morphological and morphometric anal- ysis. bio.tools. 2025. https://bio.tools/almondcv2. 10.1093/gigascience/giaf157 PMC12970599 41416705

[46] Mas-Gómez J, Rubio M, Dicenta F, et al. almondcv2 Workflow. SciCrunch Registry (RRID:SCR_027064 ). 2025. 10.1093/gigascience/giaf157 PMC12970599 41416705

[47] Mas-Gómez J, Rubio M, Dicenta F, et al. almondcv2: open RGB imaging workflow for morphological and morphometric analysis. WorkflowHub. 2025. https://workflowhub.eu/workflows/1731. 10.1093/gigascience/giaf157 PMC12970599 41416705

[48] OpenCV: Camera Calibration. 2025. https://docs.opencv.org/4.x/dc/dbb/tutorial_py_calibration.html. Accessed 31 January 2025.

[49] Pereira A, Santos C, Aguiar M, et al. Detection of retinal microlesions through YOLOR-CSP architecture and image slicing with the SAHI algorithm. International Joint Conference on Neural Networks (IJCNN): Gold Coast, Australia. 2023. 10.1109/IJCNN54540.2023.10191623.

[50] Saradopoulos I, Potamitis I, Rigakis I, et al. Image augmentation using both background extraction and the SAHI approach in the context of vision-based insect localization and counting. Information. 2025;16:10. 10.3390/info16010010.

[51] Redmon J, Divvala S, Girshick R, et al. You only look once: unified, real-time object detection. In: IEEE Conference on Computer Vision and Pattern Recognition. IEEE: Las Vegas, NV, USA. 2016. https://doi.org.10.1109/CVPR.2016.91.

[52] Train—Ultralytics YOLO Docs. 2025. https://docs.ultralytics.com/es/modes/train/. Accessed 31 January 2025.

[53] Yu Y, Wu X, Yu P, et al. Location-guided lesions representation learning via image generation for assessing plant leaf diseases severity. Plant Phenomics. 2025;7:100058. 10.1016/j.plaphe.2025.100058. 41415163 PMC12709949

[54] Bradski G, Kaehler A. The OpenCV Library. Dr Dobbs J Softw Tools. 2000. https://github.com/opencv/opencv/wiki/CiteOpenCV.

[55] OpenCV: Image Segmentation with Watershed Algorithm. 2025. https://docs.opencv.org/4.x/d3/db4/tutorial_py_watershed.html. Accessed 31 January 2025.

[56] Bonhomme V, Picq S, Gaucherel C, et al. Momocs: outline analysis using R. J Stat Soft. 2014;56:1–24. 10.18637/jss.v056.i13.

[57] Seabold S, Perktold J. Statsmodels: econometric and statistical modeling with Python. scipy. 2010. 10.25080/Majora-92bf1922-011.

[58] Ultralytics . YOLO Pre-trained Segmentation Models. https://docs.ultralytics.com/es/tasks/segment/#models.Accessed June 2025.

[59] Demir B, Sayıncı B, Çetin N, et al. Elliptic Fourier based analysis and multivariate approaches for size and shape distinctions of walnut (*Juglans regia* L.) cultivars. Grasas Aceites. 2018;69(4):e271. 10.3989/gya.0104181.

[60] Fernández i Martí A, Font i Forcada C, Socias i Company R. Genetic analysis for physical nut traits in almond. Tree Genet Genomes. 2013;9:455–65. 10.1007/s11295-012-0566-8.

[61] Font i Forcada C, Oraguzie N, Reyes-Chin-Wo S, et al. Identification of genetic loci associated with quality traits in almond via association mapping. PLoS One. 2015;10:e0127656. 10.1371/journal.pone.0127656. 26111146 PMC4482440

[62] Goonetilleke S N, Wirthensohn M G, Mather D E. Genetic analysis of quantitative variation in almond nut traits. Tree Genet Genomes. 2023;19:55. 10.1007/s11295-023-01630-w.

[63] Sánchez-Beeckman M, Fornés Comas J, Martorell O, et al. Three-dimensional image analysis for almond endocarp feature extraction and shape description. Comput Electron Agric. 2024;226:109420. 10.1016/j.compag.2024.109420.

[64] Sorkheh K, Shiran B, Khodambashi M, et al. Correlations between quantitative tree and fruit almond traits and their implications for breeding. Sci Hortic. 2010;125:323–31. 10.1016/j.scienta.2010.04.014.

[65] Feldmann M J, Hardigan M A, Famula R A, et al. Supporting data for "Multi-Dimensional Machine Learning Approaches for Fruit Shape Phenotyping in Strawberry.” GigaScience Database. 2020. 10.5524/100707. PMC7191992 32352533

[66] Feldmann M J . Classification and quantification of strawberry fruit shape. 2019. 10.5281/zenodo.3365714.Accessed June 2025.

[67] Keller B, Jung M, Bühlmann-Schütz S, et al. Dataset—the genetic basis of apple shape and size unraveled by digital phenotyping. ETH Zurich: Zurich. 2024. 10.1093/g3journal/jkae045 PMC11075547 38441135

[68] Utai K, Nagle M, Hämmerle S, et al. Mass estimation of mango fruits (Mangifera indica L., cv. ‘Nam Dokmai’) by linking image processing and artificial neural network. Eng Agricult Environ Food. 2019;12:103–10. 10.1016/j.eaef.2018.10.003.

[69] Chitwood D H, Kumar R, Headland L R, et al. A quantitative genetic basis for leaf morphology in a set of precisely defined tomato introgression lines. Plant Cell. 2013;25:2465–81. 10.1105/tpc.113.112391. 23872539 PMC3753377

[70] Migicovsky Z, Li M, Chitwood D H, et al. Morphometrics reveals complex and heritable apple leaf shapes. Front Plant Sci. 2018;8:2185. 10.3389/fpls.2017.02185. PMC5758599 29354142

[71] Wang H, Yin H, Li H, et al. Quantitative 2D fruit shape analysis of a wide range of pear genetic resources toward shape design breeding. Sci Hortic. 2024;327:112826. 10.1016/j.scienta.2023.112826.

[72] Zhao K, Wu X, Xiao Y, et al. PlanText: gradually masked guidance to align image phenotypes with trait descriptions for plant disease texts. Plant Phenomics. 2024;6:0272. 10.34133/plantphenomics.0272. 39600967 PMC11589250

[73] Mas-Gómez J, Rubio M, Dicenta F, et al. Dataset AlmondCV Pictures. 2025. 10.5281/zenodo.15423562.

[74] Mas-Gómez J, Rubio M, Dicenta F, et al. Supporting data for “Open RGB Imaging Workflow for Morphological and Morphometric Analysis of Fruits Using Deep Learning: A Case Study on Almonds.” GigaScience Database. 2025. 10.5524/102790. PMC12970599 41416705

