# Supplementary material for: Open RGB imaging workflow for morphological and morphometric analysis of fruits using deep learning: a case study on almonds
Source: Gigascience. 2025 Dec 19;15:giaf157. doi: 10.1093/gigascience/giaf157 (PMC12970599; doi:10.1093/gigascience/giaf157)
Supplement: giaf157_Supplemental_Files [file giaf157_supplemental_files.zip › Supplemmentary_Figures.docx]

**Open RGB Imaging Workflow for Morphological and Morphometric Analysis of Fruits using AI: A Case Study on Almonds.**

Mas-Gómez Jorge*^a^, Rubio Manuel^a^, Dicenta Federico^a^, Martínez-García Pedro José*^a^

^a^Fruit Breeding Group. Department of Plant Breeding, Centro de Edafología y Biología Aplicada del Segura- Spanish National Research Council (CEBAS-CSIC). Campus Universitario Espinardo, E-30100 Murcia, Spain

*Corresponding author: [jmgomez@cebas.csic.es](mailto:jmgomez@cebas.csic.es), [pjmgarcia@cebas.csic.es](mailto:pjmgarcia@cebas.csic.es)

**Supplementary Figures**


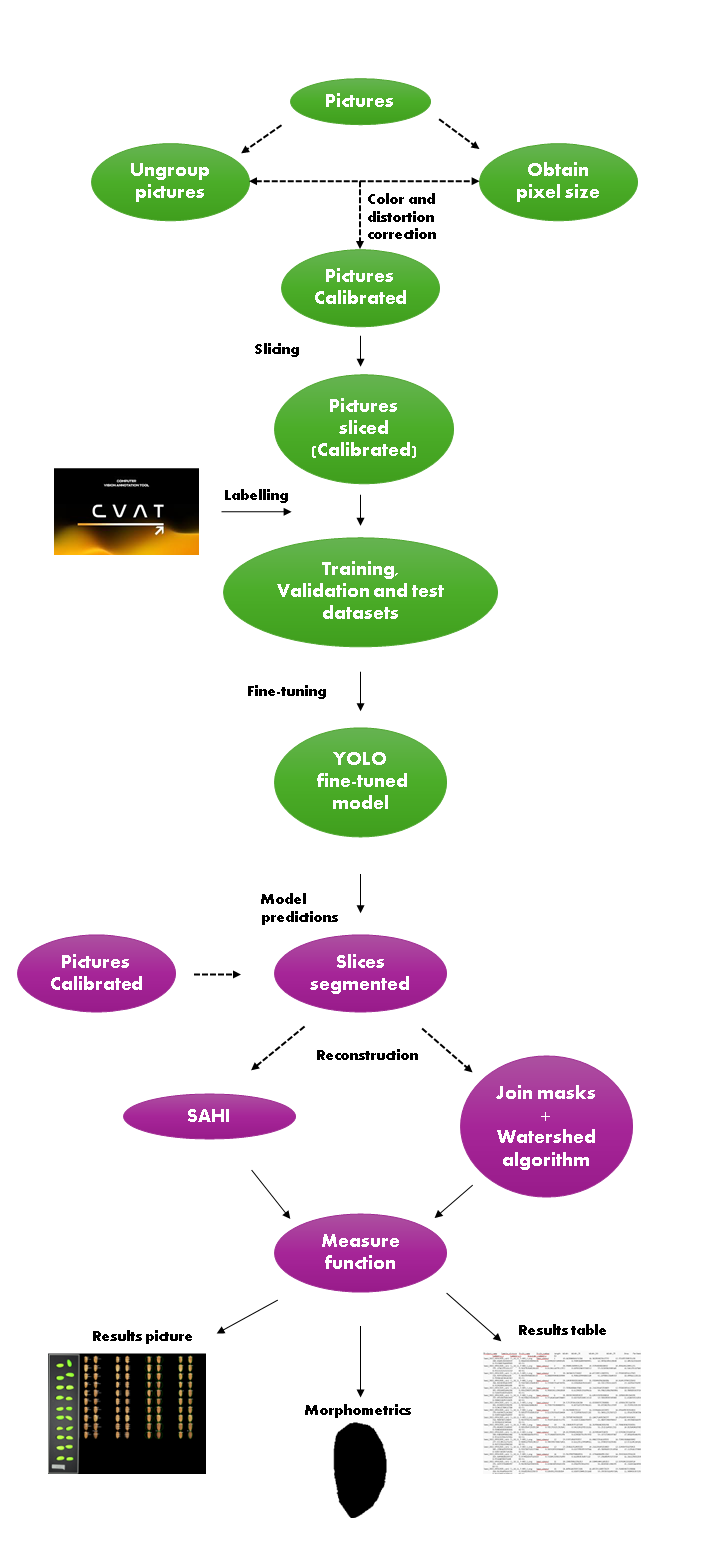


**Supplementary Figure 1.** Workflow description outlining the steps involved in developing the segmentation model (green) and deploying it (purple).

| A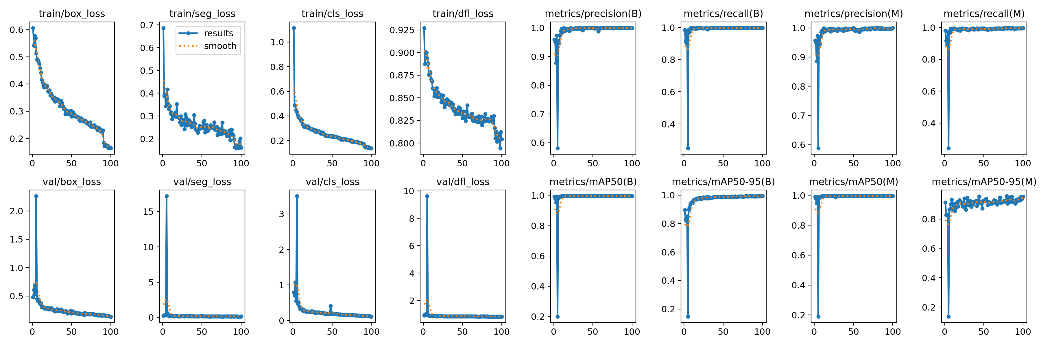 |
| --- |
| B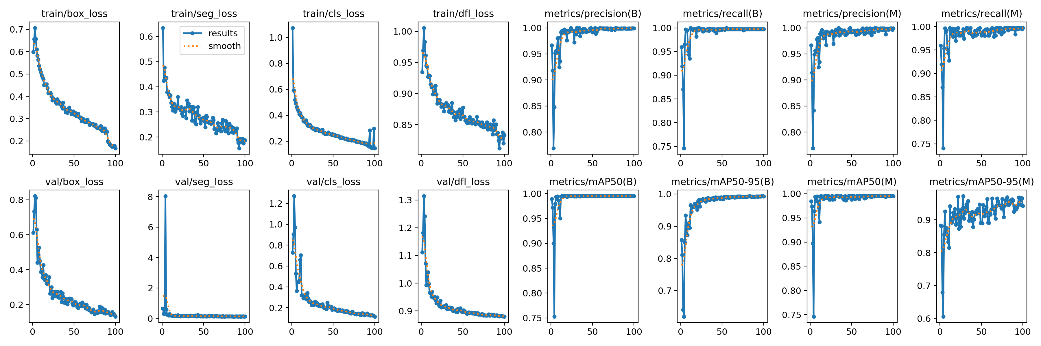 |
| C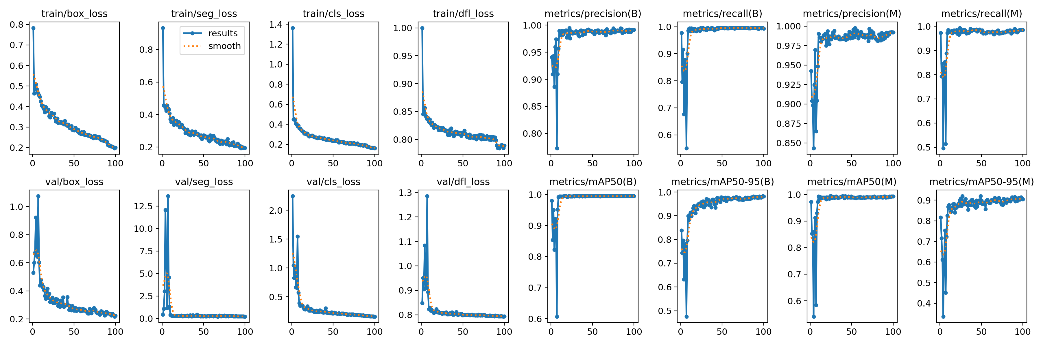 |
| D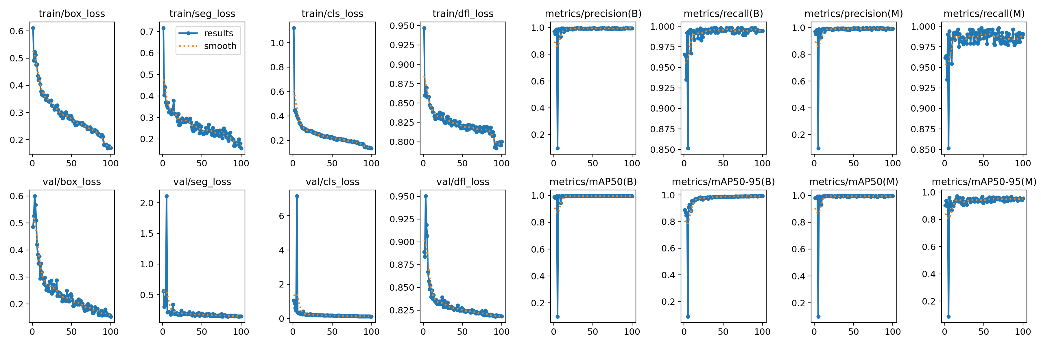 |

**Supplementary Figure 2**. YOLO metrics obtained during the fine-tuning process for the Kernel-2022 (A), Shell-2022 (B), Kernel-2023 (C), and Shell-2023 (D) datasets. A description of the metrics can be found in (Ultralytics, 2025).


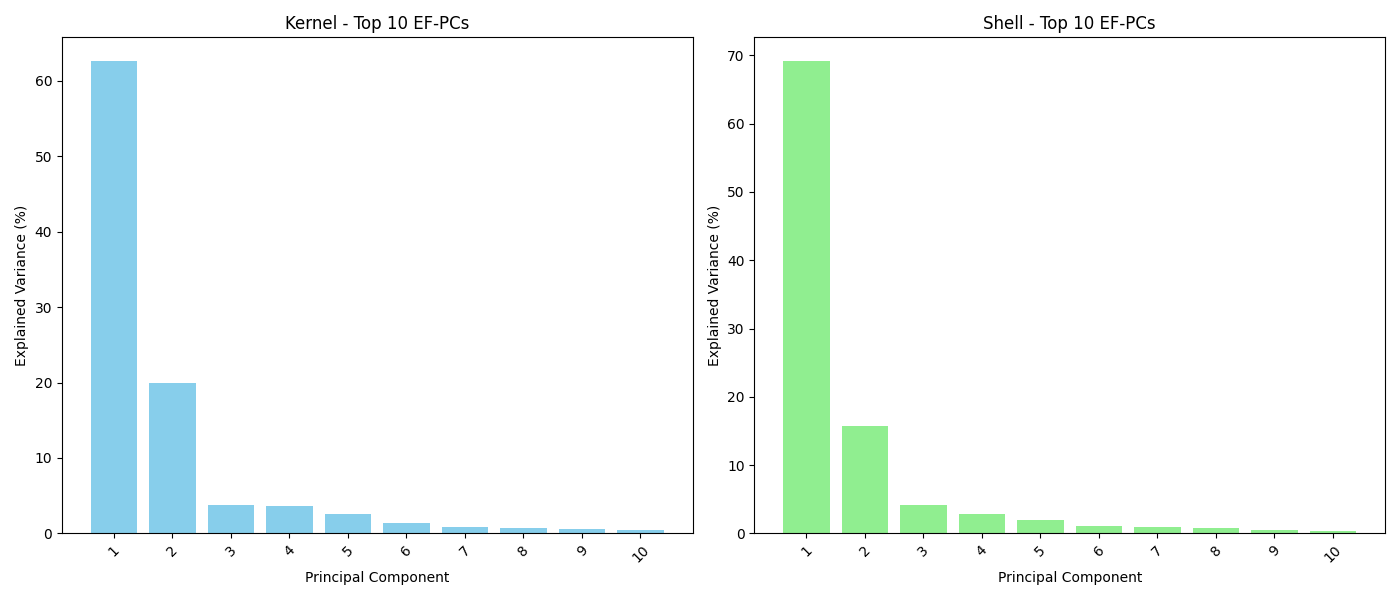


**Supplementary Figure 3.** Explained variance (%) per EF-PC in the kernel (left) and Shell (right) datasets.


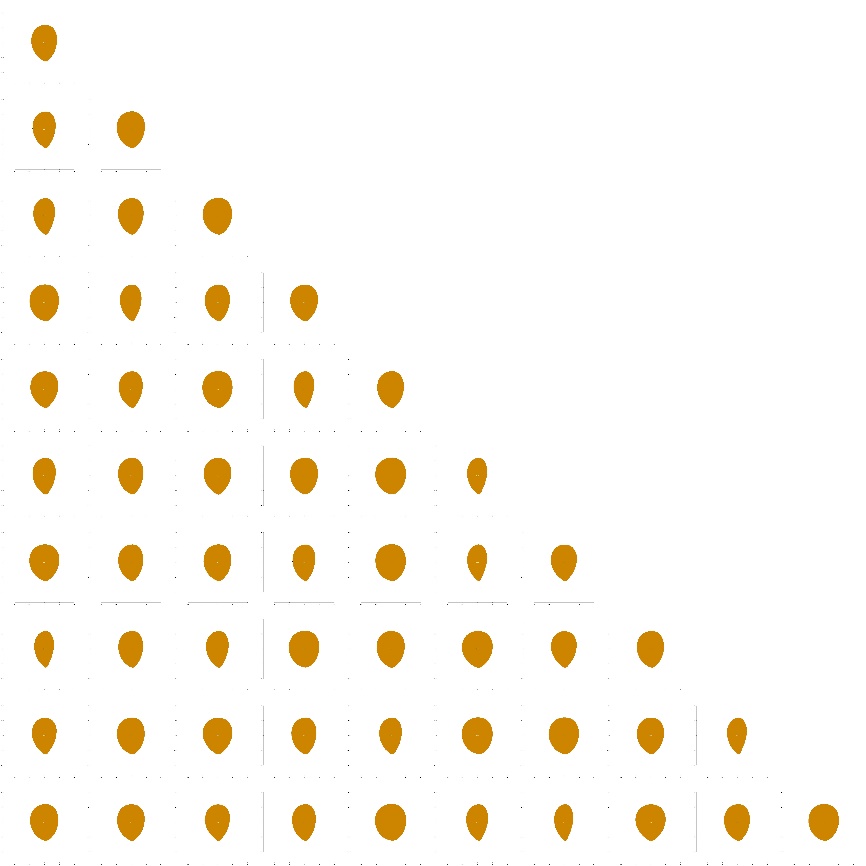


**Supplementary Figure 4.** K-means clustering using EFA-PCA results in shell dataset, showing the shapes corresponding to each centroid for scenarios ranging from k=1 to k = 10.

| 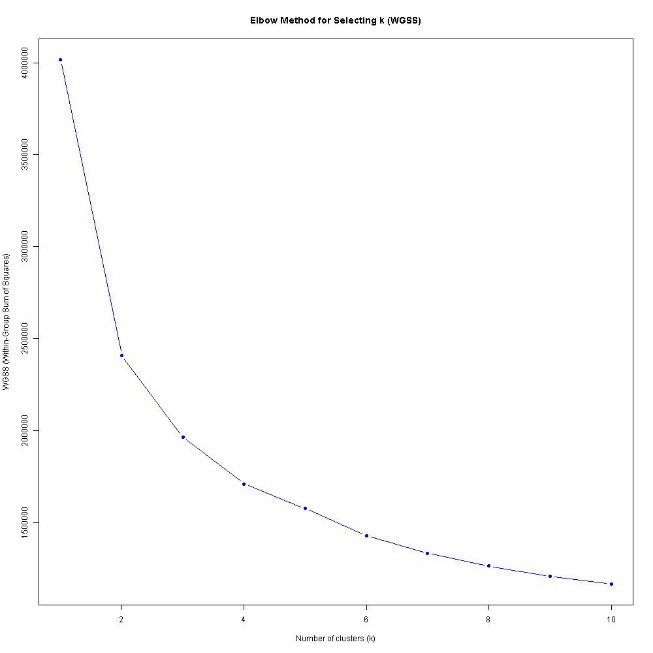 | 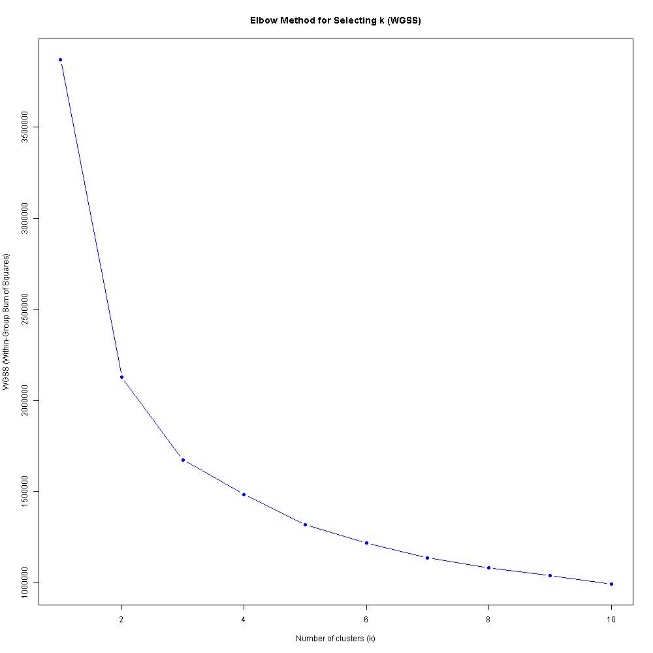 |
| --- | --- |

**Supplementary Figure 5.** Within group sum of squares decay for K-means clustering using EFA-PCA results in kernel (left) and shell (right) dataset.


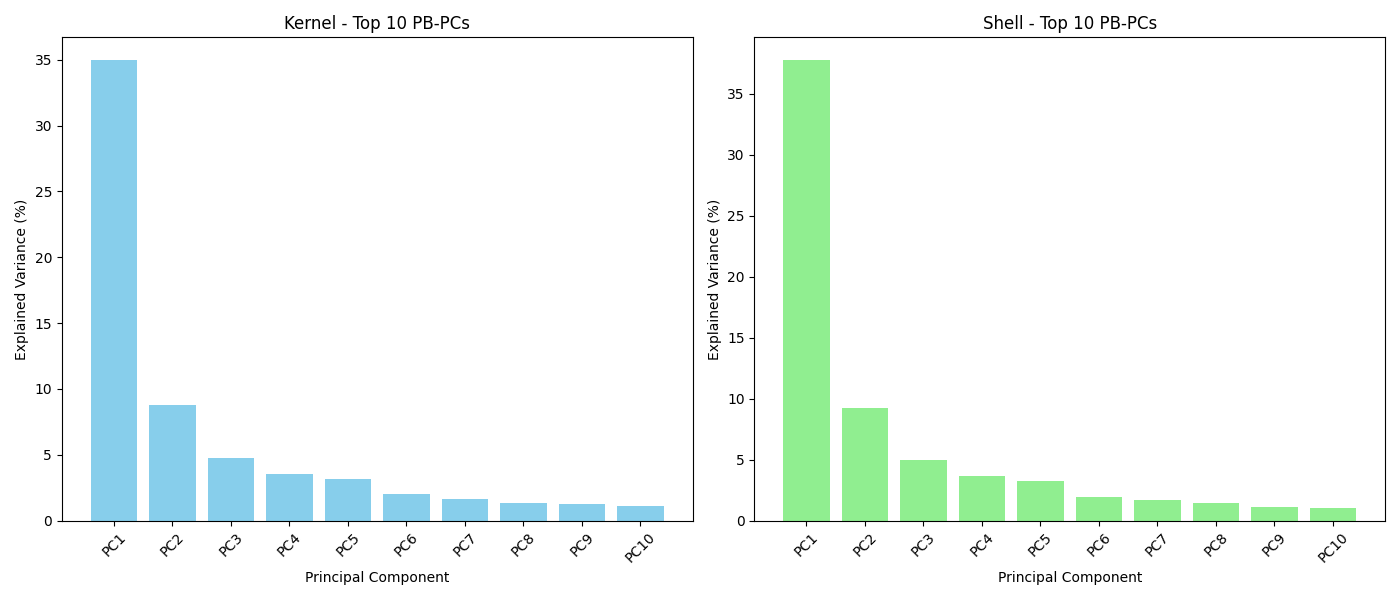


**Supplementary Figure 6.** Explained variance (%) per PB-PC in the kernel (left) and shell (right) datasets.

| 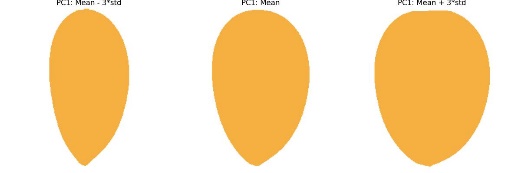 | 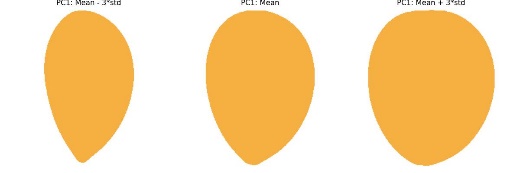 |
| --- | --- |
| 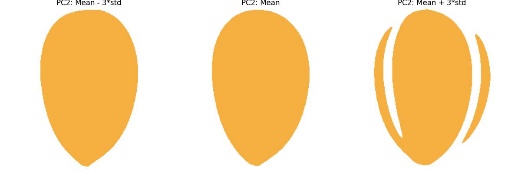 | 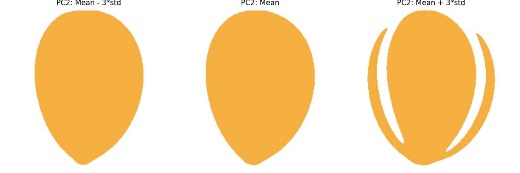 |
| 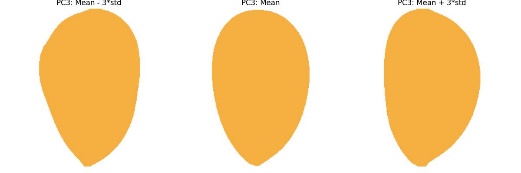 | 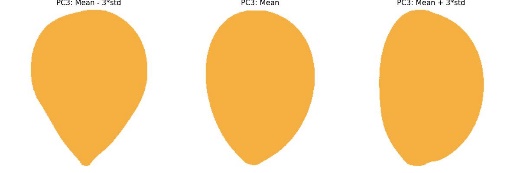 |
| 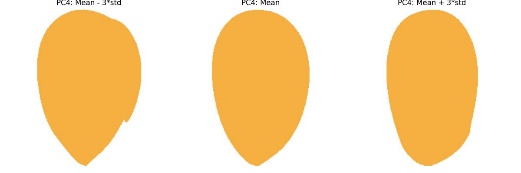 | 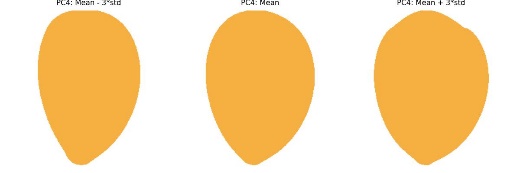 |
| 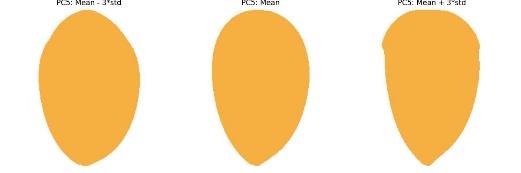 | 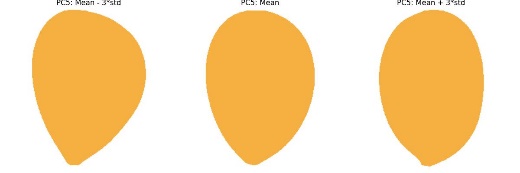 |
| 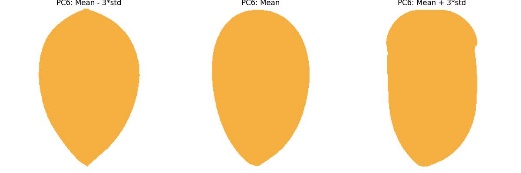 | 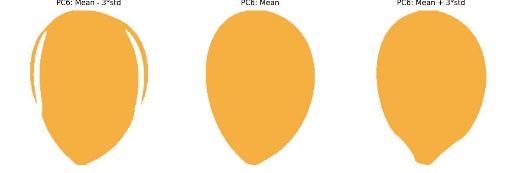 |
| 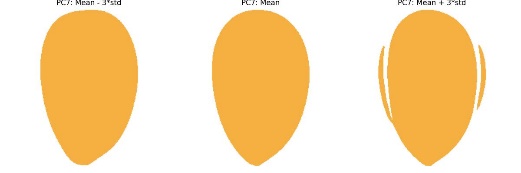 | 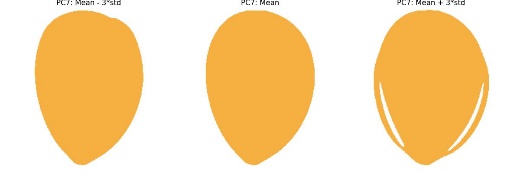 |
| 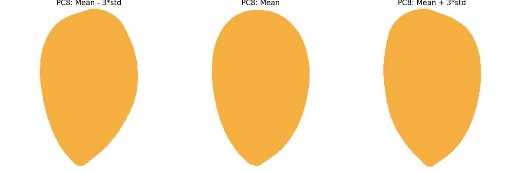 | 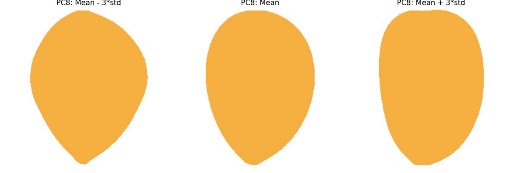 |
| 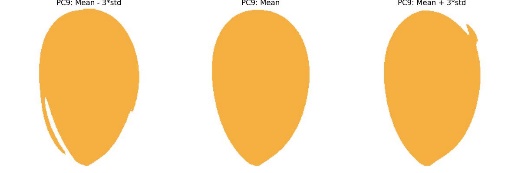 | 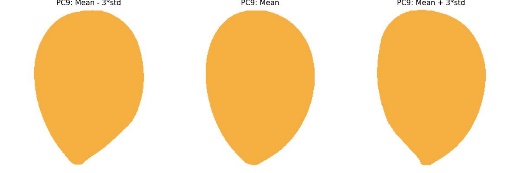 |
| 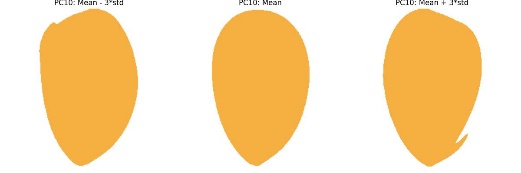 | 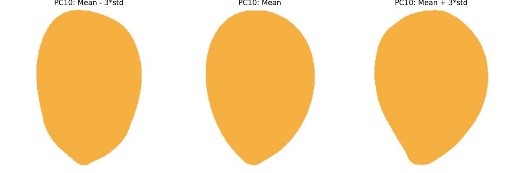 |

**Supplementary Figure 7.** Representation of the influence of the PB-PCs on shape in the kernel (left) and shell (right) datasets, from the mean shape to ±3× standard deviation.


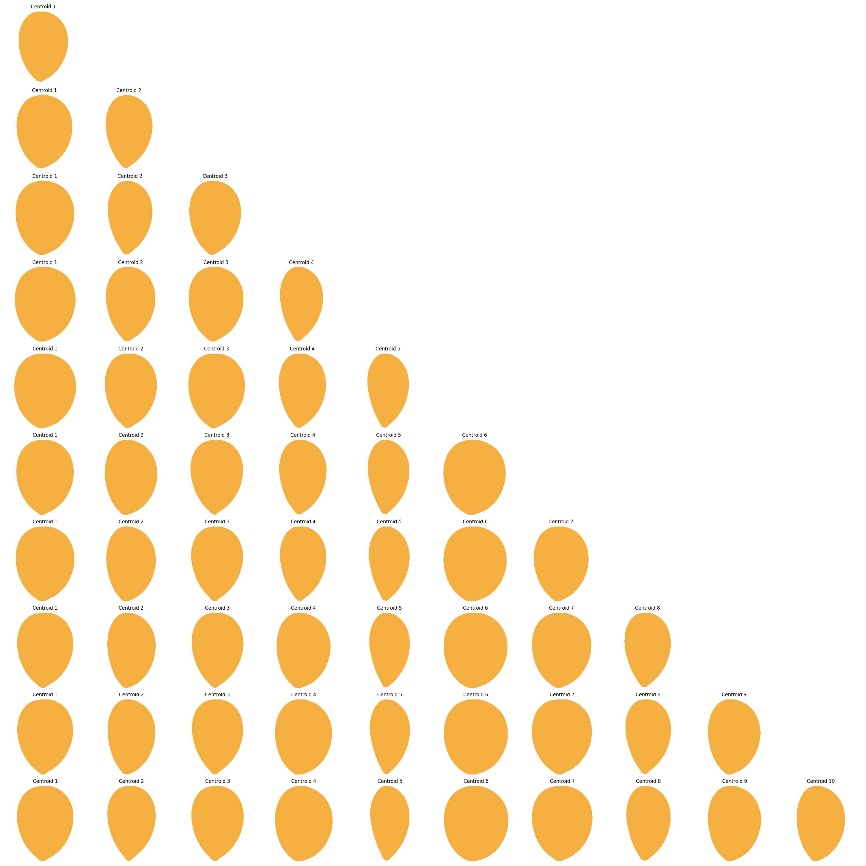


**Supplementary Figure 8.** K-means clustering using PB-PCA results in shell dataset, showing the shapes corresponding to each centroid for scenarios ranging from k=1 to k = 10.

| 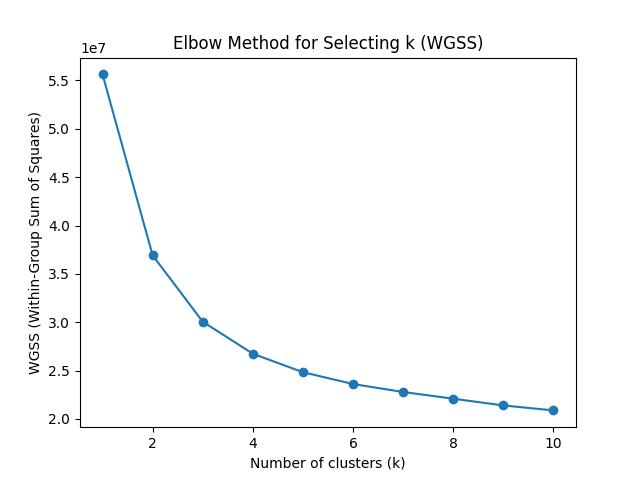 | 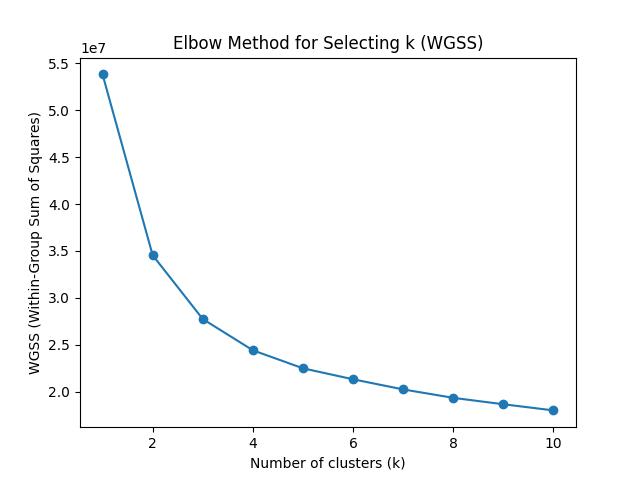 |
| --- | --- |

**Supplementary Figure 9.** Within group sum of squares decay for K-means clustering using PB-PCA results in kernel (left) and shell (right) dataset.
