# Supplementary material for: Open RGB imaging workflow for morphological and morphometric analysis of fruits using deep learning: a case study on almonds
Source: Gigascience. 2025 Dec 19;15:giaf157. doi: 10.1093/gigascience/giaf157 (PMC12970599; doi:10.1093/gigascience/giaf157)
Supplement: giaf157_GIGA-D-25-00218_Original_Submission [file giaf157_giga-d-25-00218_original_submission.pdf]

GigaScience

Open RGB Imaging Workflow for Morphological and Morphometric Analysis of Fruits using AI: A Case Study on Almonds.

--Manuscript Draft--

**Manuscript Number:**	GIGA-D-25-00218
**Full Title:**	Open RGB Imaging Workflow for Morphological and Morphometric Analysis of Fruits using AI: A Case Study on Almonds.
**Article Type:**	Research
**Funding Information:**	Ministerio de Ciencia e Innovación (PID2021-127421OB-I00) — PhD Dicenta Federico<br>Ministerio de Ciencia e Innovación (CNS2022-135936) — PhD Martínez-García José Pedro<br>Ministerio de Universidades (FPU20/00614) — Sr Jorge Mas Gómez

Abstract:

Background
High-throughput phenotyping is addressing the current bottleneck in phenotyping within breeding programs. Imaging tools are becoming the primary resource for improving the efficiency of phenotyping processes and providing large datasets for genomic selection approaches. The advent of AI brings new advantages by enhancing phenotyping methods using imaging, making them more accessible to breeding programs. In this context, we have developed an open Python workflow for analyzing morphology, colour and morphometric traits using AI, which can be applied to fruits and other plant organs.

Results
The workflow was implemented in almond (Prunus dulcis (Mill.) D. A. Webb), a species where breeding efficiency is critical due to its long breeding cycle. Over 25,000 kernels, more than 20,000 nuts, and over 600 individuals were phenotyped, making this the largest morphological study conducted in almond so far. The best segmentation and reconstruction approaches achieved error rates below 1%. Weight and area variables enabled accurate estimation of kernel thickness, with a root mean squared error (RMSE) of 0.47. Fifty-five heritable morphological, morphometric and colour traits were identified, highlighting their potential as target traits in breeding programs.

Conclusion
The proposed workflow demonstrated robust performance across diverse datasets and being effective with limited training data for fine-tuning. Its compatibility with the output of AI-based labelling tools allows users to fully leverage the advantages of these technologies—reducing manual effort, accelerating dataset preparation, and streamlining the fine-tuning process of segmentation models. This flexibility enhances the scalability and practical applicability of the workflow in real-world phenotyping scenarios, especially in the context of breeding programs.

**Corresponding Author:**	Martínez-García José Pedro<br>Centro de Edafologia y Biologia Aplicada del Segura<br>Murcia, SPAIN
**Corresponding Author Secondary Information:**	
**Corresponding Author's Institution:**	Centro de Edafologia y Biologia Aplicada del Segura
**Corresponding Author's Secondary Institution:**	
**First Author:**	Jorge Mas Gómez
**First Author Secondary Information:**	
**Order of Authors:**	Jorge Mas Gómez
	Rubio Manuel
	Dicenta Federico

Powered by Editorial Manager® and ProduXion Manager® from Aries Systems Corporation

	Martínez-García José Pedro
**Order of Authors Secondary Information:**	
**Additional Information:**	
**Question**	**Response**
Are you submitting this manuscript to a special series or article collection?	No
**Experimental design and statistics**<br><br>Full details of the experimental design and statistical methods used should be given in the Methods section, as detailed in our Minimum Standards Reporting Checklist. Information essential to interpreting the data presented should be made available in the figure legends.<br><br>Have you included all the information requested in your manuscript?	Yes
**Resources**<br><br>A description of all resources used, including antibodies, cell lines, animals and software tools, with enough information to allow them to be uniquely identified, should be included in the Methods section. Authors are strongly encouraged to cite Research Resource Identifiers (RRIDs) for antibodies, model organisms and tools, where possible.<br><br>Have you included the information requested as detailed in our Minimum Standards Reporting Checklist?	Yes
**Availability of data and materials**<br><br>All datasets and code on which the conclusions of the paper rely must be either included in your submission or deposited in publicly available repositories (where available and ethically appropriate), referencing such data using a unique identifier in the references and in	Yes

the “Availability of Data and Materials” section of your manuscript.<br><br>Have you have met the above requirement as detailed in our Minimum Standards Reporting Checklist?	
GigaScience has policies and guidelines in place for the use of generative AI-writing tools such as ChatGPT. If you have used such writing tools to assist with writing the manuscript this must be declared and cited in the text. Authors should not list AI-writing tools and other AI-assisted technologies as an author or co-author and should acknowledge that they are fully responsible for text generated or refined by AI-writing tools.<p><br><br>A summary of use (particularly in the introduction or among methods) needs to be included at the end of the paper, and the outputs should also be included as a supplementary file hosted in GigaDB or other open repositories. Please read our guidelines for more information. <p><br><br>By submitting to GigaScience, you are aware of the journal's AI-writing tools policy, and if you have declared use of such tools below, you have acknowledged this where appropriate in your manuscript and have made a summary of use and outputs available. <p><br><b>AI-assisted writing tools have been used in the preparation of this manuscript?	No

Powered by Editorial Manager® and ProduXion Manager® from Aries Systems Corporation

Manuscript

Click here to access/download;Manuscript;renamed_b05b4.docx

Open RGB Imaging Workflow for Morphological and Morphometric Analysis of Fruits using AI: A Case Study on Almonds.

Mas-Gómez Jorge, Rubio Manuel, Dicenta Federico, Martínez-García Pedro José*

Fruit Breeding Group. Department of Plant Breeding, Centro de Edafología y Biología Aplicada del Segura- Spanish National Research Council (CEBAS-CSIC). Campus Universitario Espinardo, E-30100 Murcia, Spain

*Corresponding author: pjmgarcia@cebas.csic.es

Abstract

Background

High-throughput phenotyping is addressing the current bottleneck in phenotyping within breeding programs. Imaging tools are becoming the primary resource for improving the efficiency of phenotyping processes and providing large datasets for genomic selection approaches. The advent of AI brings new advantages by enhancing phenotyping methods using imaging, making them more accessible to breeding programs. In this context, we have developed an open Python workflow for analyzing morphology, colour and morphometric traits using AI, which can be applied to fruits and other plant organs.

Results

The workflow was implemented in almond *(Prunus dulcis* (Mill.) D. A. Webb), a species where breeding efficiency is critical due to its long breeding cycle. Over 25,000 kernels, more than 20,000 nuts, and over 600 individuals were phenotyped, making this the largest morphological study conducted in almond so far. The best segmentation and reconstruction approaches achieved error rates below 1%. Weight and area variables enabled accurate estimation of kernel thickness, with a root mean squared error (RMSE) of 0.47. Fifty-five heritable morphological, morphometric and colour traits were identified, highlighting their potential as target traits in breeding programs.

Conclusion

The proposed workflow demonstrated robust performance across diverse datasets and being effective with limited training data for fine-tuning. Its compatibility with the output of AI-based labelling tools allows users to fully leverage the advantages of these technologies—reducing manual effort, accelerating dataset preparation, and streamlining

the fine-tuning process of segmentation models. This flexibility enhances the scalability and practical applicability of the workflow in real-world phenotyping scenarios, especially in the context of breeding programs.

Keywords

High-throughput Phenotyping, Almonds, Breeding, Computer Vision, Deep learning

Background

High-throughput phenotyping is becoming a valuable tool for plant breeders and researchers to meet the challenges of future food demand [1,2]. Currently, breeding programs operate in a scenario where genotype data has expanded exponentially due to the accessibility of next-generation sequencing (NGS) technologies [3,4]. Consequently, the implementation of genomic selection approaches and candidate gene discovery has advanced significantly [3,5]. However, these approaches also depend on phenotyping, which remains the primary bottleneck in breeding programs [5]. To overcome this bottleneck, classical phenotyping tasks, which are often tedious, time-consuming, subjective and/or expensive, are being replaced. Breeders are leveraging tools such as computer vision [6], artificial intelligence [7], and unmanned aerial platforms [8], among others, to meet the need for large-scale phenotyping and the generation of accurate, accessible big datasets [5].

The use of high-throughput phenotyping platforms for fruit species such as apple, mango, vineyard or citrus have helped to study architecture parameters, pigment and nutrient contents, water stress, biochemical parameters of fruits and disease detection [9]. A device for fast and accurate phenotyping of small fruit samples called “FruitPhenoBox“ was developed for breeding programs [10], leading to the identification of key marker-trait associations in apple populations [11]. Additionally, by integrating genotype data, RGB imaging, and autoencoders, a framework was developed to reconstruct apple samples using only genotype data [12]. Morphometric approaches using machine learning have been applied in strawberries to extract quantitative shape features, enabling the identification of new heritable traits [13]. Furthermore, an automatic pipeline to

phenotype morphological traits was developed for strawberries using computer vision techniques [14] and 3D imaging [15], with applicability to other fruits.

For stone fruit trees species, such as almond (*Prunus dulcis* (Mill.) D. A. Webb) the implementation of these tools is still scarce. Breeding almond, and other *Prunus* species such as apricot, peach, plum or cherry is a long and expensive task. Due to their long juvenility period the timing to obtain a new improved cultivar is around 12-15 years. Therefore, increasing efficiency is a key aspect for breeding these species, particularly in phenotyping processes of traits of interest such as fruit morphology, shape and colour [16–18]. These traits directly influence market value and consumer acceptance [19]. Preliminary efforts in imaging analysis and deep learning in almond have demonstrated that the same quantitative trait loci (QTLs) for size and shape can be successfully identified using these approaches as with traditional manual phenotyping methods [20]. Moreover, 3D imaging phenotyping methods have been developed for almonds nuts [21].

In most of the mentioned studies, segmentation — defined as the process of partitioning a digital image into meaningful regions of interest (ROIs) based on homogeneous visual features such as colour, texture, or intensity — relies on computer vision algorithms, which often require manual adjustments, limiting automation—especially as scene complexity increases [22,23]. To enhance efficiency and automation, advanced tools capable of handling this complexity are essential for fruit morphology studies [24]. In this sense, deep learning techniques have gained significant relevance due to their high segmentation accuracy, even in complex scenarios, by leveraging large training and validation datasets [25]. Instance segmentation using deep learning can be implemented through two main approaches: two-stage models (e.g., Mask R-CNN, Faster R-CNN) and one-stage models (e.g., *You look only once* (YOLO), RetinaNet) [26]. While two-stage models offer higher accuracy at the cost of lower speed and greater computational

requirements, one-stage models aim to balance accuracy and speed while using fewer resources [26]. Notably, recent advancements in YOLO models have significantly improved their accuracy while maintaining high-speed performance [23]. Nevertheless, training deep learning models from scratch requires large labelled datasets and substantial computational resources [27]. In this context, fine-tuning pre-trained generalist models allows users to develop customized models without the need for extensive datasets [27,28]. The integration of deep learning tools for labelling datasets has significantly accelerated this task [28,29]. Moreover, new techniques such as Slicing Aided Hyper Inference (SAHI) have enhanced accuracy by reducing errors in the segmentation of small objects, making it particularly valuable for seed imagery [30]. The slicing process also reduces memory requirements for processing large images, maintaining high resolution without the need to resize them for model training, and enabling the utilization of limited GPU (Graphics Processing Unit) open resources (e.g., Google Colab).

In this work, we have developed a Python-based workflow to facilitate the development and deployment of custom deep learning models for measuring morphological and morphometric traits in fruit breeding programs. The workflow includes pre-processing methods such as colour and distortion correction, image segmentation, and pixel size estimation and it was successfully applied to phenotype multiple almond breeding populations, allowing for the estimation of broad-sense heritability values. Additionally, prediction models were developed to estimate kernel thickness based on area and weight. By implementing this workflow, the study has analyzed the largest number of individuals and data points ever recorded for the almond species to date.

Material and Methods

Plant material

The workflow was implemented to study breeding populations of the CEBAS-CSIC almond breeding program located in the experimental field (Santomera, Murcia SE of Spain) during 2022 and 2023 seasons. 665 unique genotypes from six F1 populations and a germplasm collection were studied (Table 1). Population parents were traditional cultivars (‘Marcona’ and ‘Desmayo Largueta’), released cultivars of CEBAS-CSIC (‘Antoñeta’, ‘Penta’, ‘Tardona’, and ‘Florida’) and the breeding selections ‘R1000’ (INRA, France) and ‘S4017’ (CEBAS-CSIC). The target samples were shells and kernels of these almond descendants. For each genotype, 30 in-shell nuts and 30 kernels per year were sampled, photographed, and weighed, whenever available.

Table 1. The populations used in this study and the number of individuals studied per year

**Year**	**2022**		**2023**		**Unique genotypes**	
**Family**	**Shell**	**Kernel**	**Shell**	**Kernel**	**Shell**	**Kernel**
Germplasm collection	85	85	74	91	99	99
Antoñeta × Marcona	6	6	19	19	19	19
Antoñeta × Penta	161	161	142	142	183	183
Antoñeta × Tardona	57	57	56	53	71	70
Florida × Marcona	17	17	43	43	44	44
Desmayo × R1000	198	198	187	194	223	223
Marcona × S4017	0	0	26	27	26	27

Total	524	524	547	569	665	665

Workflow description

A comprehensive workflow has been developed for pre-processing, segmentation model development, deployment, morphological measurements, and morphometric analyses, implemented through interactive Python notebooks (Supplementary Figure 1) (https://github.com/jorgemasgomez/almondcv2). To optimize speed and performance, the use of a GPU is highly recommended. The workflow can be executed locally; however, it is also deployed on the Google Colab platform, enabling users to run it online and leverage free resources, such as GPUs. Although primarily written in Python, some functions integrate R for morphometric analyses, requiring R to be properly configured in the system's path if executed locally.

Colour and distortion calibration

Colour and distortion correction approaches are included in the pre-processing notebook (*1_Pre-processing_workflow.ipynb*). Colour correction enables to standardize the dataset of the images using a colour card as reference. Colour correction functions implemented in the pre-processing notebook are based on the colour correction module of PlantCV python library [31]. Distortion correction is implemented for possible radial and tangential distortion caused by the camera. Distortion correction functions are based on the OpenCV workflow for camera calibration [32]. It requires test patterns of chessboards with a known size of the squares to obtain a camera matrix that can be used subsequently with the pictures of the dataset. In addition, colour and distortion correction functions are joined in the pre-processing notebook to deploy it in the whole dataset in one step.

Auxiliary functions

Some optional auxiliary functions were included for the picture pre-processing, helping to separate the different samples in the pictures and getting the physical size of a pixel with reference objects in the picture (e.g. a coin with known diameter). These optional functions require training a model (next sections) to identify and segment the sample groups and the reference objects.

Develop your segmentation model

The approach implemented in the present work intends to simplify the development of customized segmentation models. Four key steps are necessary for performing the development: slicing, labelling, 'training' and reconstruction (*2_Develop_your_segmentation_model_workflow.ipynb*). The slicing process consists of cropping pictures into smaller patches, with the size determined by the user. Using smaller patches of high-resolution images speeds up the training and prediction processes by reducing computational requirements [30]. Additionally, this approach improves the capture of fine details, as it avoids the loss of resolution and distortion caused by resizing the entire image [33,34]. The slicing function defined in the workflow is divided into train, validation, and test datasets. The slices are obtained proportionally according to the user command to prepare for the next step. The second step, labelling, involves annotating which pixels correspond to the object of interest. For this task, the workflow was designed to accept as input the ZIP file generated by the image annotation platform CVAT in the YOLO Segmentation 1.0 format [29]. CVAT provides tools for semi-automatic segmentation using the Segment Anything Model (SAM) [28], which enables quick instance segmentation with a single click. CVAT is available for free offline via a Docker container or online with a freemium model. The third step for developing the segmentation model is 'training' the model. For that purpose, in the second notebook (*2_Develop_your_segmentation_model_workflow.ipynb*) YOLO [35] pre-trained

algorithm series can be fine-tuned (although in their web mention 'training' really is a fine-tuning [36]) using our custom labelled dataset. The training process function enables to control all the parameters of the training process (e.g. epochs, batch) and provides the results of the training *YOLO.train* method. The last step for developing the segmentation model is to reconstruct the binary picture mask, for which two approaches were used. The first approach simply joins the patches binary masks after the prediction (*slice_predict_reconstruct*). The contours are detected using *cv2.findcontours* OpenCV function [37] in the subsequent functions for morphology measurements. Watershed algorithm is implemented in such functions for separating touching objects of interest [38]. The second approach uses the SAHI pipeline (*predict_model_sahi*) [30], which is integrated in the YOLO segmentation process, and the instances segmented are identified specifically providing their contours directly. Here, SAHI python library is modified slightly to enable the *retina_mask* argument in YOLO prediction function and provide fine details in the segmentations. To assess the two reconstruction approaches, they were tested in the datasets studied and the almonds with errors in the reconstruction were annotated and removed manually.

<u>Deploy your segmentation model for morphology and morphometric analyses</u>

Once the segmentation model has been successfully 'trained' and tested, it can be deployed for morphology and morphometric analyses. In the notebook for deployment (*3_Deploy_your_segmentation_model_worflow.ipynb*), two methods were prepared to measure.

The first method is general for any group of fruit, and the traits length (the longest axis of the fruit), width (the dimension perpendicular to the length), area (the surface enclosed by the fruit contour), perimeter (the length of the outer boundary), hull-area (the area of the convex hull enclosing the shape), solidity (the ratio between area and hull-area,

reflecting compactness), aspect ratio (the ratio of length to width, indicating elongation), circularity (calculated as 4π×Area/Perimeter, with values closer to 1 indicating a more circular shape), ellipse ratio (the ratio between the minor and major axes of a fitted ellipse), and colour (L*a*b* model) are measured for each shell/kernel. This general method can be used as a template and customized according to the user's needs, for example to study additional traits or different fruit shapes. The second method is specific for almonds adding more traits such as width at three different heights (25, 50 and 75% of the length), vertical and horizontal symmetry and symmetry in the top part of the almond (to measure the shoulder). In this specific method, almonds are aligned according to the angle of fitting an ellipse (*cv2.fitEllipse* function) and flipped vertically with placing the most distant point always in the lowest part (almond tip). Some extra traits derived from the results together with the weight of the kernel/shell such as the weight kernel/shell ratio, estimation of the thickness (only for kernels) and globosity (width/thickness), were also included. For the estimation of kernel thickness, a linear and quadratic model was fitted using 228 almond kernels, using the area and the weight as predictor variables. To validate the models, the dataset was split into 80% for training 20% for validating, running it 100 times to assess the stability of the models.

Both methods export picture results, table results, and binary masks for morphometric analyses. Binary masks are flipped horizontally if the lowest pixel (tip) is the right part of the picture to avoid symmetric issues in morphometric analysis.

Morphometric analyses notebook (*4_Morphometrics_workflow.ipynb*) includes two morphometric approaches: Elliptical Fourier analysis (EFA) and pixel-based principal component analysis (PCA). EFA is conducted using Momocs v1.4.1. R package [39] but it has been implemented in the notebook in Python programming language executing a subprocess. Several functions are included for EFA: exploratory analysis, running the

EFA, after that perform a PCA on the EFA results and *kmeans* clustering. PCA performed on EFA coefficients generates as traits the components (EF-PCs) that explain the variability of the shape. Regarding the second pixel pixel-based PCA, binary masks are flattened for performing the PCA and generating also PCs as traits (PB-PCs)[13]. Result plots of both approaches can be obtained to show the influence of each trait.

RGB Imaging system

For image acquisition, group pictures of various samples were captured over a black surface template, with the samples positioned inside painted rectangles. For the 2022 dataset, images were taken using a Canon EOS 70D camera, achieving a resolution of 6 px/mm. In 2023, the imaging system was redefined due to objectives beyond the scope of this work, utilising an Arducam 8MP IMX219 camera, which resulted in images with a resolution of 4 px/mm. Both systems were illuminated using two white LED light sources.

Heritability

Broad sense heritability (H^2) was estimated using the Python library *statsmodels* [40], fitting a linear mixed model using the genotype as random effects and the year as fixed effects (Equations 1 and 2).

$$Y_{ijk} = \mu + G_i + Y_j + \varepsilon_{ijk} \quad (\textit{Equation 1})$$

Where:

Y_{ijk}: Observed phenotypic value for the k-th observation of the i-th genotype in the j-th year.

μ: Overall population mean.

G_i: Random effect of the i-th genotype

Y_j: Fixed effect of the j-th year

ε_{ijk}: Residual error term

$$\frac{\sigma^2 G}{\sigma^2 G + \sigma^2 e} (Equation\ 2)$$

Where:

σ^2G Genetic variance.

σ^2e: Residual variance.

Impact on almond breeding

To study the impact of this new tool on almond breeding, a comprehensive comparison with existing scientific articles related to quantitative almond morphology phenotyping was conducted. Several parameters such as the number of individuals studied, number of shells/kernels phenotyped, and the number of data points obtained, were compared to evaluate the benefits of the new high-throughput phenotyping tool.

Results

YOLO fine-tuned segmentation models performance and reconstruction errors.

A total of 46,737 elements (shell and kernels) were segmented, and performance errors from both reconstruction approaches were annotated (Table 2). A standard image generated by the workflow is shown in Figure 1. Four segmentation models were fine-

tuned using datasets from kernel and from shell in 2022 and 2023. The pre-trained model 'yolov11s-seg.pt' was selected, and fine-tuning was performed over 100 epochs (i.e., complete passes through the entire training dataset), using 500–1,000 image slices of 320 pixels each, split into 60% for training, 20% for validation, and 20% for testing. Metrics plots from the fine-tuning process of the datasets are shown as an example in Supplementary Figure 2. Validation masks were explored, showing precise results.

All the combinations showed errors lower than 1% except for the shell 2022 dataset using SAHI (2.60%). Performance was similar between both approaches except for a 2% difference in the shell 2022 dataset.

Figure 1. Images generated by the workflow, displayed from left to right: original image with masks, length and width measurements, widths at different lengths, circularity, and ellipse ratio.

Table 2. Performance for different datasets and reconstruction methods, including reconstruction errors.

Method	Dataset	Errors	Total elements	Error percentage
Slice_predict_reconstruct + Watershed	Kernel-2022	6	10,180	0.06%
	Kernel-2023	135	15,312	0.88%
	Shell-2022	4	10,360	0.04%
	Shell-2023	47	10,885	0.43%
SAHI	Kernel-2022	13	10,180	0.13%
	Kernel-2023	124	15,312	0.81%
	Shell-2022	269	10,360	2.60%
	Shell-2023	43	10,885	0.40%

Thickness estimation

Kernel thickness was modelled using weight and area as predictor variables, employing both linear and quadratic models. The median R^2 in the test dataset for the linear model was 0.725, while for the quadratic model it was 0.795. The corresponding median Root Mean Squared Error (RMSE) values were 0.56 for the linear model and 0.47 for the quadratic model (Figure 2A). The best-performing linear and quadratic models were then plotted, achieving R^2 values of 0.847 and 0.8815, respectively, and RMSE values of 0.451 and 0.381, respectively (Figure 2B). The quadratic model trained with the complete dataset was employed for estimating thickness across all samples in subsequent analysis.

Figure 2. Results of the kernel thickness modelling, showing the R^2 (A, left) and RMSE (A, right) over 100 iterations, along with scatter plots for the best linear (B, left) and quadratic (B, right) models.

Morphometric results

Elliptical Fourier analysis (EFA) was conducted using all the kernel and shell binary masks (separately), placing 10 harmonics in both datasets after a visual exploration analysis. PCA was performed on the EFA coefficients, being 62.63%, 19.93%, and 3.83% explained by the three first PCs for the kernel analysis, and 69.19%, 15.75%, and 4.15% explained by the three first PCs for the shell analysis (Figure 3 and Supplementary Figure 3). Only the first 6 components on each dataset were used for subsequent analysis because they explained at least 1% of the variability. K-means clustering from k=1 to k=10 was run to show the main groups of the datasets (Figure 4 and Supplementary Figure 4. The optimal number of clusters was studied and no abrupt changes in the slope by elbow method were observed in any dataset (Supplementary Figure 5).

Figure 3. EFA-PCA scatterplot results for the kernel A) EF-PC1/EF-PC2 and B) EF-PC1/EF-PC3 and shell C) EF-PC1/EF-PC2 and D) EF-PC1/EF-PC3 datasets. The influence of the PCs on shape is represented in the plots.

Figure 4. K-means clustering results using EFA-PCA in kernel dataset, showing the shapes corresponding to each centroid for scenarios ranging from k=1 to k = 10.

Pixel-based PCA was performed in kernel and shell datasets, obtaining 34.96%, 8.78% and 4.75% variance explained by the three first PCs in the kernel dataset and 37.72%, 9.19% and 4.96% in the shell dataset (Supplementary Figure 6). The PCs influence in the shape was studied by plotting the +-3x deviation from the mean shape (Figure 5 and Supplementary Figure 7). Ten PB-PCs were collected for each dataset, explaining at least 1% of the variance for each one of them. K-means clustering was also carried out from k=1 to k=10 (Figure 5 and Supplementary Figure 8), and no abrupt changes were observed by the elbow method to determine the optimal number of clusters (Supplementary Figure 9).

Figure 5. Example images for Shell PB-PC3 (A) and Kernel PB-PC5 (B) morphometric traits, showing two contrasting phenotypes for each trait. Panels A1 and B1 show the original almond images; A2 and B2 show the corresponding binary masks; and A3 and B3 display the pixel-based PCA traits, from the mean shape to ±3 standard deviations.

Figure 6. K-means clustering results using PB-PCA in kernel dataset, showing the shapes corresponding to each centroid for scenarios ranging from k=1 to k = 10.

Correlations

To analyse and interpret the phenotype data obtained, the Pearson correlation between all morphological and morphometric traits was calculated (Figure 7). In absolute value, 340 pairwise correlations were higher than 0.75, with the traits of shell almond area, hull area, and perimeter exhibiting the highest number of strong pairwise correlations. High correlations (greater than 0.95) were observed between kernel and shell morphometric traits across different approaches, such as EF-PC1 with PB-PC1. Additionally, strong

correlations were found between these two PC1 components and morphological traits like aspect ratio and ellipse ratio.

Figure 7. Heatmap of the Pearson correlations between all morphological and morphometric traits.

Heritability

Broad sense heritability (H^2) was estimated for all the traits studied here (Figure 8). Fifty-five out of the 73 traits showed heritabilities ≥ 0.25. Weight ratio kernel/shell, shell weight, and shell EF-PC1 showed the highest heritability values (0.9, 0.77 and 0.70 respectively). Between morphometric traits, those related with PC1 showed high heritability values (higher than 0.68), and others such as Kernel PB-PC2, Shell PB-PC2, Shell EF-PC4 and Shell PB-PC3 showed medium values (higher than 0.3).

Figure 8. Broad-sense heritability of all traits studied in the almond populations.

Impact on almond breeding

Nine scientific articles related to almond morphology and breeding were collected to compare the performance of the high-throughput approach implemented in the current study (Supplementary Table 1 and Figure 9). A total of 25,597 kernels were studied (an average of 12,799 per season), along with 21,316 shells (an average of 10,658 per season)

(Figure 9). The entire dataset comprised 660 individuals, and 1,646,236 data points were collected (Figure 9).

Figure 9. Bar plot for comparison among articles related to quantitative almond morphology traits [17,20,21,41–45].

Discussion

The comprehensive workflow presented here introduces a new high-throughput phenotyping tool using almonds as a case of study for fruits. This approach has enabled the analysis of 665 genotypes—the largest dataset for almond morphological quantitatively measured traits reported to date, to the best of the authors' knowledge. The development of this new phenotyping tool marks a breakthrough in addressing the phenotyping bottleneck in almonds. The developed models were adjusted to the target samples used but can be adapted to phenotype other kinds of fruits and other types of organs such as leaves or roots. Furthermore, as it is an open source, it can be used by other plants breeding programs. In fact, the workflow was successfully applied to publicly available image datasets of apples and strawberries [11,13]. Example results and a video tutorial can be found in the workflow's GitHub repository: https://github.com/jorgemasgomez/almondcv2.

Clearly, recent advancements in AI segmentation models, such as YOLO [35] and SAM [28], enable breeding programs to develop fine-tuned models for specific applications, even without large datasets. Additionally, progress in labeling tools like CVAT [29], which integrate AI-assisted features, accelerates the tedious and time-consuming labeling process. The new workflow designed here uses Jupyter notebooks for ease of customisation, eliminating the need for high-performance hardware (e.g., GPUs),

allowing users to run the tool efficiently in cloud-based environments such as Google Colab. Moreover, the reconstruction approaches employed showed good performance in general terms. A lower performance was noticed only in the shell 2022 using SAHI, which may be improved with SAHI parameter optimisation.

The thickness estimation models demonstrated high accuracy, highlighting the usefulness of combining imaging phenotyping tools with traditional phenotyping processes, such as weighing [46]. This approach enhances the phenotyping process by offering an alternative to capturing transversal pictures and makes phenotyping more accessible without relying on more complex instruments like 3D cameras or approaches such as 3D reconstruction [15,21]. However, the approach employed takes advantage of the similar density among kernels, but it would not be effective for shell nuts due to the variability in shell hardness and density.

Morphometric analyses provided new quantitative traits for almond shape breeding. Both Elliptical Fourier Analysis and Pixel-based PCA showed a large variance explained by the PC1 in kernel and shell datasets. A high correlation between the different PCs1 and aspect ratio traits was identified and can also be observed graphically (Figure 3 and Supplementary Figure 7). Indeed, aspect ratio has been highly correlated with PCs1 in tomato and apple leaves [47,48], in pear fruit [49], walnut [41], and in a shape discriminator almond nut [19]. Although the interpretation of some morphometric traits could be abstract, patterns in different parts of the shape can be observed as in the tip (e.g. kernel PB-PC4), top (e.g. kernel PB-PC5), and side curvature (e.g. kernel EF-PC3). Moreover, some morphometric traits (apart from those related to aspect ratio) showed medium heritability values (>0.3) which could place them as breeding targets.

The number of kernels and nuts analyzed in this comparative study is considerably higher than in previous research, and the difference becomes even more pronounced when

comparing metrics per season. Data points collected were from 11 to 123 times higher than those reported in other works [17,20,21,41–45]. More importantly, this progress has been achieved while reducing both time and economic resources, as a single person was able to phenotype the entire data set for one season in just two weeks. Currently, shell cracking remains the primary bottleneck due to the manual process, which is challenging to automate because of variability in shell hardness and size. This extensive dataset will facilitate future studies aimed at dissecting quantitative traits and implementing genomic selection approaches.

Additional Files

Supplementary Table 1. Article metrics studied related to quantitative almond morphological traits.

Supplementary Figure 1. Workflow description outlining the steps involved in developing the segmentation model (green) and deploying it (purple).

Supplementary Figure 2. YOLO metrics obtained during the fine-tuning process for the Kernel-2022 (A), Shell-2022 (B), Kernel-2023 (C), and Shell-2023 (D) datasets. A description of the metrics can be found in (Ultralytics, 2025).

Supplementary Figure 3. Explained variance (%) per EF-PC in the kernel (left) and shell (right) datasets.

Supplementary Figure 4. K-means clustering using EFA-PCA results in the shell dataset, showing the shapes corresponding to each centroid for scenarios ranging from k=1 to k=10.

Supplementary Figure 5. Within group sum of squares decay for K-means clustering using EFA-PCA results in kernel (left) and shell (right) dataset.

Supplementary Figure 6. Explained variance (%) per PB-PC in the kernel (left) and shell (right) datasets.

Supplementary Figure 7. Representation of the influence of the PB-PCs on shape in the kernel (left) and shell (right) datasets, from the mean shape to ±3× standard deviation.

Supplementary Figure 8. K-means clustering using PB-PCA results in the shell dataset, showing the shapes corresponding to each centroid for scenarios ranging from k=1 to k=10.

Supplementary Figure 9. Within group sum of squares decay for K-means clustering using PB-PCA results in kernel (left) and shell (right) dataset.

Abbreviations

EFA: Elliptical Fourier analysis; GPU: Graphics Processing Unit; H2: Broad sense heritability; NGS: Next-generation sequencing; PCA: Principal component analysis; QTL: quantitative trait loci; RMSE: Root mean squared error; ROI: Region of Interest; SAHI: Slicing Aided Hyper Inference; SAM: Segment Anything Model; YOLO: You Only Look Once

Acknowledgements

We would like to thank María del Mar Gómez Abajo, Francisco Gómez Lopez, Lucía Rodríguez Robles, Antonio Moreno Marín, Luis Miguel Serrano Sánchez, and Teresa Cremades Rosado for their assistance with the sampling, as well as Juan Antonio Tudela for lending us his camera and set.

Author contributions

J.M.G. developed the phenotyping workflow, implemented the technical components, and performed image processing and analyses. J.M.G. and P.J.M.G. conceived and designed the study. J.M.G., P.J.M.G., and M.R. carried out field sampling. M.R. and F.D. contributed to the revision and refinement of the manuscript. All authors participated in the writing and revision of the manuscript and approved the final version.

Funding

This work was supported by grant PID2021-127421OB-I00 funded by MICIU/AEI/10.13039/501100011033, by grant CNS2022-135936 funded by MICIU/AEI /10.13039/501100011033 and European Union NextGenerationEU/PRTR and by “ERDF A way of making Europe” and formed part of the AGROALNEXT program and was supported by MCIN with funding from European Union NextGenerationEU (PRTR-C17.I1) and by the Fundación Séneca with funding from Comunidad Autónoma Región de Murcia (CARM). J.M.-G acknowledges the Spanish MICIU for his predoctoral grant (FPU20/00614).

Availability of source code and requirements

Project name: AlmondCV

Project home page: https://github.com/jorgemasgomez/almondcv2

Operating system(s): Platform independent

Programming language: Python, R

Other requirements: see public environment file released under GNU GPL v3

Data availability

The data supporting the results of this article are available in the Zenodo repository [50].

Temporal restricted link for reviewers.

Competing interests

The authors declare that they have no competing interests.

References

1. Singh A, Ganapathysubramanian B, Singh AK, Sarkar S. Machine Learning for High-Throughput Stress Phenotyping in Plants. *Trends Plant Sci*. 2016; doi: 10.1016/j.tplants.2015.10.015.

2. Kim JY. Roadmap to High Throughput Phenotyping for Plant Breeding. *J Biosyst Eng*. 2020; doi: 10.1007/s42853-020-00043-0.

3. Bhat JA, Ali S, Salgotra RK, Mir ZA, Dutta S, Jadon V, et al.. Genomic Selection in the Era of Next Generation Sequencing for Complex Traits in Plant Breeding. *Front Genet*. Frontiers; 2016; doi: 10.3389/fgene.2016.00221.

4. Wetterstrand K: DNA Sequencing Costs: Data. https://www.genome.gov/about-genomics/fact-sheets/DNA-Sequencing-Costs-Data (2013). Accessed 2025 Jan 31.

5. Yang W, Feng H, Zhang X, Zhang J, Doonan JH, Batchelor WD, et al.. Crop Phenomics and High-Throughput Phenotyping: Past Decades, Current Challenges, and Future Perspectives. *Molecular Plant*. 2020; doi: 10.1016/j.molp.2020.01.008.

6. Moore CR, Johnson LS, Kwak I-Y, Livny M, Broman KW, Spalding EP. High-throughput computer vision introduces the time axis to a quantitative trait map of a plant growth response. *Genetics*. 2013; doi: 10.1534/genetics.113.153346.

7. Sheikh M, Iqra F, Ambreen H, Pravin KA, Ikra M, Chung YS. Integrating artificial intelligence and high-throughput phenotyping for crop improvement. *Journal of Integrative Agriculture*. 2024; doi: 10.1016/j.jia.2023.10.019.

8. Condorelli GE, Maccaferri M, Newcomb M, Andrade-Sanchez P, White JW, French AN, et al.. Comparative Aerial and Ground Based High Throughput Phenotyping for the Genetic Dissection of NDVI as a Proxy for Drought Adaptive Traits in Durum Wheat. *Front Plant Sci*. Frontiers; 2018; doi: 10.3389/fpls.2018.00893.

9. Huang Y, Ren Z, Li D, Liu X. Phenotypic techniques and applications in fruit trees: a review. *Plant Methods*. 2020; doi: 10.1186/s13007-020-00649-7.

10. Kirchgessner N, Hodel M, Studer B, Patocchi A, Broggini GAL. FruitPhenoBox – a device for rapid and automated fruit phenotyping of small sample sizes. *Plant Methods*. 2024; doi: 10.1186/s13007-024-01206-2.

11. Keller B, Jung M, Bühlmann-Schütz S, Hodel M, Studer B, Broggini GAL, et al.. The genetic basis of apple shape and size unraveled by digital phenotyping. *G3 (Bethesda)*. 2024; doi: 10.1093/g3journal/jkae045.

12. Jurado-Ruiz F, Rousseau D, Botía JA, Aranzana MJ. GenoDrawing: An Autoencoder Framework for Image Prediction from SNP Markers. *Plant Phenomics*. 2023; doi: 10.34133/plantphenomics.0113.

13. Feldmann MJ, Hardigan MA, Famula RA, López CM, Tabb A, Cole GS, et al.. Multi-dimensional machine learning approaches for fruit shape phenotyping in strawberry. *GigaScience*. 2020; doi: 10.1093/gigascience/giaa030.

14. Zingaretti LM, Monfort A, Pérez-Enciso M. Automatic Fruit Morphology Phenome and Genetic Analysis: An Application in the Octoploid Strawberry. *Plant Phenomics*. 2021; doi: 10.34133/2021/9812910.

15. Feldmann MJ, Tabb A. Cost-effective, high-throughput phenotyping system for 3D reconstruction of fruit form. *The Plant Phenome Journal*. 2022; doi: 10.1002/ppj2.20029.

16. Janick J, Schirra M. Postharvest technology and utilization of almonds. *Horticultural Reviews*. 1997;

17. Martínez-García PJ, Rubio M, Cremades T, Dicenta F. Inheritance of shell and kernel shape in almond (Prunus dulcis). *Scientia Horticulturae*. 2019; doi: 10.1016/j.scienta.2018.09.041.

18. Socias R, Kodad O, Alonso J, Gradziel T. Almond Quality: A Breeding Perspective. *Horticultural Reviews*. 2008;

19. Demir B, Sayinci B, Çetin N, Yaman M, Çömlek R. Shape Discrimination of Almond Cultivars by Elliptic Fourier Descriptors. *Erwerbs-Obstbau*. 2019; doi: 10.1007/s10341-019-00423-7.

20. Pérez de Los Cobos F, Romero A, Lipan L, Miarnau X, Arús P, Eduardo I, et al.. QTL mapping of almond kernel quality traits in the F1 progeny of “Marcona” × “Marinada.” *Front Plant Sci*. 2024; doi: 10.3389/fpls.2024.1504198.

21. Sánchez-Beeckman M, Fornés Comas J, Martorell O, Alonso Segura JM, Buades A. Three-dimensional image analysis for almond endocarp feature extraction and shape description. *Computers and Electronics in Agriculture*. 2024; doi: 10.1016/j.compag.2024.109420.

22. Ngugi LC, Abelwahab M, Abo-Zahhad M. Recent advances in image processing techniques for automated leaf pest and disease recognition – A review. *Information Processing in Agriculture*. 2021; doi: 10.1016/j.inpa.2020.04.004.

23. Sapkota R, Ahmed D, Karkee M. Comparing YOLOv8 and Mask R-CNN for instance segmentation in complex orchard environments. *Artificial Intelligence in Agriculture*. 2024; doi: 10.1016/j.aiia.2024.07.001.

24. Xue W, Ding H, Jin T, Meng J, Wang S, Liu Z, et al.. CucumberAI: Cucumber Fruit Morphology Identification System Based on Artificial Intelligence. *Plant Phenomics*. American Association for the Advancement of Science; 2024; doi: 10.34133/plantphenomics.0193.

25. Katal N, Rzanny M, Mäder P, Wäldchen J. Deep Learning in Plant Phenological Research: A Systematic Literature Review. *Front Plant Sci*. Frontiers; 2022; doi: 10.3389/fpls.2022.805738.

26. Gu W, Bai S, Kong L. A review on 2D instance segmentation based on deep neural networks. *Image and Vision Computing*. 2022; doi: 10.1016/j.imavis.2022.104401.

27. Bommasani R, Hudson DA, Adeli E, Altman R, Arora S, Arx S von, et al.. On the Opportunities and Risks of Foundation Models. arXiv;

28. Kirillov A, Mintun E, Ravi N, Mao H, Rolland C, Gustafson L, et al.. Segment Anything. *2023 IEEE/CVF International Conference on Computer Vision (ICCV).*

29. Sekachev B, Manovich N, Zhiltsov M, Zhavoronkov A, Kalinin D, Hoff B, et al.. opencv/cvat: v1.1.0. Zenodo;

30. Akyon FC, Onur Altinuc S, Temizel A. Slicing Aided Hyper Inference and Fine-Tuning for Small Object Detection. *2022 IEEE International Conference on Image Processing (ICIP).*

31. Gehan MA, Fahlgren N, Abbasi A, Berry JC, Callen ST, Chavez L, et al.. PlantCV v2: Image analysis software for high-throughput plant phenotyping. *PeerJ*. PeerJ Inc.; 2017; doi: 10.7717/peerj.4088.

32. : OpenCV: Camera Calibration. https://docs.opencv.org/4.x/dc/dbb/tutorial_py_calibration.html (2025). Accessed 2025 Jan 31.

33. Pereira A, Santos C, Aguiar M, Welfer D, Dias M, Ribeiro M, et al.. Detection of retinal microlesions through YOLOR-CSP architecture and image slicing with the SAHI algorithm. *2023 International Joint Conference on Neural Networks (IJCNN).*

34. Saradopoulos I, Potamitis I, Rigakis I, Konstantaras A, Barbounakis IS. Image Augmentation Using Both Background Extraction and the SAHI Approach in the Context of Vision-Based Insect Localization and Counting. *Information*. Multidisciplinary Digital Publishing Institute; 2025; doi: 10.3390/info16010010.

35. Redmon J, Divvala S, Girshick R, Farhadi A. You Only Look Once: Unified, Real-Time Object Detection. *2016 IEEE Conference on Computer Vision and Pattern Recognition (CVPR).*

36. : Train - Ultralytics YOLO Docs. https://docs.ultralytics.com/es/modes/train/ (2025). Accessed 2025 Jan 31.

37. Bradski G, Kaehler A. OpenCV. *Dr Dobb's journal of software tools*. 32000;

38. : OpenCV: Image Segmentation with Watershed Algorithm. https://docs.opencv.org/4.x/d3/db4/tutorial_py_watershed.html (2025). Accessed 2025 Jan 31.

39. Bonhomme V, Picq S, Gaucherel C, Claude J. Momocs: Outline Analysis Using R. *Journal of Statistical Software*. 2014; doi: 10.18637/jss.v056.i13.

40. Seabold S, Perktold J. Statsmodels: Econometric and Statistical Modeling with Python. *scipy*. 2010; doi: 10.25080/Majora-92bf1922-011.

41. Demir B, Sayıncı B, Çetin N, Yaman M, Çömlek R, Aydın Y, et al.. Elliptic Fourier based analysis and multivariate approaches for size and shape distinctions of walnut (Juglans regia L.) cultivars. *Grasas y Aceites*. 2018; doi: 10.3989/gya.0104181.

42. Fernández i Martí A, Font i Forcada C, Socias i Company R. Genetic analysis for physical nut traits in almond. *Tree Genetics & Genomes*. 2013; doi: 10.1007/s11295-012-0566-8.

43. Forcada CF i, Oraguzie N, Reyes-Chin-Wo S, Espiau MT, Company RS i, Martí AF i. Identification of Genetic Loci Associated with Quality Traits in Almond via Association Mapping. *PLOS ONE*. Public Library of Science; 2015; doi: 10.1371/journal.pone.0127656.

44. Goonetilleke SN, Wirthensohn MG, Mather DE. Genetic analysis of quantitative variation in almond nut traits. *Tree Genetics & Genomes*. 2023; doi: 10.1007/s11295-023-01630-w.

45. Sorkheh K, Shiran B, Khodambashi M, Moradi H, Gradziel TM, Martínez-Gómez P. Correlations between quantitative tree and fruit almond traits and their implications for breeding. *Scientia Horticulturae*. 2010; doi: 10.1016/j.scienta.2010.04.014.

46. Utai K, Nagle M, Hämmerle S, Spreer W, Mahayothee B, Müller J. Mass estimation of mango fruits (Mangifera indica L., cv. ‘Nam Dokmai’) by linking image processing and artificial neural network. *Engineering in Agriculture, Environment and Food*. 2019; doi: 10.1016/j.eaef.2018.10.003.

47. Chitwood DH, Kumar R, Headland LR, Ranjan A, Covington MF, Ichihashi Y, et al.. A Quantitative Genetic Basis for Leaf Morphology in a Set of Precisely Defined Tomato Introgression Lines. *The Plant Cell*. 2013; doi: 10.1105/tpc.113.112391.

48. Migicovsky Z, Li M, Chitwood DH, Myles S. Morphometrics Reveals Complex and Heritable Apple Leaf Shapes. *Front Plant Sci*. Frontiers; 2018; doi: 10.3389/fpls.2017.02185.

49. Wang H, Yin H, Li H, Wu G, Guo W, Qi K, et al.. Quantitative 2D fruit shape analysis of a wide range of pear genetic resources toward shape design breeding. *Scientia Horticulturae*. 2024; doi: 10.1016/j.scienta.2023.112826.

50. Mas-Gómez J, Rubio M, Dicenta F, Martínez-García PJ. Dataset AlmondCV Pictures. 2025; doi: 10.5281/zenodo.15423562.

Click here to access/download;Figure;Figure1.png

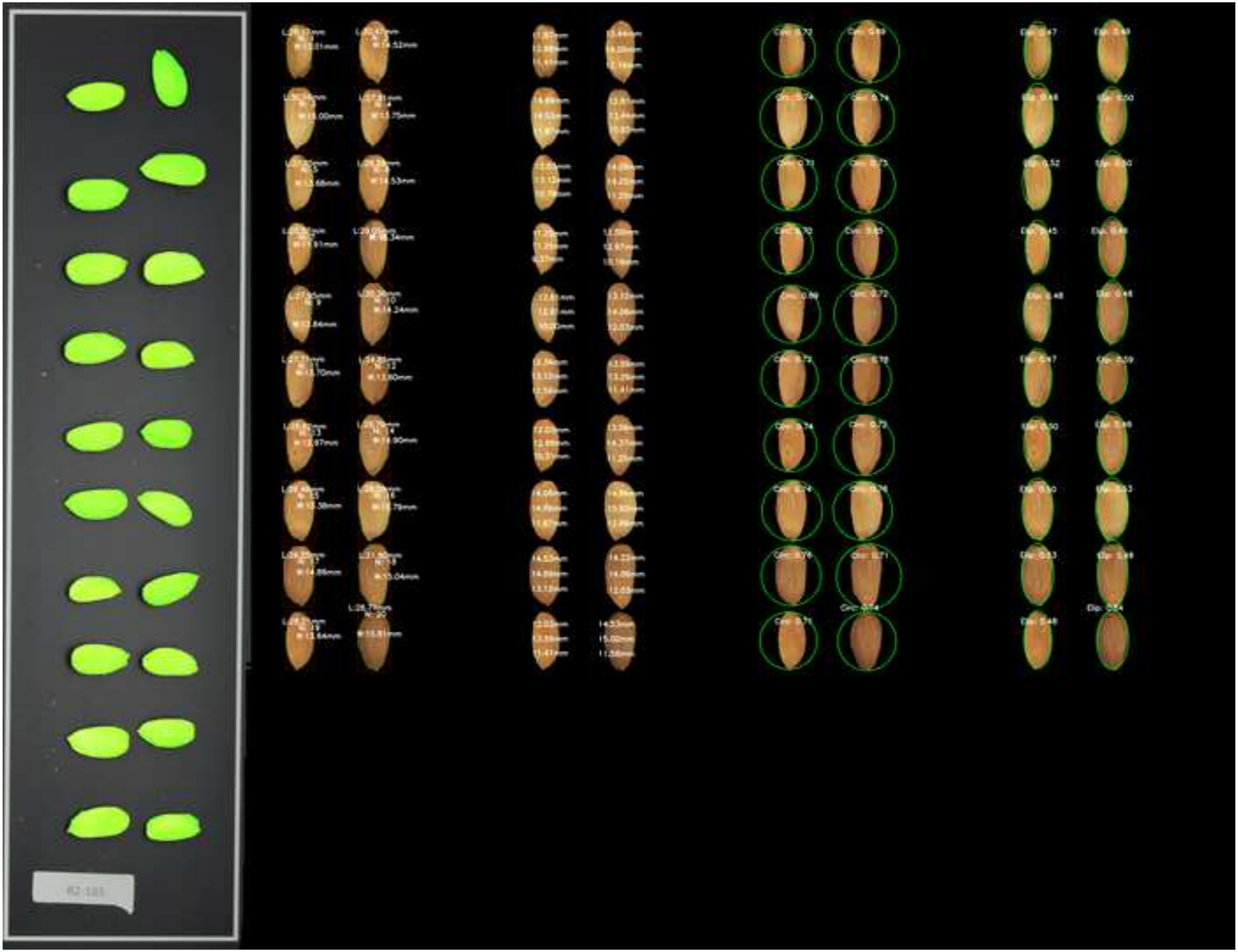

Figure_2A

Click here to access/download;Figure;Figure2A.png

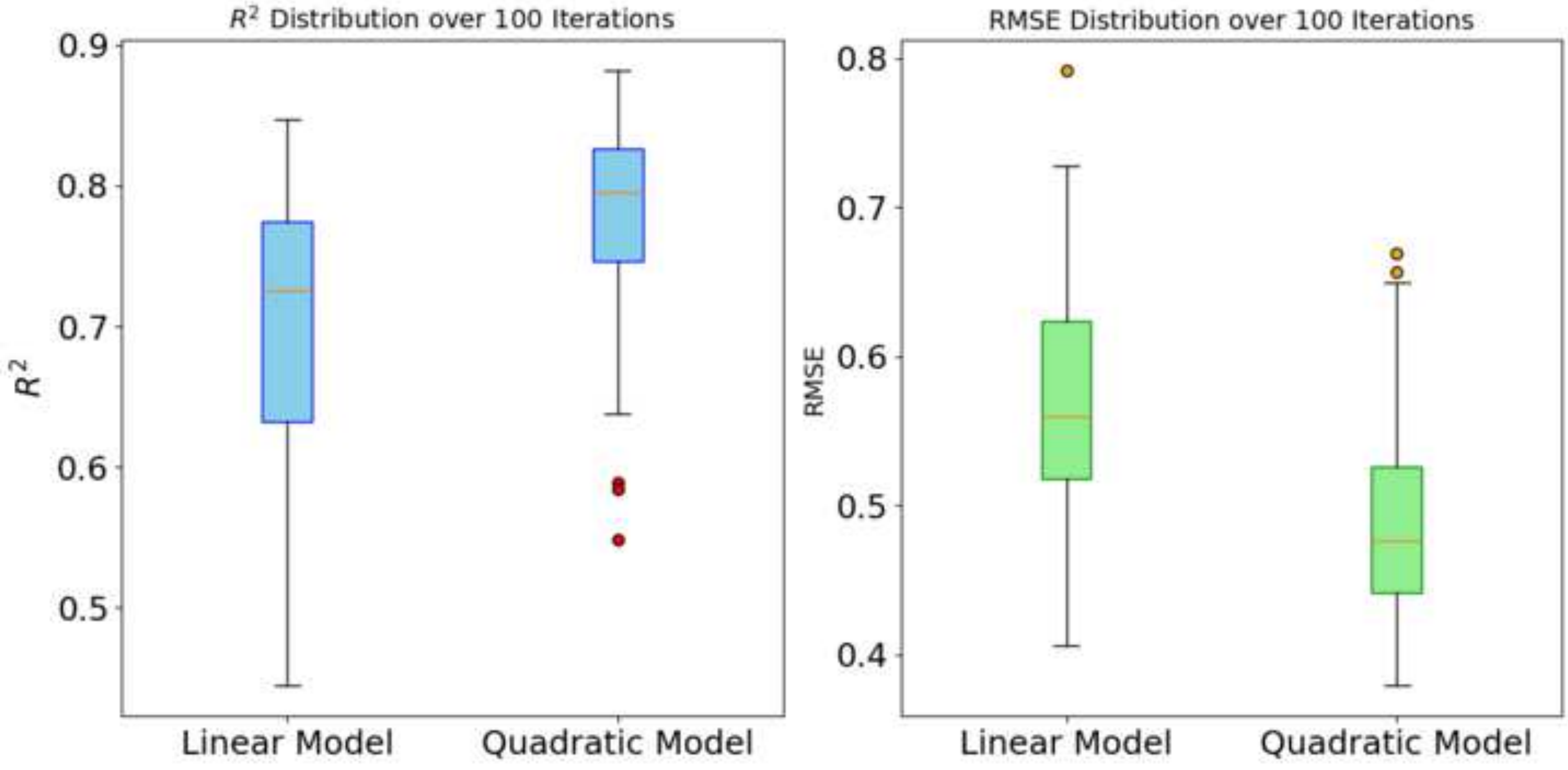

Figure_2B

Click here to access/download;Figure;Figure2B.png

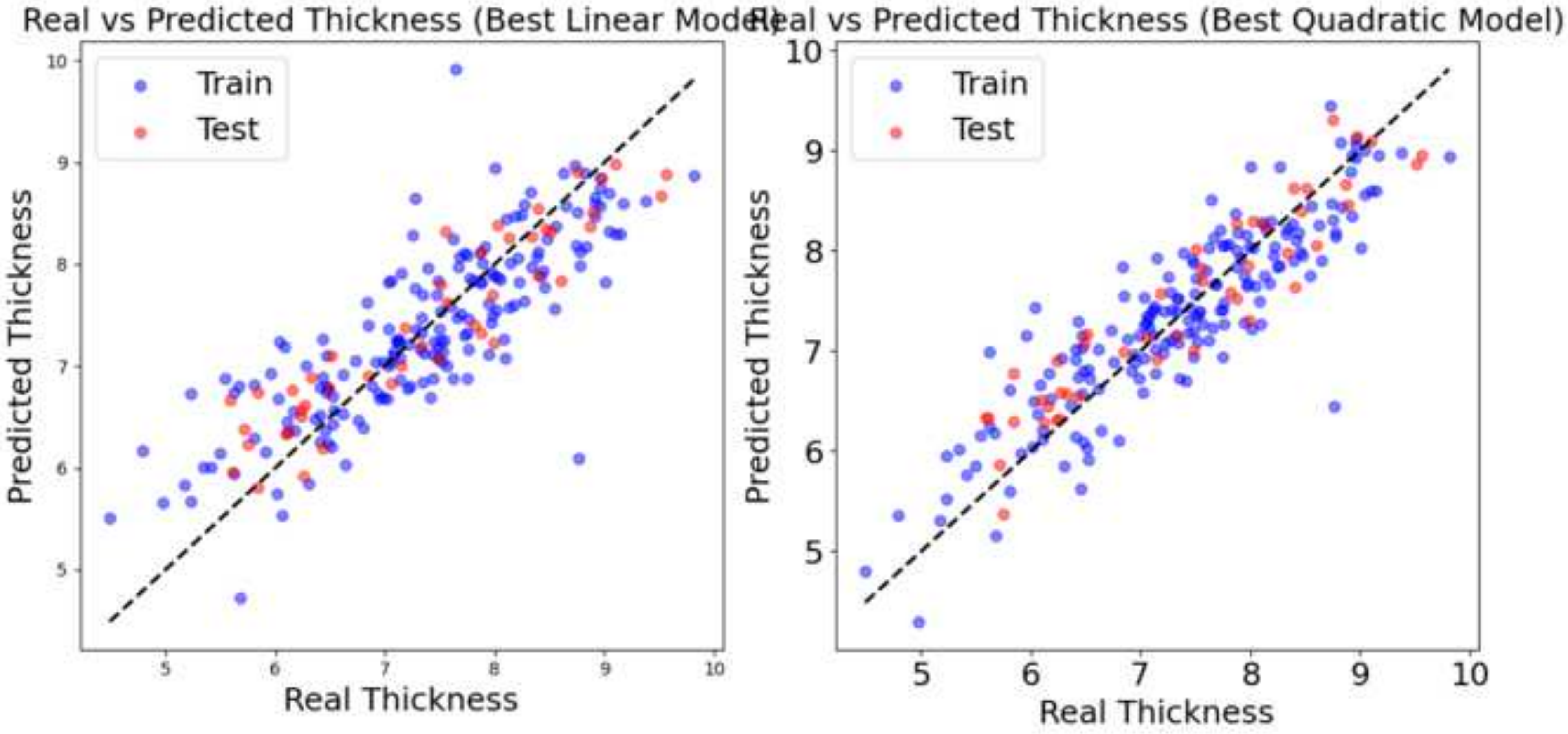

Figure_3

Click here to access/download;Figure;Figure3.jpg

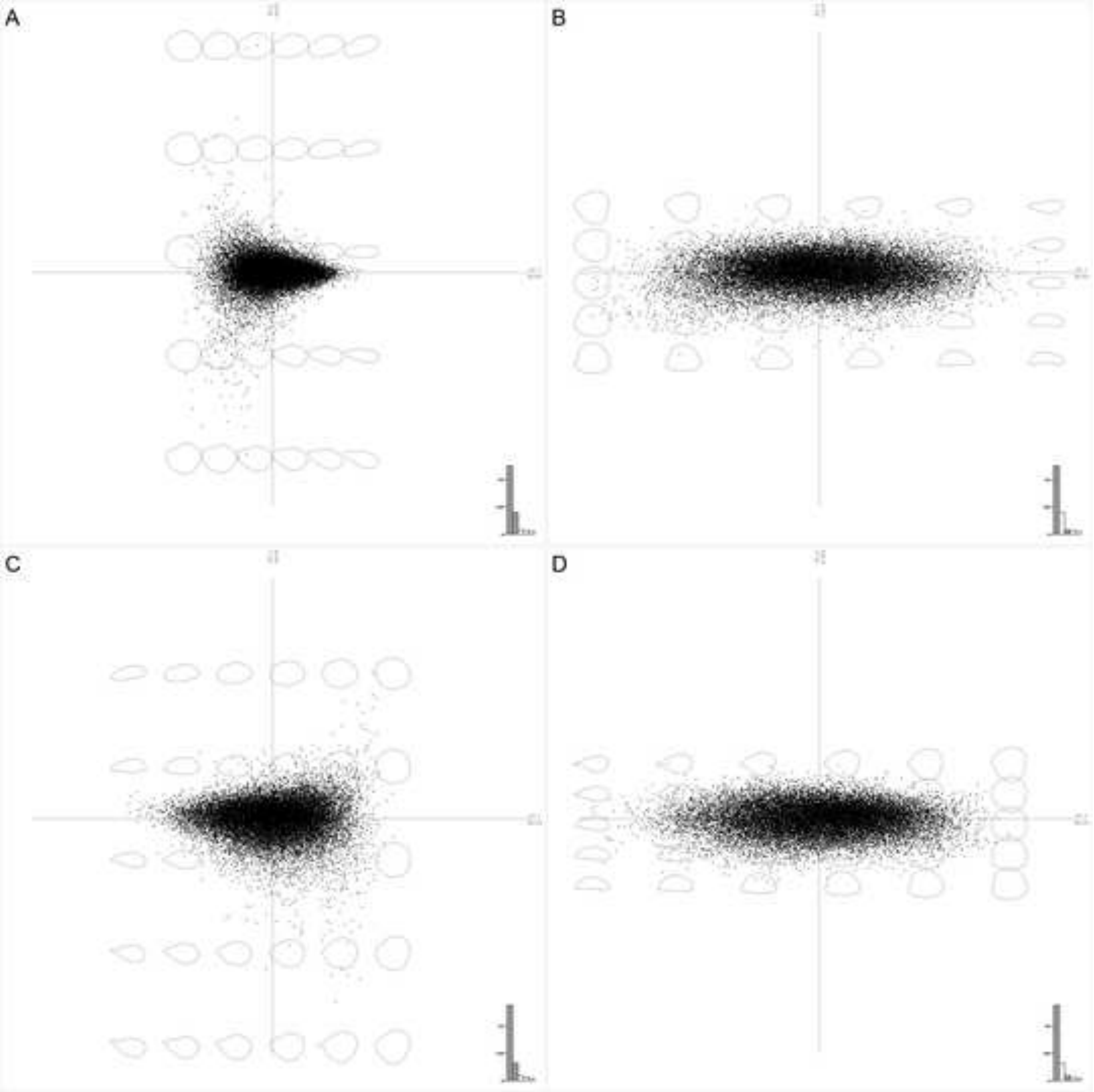

Figure_4 Click here to access/download;Figure;Figure4.jpg

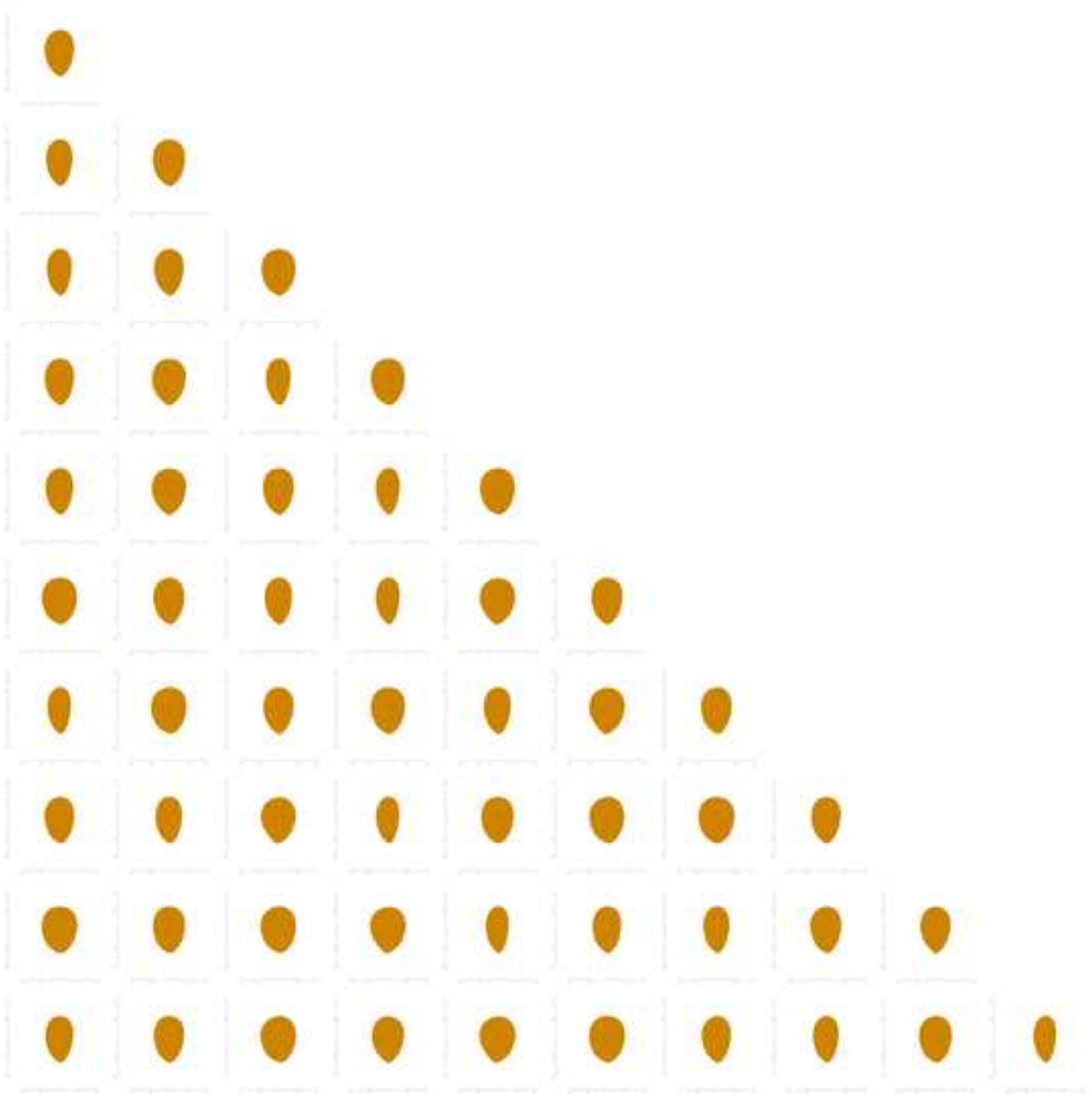

Figure_5

Click here to access/download;Figure;Figure5.png

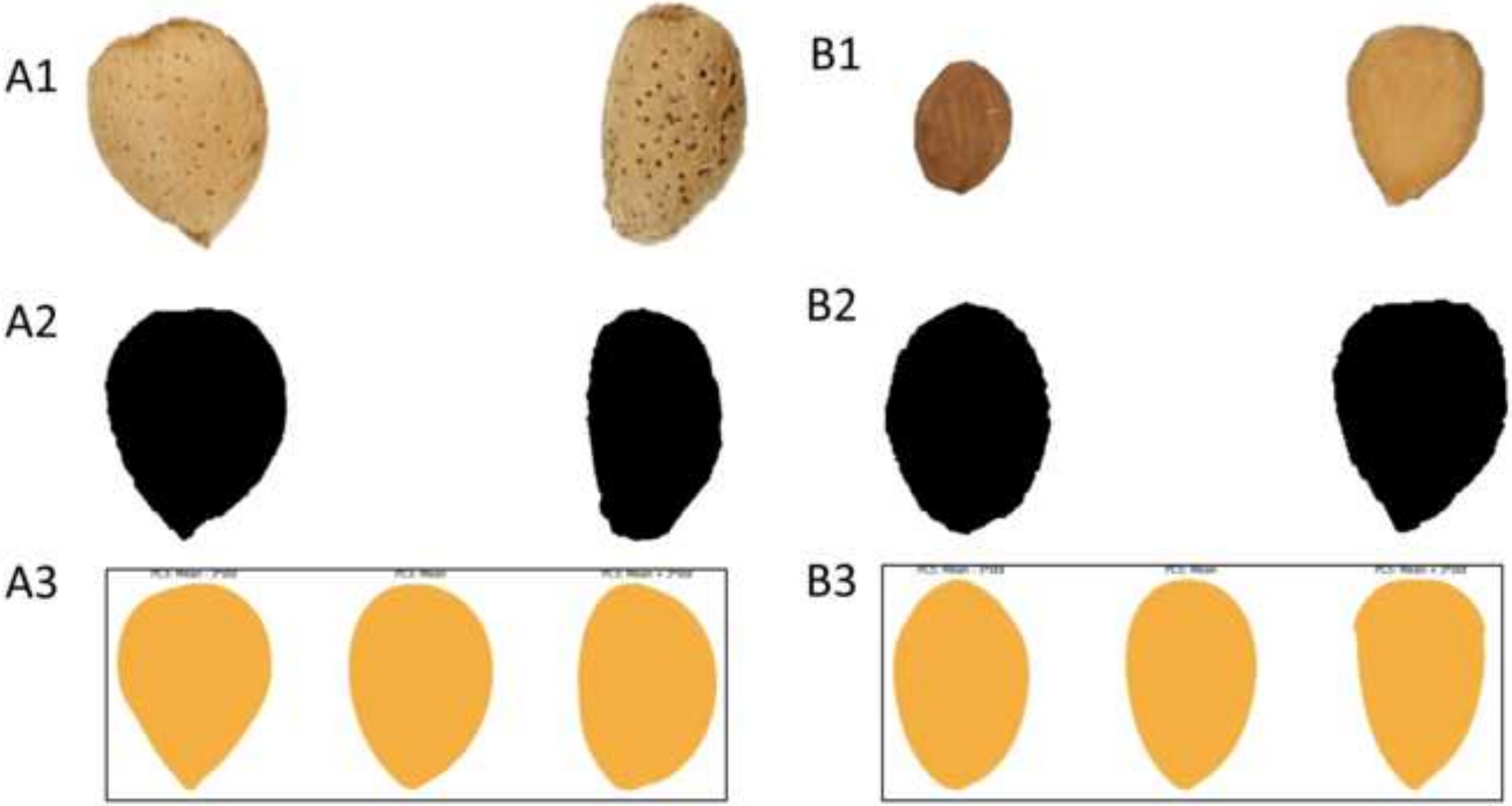

Figure_6

Click here to access/download;Figure;Figure6.jpg

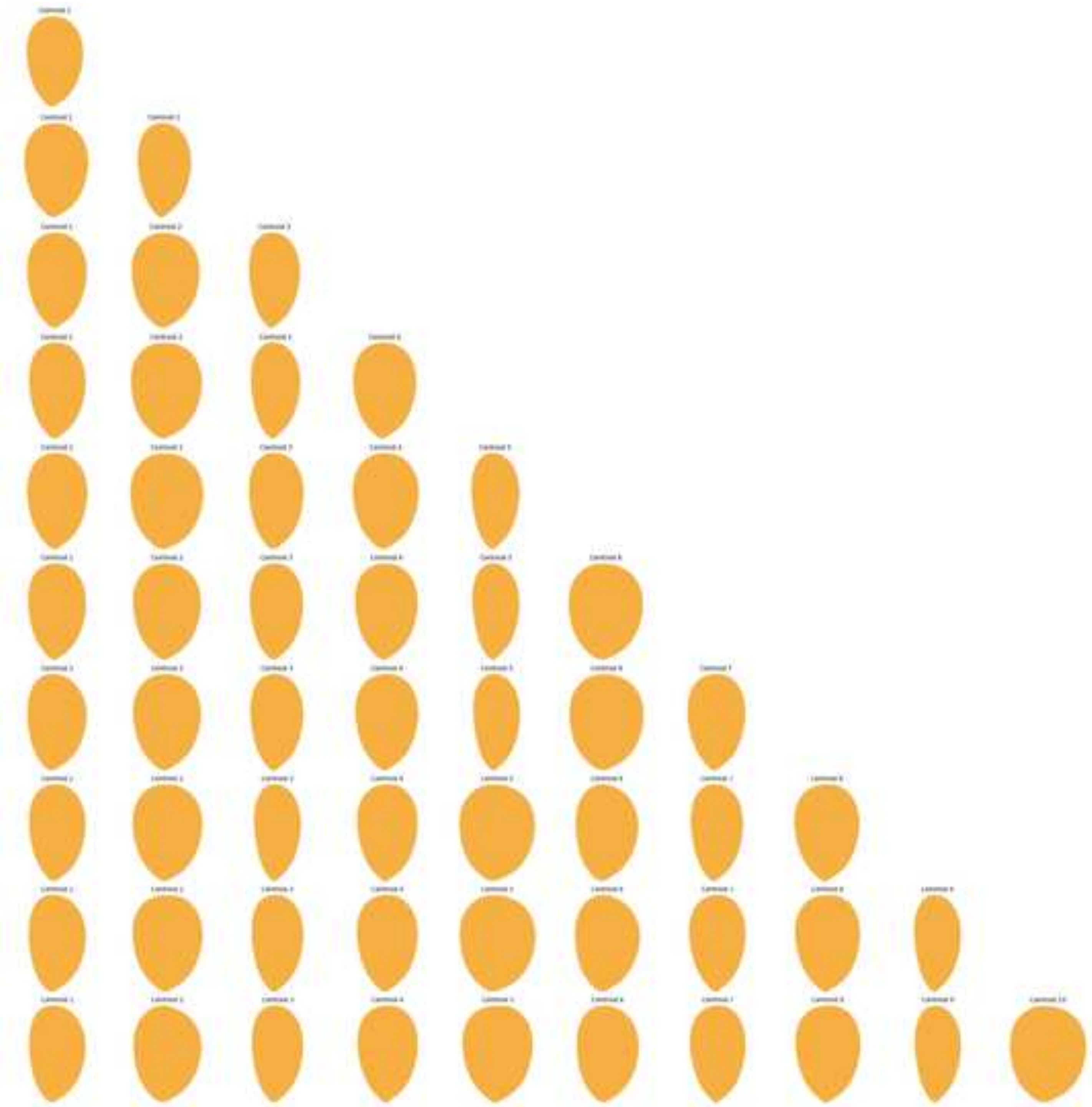

Figure_7

Click here to access/download;Figure;Figure7.png

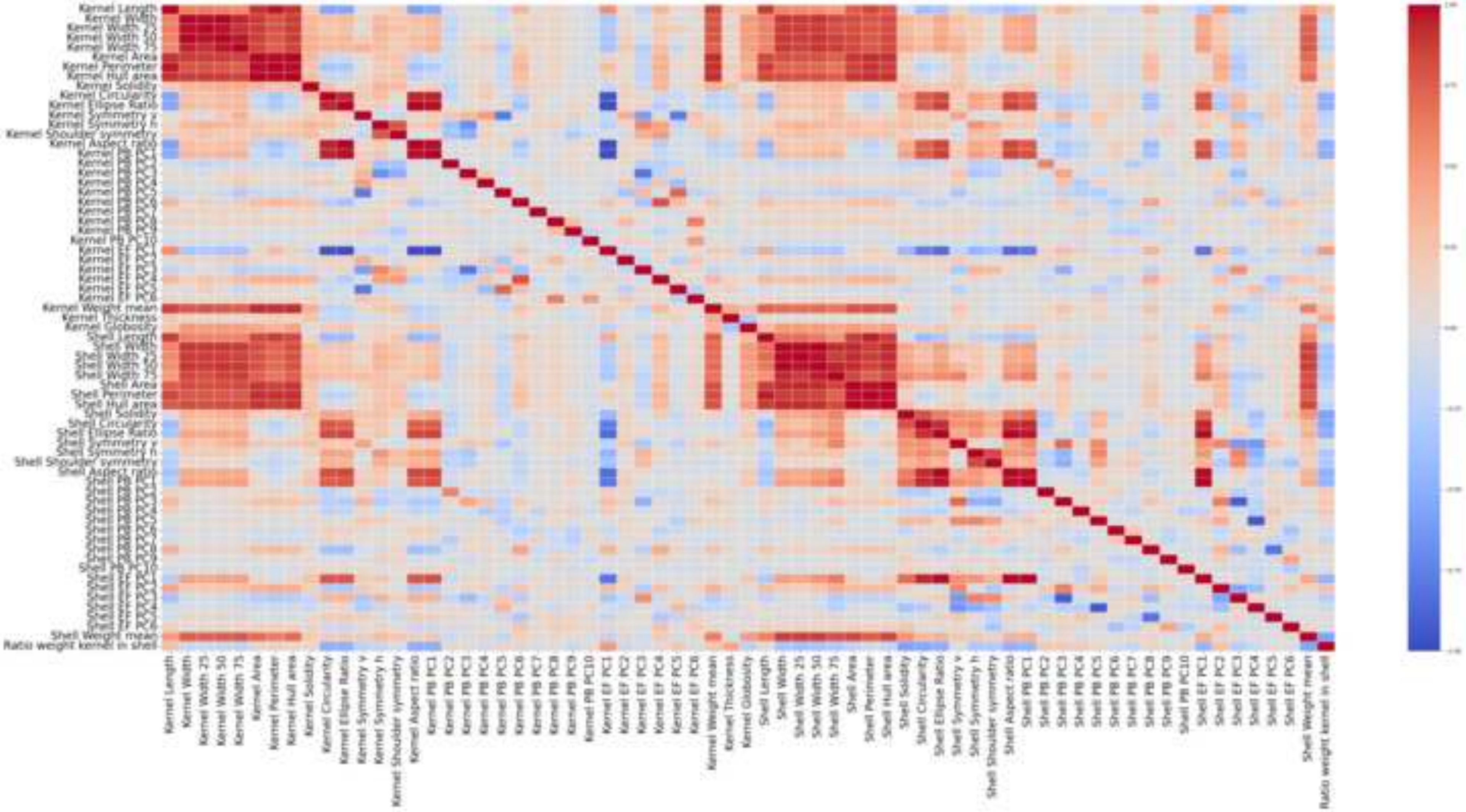

Figure_8

Click here to access/download;Figure;Figure8.png

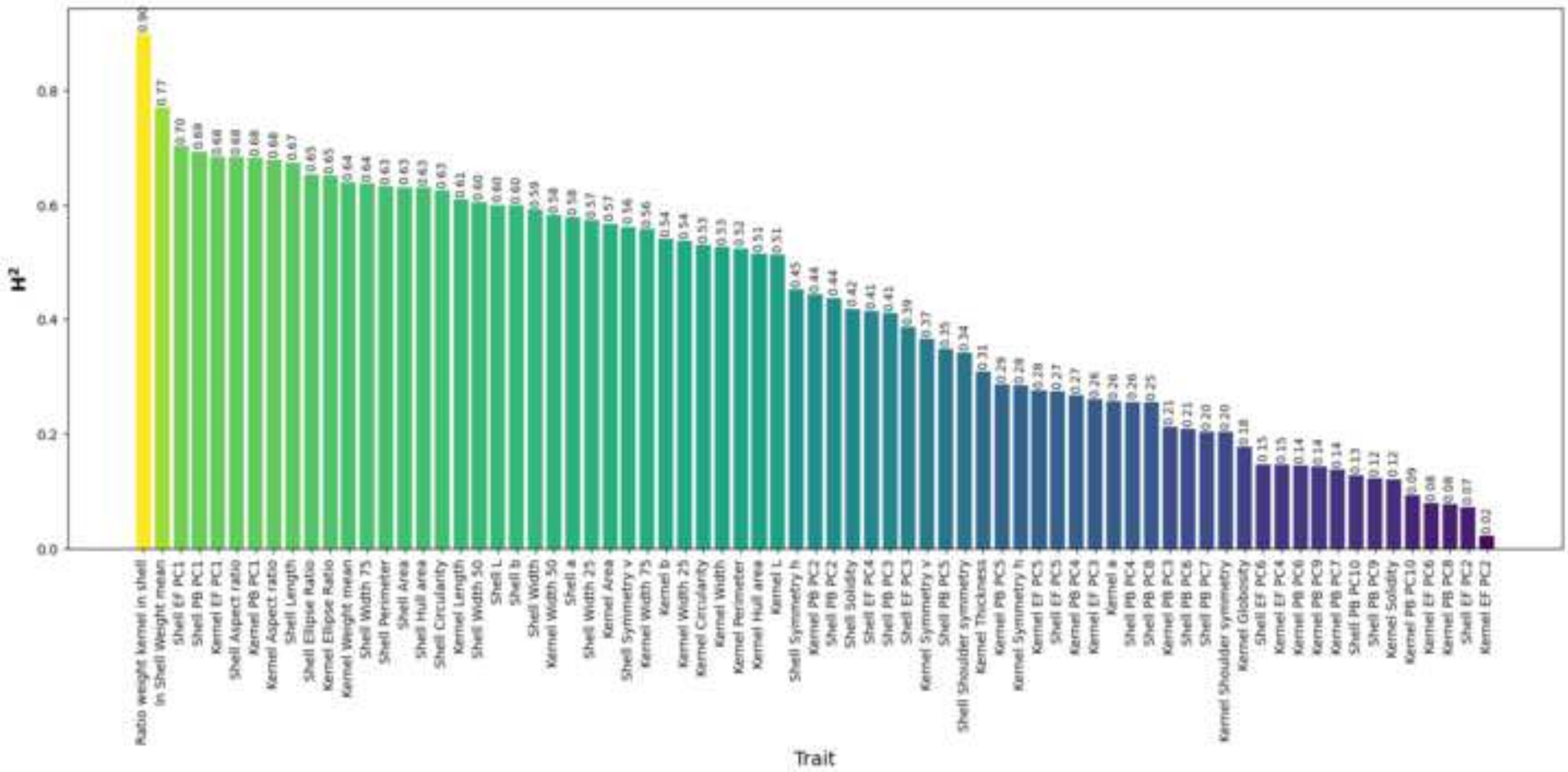

Figure_9

Click here to access/download;Figure;Figure9.png

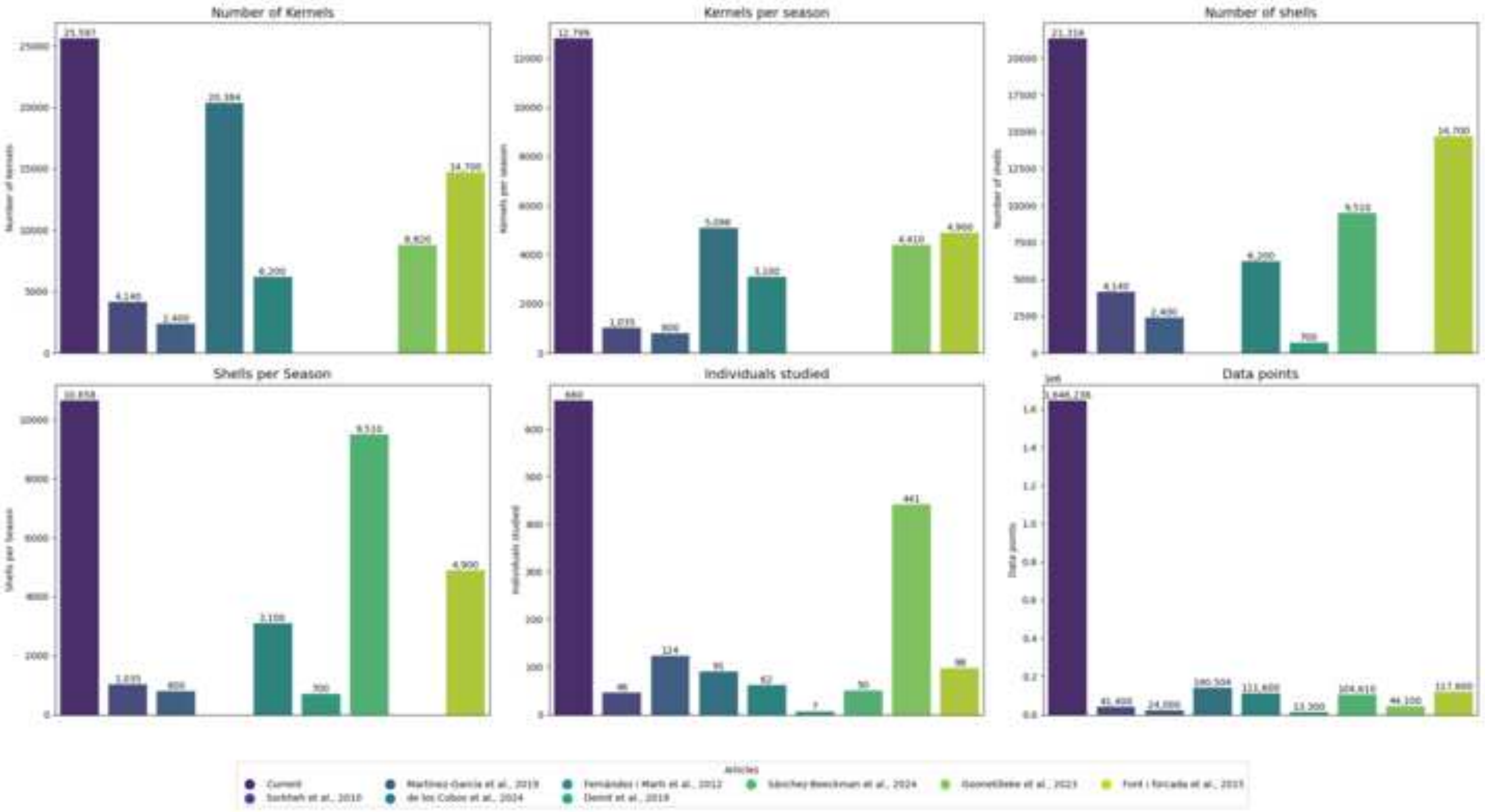

Supplementary Figures

Click here to access/download
Supplementary Material
Supplemmentary_Figures.docx

Supplementary Tables

Click here to access/download
Supplementary Material
Supplementary_tables.xlsx

Cover_letter Click here to access/download;Personal Cover;Cover_letter_Gigascience.pdf

Dear Editor,

We are pleased to submit an original research article entitled “Open RGB Imaging Workflow for Morphological and Morphometric Analysis of Fruits using AI: A Case Study on Almonds” for your consideration for publication in the Thematic Series “Plant Phenomics: Data Integration and Analyses” of GigaScience.

This manuscript presents a new open-access workflow for the morphological and morphometric analysis of fruits using AI, integrating multiple analytical steps into a single workflow — from image calibration to advanced morphometric analysis. Using this high-throughput phenotyping approach, we generated the largest dataset of quantitative morphological traits in almond to date, with over 45,000 kernels and shells phenotyped across 660 genotypes and more than 1.5 million data points collected. This improvement in phenotyping efficiency will enhance genomic selection strategies in breeding programs by incorporating accurate quantitative morphological traits, thereby increasing selection accuracy. Moreover, the complex genetic architecture of morphological and morphometric traits makes quantitative data essential for dissecting the underlying genetic basis.

Although almonds were used as the primary case study, we also tested the workflow on apples and strawberries by leveraging publicly available fruit image datasets. The tool includes several novel features that enhance accessibility, such as the ability to fine-tune pre-trained models, enabling users to develop their own segmentation models with minimal training data. Furthermore, the workflow is adaptable to other plant organs such as leaves or flowers. Its accessibility and adaptability for different purposes have contributed to the positive reception of this work by the *Rosaceae* genomics community, and it was recently awarded Best PhD Talk at the 12th *Rosaceae* Genomics Conference (Barcelona, 2025).

We believe this manuscript fits well within the scope of the Thematic Series and represents a valuable resource for breeders and researchers alike. We declare no conflicts of interest, and all authors have approved the manuscript for submission. We also confirm that this work has not been published in any other scientific journal. A preprint version has been shared on bioRxiv to facilitate early dissemination, in alignment with your journal’s Editorial Policies and Open Science Reporting Standards.

Thank you for considering our manuscript. We look forward to the possibility of contributing to your journal.

Sincerely,

Pedro José Martínez-García
Tenured Scientist
Department of Plant Breeding
Centro de Edafología y Biología Aplicada del Segura (CEBAS)
Spanish National Research Council (CSIC)
