# Supplementary material for: Open RGB imaging workflow for morphological and morphometric analysis of fruits using deep learning: a case study on almonds
Source: Gigascience. 2025 Dec 19;15:giaf157. doi: 10.1093/gigascience/giaf157 (PMC12970599; doi:10.1093/gigascience/giaf157)
Supplement: giaf157_GIGA-D-25-00218_Revision_1 [file giaf157_giga-d-25-00218_revision_1.pdf]

GigaScience

Open RGB Imaging Workflow for Morphological and Morphometric Analysis of Fruits using Deep Learning: A Case Study on Almonds.

--Manuscript Draft--

**Manuscript Number:**	GIGA-D-25-00218R1
**Full Title:**	Open RGB Imaging Workflow for Morphological and Morphometric Analysis of Fruits using Deep Learning: A Case Study on Almonds.
**Article Type:**	Research
**Funding Information:**	Ministerio de Ciencia e Innovación (PID2021-127421OB-I00) — PhD Dicenta Federico; Ministerio de Ciencia e Innovación (CNS2022-135936) — PhD Martínez-García José Pedro; Ministerio de Universidades (FPU20/00614) — Sr Jorge Mas Gómez

Abstract:

Abstract

Background

High-throughput phenotyping is addressing the current bottleneck in phenotyping within breeding programs. Imaging tools are becoming the primary resource for improving the efficiency of phenotyping processes and providing large datasets for genomic selection approaches. The advent of AI brings new advantages by enhancing phenotyping methods using imaging, making them more accessible to breeding programs. In this context, we have developed an open Python workflow for analyzing morphology, colour and morphometric traits using AI, which can be applied to fruits and other plant organs.

Results

The workflow was implemented in almond (Prunus dulcis (Mill.) D. A. Webb), a species where breeding efficiency is critical due to its long breeding cycle. Over 25,000 kernels, more than 20,000 nuts, and over 600 individuals were phenotyped, making this the largest morphological study conducted in almond so far. The best segmentation and reconstruction approaches achieved error rates below 1%. Weight and area variables enabled accurate estimation of kernel thickness, with a root mean squared error (RMSE) of 0.47. Fifty-five heritable morphological, morphometric and colour traits were identified, highlighting their potential as target traits in breeding programs.

Conclusion

The proposed workflow demonstrated robust performance across diverse datasets and being effective with limited training data for fine-tuning. Its compatibility with the output of AI-based labelling tools allows users to fully leverage the advantages of these technologies—reducing manual effort, accelerating dataset preparation, and streamlining the fine-tuning process of segmentation models. This flexibility enhances the scalability and practical applicability of the workflow in real-world phenotyping scenarios, especially in the context of breeding programs.

**Corresponding Author:**	Martínez-García José Pedro<br>Centro de Edafologia y Biologia Aplicada del Segura<br>Murcia, SPAIN
**Corresponding Author Secondary Information:**	
**Corresponding Author's Institution:**	Centro de Edafologia y Biologia Aplicada del Segura
**Corresponding Author's Secondary Institution:**	
**First Author:**	Jorge Mas Gómez
**First Author Secondary Information:**	
**Order of Authors:**	Jorge Mas Gómez
	Rubio Manuel

Powered by Editorial Manager® and ProduXion Manager® from Aries Systems Corporation

	Dicenta Federico
	Martínez-García José Pedro
**Order of Authors Secondary Information:**	

Response to Reviewers:

Dear Editor and Reviewers,
We sincerely appreciate your contributions and comments and proceed to address your suggestions point-by-point in this document. Responses are in red color.
Editor reports:
We would like to confirm that the new software has been registered in the bio.tools and SciCrunch.org databases and a RRID (Research Resource Identification Initiative ID) and biotoolsID identifiers were received, and it is now included in the manuscript. Also a new title has been provided according to reviewer#1 comments: “Open RGB Imaging Workflow for Morphological and Morphometric Analysis of Fruits using deep learning: A Case Study on Almonds.”

Reviewer reports:

Reviewer #1: The methods described in this article represent a useful tool for fast and reliable morphometric analysis of almonds with potential applications in fruits. The pipeline is technically sound, and publicly available workflow will advance the adoption of this technology. However, there are critical concerns which needs to be addressed before the manuscript could be further proceeded for publication in the journal –
Response: Thank you for your comments.
Major Comments -
1. Authors claim this technique as a new phenotyping tool with breakthrough implications; however, I object to this claim. Numerous studies have utilized this technique in plant phenotyping to the extent that labeling it as a new phenotyping tool may not be ideal. Additionally, for kernel or seed morphometrics, a wide array of user-friendly, open-source tools have already been developed and are readily available, for example,
- SeedExtractor https://www.frontiersin.org/journals/plant-science/articles/10.3389/fpls.2020.581546/full#h3
- SmartGrain https://academic.oup.com/plphys/article/160/4/1871/6109568
- GrainScan https://link.springer.com/article/10.1186/1746-4811-10-23
- PlantCV https://peerj.com/articles/4088/

These tools and a lot of other options can be readily used for almond kernel morphometrics. Authors are requested to discuss/compare advantages/performance of their model with SeedExtractor, SmartGrain, and GrainScan.
Response: Thank you for your comments. It is clear that the mentioned software are valuable and useful tools for high-throughput phenotyping. However, to better highlight the advantages of our phenotyping workflows over these tools, we have summarized them in the following table.
Key pointAlmondCVSeedExtractorSmartGrainGrainScanPlantCV
Open-SourceYesYes, for standalone versionNoNoYes
Availability for all operative systemsYesYesNoNoYes
LanguagePython mainly. Complements in RMatlabVisual C++NAPython
Online runYesNoNoNoYes
Color measureYesYesNAYesAvailable functions
Morphology measuresLength, width, area, perimeter, hull-area, solidity, aspect ratio, circularity, ellipse ratio, colour (L*a*b* model), width at three different heights (25, 50 and 75% of the length), vertical and horizontal symmetry and shoulder symmetry, estimation of the thickness, globosityarea, perimeter,length, width), circularity, seed number, color intensity Area, perimeter length, circularity, length, width, length-to-width ratio, intersection of length and width, center of gravity, Area, length, width, color area, convex_hull_area, solidity, perimeter, width, height, longest_path, center_of_mass, convex_hull_vertices, object_in_frame, ellipse_center, ellipse_major_axis, ellipse_minor_axis, ellipse_angle, ellipse_eccentricity, color
Morphometric analysesElliptical Fourier and Pixel-Based PCANANoNALandmarks
Camera and color calibrationYesNANAYesAvailable functions
Segmentation typeDeep LearningThresholdingThresholdingThresholdingThresholding, Naive Bayes, Edge detection,k-means
Contact between elements solutionSAHI and WatershedNANAWatershedWatershed
Workflow IntegrationYesYes, but without calibration, morphometrics.Yes, but without

calibration, morphometrics.Yes, without morphometricsIndependent workflows, integrated in some cases.

Regarding the key points: (1) our workflow is open source, enabling customization and further development for future studies and applications, as already demonstrated in the manuscript with other fruits; (2) it can run on any operating system with Python installed; (3) together with PlantCV, it is the only one among those mentioned written in Python—a language more widely used than MATLAB, with greater availability of learning resources, generally easier to adopt, and therefore more accessible for a broader range of breeding programs; (4) it can also run online using free and widely adopted resources such as Google Colab; (5) not all of the mentioned tools can analyze color; (6) not all provide the complete set of morphological measurements offered here; (7) none integrate both elliptical Fourier analyses and pixel-based PCA for morphometric analyses; (8) AlmondCV includes functions for camera and color calibration, which are not available in all of them; (9) for segmentation, AlmondCV introduces a novel approach not present in the others, using deep learning models and allowing easy development of a custom segmentation model, whereas the rest mainly rely on thresholding, which, as noted in the SeedExtractor paper and confirmed by our experience, often requires manual parameter adjustment: “Due to a wide range of variation in seed size and color, it is difficult to automatically set optimal size and color ranges (for all the color spaces),” a limitation that can be critical in breeding programs with high phenotypic diversity; additionally, the SeedExtractor paper states (10): “The application requires that the seeds are not touching each other when imaged,” an issue we also encountered and addressed through a watershed algorithm similar to GrainScan (but here open source) and a novel method using SAHI that separates elements in their respective functions; and (10) our workflow integrates multiple components—camera calibration, image segmentation, and morphological/morphometric trait extraction—that are not fully combined in any of the other tools mentioned.
This has been explained in the introduction to provide clarity for readers
The term ‘breakthrough’ refers to the phenotyping bottleneck in almond breeding, which our study surpasses relative to previous research and current breeding practices. We have, however, reformulated the sentence to improve clarity

2. Within horticultural crops, workflows and studies (not acknowledged in this article) are available that can be adapted or modified to do the same thing. For example - publications from as early as 2020 used machine learning models to measure size and mass of almonds, however, this relevant study was not acknowledged by the authors https://onlinelibrary.wiley.com/doi/full/10.1111/jfpe.13374 . Discuss how the presented method is better than the aforementioned article, justify the claim 'breakthrough'.

Response: Thank you for your comments. Regarding the use of the term ‘breakthrough,’ as mentioned earlier, we refer to the phenotyping bottleneck in almond breeding, which is justified by comparison with previous studies and current phenotyping practices in almond breeding programs. With respect to the advantages over the mentioned article, many of the points noted in our previous comment also apply (e.g., thresholding instead of deep learning, lack of open-source availability, unspecified programming language, unknown compatibility with different operating systems, measurement limited to length, and no calibration procedures). Moreover, that study analyzed only a single variety (NonPareil), whereas our workflow phenotypes more than 600 genotypes, capturing much greater diversity and providing a broader test. In addition, the previous study focuses primarily on industrial applications, whereas our approach emphasizes the accuracy and diversity required in breeding programs. We have included this context in the introduction to highlight the use of imaging in almonds.

3. The authors claim successfully testing the pipeline for apples and strawberries. Information for fruit size can be extracted from 2D images; however, the example results show only length, width, circularity, and ellipse ratio. How do these parameters assist fruit breeders? Since it is segmentation based classification using a reference scale, the aforementioned tools particularly SeedExtractor can generate similar results. Does it qualify the tool for integration into fruit crops breeding pipeline? Moreover, fruit breeders require on-tree analysis, recent advancements have enabled 3D sensing for

significantly better detection particularly using cost effective RGB-D cameras.

Response: Thank you for your comments. In this study, we only test the workflow on the specified species, and the traits measured are all described in the methodology section for the general method (length, width, area, perimeter, hull area, solidity, aspect ratio, circularity, ellipse ratio, and colour using the Lab* model). Additionally, when fruits are placed in an ordered and flat orientation, the same traits as for almonds can be measured (e.g., width at three different heights: 25%, 50%, and 75% of the length, vertical and horizontal symmetry). In the manuscript, we propose that our workflow could be expanded to other fruits and allow the development of crop-specific traits using the existing functions as a base/template, as illustrated with simple examples in apple and strawberry.
In addition, almond shape has been traditionally evaluated subjectively and is time-consuming (Martinez-Garcia et al 2019). By definition, however, morphological traits are highly dimensional (Zingaretii et al 2021). Computing only linear, univariate phenotype leads to a loss of information by extremely simplifying the features of a shape (Klingenberg 2010, Claes 2014). The use of geometric-morphometric approaches for shape analysis is warranted (Dryden 1998). From a breeding point of view, our advancement in high-throughput phenotyping can generate more precise phenotypic datasets that provide valuable information for understanding the genetic basis of our traits, can help to a better estimation of heritability and to improve predictive accuracy in strategies such as Genomic Selection. The integration of cutting-edge technologies like GS and HTP has the potential to accelerate gains in breeding.
Regarding differences with SeedExtractor, as mentioned in our first comment, SeedExtractor does not allow, for instance, the extraction of morphometric traits via elliptical Fourier analyses or pixel-based PCA. As noted in the introduction, these approaches have been applied in apple and strawberry breeding for target traits, providing examples of how such traits can assist fruit breeders (Feldmann et al., 2020; Keller et al., 2024).
Regarding 3D fruit phenotyping in the field, we agree that it represents a promising area for breeding. However, it typically requires more hardware and software resources, which can complicate implementation and limit adoption by breeding programs. A clear example is Li et al. (2025), who developed a sophisticated autonomous rover to phenotype traits such as fruit number, maturity, and yield in blueberry. Moreover, field phenotyping may not be equally effective for all species; for instance, almonds present additional challenges due to their shells, tree architecture, and the dispersed bearing of fruits, which is more complex than in blueberry bushes. Additionally, measurement accuracy for morphological fruit traits would generally be lower in the field than under controlled conditions, which can be critical for breeding schemes.

Minor Comments -
1. Change title - This study uses deep learning models which are a type of AI. AI is a broader term. Additionally, the potential for utility in fruit breeding pipeline appears to be limited. Suggested - Open RGB imaging workflow for morphological and morphometric analysis of almond kernels using deep learning.
Response: Thank you for your comments. We agree that “deep learning” is more precise than the broader term “AI.” However, we respectfully disagree with the assessment that the workflow has limited utility in fruit breeding pipelines. Our workflow provides tools that are relevant to breeding programs, as demonstrated in species such as strawberry and apple (Feldmann et al., 2020; Keller et al., 2024), while also incorporating new advances and features that enhance phenotyping capabilities.
2. The video tutorial showed using YOLOv8, mention it in the methods. Add information for all settings used in CVAT.
Response: Thank you for your comments. We have included it in the “Develop your segmentation model” section.

Reviewer #2: We would like to thank you for submitting your manuscript to our journal.The manuscript proposes an AI-powered open RGB imaging workflow for morphological and morphometric analysis of fruits or other plant organs, with almonds as a case study. The workflow, developed in Python, covers the full pipeline from image pre-processing, segmentation model development and deployment, to trait measurement and analysis. It aims to improve the efficiency and accuracy of

Powered by Editorial Manager® and ProduXion Manager® from Aries Systems Corporation

phenotyping in breeding programs by addressing the limitations of traditional methods, such as their time-consuming and labor-intensive nature.
Response: Thank you for your comments.

However, there are still the following problems in this paper that need further improvement:

1.Table formatting: Some of the tables in the manuscript do not follow the formatting standards of the journal. The authors are encouraged to revise them accordingly to ensure clarity, consistency, and ease of understanding.
Response: Thank you for your comments. We have followed the table preparation guidelines described in the Instructions to Authors (https://academic.oup.com/gigascience/pages/instructions_to_authors#Preparing%20Tables), including the requirement that all tables are introduced in the text, properly formatted, and have titles with fewer than 15 words. However, we are not certain which specific formatting aspect may not meet the journal's standards. We would greatly appreciate further clarification so we can make the necessary adjustments to ensure full compliance.

2.Formula presentation: Certain mathematical formulas are not clearly formatted and appear disorganized. The authors should re-typeset the equations to improve readability and provide clearer explanations for each formula.
Response: Thank you for your comments. In our original Word document, the formulas appear correctly formatted. We suspect that the conversion process during submission may have altered their layout. We will recheck the typesetting in the submitted version and re-typeset the equations to ensure they are properly aligned and readable.

3.Introduction: The introduction could be strengthened by more thoroughly explaining the relationship between phenotypic data and breeding. The authors may also discuss how phenotyping data supports genomic selection and accelerates breeding via high-throughput workflows.
Response: Thank you for your comments. We have reinforced the introduction to clarify the importance of phenotyping in breeding and its impact in genomic selection.
4.Methods section: While the paper clearly explains how morphological traits and kernel thickness are measured, it does not sufficiently explain how this data contributes to breeding decisions. The authors should elaborate on how the extracted traits are applied in practical breeding or selection strategies.
Response: Thank you for your comments. We have reinforced the discussion to clarify the importance how can be incorporated in breeding approaches.

5.Lack of algorithmic novelty: While the integration of existing tools is commendable, the core methods used (e.g., YOLO, SAHI) are based on publicly available models, without introducing new algorithmic components or comparative ablation studies. The authors are advised to clarify the unique contribution of their workflow, especially in terms of engineering integration or practical usability.
Response: Thank you for your comments. We acknowledge that the core methods employed are publicly available, which we also consider a strength of our work due to the use of pre-trained generalist models (Bommasani et al., 2021). Furthermore, we implemented specific adaptations in the SAHI method, as indicated in the Develop your segmentation model section of the methodology, to enable the use of the retina_mask argument, which improves fine-detail accuracy in segmentation. In any case, we have clarified our contribution in terms of integration and practical usability in the manuscript, specifically at the end of the introduction.
Bommasani R, Hudson DA, Adeli E, Altman R, Arora S, Arx S von, et al.2021. On the Opportunities and Risks of Foundation Models. arXiv;

6.Limited evaluation metrics: The performance of segmentation models is only reported using error percentage. The inclusion of standard metrics such as IoU, Precision, Recall, and F1-score would allow for a more comprehensive evaluation and comparison across models (see LLRL methods).
Response: Thank you for your comments. We agree, and indeed these metrics are presented in Supplementary Figure 2, although they were not discussed in detail in the original manuscript. We have now included a more detailed discussion of these results in the Results section ("YOLO fine-tuned segmentation models performance and

Powered by Editorial Manager® and ProduXion Manager® from Aries Systems Corporation

reconstruction errors”).

7.Figures and captions: Currently, figure images and their descriptions are placed separately, which may reduce readability. It is recommended to place figure captions immediately beneath or alongside the figures to enhance the paper's coherence and user-friendliness.
Response: Thank you for your comments. We agree with your observation; however, according to the journal’s guidelines, figure files must be submitted separately and not embedded within the manuscript.
https://academic.oup.com/gigascience/pages/instructions_to_authors#Preparing%20Figures

8.Trait extension suggestions: In order to enhance the expressiveness and resolution of phenotypic trait modeling, authors are advised to refer to the relevant research on extracting fine-grained phenotypic features in plant images in recent years. For example, PlanText proposed a progressive visual guidance strategy to help improve the modeling quality of phenotypic traits in images.
Response: Thank you for your comments. We were not aware of this visual guidance strategy, but we agree that it could be particularly useful for the abstract traits discussed in our manuscript. We have now included a brief mention of this possibility in the discussion.

Therefore, I would like to give a "Major Revision" recommendation.

Reviewer #3: The present study entitled "Open RGB Imaging Workflow for Morphological and Morphometric Analysis of Fruits
using AI: A Case Study on Almonds" reported the development of a Python-based image analysis pipeline that extract morphological traits of almond nut shells and kernels. A case study was conducted to use the developed pipeline to analyze breeding populations of 665 genotypes and extract both general morphology traits such as height, length, area, aspect ratio, etc. and specialized traits for almond such as width at three heights, vertical and horizontal symmetry, etc. Further, each nut shell or kernel was weighed, so models were established to use the weight and morphological traits to predict the thickness of each nut shell or kernel. In addition to morphological traits, morphometric (or shape) was extracted for each nut shell or kernel. Clustering analysis was performed on the morphometric traits to identify variability among genotypes. To further validate the efficacy of the extracted traits, broad-sense heritability was calculated and used as a criterion.

The major contribution of this study is the integration of different components (e.g., camera calibration, image segmentation, and morphological/morphometric trait extraction, etc.) as a user-accessible, open-source Python implementation for the plant breeding community, especially for almond breeders. However, there several aspects that could be further improved.
Response: Thank you for your comments.

First, the present study showed the most number of samples that were phenotyped by the proposed pipeline among recent efforts on almond nut shell and/or kernel phenotyping. However, there was no clear evidence to demonstrate direct benefits to ongoing almond breeding. Certain traits (e.g., aspect ratio, tip/top/side curvatures) could be included in a breeding program, but what's the significance of including these traits in breeding programs. Are they crucial to either improve the productivity, quality, or other management practices or processing practices for the almond industry, especially given breeding context?
Response: Thank you for your comments. We agree that we should provide more information on the benefits of these traits. For this reason, we have added a discussion on this topic in the Introduction.
“These traits directly influence market value and consumer acceptance [25,26]. Larger kernel sizes are generally more economically valuable [24], while smaller sizes are preferred for specific applications, such as chocolate bars [22]. Shape is also important for industrial processes: elongated almonds are preferred for slicing, whereas round almonds are generally more appreciated by consumers [22,23][23]. Geometric parameters also affect the mechanical properties of almonds and other nuts, which is

Powered by Editorial Manager® and ProduXion Manager® from Aries Systems Corporation

relevant for processing [27,28]. For example, elongated shapes have been shown to result in lower breakage during blanching, while rounder shapes reduce breakage during shelling in Spanish almond industry machinery [29]."
We have also highlighted in the Discussion the concerns regarding breakage during industrial processing and the role that almond nut and kernel shape can play in this context.

Second, the pipeline uses deep learning-based segmentation which is powerful to handle complex background. Based on the limited figures or example images in the GitHub repo, the background is mostly single colored (e.g., white or black) without appearances that may confuse even conventional segmentation, especially if image color is calibrated. Assuming most of the almond nut shell and kernel analyses would be done in a laboratory condition, it is not convincing why conventional segmentation methods may not be preferred if both illumination and camera configuration can be well controlled. Ultimately, the question is whether it is worthy the effort of labeling hundreds of images to fine-tune a deep learning segmentation model compared to a careful hardware-software design to make operation more efficient. Or with the simplified background, vision foundation models such as SAM will be sufficient.
Response: Thank you for your comments. We have carefully considered these aspects. While we initially explored conventional segmentation methods, our experience indicates that deep learning-based segmentation models offer clear advantages for a high-throughput phenotyping system:
1.We observed that due to the large phenotypic diversity in almonds—including variations in color, shape, texture, and size—thresholding techniques and similar approaches often require manual adjustments. We believe this issue is likely to occur in breeding programs of other species with high phenotypic diversity. Such manual interventions hinder automation, whether processing images in real time or during post-processing. Indeed, similar limitations have been reported in other software, such as SeedExtractor: "Due to a wide range of variation in seed size and color, it is difficult to automatically set optimal size and color ranges (for all the color spaces)."
2.Another aspect highlighted in SeedExtractor, which we also observed when using conventional segmentation, is that "The application requires that the seeds are not touching each other when imaged." This challenge has been addressed in software such as GrainScan, which implements a watershed algorithm (although it is not open-source). In our work, we applied an open-source watershed approach, and additionally employed another method using SAHI, which effectively separates individual elements for downstream analysis.
3.Additionally, we captured images of multiple groups of almonds placed inside painted rectangles (see Methodology), for which we fine-tuned simple deep learning models to recognize and separate the groups. Using conventional segmentation, we frequently encountered problems when almonds touched the rectangle boundaries or when the contours of the rectangles were not correctly enclosed during processing.
4.With fine-tuned deep learning models, we can relax the strict requirements on hardware and software design, which becomes particularly important when working with the large sample sizes as here. In such scenarios, processing speed can be just as critical as precision during image acquisition for the overall feasibility of the process.
5.For phenotyping processes, minimizing manual adjustments facilitates the adoption of the task by technicians without a computer vision background, which also helps to accelerate the overall workflow.
In response to whether it is worthwhile to label hundreds of images to fine-tune a deep learning segmentation model, we argue that it is. The labeling process has become significantly faster, taking approximately 30 minutes to annotate all images using a combination of CVAT and SAM, as demonstrated here. Regarding the direct use of foundation models, Bommasani et al. (2021) note that foundation models "are intermediary assets; they are unfinished and generally should not be used directly, instead requiring adaptation for specific downstream tasks." Given that fine-tuning is not time- or cost-intensive, it improves segmentation performance, accuracy, and consistency, while reducing the need for manual supervision.
Bommasani R, Hudson DA, Adeli E, Altman R, Arora S, Arx S von, et al.2021. On the Opportunities and Risks of Foundation Models. arXiv;

SeedExtractor https://www.frontiersin.org/journals/plant-science/articles/10.3389/fpls.2020.581546/full#h3

Powered by Editorial Manager® and ProduXion Manager® from Aries Systems Corporation

	GrainScan https://link.springer.com/article/10.1186/1746-4811-10-23<br><br>Third, in the Introduction section, some technical statements should be revised to make them accurate. For example, image segmentation is a core computer vision task rather than relying on computer vision algorithms. One-stage and two-stage strategies are used to differentiate models for object detection not image segmentation. Further, Faster RCNN is an object detection model and cannot do image segmentation. It is highly recommended that the authors could find a computer science or engineering colleague to proofread the technical statements to ensure the accuracy.<br>Response: Thank you for your comments. We have revised the Introduction to improve technical accuracy. Regarding the use of "one-stage" and "two-stage" terminology in instance segmentation, we acknowledge that these terms are more commonly associated with object detection. However, we have retained them in our manuscript because they are used in recent literature on instance segmentation (e.g., Bolya et al., 2019; Gu et al., 2022) to describe architectural strategies. We are open to updating the terminology if more precise references or conventions are suggested.<br>Bolya, D., Zhou, C., Xiao, F., & Lee, Y. J. (2019). Yolact: Real-time instance segmentation. In Proceedings of the IEEE/CVF international conference on computer vision (pp. 9157-9166).<br>Gu, W., Bai, S., & Kong, L. (2022). A review on 2D instance segmentation based on deep neural networks. Image and Vision Computing, 120, 104401.<br><br>Last, it is appreciated the authors effort on making an open-source software for the community. However, the dataset can be equally important to advance the scientific discovery and technology development. Is there any plan to make the dataset publicly available to help facilitate the development of additional computer vision algorithms for almond phenotyping?<br>Response: Thank you for your comments. The dataset was submitted alongside the manuscript to GigaScience and has also been deposited in Zenodo. It is currently available to reviewers (see Data Availability section) and will be made publicly accessible to the scientific community upon publication.<br><br>Please also take a moment to check our website at https://www.editorialmanager.com/giga/l.asp?i=218825&l=T02XNJYZ for any additional comments that were saved as attachments. Please note that as GigaScience has a policy of open peer review, you will be able to see the names of the reviewers.
**Additional Information:**	
**Question**	**Response**
Are you submitting this manuscript to a special series or article collection?	No
**Experimental design and statistics**<br><br>Full details of the experimental design and statistical methods used should be given in the Methods section, as detailed in our Minimum Standards Reporting Checklist. Information essential to interpreting the data presented should be made available in the figure legends.<br><br>Have you included all the information requested in your manuscript?	Yes

**Resources**<br><br>A description of all resources used, including antibodies, cell lines, animals and software tools, with enough information to allow them to be uniquely identified, should be included in the Methods section. Authors are strongly encouraged to cite Research Resource Identifiers (RRIDs) for antibodies, model organisms and tools, where possible.<br><br>Have you included the information requested as detailed in our Minimum Standards Reporting Checklist?	Yes
**Availability of data and materials**<br><br>All datasets and code on which the conclusions of the paper rely must be either included in your submission or deposited in publicly available repositories (where available and ethically appropriate), referencing such data using a unique identifier in the references and in the “Availability of Data and Materials” section of your manuscript.<br><br>Have you have met the above requirement as detailed in our Minimum Standards Reporting Checklist?	Yes
GigaScience has policies and guidelines in place for the use of generative AI-writing tools such as ChatGPT. If you have used such writing tools to assist with writing the manuscript this must be declared and cited in the text. Authors should not list AI-writing tools and other AI-assisted technologies as an author or co-author and should acknowledge that they are fully responsible for text generated or refined by AI-writing tools.<p><br><br>A summary of use (particularly in the introduction or among methods) needs to be included at the end of the paper, and the outputs should also be included as a supplementary file hosted in GigaDB or other open repositories. Please <a	No

href=https://academic.oup.com/gigascience/pages/editorial_policies_and_reporting_standards target="_new" > read our guidelines for more information. <p><br><br>By submitting to GigaScience, you are aware of the journal's AI-writing tools policy, and if you have declared use of such tools below, you have acknowledged this where appropriate in your manuscript and have made a summary of use and outputs available. <p><br><b>AI-assisted writing tools have been used in the preparation of this manuscript?	

Powered by Editorial Manager® and ProduXion Manager® from Aries Systems Corporation

Manuscript_clean_version Click here to access/download;Manuscript;renamed_8580a.docx

Open RGB Imaging Workflow for Morphological and Morphometric Analysis of Fruits using Deep Learning: A Case Study on Almonds.

Jorge Mas-Gómez [0000-0002-3663-6743]

Manuel Rubio [0000-0003-4605-8227]

Federico Dicenta [0000-0002-7647-5235]

Pedro José Martínez-García* [0000-0002-7647-5235]

Fruit Breeding Group. Department of Plant Breeding, Centro de Edafología y Biología Aplicada del Segura- Spanish National Research Council (CEBAS-CSIC). Campus Universitario Espinardo, E-30100 Murcia, Spain

*Corresponding author: pjmgarcia@cebas.csic.es

Abstract

Background

High-throughput phenotyping is addressing the current bottleneck in phenotyping within breeding programs. Imaging tools are becoming the primary resource for improving the efficiency of phenotyping processes and providing large datasets for genomic selection approaches. The advent of AI brings new advantages by enhancing phenotyping methods using imaging, making them more accessible to breeding programs. In this context, we have developed an open Python workflow for analyzing morphology, colour and morphometric traits using AI, which can be applied to fruits and other plant organs.

Results

The workflow was implemented in almond *(Prunus dulcis* (Mill.) D. A. Webb), a species where breeding efficiency is critical due to its long breeding cycle. Over 25,000 kernels, more than 20,000 nuts, and over 600 individuals were phenotyped, making this the largest morphological study conducted in almond so far. The best segmentation and reconstruction approaches achieved error rates below 1%. Weight and area variables enabled accurate estimation of kernel thickness, with a root mean squared error (RMSE) of 0.47. Fifty-five heritable morphological, morphometric and colour traits were identified, highlighting their potential as target traits in breeding programs.

Conclusion

The proposed workflow demonstrated robust performance across diverse datasets and being effective with limited training data for fine-tuning. Its compatibility with the output of AI-based labelling tools allows users to fully leverage the advantages of these technologies—reducing manual effort, accelerating dataset preparation, and streamlining

the fine-tuning process of segmentation models. This flexibility enhances the scalability and practical applicability of the workflow in real-world phenotyping scenarios, especially in the context of breeding programs.

Keywords

High-throughput Phenotyping, Almonds, Breeding, Computer Vision, Deep learning

Background

An observable performance of a plant (resulting from its genotype, environment, and their interaction) is defined as the phenotype. Phenotypes allow the portion attributable to genetic effects to be identified through quantitative genetics. The process of measuring and describing plant phenotypes is called phenotyping, and it remains the primary bottleneck in breeding programs [1]. To overcome this bottleneck, classical phenotyping tasks, which are often tedious, time-consuming, subjective and/or expensive, are being replaced. Breeders are leveraging tools such as computer vision [2], artificial intelligence [3], and unmanned aerial platforms [4], among others, to meet the need for large-scale phenotyping and the generation of accurate, accessible big datasets [1]. Such high-throughput phenotyping enables to phenotype larger populations and the generation of quantitative measurements, which can improve prediction accuracy and response to selection in Genomic Selection (GS) schemes [5–8]. Clearly, high-throughput phenotyping is becoming a valuable tool for plant breeders and researchers to meet the challenges of future food demand [9,10]. The use of high-throughput phenotyping platforms for fruit species such as apple, mango, vineyard or citrus have helped to study architecture parameters, pigment and nutrient contents, water stress, biochemical parameters of fruits and disease detection [11]. A device for fast and accurate phenotyping of small fruit samples called "FruitPhenoBox" was developed for breeding programs [12], leading to the identification of key marker-trait associations in apple populations [13]. Additionally, by integrating genotype data, RGB imaging, and autoencoders, a framework was developed to reconstruct apple samples using only genotype data [14]. Morphometric approaches using machine learning have been applied in strawberries to extract quantitative shape features, enabling the identification of new heritable traits [15]. Furthermore, an automatic pipeline to phenotype morphological traits was developed for

strawberries using computer vision techniques [16] and 3D imaging [17], with applicability to other fruits.

For stone fruit trees species, such as almond (*Prunus dulcis* (Mill.) D. A. Webb) the implementation of these tools is still scarce. Breeding almond trees, and other *Prunus* species such as apricot, peach, plum or cherry is a long and expensive task. Due to their long juvenility period the timing to obtain a new improved cultivar is around 12-15 years. Therefore, increasing efficiency is a key aspect for breeding these species, particularly in phenotyping processes of traits of interest such as fruit morphology, shape and colour [18–20]. These traits directly influence market value and consumer acceptance [21,22] and as have been commented, these traits are highly dimensional [16] and computing only linear, univariate phenotype leads to a loss of information by extremely simplifying the features of a trait [23,24], requiring the use of geometric-morphometric approaches [25].

Larger kernel sizes are generally more economically valuable [20], while smaller sizes are preferred for specific applications, such as chocolate bars [18]. Shape is also important for industrial processes: elongated almonds are preferred for slicing, whereas round almonds are generally more appreciated by consumers [18,19]. Geometric parameters also affect the mechanical properties of almonds and other nuts, which is relevant for processing [26,27]. For example, elongated shapes have been shown to result in lower breakage during blanching, while rounder shapes reduce breakage during shelling in Spanish almond industry machinery [28]. Preliminary efforts in imaging analysis and deep learning in almond have demonstrated that the same quantitative trait loci (QTLs) for size and shape can be successfully identified using these approaches as with traditional manual phenotyping methods [29]. Imaging has also been applied in “Nonpareil” almond cultivar, to measure length and predict kernel mass using machine learning, for industrial applications [30]. Moreover, 3D imaging phenotyping methods have been developed for

almonds nuts. Software for measuring seed morphology has been developed, enabling the quantification of both shape and color traits [31–33]. However, none of these tools allow for detailed morphometric analyses of kernel shape, which can be an important commercial trait in almonds [21]. A common challenge is that elements (such as seeds or fruits) often touch each other [31] and algorithms such as watershed segmentation are not implemented in all available software. Among these tools, only SeedExtractor is open-source, providing a foundation for customization and further development, although it is implemented in Matlab [31]. In contrast, widely adopted programming languages can facilitate faster and broader adoption. In this regard, PlantCV, written in Python, offers numerous tutorials for learning computer vision in plant phenotyping [34].

In most of the mentioned studies, segmentation — defined as the process of partitioning a digital image into meaningful regions of interest (ROIs) based on homogeneous visual features such as colour, texture, or intensity — is a core computer vision task, which its conventional use through binary thresholding often require manual adjustments, limiting automation—especially as scene complexity increases [35,36]. To enhance efficiency and automation, advanced tools capable of handling this complexity are essential for fruit morphology studies [37]. In this sense, deep learning techniques have gained significant relevance due to their high segmentation accuracy, even in complex scenarios, by leveraging large training and validation datasets [38]. Instance segmentation using deep learning can be implemented through two main approaches: two-stage models (e.g., Mask R-CNN) and one-stage models (e.g., *You look only once* (YOLO), RetinaNet) [39]. While two-stage models offer higher accuracy at the cost of lower speed and greater computational requirements, one-stage models aim to balance accuracy and speed while using fewer resources [39]. Notably, recent advancements in YOLO models have significantly improved their accuracy while maintaining high-speed performance [36].

Nevertheless, training deep learning models from scratch requires large labelled datasets and substantial computational resources [40]. In this context, fine-tuning pre-trained generalist models allows users to develop customized models without the need for extensive datasets [40,41]. The integration of deep learning tools for labelling datasets has significantly accelerated this task [41,42]. Moreover, new techniques such as Slicing Aided Hyper Inference (SAHI) have enhanced accuracy by reducing errors in the segmentation of small objects, making it particularly valuable for seed imagery [43]. The slicing process also reduces memory requirements for processing large images, maintaining high resolution without the need to resize them for model training, and enabling the utilization of limited GPU (Graphics Processing Unit) open resources (e.g., Google Colab).

In this work, we have developed a Python-based workflow to facilitate the development and deployment of custom deep learning models for measuring morphological and morphometric traits in fruit breeding programs. The main contribution of the workflow is the integration of pre-processing methods such as colour and distortion correction, development and deployment of image segmentation fine-tuned generalist models, morphology measurements, and morphometric analyses. The workflow was successfully applied to phenotype multiple almond breeding populations, allowing for the estimation of broad-sense heritability values. Additionally, prediction models were developed to estimate kernel thickness based on area and weight. By implementing this workflow, the study has analyzed the largest number of individuals and data points ever recorded for the almond species to date.

Material and Methods

Plant material

The workflow was implemented to study breeding populations of the CEBAS-CSIC almond breeding program located in the experimental field (Santomera, Murcia SE of Spain) during 2022 and 2023 seasons. 665 unique genotypes from six F1 populations and a germplasm collection were studied (Table 1). Population parents were traditional cultivars (‘Marcona’ and ‘Desmayo Largueta’), released cultivars of CEBAS-CSIC (‘Antoñeta’, ‘Penta’, ‘Tardona’, and ‘Florida’) and the breeding selections ‘R1000’ (INRA, France) and ‘S4017’ (CEBAS-CSIC). The target samples were shells and kernels of these almond descendants. For each genotype, 30 in-shell nuts and 30 kernels per year were sampled, photographed, and weighed, whenever available.

Table 1. The populations used in this study and the number of individuals studied per year

Year	2022		2023		Unique genotypes	
**Family**	**Shell**	**Kernel**	**Shell**	**Kernel**	**Shell**	**Kernel**
Germplasm collection	85	85	74	91	99	99
Antoñeta × Marcona	6	6	19	19	19	19
Antoñeta × Penta	161	161	142	142	183	183
Antoñeta × Tardona	57	57	56	53	71	70
Florida × Marcona	17	17	43	43	44	44
Desmayo × R1000	198	198	187	194	223	223
Marcona × S4017	0	0	26	27	26	27

Total	524	524	547	569	665	665

Workflow description

A comprehensive workflow has been developed for pre-processing, segmentation model development, deployment, morphological measurements, and morphometric analyses, implemented through interactive Python notebooks (Supplementary Figure 1) (https://github.com/jorgemasgomez/almondcv2). To optimize speed and performance, the use of a GPU is highly recommended. The workflow can be executed locally; however, it is also deployed on the Google Colab platform, enabling users to run it online and leverage free resources, such as GPUs. Although primarily written in Python, some functions integrate R for morphometric analyses, requiring R to be properly configured in the system's path if executed locally.

Colour and distortion calibration

Colour and distortion correction approaches are included in the pre-processing notebook (*1_Pre-processing_workflow.ipynb*). Colour correction enables to standardize the dataset of the images using a colour card as reference. Colour correction functions implemented in the pre-processing notebook are based on the colour correction module of PlantCV python library [34]. Distortion correction is implemented for possible radial and tangential distortion caused by the camera. Distortion correction functions are based on the OpenCV workflow for camera calibration [44]. It requires test patterns of chessboards with a known size of the squares to obtain a camera matrix that can be used subsequently with the pictures of the dataset. In addition, colour and distortion correction functions are joined in the pre-processing notebook to deploy it in the whole dataset in one step.

Auxiliary functions

Some optional auxiliary functions were included for the picture pre-processing, helping to separate the different samples in the pictures and getting the physical size of a pixel with reference objects in the picture (e.g. a coin with known diameter). These optional functions require training a model (next sections) to identify and segment the sample groups and the reference objects.

Develop your segmentation model

The approach implemented in the present work intends to simplify the development of customized segmentation models. Four key steps are necessary for performing the development: slicing, labelling, 'training' and reconstruction (*2_Develop_your_segmentation_model_workflow.ipynb*). The slicing process consists of cropping pictures into smaller patches, with the size determined by the user. Using smaller patches of high-resolution images speeds up the training and prediction processes by reducing computational requirements [43]. Additionally, this approach improves the capture of fine details, as it avoids the loss of resolution and distortion caused by resizing the entire image [45,46]. The slicing function defined in the workflow is divided into train, validation, and test datasets. The slices are obtained proportionally according to the user command to prepare for the next step. The second step, labelling, involves annotating which pixels correspond to the object of interest. For this task, the workflow was designed to accept as input the ZIP file generated by the image annotation platform CVAT in the YOLO Segmentation 1.0 format [42]. CVAT provides tools for semi-automatic segmentation using the Segment Anything Model (SAM) [41], which enables quick instance segmentation with a single click. CVAT is available for free offline via a Docker container or online with a freemium model. The third step for developing the segmentation model is 'training' the model. For that purpose, in the second notebook (*2_Develop_your_segmentation_model_workflow.ipynb*) YOLO [47] pre-trained

algorithm series can be fine-tuned (although in their web mention 'training' really is a fine-tuning [48]) using our custom labelled dataset. The training process function enables to control all the parameters of the training process (e.g. epochs, batch) and provides the results of the training *YOLO.train* method. The last step for developing the segmentation model is to reconstruct the binary picture mask, for which two approaches were used. The first approach simply joins the patches binary masks after the prediction (*slice_predict_reconstruct*). The contours are detected using *cv2.findcontours* OpenCV function [49] in the subsequent functions for morphology measurements. Watershed algorithm is implemented in such functions for separating touching objects of interest [50]. The second approach uses the SAHI pipeline (*predict_model_sahi*) [43], which is integrated in the YOLO segmentation process, and the instances segmented are identified specifically providing their contours directly, solving also the problem of touching objects of interest. Here, SAHI python library is modified slightly to enable the *retina_mask* argument in YOLO prediction function and provide fine details in the segmentations. A video tutorial describing the development of the model can be found in the workflow's GitHub repository: https://github.com/jorgemasgomez/almondcv2. To assess the two reconstruction approaches, they were tested in the datasets studied and the almonds with errors in the reconstruction were annotated and removed manually.

Deploy your segmentation model for morphology and morphometric analyses

Once the segmentation model has been successfully 'trained' and tested, it can be deployed for morphology and morphometric analyses. In the notebook for deployment (*3_Deploy_your_segmentation_model_worflow.ipynb*), two methods were prepared to measure.

The first method is general for any group of fruit, and the traits length (the longest axis of the fruit), width (the dimension perpendicular to the length), area (the surface enclosed

by the fruit contour), perimeter (the length of the outer boundary), hull-area (the area of the convex hull enclosing the shape), solidity (the ratio between area and hull-area, reflecting compactness), aspect ratio (the ratio of length to width, indicating elongation), circularity (calculated as $4\pi \times$Area/Perimeter, with values closer to 1 indicating a more circular shape), ellipse ratio (the ratio between the minor and major axes of a fitted ellipse), and colour (L*a*b* model) are measured for each shell/kernel. This general method can be used as a template and customized according to the user's needs, for example to study additional traits or different fruit shapes. The second method is specific for almonds adding more traits such as width at three different heights (25, 50 and 75% of the length), vertical and horizontal symmetry and symmetry in the top part of the almond (to measure the shoulder). In this specific method, almonds are aligned according to the angle of fitting an ellipse (*cv2.fitEllipse* function) and flipped vertically with placing the most distant point always in the lowest part (almond tip). Some extra traits derived from the results together with the weight of the kernel/shell such as the weight kernel/shell ratio, estimation of the thickness (only for kernels) and globosity (width/thickness), were also included. For the estimation of kernel thickness, a linear and quadratic model was fitted using 227 almond kernels, using the area and the weight as predictor variables. To validate the models, the dataset was split into 80% for training 20% for validating, running it 100 times to assess the stability of the models.

Both methods export picture results, table results, and binary masks for morphometric analyses. Binary masks are flipped horizontally if the lowest pixel (tip) is the right part of the picture to avoid symmetric issues in morphometric analysis.

Morphometric analyses notebook (*4_Morphometrics_workflow.ipynb*) includes two morphometric approaches: Elliptical Fourier analysis (EFA) and pixel-based principal component analysis (PCA). EFA is conducted using Momocs v1.4.1. R package [51] but

it has been implemented in the notebook in Python programming language executing a subprocess. Several functions are included for EFA: exploratory analysis, running the EFA, after that perform a PCA on the EFA results and *kmeans* clustering. PCA performed on EFA coefficients generates as traits the components (EF-PCs) that explain the variability of the shape. Regarding the second pixel pixel-based PCA, binary masks are flattened for performing the PCA and generating also PCs as traits (PB-PCs)[15]. Result plots of both approaches can be obtained to show the influence of each trait.

RGB Imaging system

For image acquisition, group pictures of various samples were captured over a black surface template, with the samples positioned inside painted rectangles. For the 2022 dataset, images were taken using a Canon EOS 70D camera, achieving a resolution of 6 px/mm. In 2023, the imaging system was redefined due to objectives beyond the scope of this work, utilising an Arducam 8MP IMX219 camera, which resulted in images with a resolution of 4 px/mm. Both systems were illuminated using two white LED light sources.

Heritability

Broad sense heritability (H^2) was estimated using the Python library *statsmodels* [52], fitting a linear mixed model using the genotype as random effects and the year as fixed effects (Equations 1 and 2).

$$Y_{ijk} = \mu + G_i + Y_j + \varepsilon_{ijk} \quad (\textit{Equation 1})$$

Where:

Y_{ijk}: Observed phenotypic value for the k-th observation of the i-th genotype in the j-th year.

μ: Overall population mean.

G_i: Random effect of the i-th genotype

Y_j: Fixed effect of the j-th year

ε_{ijk}: Residual error term

$$\frac{\sigma^2 G}{\sigma^2 G + \sigma^2 e} \quad (Equation\ 2)$$

Where:

σ^2G Genetic variance.

σ^2e: Residual variance.

Impact on almond breeding

To study the impact of this new tool on almond breeding, a comprehensive comparison with existing scientific articles related to quantitative almond morphology phenotyping was conducted. Several parameters such as the number of individuals studied, number of shells/kernels phenotyped, and the number of data points obtained, were compared to evaluate the benefits of the new high-throughput phenotyping tool.

Results

YOLO fine-tuned segmentation models performance and reconstruction errors.

A total of 46,729 elements (shell and kernels) were segmented (using watershed approach for subsequent steps), and performance errors from both reconstruction approaches were annotated (Table 2). A standard image generated by the workflow is shown in Figure 1. Four segmentation models were fine-tuned using datasets from kernel and from shell in 2022 and 2023. The pre-trained model 'yolov11s-seg.pt' [53] was selected, and fine-tuning was performed over 100 epochs (i.e., complete passes through the entire training dataset), using 500–1,000 image slices of 320 pixels each, split into 60% for training, 20% for validation, and 20% for testing. Metrics plots from the fine-tuning process of the datasets are shown as an example in Supplementary Figure 2. In general, training and validation loss plots exhibited a consistent decrease in the process, with no evidence of overfitting. Precision and recall exceeded 0.9 early in training and remained stable thereafter. The Mean Average Precision (mAP) calculated at an intersection over union (IoU) threshold of 0.50 (mAP@50) reached ~1.0 values, while the more stringent, at varying IoU thresholds, ranging from 0.50 to 0.95, (mAP@50-95) stabilized later in training but consistently remained above 0.9. Validation masks were explored, showing precise results. All the combinations (dataset + reconstruction approach) showed errors lower than 2% except for the shell 2022 dataset using SAHI (2.93%) (errors were considered when an almond was incorrectly reconstructed, not detected, or if it was something other than a target sample).

Figure 1. Images generated by the workflow, displayed from left to right: original image with masks, length and width measurements, widths at different lengths, circularity, and ellipse ratio.

Table 2. Performance for different datasets and reconstruction methods, including reconstruction errors.

Method	Dataset	Errors	Total elements	Total phenotyped elements	Error percentage
Slice_predict_reconstruct + Watershed	Kernel-2022	5	10,184	10,180	0.05%
	Kernel-2023	264	15,568	15,304	1.70%
	Shell-2022	4	10,364	10,360	0.04%
	Shell-2023	60	10,945	10,885	0.55%
SAHI	Kernel-2022	14	10,184	10,178	0.14%
	Kernel-2023	139	15,568	15,429	0.89%
	Shell-2022	304	10,364	10,288	2.93%
	Shell-2023	44	10,945	10,902	0.40%

Thickness estimation

Kernel thickness was modelled using weight and area as predictor variables, employing both linear and quadratic models. The median R^2 in the test dataset for the linear model was 0.725, while for the quadratic model it was 0.795. The corresponding median Root Mean Squared Error (RMSE) values were 0.56 for the linear model and 0.47 for the quadratic model (Figure 2A). The best-performing linear and quadratic models were then plotted, achieving R^2 values of 0.847 and 0.8815, respectively, and RMSE values of 0.451 and 0.381, respectively (Figure 2B). The quadratic model trained with the complete dataset was employed for estimating thickness across all samples in subsequent analysis.

Figure 2. Results of the kernel thickness modelling, showing the R^2 (A, left) and RMSE (A, right) over 100 iterations, along with scatter plots for the best linear (B, left) and quadratic (B, right) models.

Morphometric results

Elliptical Fourier analysis (EFA) was conducted using all the kernel and shell binary masks (separately), placing 10 harmonics in both datasets after a visual exploration analysis. PCA was performed on the EFA coefficients, being 62.63%, 19.93%, and 3.83% explained by the three first PCs for the kernel analysis, and 69.19%, 15.75%, and 4.15% explained by the three first PCs for the shell analysis (Figure 3 and Supplementary Figure 3). Only the first 6 components on each dataset were used for subsequent analysis because they explained at least 1% of the variability. K-means clustering from k=1 to k=10 was run to show the main groups of the datasets (Figure 4 and Supplementary Figure 4. The optimal number of clusters was studied and no abrupt changes in the slope by elbow method were observed in any dataset (Supplementary Figure 5).

Figure 3. EFA-PCA scatterplot results for the kernel A) EF-PC1/EF-PC2 and B) EF-PC1/EF-PC3 and shell C) EF-PC1/EF-PC2 and D) EF-PC1/EF-PC3 datasets. The influence of the PCs on shape is represented in the plots.

Figure 4. K-means clustering results using EFA-PCA in kernel dataset, showing the shapes corresponding to each centroid for scenarios ranging from k=1 to k = 10.

Pixel-based PCA was performed in kernel and shell datasets, obtaining 34.96%, 8.78% and 4.75% variance explained by the three first PCs in the kernel dataset and 37.72%, 9.19% and 4.96% in the shell dataset (Supplementary Figure 6). The PCs influence in the shape was studied by plotting the +-3x deviation from the mean shape (Figure 5 and Supplementary Figure 7). Ten PB-PCs were collected for each dataset, explaining at least 1% of the variance for each one of them. K-means clustering was also carried out from k=1 to k=10 (Figure 5 and Supplementary Figure 8), and no abrupt changes were observed by the elbow method to determine the optimal number of clusters (Supplementary Figure 9).

Figure 5. Example images for Shell PB-PC3 (A) and Kernel PB-PC5 (B) morphometric traits, showing two contrasting phenotypes for each trait. Panels A1 and B1 show the original almond images; A2 and B2 show the corresponding binary masks; and A3 and B3 display the pixel-based PCA traits, from the mean shape to ±3 standard deviations.

Figure 6. K-means clustering results using PB-PCA in kernel dataset, showing the shapes corresponding to each centroid for scenarios ranging from k=1 to k = 10.

Correlations

To analyse and interpret the phenotype data obtained, the Pearson correlation between all morphological and morphometric traits was calculated (Figure 7). In absolute value, 340 pairwise correlations were higher than 0.75, with the traits of shell almond area, hull area, and perimeter exhibiting the highest number of strong pairwise correlations. High correlations (greater than 0.95) were observed between kernel and shell morphometric traits across different approaches, such as EF-PC1 with PB-PC1. Additionally, strong

correlations were found between these two PC1 components and morphological traits like aspect ratio and ellipse ratio.

Figure 7. Heatmap of the Pearson correlations between all morphological and morphometric traits.

Heritability

Broad sense heritability (H^2) was estimated for all the traits studied here (Figure 8). Fifty-five out of the 73 traits showed heritabilities ≥ 0.25. Weight ratio kernel/shell, shell weight, and shell EF-PC1 showed the highest heritability values (0.9, 0.77 and 0.70 respectively). Between morphometric traits, those related with PC1 showed high heritability values (higher than 0.68), and others such as Kernel PB-PC2, Shell PB-PC2, Shell EF-PC4 and Shell PB-PC3 showed medium values (higher than 0.3).

Figure 8. Broad-sense heritability of all traits studied in the almond populations.

Impact on almond breeding

Nine scientific articles related to almond morphology and breeding were collected to compare the performance of the high-throughput approach implemented in the current study (Supplementary Table 1 and Figure 9). A total of 25,597 kernels were studied (an average of 12,799 per season), along with 21,316 shells (an average of 10,658 per season)

(Figure 9). The entire dataset comprised 660 individuals, and 1,579,443 data points were collected (Figure 9).

Figure 9. Bar plot for comparison among articles related to quantitative almond morphology traits [19,29,54–59].

Discussion

The comprehensive workflow presented here introduces a new high-throughput phenotyping tool using almonds as a case of study for fruits. This approach has enabled the analysis of 665 genotypes—the largest dataset for almond morphological quantitatively measured traits reported to date, to the best of the authors' knowledge. The development of this new phenotyping tool represents significant progress in addressing the phenotyping bottleneck in almond breeding programs. The developed methods were adjusted to the target samples used but can be adapted to phenotype other kinds of fruits and other types of organs such as leaves or roots. Furthermore, as it is open source, it can be used by other plants breeding programs. In fact, the workflow was successfully applied to publicly available image datasets of apples and strawberries [13,15,60–62]. Example results can be found in the workflow's GitHub repository: https://github.com/jorgemasgomez/almondcv2.

Clearly, recent advancements in AI segmentation models, such as YOLO [47] and SAM [41], enable breeding programs to develop fine-tuned models for specific applications, even without large datasets. Additionally, progress in labeling tools like CVAT [42], which integrate AI-assisted features, accelerates the tedious and time-consuming labeling process. The new workflow designed here uses Jupyter notebooks for ease of customisation, eliminating the need for high-performance hardware (e.g., GPUs),

allowing users to run the tool efficiently in cloud-based environments such as Google Colab. Moreover, the reconstruction approaches employed showed good performance in general terms. A lower performance was noticed only in the shell 2022 using SAHI, which may be improved with SAHI parameter optimisation.

The thickness estimation models demonstrated high accuracy, highlighting the usefulness of combining imaging phenotyping tools with traditional phenotyping processes, such as weighing [63]. This approach enhances the phenotyping process by offering an alternative to capturing transversal pictures and makes phenotyping more accessible without relying on more complex instruments like 3D cameras or approaches such as 3D reconstruction [17,58]. However, the approach employed takes advantage of the similar density among kernels, but it would not be effective for shell nuts due to the variability in shell hardness and density.

Morphometric analyses provided new quantitative traits for almond shape breeding. Both Elliptical Fourier Analysis and Pixel-based PCA showed a large variance explained by the PC1 in kernel and shell datasets. A high correlation between the different PCs1 and aspect ratio traits was identified and can also be observed graphically (Figure 3 and Supplementary Figure 7). Indeed, aspect ratio has been highly correlated with PCs1 in tomato and apple leaves [64,65], in pear fruit [66], walnut [54], and in a shape discriminator almond nut [21]. Although the interpretation of some morphometric traits could be abstract, patterns in different parts of the shape can be observed as in the tip (e.g. kernel PB-PC4), top (e.g. kernel PB-PC5), and side curvature (e.g. kernel EF-PC3). Further interpretation of these traits could be implemented by using visual guidance through text models [67]. In particular, a recent study highlighted the potential role of almond shape in breakage during processing [28], which indicates that morphology and morphometric analyses could play an important role in future research, with clear and

easy interpretation being necessary. Moreover, some morphometric traits (apart from those related to aspect ratio) showed medium heritability values (>0.3) which could place them as breeding targets. In general, all the quantitative traits extracted can be incorporated into genomic selection schemes, achieving higher accuracy than with classical shape descriptors and allowing weighted importance in selection through a selection index [8]. This approach facilitates the development of breeding populations with target kernel size and shape tailored to the requirements of the intended end use [19–21].

The number of kernels and nuts analyzed in this comparative study is considerably higher than in previous research, and the difference becomes even more pronounced when comparing metrics per season. Data points collected were from 11 to 123 times higher than those reported in other works [19,29,54–59]. More importantly, this progress has been achieved while reducing both time and economic resources, as a single person was able to phenotype the entire data set for one season in just two weeks. Currently, shell cracking remains the primary bottleneck due to the manual process, which is challenging to automate because of variability in shell hardness and size. This extensive dataset will facilitate future studies aimed at dissecting quantitative traits and implementing genomic selection approaches.

Additional Files

Supplementary Table 1. Article metrics studied related to quantitative almond morphological traits.

Supplementary Figure 1. Workflow description outlining the steps involved in developing the segmentation model (green) and deploying it (purple).

Supplementary Figure 2. YOLO metrics obtained during the fine-tuning process for the Kernel-2022 (A), Shell-2022 (B), Kernel-2023 (C), and Shell-2023 (D) datasets. A description of the metrics can be found in (Ultralytics, 2025).

Supplementary Figure 3. Explained variance (%) per EF-PC in the kernel (left) and shell (right) datasets.

Supplementary Figure 4. K-means clustering using EFA-PCA results in the shell dataset, showing the shapes corresponding to each centroid for scenarios ranging from k=1 to k=10.

Supplementary Figure 5. Within group sum of squares decay for K-means clustering using EFA-PCA results in kernel (left) and shell (right) dataset.

Supplementary Figure 6. Explained variance (%) per PB-PC in the kernel (left) and shell (right) datasets.

Supplementary Figure 7. Representation of the influence of the PB-PCs on shape in the kernel (left) and shell (right) datasets, from the mean shape to ±3× standard deviation.

Supplementary Figure 8. K-means clustering using PB-PCA results in the shell dataset, showing the shapes corresponding to each centroid for scenarios ranging from k=1 to k=10.

Supplementary Figure 9. Within group sum of squares decay for K-means clustering using PB-PCA results in kernel (left) and shell (right) dataset.

Abbreviations

EFA: Elliptical Fourier analysis; GPU: Graphics Processing Unit; H2: Broad sense heritability; NGS: Next-generation sequencing; PCA: Principal component analysis;

QTL: quantitative trait loci; RMSE: Root mean squared error; ROI: Region of Interest; SAHI: Slicing Aided Hyper Inference; SAM: Segment Anything Model; YOLO: You Only Look Once

Acknowledgements

We would like to thank María del Mar Gómez Abajo, Francisco Gómez Lopez, Lucía Rodríguez Robles, Antonio Moreno Marín, Luis Miguel Serrano Sánchez, and Teresa Cremades Rosado for their assistance with the sampling, as well as Juan Antonio Tudela for lending us his camera and set.

Author contributions

J.M.G. developed the phenotyping workflow, implemented the technical components, and performed image processing and analyses. J.M.G. and P.J.M.G. conceived and designed the study. J.M.G., P.J.M.G., and M.R. carried out field sampling. M.R. and F.D. contributed to the revision and refinement of the manuscript. All authors participated in the writing and revision of the manuscript and approved the final version.

Funding

This work was supported by grant PID2021-127421OB-I00 funded by MICIU/AEI/10.13039/501100011033, by grant CNS2022-135936 funded by MICIU/AEI /10.13039/501100011033 and European Union NextGenerationEU/PRTR and by "ERDF A way of making Europe" and formed part of the AGROALNEXT program and was supported by MCIN with funding from European Union NextGenerationEU (PRTR-C17.I1) and by the Fundación Séneca with funding from Comunidad Autónoma Región de Murcia (CARM). J.M.-G acknowledges the Spanish MICIU for his predoctoral grant (FPU20/00614).

Availability of source code and requirements

Project name: AlmondCV

Project home page: https://github.com/jorgemasgomez/almondcv2

Operating system(s): Platform independent

Programming language: Python, R

Other requirements: see public environment file released under GNU GPL v3

RRID: SCR_027064

WorkflowHub: https://workflowhub.eu/workflows/1731

Data availability

The data supporting the results of this article are available in the Zenodo repository [68].

Temporal restricted link for reviewers.

Competing interests

The authors declare that they have no competing interests.

References

1. Yang W, Feng H, Zhang X, Zhang J, Doonan JH, Batchelor WD, et al.. Crop Phenomics and High-Throughput Phenotyping: Past Decades, Current Challenges, and Future Perspectives. *Molecular Plant*. 2020; doi: 10.1016/j.molp.2020.01.008.

2. Moore CR, Johnson LS, Kwak I-Y, Livny M, Broman KW, Spalding EP. High-throughput computer vision introduces the time axis to a quantitative trait map of a plant growth response. *Genetics*. 2013; doi: 10.1534/genetics.113.153346.

3. Sheikh M, Iqra F, Ambreen H, Pravin KA, Ikra M, Chung YS. Integrating artificial intelligence and high-throughput phenotyping for crop improvement. *Journal of Integrative Agriculture*. 2024; doi: 10.1016/j.jia.2023.10.019.

4. Condorelli GE, Maccaferri M, Newcomb M, Andrade-Sanchez P, White JW, French AN, et al.. Comparative Aerial and Ground Based High Throughput Phenotyping for the Genetic Dissection of NDVI as a Proxy for Drought Adaptive Traits in Durum Wheat. *Front Plant Sci*. Frontiers; 2018; doi: 10.3389/fpls.2018.00893.

5. Azevedo CF, Ferrão LFV, Benevenuto J, de Resende MDV, Nascimento M, Nascimento ACC, et al.. Using visual scores for genomic prediction of complex traits in breeding programs. *Theor Appl Genet*. 2023; doi: 10.1007/s00122-023-04512-w.

6. Gorjanc G, Cleveland MA, Houston RD, Hickey JM. Potential of genotyping-by-sequencing for genomic selection in livestock populations. *Genet Sel Evol*. 2015; doi: 10.1186/s12711-015-0102-z.

7. Gorjanc G, Dumasy J-F, Gonen S, Gaynor RC, Antolin R, Hickey JM. Potential of Low-Coverage Genotyping-by-Sequencing and Imputation for Cost-Effective Genomic Selection in Biparental Segregating Populations. *Crop Science*. 2017; doi: 10.2135/cropsci2016.08.0675.

8. Kizilkaya K, Fernando RL, Garrick DJ. Reduction in accuracy of genomic prediction for ordered categorical data compared to continuous observations. *Genetics Selection Evolution*. Springer; 46:372014;

9. Singh A, Ganapathysubramanian B, Singh AK, Sarkar S. Machine Learning for High-Throughput Stress Phenotyping in Plants. *Trends Plant Sci*. 2016; doi: 10.1016/j.tplants.2015.10.015.

10. Kim JY. Roadmap to High Throughput Phenotyping for Plant Breeding. *J Biosyst Eng*. 2020; doi: 10.1007/s42853-020-00043-0.

11. Huang Y, Ren Z, Li D, Liu X. Phenotypic techniques and applications in fruit trees: a review. *Plant Methods*. 2020; doi: 10.1186/s13007-020-00649-7.

12. Kirchgessner N, Hodel M, Studer B, Patocchi A, Broggini GAL. FruitPhenoBox – a device for rapid and automated fruit phenotyping of small sample sizes. *Plant Methods*. 2024; doi: 10.1186/s13007-024-01206-2.

13. Keller B, Jung M, Bühlmann-Schütz S, Hodel M, Studer B, Broggini GAL, et al.. The genetic basis of apple shape and size unraveled by digital phenotyping. *G3 (Bethesda)*. 2024; doi: 10.1093/g3journal/jkae045.

14. Jurado-Ruiz F, Rousseau D, Botía JA, Aranzana MJ. GenoDrawing: An Autoencoder Framework for Image Prediction from SNP Markers. *Plant Phenomics*. 2023; doi: 10.34133/plantphenomics.0113.

15. Feldmann MJ, Hardigan MA, Famula RA, López CM, Tabb A, Cole GS, et al.. Multi-dimensional machine learning approaches for fruit shape phenotyping in strawberry. *GigaScience*. 2020; doi: 10.1093/gigascience/giaa030.

16. Zingaretti LM, Monfort A, Pérez-Enciso M. Automatic Fruit Morphology Phenome and Genetic Analysis: An Application in the Octoploid Strawberry. *Plant Phenomics*. 2021; doi: 10.34133/2021/9812910.

17. Feldmann MJ, Tabb A. Cost-effective, high-throughput phenotyping system for 3D reconstruction of fruit form. *The Plant Phenome Journal*. 2022; doi: 10.1002/ppj2.20029.

18. Janick J, Schirra M. Postharvest technology and utilization of almonds. *Horticultural Reviews*. 1997;

19. Martínez-García PJ, Rubio M, Cremades T, Dicenta F. Inheritance of shell and kernel shape in almond (Prunus dulcis). *Scientia Horticulturae*. 2019; doi: 10.1016/j.scienta.2018.09.041.

20. Socias R, Kodad O, Alonso J, Gradziel T. Almond Quality: A Breeding Perspective. *Horticultural Reviews*. 2008;

21. Demir B, Sayinci B, Çetin N, Yaman M, Çömlek R. Shape Discrimination of Almond Cultivars by Elliptic Fourier Descriptors. *Erwerbs-Obstbau*. 2019; doi: 10.1007/s10341-019-00423-7.

22. Verdú A, Izquierdo S, Socias i Company R. Processing and industrialization. *Almonds: botany, production and uses*.

23. Klingenberg CP, Debat V, Roff DA. Quantitative genetics of shape in cricket wings: developmental integration in a functional structure. *Evolution*. 2010; doi: 10.1111/j.1558-5646.2010.01030.x.

24. Claes P, Shriver MD. Establishing a Multidisciplinary Context for Modeling 3D Facial Shape from DNA. *PLOS Genetics*. Public Library of Science; 2014; doi: 10.1371/journal.pgen.1004725.

25. Dryden IL, Mardia KV. Statistical shape analysis. Wiley;

26. Altuntas E, Gercekcioglu R, Kaya C. Selected Mechanical and Geometric Properties of Different Almond Cultivars. *International Journal of Food Properties*. Taylor & Francis; 2010; doi: 10.1080/10942910802331504.

27. Koyuncu MA, Ekinci K, Gun A. The effects of altitude on fruit quality and compression load for cracking of walnuts (Juglans regia L.). *Journal of Food Quality*. John Wiley & Sons, Ltd; 2004; doi: 10.1111/j.1745-4557.2004.00689.x.

28. Lipan L, Miarnau X, Cutrone M, Calle A, Sendra E, Carbonell-Barrachina ÁA, et al.. Impact of industrial shelling and blanching on almond kernel integrity and color. *LWT*. 2025; doi: 10.1016/j.lwt.2025.117472.

29. Pérez de Los Cobos F, Romero A, Lipan L, Miarnau X, Arús P, Eduardo I, et al.. QTL mapping of almond kernel quality traits in the F1 progeny of “Marcona” × “Marinada.” *Front Plant Sci*. 2024; doi: 10.3389/fpls.2024.1504198.

30. Vidyarthi SK, Tiwari R, Singh SK. Stack ensembled model to measure size and mass of almond kernels. *Journal of food process Engineering*. Wiley Online Library; 43:e133742020;

31. Zhu F, Paul P, Hussain W, Wallman K, Dhatt BK, Sandhu J, et al.. SeedExtractor: An Open-Source GUI for Seed Image Analysis. *Frontiers in Plant Science*. Volume 11-20202021;

32. Whan AP, Smith AB, Cavanagh CR, Ral J-PF, Shaw LM, Howitt CA, et al.. GrainScan: a low cost, fast method for grain size and colour measurements. *Plant Methods*. 2014; doi: 10.1186/1746-4811-10-23.

33. Tanabata T, Shibaya T, Hori K, Ebana K, Yano M. SmartGrain: High-Throughput Phenotyping Software for Measuring Seed Shape through Image Analysis. *Plant Physiology*. 2012; doi: 10.1104/pp.112.205120.

34. Gehan MA, Fahlgren N, Abbasi A, Berry JC, Callen ST, Chavez L, et al.. PlantCV v2: Image analysis software for high-throughput plant phenotyping. *PeerJ*. PeerJ Inc.; 2017; doi: 10.7717/peerj.4088.

35. Ngugi LC, Abelwahab M, Abo-Zahhad M. Recent advances in image processing techniques for automated leaf pest and disease recognition – A review. *Information Processing in Agriculture*. 2021; doi: 10.1016/j.inpa.2020.04.004.

36. Sapkota R, Ahmed D, Karkee M. Comparing YOLOv8 and Mask R-CNN for instance segmentation in complex orchard environments. *Artificial Intelligence in Agriculture*. 2024; doi: 10.1016/j.aiia.2024.07.001.

37. Xue W, Ding H, Jin T, Meng J, Wang S, Liu Z, et al.. CucumberAI: Cucumber Fruit Morphology Identification System Based on Artificial Intelligence. *Plant Phenomics*. American Association for the Advancement of Science; 2024; doi: 10.34133/plantphenomics.0193.

38. Katal N, Rzanny M, Mäder P, Wäldchen J. Deep Learning in Plant Phenological Research: A Systematic Literature Review. *Front Plant Sci*. Frontiers; 2022; doi: 10.3389/fpls.2022.805738.

39. Gu W, Bai S, Kong L. A review on 2D instance segmentation based on deep neural networks. *Image and Vision Computing*. 2022; doi: 10.1016/j.imavis.2022.104401.

40. Bommasani R, Hudson DA, Adeli E, Altman R, Arora S, Arx S von, et al.. On the Opportunities and Risks of Foundation Models. arXiv;

41. Kirillov A, Mintun E, Ravi N, Mao H, Rolland C, Gustafson L, et al.. Segment Anything. *2023 IEEE/CVF International Conference on Computer Vision (ICCV)*.

42. Sekachev B, Manovich N, Zhiltsov M, Zhavoronkov A, Kalinin D, Hoff B, et al.. opencv/cvat: v1.1.0. Zenodo;

43. Akyon FC, Onur Altinuc S, Temizel A. Slicing Aided Hyper Inference and Fine-Tuning for Small Object Detection. *2022 IEEE International Conference on Image Processing (ICIP)*.

44. : OpenCV: Camera Calibration. https://docs.opencv.org/4.x/dc/dbb/tutorial_py_calibration.html (2025). Accessed 2025 Jan 31.

45. Pereira A, Santos C, Aguiar M, Welfer D, Dias M, Ribeiro M, et al.. Detection of retinal microlesions through YOLOR-CSP architecture and image slicing with the SAHI algorithm. *2023 International Joint Conference on Neural Networks (IJCNN)*.

46. Saradopoulos I, Potamitis I, Rigakis I, Konstantaras A, Barbounakis IS. Image Augmentation Using Both Background Extraction and the SAHI Approach in the Context of Vision-Based Insect Localization and Counting. *Information*. Multidisciplinary Digital Publishing Institute; 2025; doi: 10.3390/info16010010.

47. Redmon J, Divvala S, Girshick R, Farhadi A. You Only Look Once: Unified, Real-Time Object Detection. *2016 IEEE Conference on Computer Vision and Pattern Recognition (CVPR)*.

48. : Train - Ultralytics YOLO Docs. https://docs.ultralytics.com/es/modes/train/ (2025). Accessed 2025 Jan 31.

49. Bradski G, Kaehler A. OpenCV. *Dr Dobb's journal of software tools*. 32000;

50. : OpenCV: Image Segmentation with Watershed Algorithm. https://docs.opencv.org/4.x/d3/db4/tutorial_py_watershed.html (2025). Accessed 2025 Jan 31.

51. Bonhomme V, Picq S, Gaucherel C, Claude J. Momocs: Outline Analysis Using R. *Journal of Statistical Software*. 2014; doi: 10.18637/jss.v056.i13.

52. Seabold S, Perktold J. Statsmodels: Econometric and Statistical Modeling with Python. *scipy*. 2010; doi: 10.25080/Majora-92bf1922-011.

53. Ultralytics. YOLO Pre-trained Segmentation Models. https://docs.ultralytics.com/es/tasks/segment/#models;

54. Demir B, Sayıncı B, Çetin N, Yaman M, Çömlek R, Aydın Y, et al.. Elliptic Fourier based analysis and multivariate approaches for size and shape distinctions of walnut (Juglans regia L.) cultivars. *Grasas y Aceites*. 2018; doi: 10.3989/gya.0104181.

55. Fernández i Martí A, Font i Forcada C, Socias i Company R. Genetic analysis for physical nut traits in almond. *Tree Genetics & Genomes*. 2013; doi: 10.1007/s11295-012-0566-8.

56. Forcada CF i, Oraguzie N, Reyes-Chin-Wo S, Espiau MT, Company RS i, Martí AF i. Identification of Genetic Loci Associated with Quality Traits in Almond via Association Mapping. *PLOS ONE*. Public Library of Science; 2015; doi: 10.1371/journal.pone.0127656.

57. Goonetilleke SN, Wirthensohn MG, Mather DE. Genetic analysis of quantitative variation in almond nut traits. *Tree Genetics & Genomes*. 2023; doi: 10.1007/s11295-023-01630-w.

58. Sánchez-Beeckman M, Fornés Comas J, Martorell O, Alonso Segura JM, Buades A. Three-dimensional image analysis for almond endocarp feature extraction and shape description. *Computers and Electronics in Agriculture*. 2024; doi: 10.1016/j.compag.2024.109420.

59. Sorkheh K, Shiran B, Khodambashi M, Moradi H, Gradziel TM, Martínez-Gómez P. Correlations between quantitative tree and fruit almond traits and their implications for breeding. *Scientia Horticulturae*. 2010; doi: 10.1016/j.scienta.2010.04.014.

60. Feldmann M J, Hardigan M A, Famula R A, López C M, Tabb A, Cole G S, et al.. Supporting data for "Multi-Dimensional Machine Learning Approaches for Fruit Shape Phenotyping in Strawberry." GigaScience Database;

61. Feldmann MJ. Classification and Quantification of Strawberry Fruit Shape. 2019; doi: 10.5281/zenodo.3365714.

62. Keller B, Jung M, Bühlmann-Schütz S, Hodel M, Studer B, Broggini G, et al.. Dataset - The genetic basis of apple shape and size unraveled by digital phenotyping. ETH Zurich;

63. Utai K, Nagle M, Hämmerle S, Spreer W, Mahayothee B, Müller J. Mass estimation of mango fruits (Mangifera indica L., cv. 'Nam Dokmai') by linking image processing and artificial neural network. *Engineering in Agriculture, Environment and Food*. 2019; doi: 10.1016/j.eaef.2018.10.003.

64. Chitwood DH, Kumar R, Headland LR, Ranjan A, Covington MF, Ichihashi Y, et al.. A Quantitative Genetic Basis for Leaf Morphology in a Set of Precisely Defined Tomato Introgression Lines. *The Plant Cell*. 2013; doi: 10.1105/tpc.113.112391.

65. Migicovsky Z, Li M, Chitwood DH, Myles S. Morphometrics Reveals Complex and Heritable Apple Leaf Shapes. *Front Plant Sci*. Frontiers; 2018; doi: 10.3389/fpls.2017.02185.

66. Wang H, Yin H, Li H, Wu G, Guo W, Qi K, et al.. Quantitative 2D fruit shape analysis of a wide range of pear genetic resources toward shape design breeding. *Scientia Horticulturae*. 2024; doi: 10.1016/j.scienta.2023.112826.

67. Zhao K, Wu X, Xiao Y, Jiang S, Yu P, Wang Y, et al.. PlanText: Gradually Masked Guidance to Align Image Phenotypes with Trait Descriptions for Plant Disease Texts. *Plant Phenomics*. AAAS; 6:02722024;

68. Mas-Gómez J, Rubio M, Dicenta F, Martínez-García PJ. Dataset AlmondCV Pictures. 2025; doi: 10.5281/zenodo.15423562.

Manuscript_trackchanges_version

Click here to access/download;Manuscript;renamed_b3acf.docx

Open RGB Imaging Workflow for Morphological and Morphometric Analysis of Fruits using ~~AI~~D~~d~~eep L~~l~~earning: A Case Study on Almonds.

Jorge Mas-Gómez [0000-0002-3663-6743]

Manuel Rubio [0000-0003-4605-8227]

Federico Dicenta [0000-0002-7647-5235]

Pedro José Martínez-García* [0000-0002-7647-5235]

Fruit Breeding Group. Department of Plant Breeding, Centro de Edafología y Biología Aplicada del Segura- Spanish National Research Council (CEBAS-CSIC). Campus Universitario Espinardo, E-30100 Murcia, Spain

*Corresponding author: pjmgarcia@cebas.csic.es

Formatted: English (United States)

Abstract

Background

High-throughput phenotyping is addressing the current bottleneck in phenotyping within breeding programs. Imaging tools are becoming the primary resource for improving the efficiency of phenotyping processes and providing large datasets for genomic selection approaches. The advent of AI brings new advantages by enhancing phenotyping methods using imaging, making them more accessible to breeding programs. In this context, we have developed an open Python workflow for analyzing morphology, colour and morphometric traits using AI, which can be applied to fruits and other plant organs.

Results

The workflow was implemented in almond *(Prunus dulcis* (Mill.) D. A. Webb), a species where breeding efficiency is critical due to its long breeding cycle. Over 25,000 kernels, more than 20,000 nuts, and over 600 individuals were phenotyped, making this the largest morphological study conducted in almond so far. The best segmentation and reconstruction approaches achieved error rates below 1%. Weight and area variables enabled accurate estimation of kernel thickness, with a root mean squared error (RMSE) of 0.47. Fifty-five heritable morphological, morphometric and colour traits were identified, highlighting their potential as target traits in breeding programs.

Conclusion

The proposed workflow demonstrated robust performance across diverse datasets and being effective with limited training data for fine-tuning. Its compatibility with the output of AI-based labelling tools allows users to fully leverage the advantages of these technologies—reducing manual effort, accelerating dataset preparation, and streamlining

the fine-tuning process of segmentation models. This flexibility enhances the scalability and practical applicability of the workflow in real-world phenotyping scenarios, especially in the context of breeding programs.

Keywords

High-throughput Phenotyping, Almonds, Breeding, Computer Vision, Deep learning

Background

An observable performance of a plant (resulting from its genotype, environment, and their interaction) is defined as the phenotype. Phenotypes allow the portion attributable to genetic effects to be identified through quantitative genetics. The process of measuring and describing plant phenotypes is called phenotyping, and it remains the primary bottleneck in breeding programs [1][5]. To overcome this bottleneck, classical phenotyping tasks, which are often tedious, time-consuming, subjective and/or expensive, are being replaced. Breeders are leveraging tools such as computer vision [2], artificial intelligence [3], and unmanned aerial platforms [4], among others, to meet the need for large-scale phenotyping and the generation of accurate, accessible big datasets [1]. Such high-throughput phenotyping enables to phenotype larger populations and the generation of quantitative measurements, which can improve prediction accuracy and response to selection in Genomic Selection (GS) schemes [5–8]. Clearly, hHigh-throughput phenotyping is becoming a valuable tool for plant breeders and researchers to meet the challenges of future food demand [9,10]. Currently, breeding programs operate in a scenario where genotype data has expanded exponentially due to the accessibility of next-generation sequencing (NGS) technologies . Consequently, the implementation of genomic selection approaches and candidate gene discovery has advanced significantly . Genomic selection (GS) is a breeding approach which accelerates genetic gain by using large numbers of molecular markers (commonly thousands of single nucleotide polymorphisms SNPs) in combination with phenotypic data to estimate the genetic potential of individuals (breeding value) . Phenotypeing is the foundation of plant breeding, as it reflects an individual's observable performance (resulting from genotype, environment, and their interaction) and allows the portion attributable to genetic effects to be identified through quantitative genetics . However, these approaches also depend

on phenotypingphenotyping, which remains the primary bottleneck in breeding programs . To overcome this bottleneck, classical phenotyping tasks, which are often tedious, time-consuming, subjective and/or expensive, are being replaced. Breeders are leveraging tools such as computer vision , artificial intelligence , and unmanned aerial platforms , among others, to meet the need for large-scale phenotyping and the generation of accurate, accessible big datasets . Such high-throughput phenotyping enables to phenotype larger populations and the generation of quantitative measurements, which can improve prediction accuracy and response to selection in GS schemes.

Formatted: Font: (Default) Times New Roman

The use of high-throughput phenotyping platforms for fruit species such as apple, mango, vineyard or citrus have helped to study architecture parameters, pigment and nutrient contents, water stress, biochemical parameters of fruits and disease detection [11]. A device for fast and accurate phenotyping of small fruit samples called “FruitPhenoBox“ was developed for breeding programs [12], leading to the identification of key marker-trait associations in apple populations [13]. Additionally, by integrating genotype data, RGB imaging, and autoencoders, a framework was developed to reconstruct apple samples using only genotype data [14]. Morphometric approaches using machine learning have been applied in strawberries to extract quantitative shape features, enabling the identification of new heritable traits [15]. Furthermore, an automatic pipeline to phenotype morphological traits was developed for strawberries using computer vision techniques [16] and 3D imaging [17], with applicability to other fruits.

For stone fruit trees species, such as almond (*Prunus dulcis* (Mill.) D. A. Webb) the implementation of these tools is still scarce. Breeding almond trees, and other *Prunus* species such as apricot, peach, plum or cherry is a long and expensive task. Due to their long juvenility period the timing to obtain a new improved cultivar is around 12-15 years. Therefore, increasing efficiency is a key aspect for breeding these species, particularly in

phenotyping processes of traits of interest such as fruit morphology, shape and colour [18–20]. -These traits directly influence market value and consumer acceptance [21,22] and as have been commented, these traits are highly dimensional [16] and computing only linear, univariate phenotype leads to a loss of information by extremely simplifying the features of a trait [23,24], requiring the use of geometric-morphometric approaches [25].

~~These traits directly influence market value and consumer acceptance.~~ Larger kernel sizes are generally more economically valuable [20], while smaller sizes are preferred for specific applications, such as chocolate bars [18]. Shape is also important for industrial processes: elongated almonds are preferred for slicing, whereas round almonds are generally more appreciated by consumers [18,19]. Geometric parameters also affect the mechanical properties of almonds and other nuts, which is relevant for processing [26,27]. For example, elongated shapes have been shown to result in lower breakage during blanching, while rounder shapes reduce breakage during shelling in Spanish almond industry machinery [28]. Preliminary efforts in imaging analysis and deep learning in almond have demonstrated that the same quantitative trait loci (QTLs) for size and shape can be successfully identified using these approaches as with traditional manual phenotyping methods [29]. Imaging has also been applied in "Nonpareil" almond ~~variety~~cultivar, to measure length and predict kernel mass using machine learning, for industrial applications [30]. Moreover, 3D imaging phenotyping methods have been developed for almonds nuts. –Software for measuring seed morphology has been developed, enabling the quantification of both shape and color traits~~.~~ [31–33]. However, none of these tools allow for detailed morphometric analyses of kernel shape, which can be an important commercial trait in almonds [21]. A common challenge is that elements (such as seeds or fruits) often touch each other [31] and algorithms such as watershed segmentation are not implemented in all available software. Among these tools, only

SeedExtractor is open-source, providing a foundation for customization and further development, although it is implemented in Matlab [31]. In contrast, widely adopted programming languages can facilitate faster and broader adoption. In this regard, PlantCV, written in Python, offers numerous tutorials for learning computer vision in plant phenotyping [34].

In most of the mentioned studies, segmentation — defined as the process of partitioning a digital image into meaningful regions of interest (ROIs) based on homogeneous visual features such as colour, texture, or intensity — ~~relies on computer vision algorithms~~is a core computer vision task, which its conventional use through binary thresholding often require manual adjustments, limiting automation—especially as scene complexity increases [35,36]. To enhance efficiency and automation, advanced tools capable of handling this complexity are essential for fruit morphology studies [37]. In this sense, deep learning techniques have gained significant relevance due to their high segmentation accuracy, even in complex scenarios, by leveraging large training and validation datasets [38]. Instance segmentation using deep learning can be implemented through two main approaches: two-stage models (e.g., Mask R-CNN~~, Faster R-CNN~~) and one-stage models (e.g., *You look only once* (YOLO), RetinaNet) [39]. While two-stage models offer higher accuracy at the cost of lower speed and greater computational requirements, one-stage models aim to balance accuracy and speed while using fewer resources [39]. Notably, recent advancements in YOLO models have significantly improved their accuracy while maintaining high-speed performance [36]. Nevertheless, training deep learning models from scratch requires large labelled datasets and substantial computational resources [40]. In this context, fine-tuning pre-trained generalist models allows users to develop customized models without the need for extensive datasets [40,41]. The integration of deep learning tools for labelling datasets has significantly accelerated this task [41,42].

Moreover, new techniques such as Slicing Aided Hyper Inference (SAHI) have enhanced accuracy by reducing errors in the segmentation of small objects, making it particularly valuable for seed imagery [43]. The slicing process also reduces memory requirements for processing large images, maintaining high resolution without the need to resize them for model training, and enabling the utilization of limited GPU (Graphics Processing Unit) open resources (e.g., Google Colab).

In this work, we have developed a Python-based workflow to facilitate the development and deployment of custom deep learning models for measuring morphological and morphometric traits in fruit breeding programs. The main contribution of the workflow ~~includes~~ is the integration of pre-processing methods such as colour and distortion correction, development and deployment of image segmentation fine-tuned generalist models, ~~—~~morphology measurements, and morphometric analyses~~and pixel size estimation.~~ ~~and i~~The workflow~~t~~ was successfully applied to phenotype multiple almond breeding populations, allowing for the estimation of broad-sense heritability values. Additionally, prediction models were developed to estimate kernel thickness based on area and weight. By implementing this workflow, the study has analyzed the largest number of individuals and data points ever recorded for the almond species to date.

Material and Methods

Plant material

The workflow was implemented to study breeding populations of the CEBAS-CSIC almond breeding program located in the experimental field (Santomera, Murcia SE of Spain) during 2022 and 2023 seasons. 665 unique genotypes from six F1 populations and

a germplasm collection were studied (Table 1). Population parents were traditional cultivars (‘Marcona’ and ‘Desmayo Largueta’), released cultivars of CEBAS-CSIC (‘Antoñeta’, ‘Penta’, ‘Tardona’, and ‘Florida’) and the breeding selections ‘R1000’ (INRA, France) and ‘S4017’ (CEBAS-CSIC). The target samples were shells and kernels of these almond descendants. For each genotype, 30 in-shell nuts and 30 kernels per year were sampled, photographed, and weighed, whenever available.

Table 1. The populations used in this study and the number of individuals studied per year

**Year**	**2022**		**2023**		**Unique genotypes**	
**Family**	**Shell**	**Kernel**	**Shell**	**Kernel**	**Shell**	**Kernel**
Germplasm collection	85	85	74	91	99	99
Antoñeta × Marcona	6	6	19	19	19	19
Antoñeta × Penta	161	161	142	142	183	183
Antoñeta × Tardona	57	57	56	53	71	70
Florida × Marcona	17	17	43	43	44	44
Desmayo × R1000	198	198	187	194	223	223
Marcona × S4017	0	0	26	27	26	27
Total	524	524	547	569	665	665

Workflow description

A comprehensive workflow has been developed for pre-processing, segmentation model development, deployment, morphological measurements, and morphometric analyses, implemented through interactive Python notebooks (Supplementary Figure 1) (https://github.com/jorgemasgomez/almondcv2). To optimize speed and performance, the use of a GPU is highly recommended. The workflow can be executed locally; however, it is also deployed on the Google Colab platform, enabling users to run it online and leverage free resources, such as GPUs. Although primarily written in Python, some functions integrate R for morphometric analyses, requiring R to be properly configured in the system's path if executed locally.

Colour and distortion calibration

Colour and distortion correction approaches are included in the pre-processing notebook (*1_Pre-processing_workflow.ipynb*). Colour correction enables to standardize the dataset of the images using a colour card as reference. Colour correction functions implemented in the pre-processing notebook are based on the colour correction module of PlantCV python library [34]. Distortion correction is implemented for possible radial and tangential distortion caused by the camera. Distortion correction functions are based on the OpenCV workflow for camera calibration [44]. It requires test patterns of chessboards with a known size of the squares to obtain a camera matrix that can be used subsequently with the pictures of the dataset. In addition, colour and distortion correction functions are joined in the pre-processing notebook to deploy it in the whole dataset in one step.

Auxiliary functions

Some optional auxiliary functions were included for the picture pre-processing, helping to separate the different samples in the pictures and getting the physical size of a pixel with reference objects in the picture (e.g. a coin with known diameter). These optional

functions require training a model (next sections) to identify and segment the sample groups and the reference objects.

Develop your segmentation model

The approach implemented in the present work intends to simplify the development of customized segmentation models. Four key steps are necessary for performing the development: slicing, labelling, ‘training’ and reconstruction (*2_Develop_your_segmentation_model_workflow.ipynb*). The slicing process consists of cropping pictures into smaller patches, with the size determined by the user. Using smaller patches of high-resolution images speeds up the training and prediction processes by reducing computational requirements [43]. Additionally, this approach improves the capture of fine details, as it avoids the loss of resolution and distortion caused by resizing the entire image [45,46]. The slicing function defined in the workflow is divided into train, validation, and test datasets. The slices are obtained proportionally according to the user command to prepare for the next step. The second step, labelling, involves annotating which pixels correspond to the object of interest. For this task, the workflow was designed to accept as input the ZIP file generated by the image annotation platform CVAT in the YOLO Segmentation 1.0 format [42]. CVAT provides tools for semi-automatic segmentation using the Segment Anything Model (SAM) [41], which enables quick instance segmentation with a single click. CVAT is available for free offline via a Docker container or online with a freemium model. The third step for developing the segmentation model is ‘training’ the model. For that purpose, in the second notebook (*2_Develop_your_segmentation_model_workflow.ipynb*) YOLO [47] pre-trained algorithm series can be fine-tuned (although in their web mention ‘training’ really is a fine-tuning [48]) using our custom labelled dataset. The training process function enables to control all the parameters of the training process (e.g. epochs, batch) and provides the

results of the training *YOLO.train* method. The last step for developing the segmentation model is to reconstruct the binary picture mask, for which two approaches were used. The first approach simply joins the patches binary masks after the prediction (*slice_predict_reconstruct*). The contours are detected using *cv2.findcontours* OpenCV function [49] in the subsequent functions for morphology measurements. Watershed algorithm is implemented in such functions for separating touching objects of interest [50]. The second approach uses the SAHI pipeline (*predict_model_sahi*) [43], which is integrated in the YOLO segmentation process, and the instances segmented are identified specifically providing their contours directly, solving also the problem of touching objects of interest. Here, SAHI python library is modified slightly to enable the *retina_mask* argument in YOLO prediction function and provide fine details in the segmentations. A video tutorial describing the development of the model can be found in the workflow's GitHub repository: https://github.com/jorgemasgomez/almondcv2. To assess the two reconstruction approaches, they were tested in the datasets studied and the almonds with errors in the reconstruction were annotated and removed manually.

Deploy your segmentation model for morphology and morphometric analyses

Once the segmentation model has been successfully 'trained' and tested, it can be deployed for morphology and morphometric analyses. In the notebook for deployment (*3_Deploy_your_segmentation_model_worflow.ipynb*), two methods were prepared to measure.

The first method is general for any group of fruit, and the traits length (the longest axis of the fruit), width (the dimension perpendicular to the length), area (the surface enclosed by the fruit contour), perimeter (the length of the outer boundary), hull-area (the area of the convex hull enclosing the shape), solidity (the ratio between area and hull-area, reflecting compactness), aspect ratio (the ratio of length to width, indicating elongation),

circularity (calculated as $4\pi\times$Area/Perimeter, with values closer to 1 indicating a more circular shape), ellipse ratio (the ratio between the minor and major axes of a fitted ellipse), and colour (L*a*b* model) are measured for each shell/kernel. This general method can be used as a template and customized according to the user's needs, for example to study additional traits or different fruit shapes. The second method is specific for almonds adding more traits such as width at three different heights (25, 50 and 75% of the length), vertical and horizontal symmetry and symmetry in the top part of the almond (to measure the shoulder). In this specific method, almonds are aligned according to the angle of fitting an ellipse (*cv2.fitEllipse* function) and flipped vertically with placing the most distant point always in the lowest part (almond tip). Some extra traits derived from the results together with the weight of the kernel/shell such as the weight kernel/shell ratio, estimation of the thickness (only for kernels) and globosity (width/thickness), were also included. For the estimation of kernel thickness, a linear and quadratic model was fitted using 227 almond kernels, using the area and the weight as predictor variables. To validate the models, the dataset was split into 80% for training 20% for validating, running it 100 times to assess the stability of the models.

Both methods export picture results, table results, and binary masks for morphometric analyses. Binary masks are flipped horizontally if the lowest pixel (tip) is the right part of the picture to avoid symmetric issues in morphometric analysis.

Morphometric analyses notebook (*4_Morphometrics_workflow.ipynb*) includes two morphometric approaches: Elliptical Fourier analysis (EFA) and pixel-based principal component analysis (PCA). EFA is conducted using Momocs v1.4.1. R package [51] but it has been implemented in the notebook in Python programming language executing a subprocess. Several functions are included for EFA: exploratory analysis, running the EFA, after that perform a PCA on the EFA results and *kmeans* clustering. PCA performed

on EFA coefficients generates as traits the components (EF-PCs) that explain the variability of the shape. Regarding the second pixel pixel-based PCA, binary masks are flattened for performing the PCA and generating also PCs as traits (PB-PCs)[15]. Result plots of both approaches can be obtained to show the influence of each trait.

RGB Imaging system

For image acquisition, group pictures of various samples were captured over a black surface template, with the samples positioned inside painted rectangles. For the 2022 dataset, images were taken using a Canon EOS 70D camera, achieving a resolution of 6 px/mm. In 2023, the imaging system was redefined due to objectives beyond the scope of this work, utilising an Arducam 8MP IMX219 camera, which resulted in images with a resolution of 4 px/mm. Both systems were illuminated using two white LED light sources.

Heritability

Broad sense heritability (H^2) was estimated using the Python library *statsmodels* [52], fitting a linear mixed model using the genotype as random effects and the year as fixed effects (Equations 1 and 2).

$$Y_{ijk} = \mu + G_i + Y_j + \varepsilon_{ijk} \quad (\textit{Equation 1})$$

Where:

Y_{ijk}: Observed phenotypic value for the k-th observation of the i-th genotype in the j-th year.

μ: Overall population mean.

G_i: Random effect of the i-th genotype

Y_j: Fixed effect of the j-th year

ε_{ijk}: Residual error term

Formatted: Font:

$$\frac{\sigma^2 G}{\sigma^2 G + \sigma^2 e} \text{ (Equation 2)}$$

Where:

$\sigma^2 G$ Genetic variance.

$\sigma^2 e$: Residual variance.

Impact on almond breeding

To study the impact of this new tool on almond breeding, a comprehensive comparison with existing scientific articles related to quantitative almond morphology phenotyping was conducted. Several parameters such as the number of individuals studied, number of shells/kernels phenotyped, and the number of data points obtained, were compared to evaluate the benefits of the new high-throughput phenotyping tool.

Results

YOLO fine-tuned segmentation models performance and reconstruction errors.

A total of 46,729 elements (shell and kernels) were segmented (using watershed approach for subsequent steps), and performance errors from both reconstruction approaches were

annotated (Table 2). A standard image generated by the workflow is shown in Figure 1. Four segmentation models were fine-tuned using datasets from kernel and from shell in 2022 and 2023. The pre-trained model 'yolov11s-seg.pt' [53] was selected, and fine-tuning was performed over 100 epochs (i.e., complete passes through the entire training dataset), using 500–1,000 image slices of 320 pixels each, split into 60% for training, 20% for validation, and 20% for testing. Metrics plots from the fine-tuning process of the datasets are shown as an example in Supplementary Figure 2. ~~.~~In general, training and validation loss plots exhibited a consistent decrease in the process, with no evidence of overfitting. Precision and recall exceeded 0.9 early in training and remained stable thereafter. The Mean Average Precision (mAP) calculated at an intersection over union (IoU) threshold of 0.50 (mAP@50) reached ~1.0 values, while the more stringent, at varying IoU thresholds, ranging from 0.50 to 0.95, ~~–~~(mAP@50-95) stabilized later in training but consistently remained above 0.9. ~~-~~Validation masks were explored, showing precise results. All the combinations (dataset + reconstruction approach) showed ~~errors lower~~errors lower than 2% except for the shell 2022 dataset using SAHI (2.93%) (e~~E~~rrors were considered when an almond was incorrectly reconstructed, not detected, or if it was something other than a target sample).

Figure 1. Images generated by the workflow, displayed from left to right: original image with masks, length and width measurements, widths at different lengths, circularity, and ellipse ratio.

Table 2. Performance for different datasets and reconstruction methods, including reconstruction errors.

Method	Dataset	Errors	Total elements	Total phenotyped elements	Error percentage
Slice_predict_reconstruct + Watershed	Kernel-2022	5	10,184	10,180	0.05%
	Kernel-2023	264	15,568	15,304	1.70%
	Shell-2022	4	10,364	10,360	0.04%
	Shell-2023	60	10,945	10,885	0.55%
SAHI	Kernel-2022	14	10,184	10,178	0.14%
	Kernel-2023	139	15,568	15,429	0.89%
	Shell-2022	304	10,364	10,288	2.93%
	Shell-2023	44	10,945	10,902	0.40%

Thickness estimation

Kernel thickness was modelled using weight and area as predictor variables, employing both linear and quadratic models. The median R^2 in the test dataset for the linear model was 0.725, while for the quadratic model it was 0.795. The corresponding median Root Mean Squared Error (RMSE) values were 0.56 for the linear model and 0.47 for the quadratic model (Figure 2A). The best-performing linear and quadratic models were then plotted, achieving R^2 values of 0.847 and 0.8815, respectively, and RMSE values of 0.451 and 0.381, respectively (Figure 2B). The quadratic model trained with the complete dataset was employed for estimating thickness across all samples in subsequent analysis.

Figure 2. Results of the kernel thickness modelling, showing the R^2 (A, left) and RMSE (A, right) over 100 iterations, along with scatter plots for the best linear (B, left) and quadratic (B, right) models.

Morphometric results

Elliptical Fourier analysis (EFA) was conducted using all the kernel and shell binary masks (separately), placing 10 harmonics in both datasets after a visual exploration analysis. PCA was performed on the EFA coefficients, being 62.63%, 19.93%, and 3.83% explained by the three first PCs for the kernel analysis, and 69.19%, 15.75%, and 4.15% explained by the three first PCs for the shell analysis (Figure 3 and Supplementary Figure 3). Only the first 6 components on each dataset were used for subsequent analysis because they explained at least 1% of the variability. K-means clustering from k=1 to k=10 was run to show the main groups of the datasets (Figure 4 and Supplementary Figure 4. The optimal number of clusters was studied and no abrupt changes in the slope by elbow method were observed in any dataset (Supplementary Figure 5).

Figure 3. EFA-PCA scatterplot results for the kernel A) EF-PC1/EF-PC2 and B) EF-PC1/EF-PC3 and shell C) EF-PC1/EF-PC2 and D) EF-PC1/EF-PC3 datasets. The influence of the PCs on shape is represented in the plots.

Figure 4. K-means clustering results using EFA-PCA in kernel dataset, showing the shapes corresponding to each centroid for scenarios ranging from k=1 to k = 10.

Pixel-based PCA was performed in kernel and shell datasets, obtaining 34.96%, 8.78% and 4.75% variance explained by the three first PCs in the kernel dataset and 37.72%, 9.19% and 4.96% in the shell dataset (Supplementary Figure 6). The PCs influence in the shape was studied by plotting the +-3x deviation from the mean shape (Figure 5 and Supplementary Figure 7). Ten PB-PCs were collected for each dataset, explaining at least 1% of the variance for each one of them. K-means clustering was also carried out from k=1 to k=10 (Figure 5 and Supplementary Figure 8), and no abrupt changes were observed by the elbow method to determine the optimal number of clusters (Supplementary Figure 9).

Figure 5. Example images for Shell PB-PC3 (A) and Kernel PB-PC5 (B) morphometric traits, showing two contrasting phenotypes for each trait. Panels A1 and B1 show the original almond images; A2 and B2 show the corresponding binary masks; and A3 and B3 display the pixel-based PCA traits, from the mean shape to ±3 standard deviations.

Figure 6. K-means clustering results using PB-PCA in kernel dataset, showing the shapes corresponding to each centroid for scenarios ranging from k=1 to k = 10.

Correlations

To analyse and interpret the phenotype data obtained, the Pearson correlation between all morphological and morphometric traits was calculated (Figure 7). In absolute value, 340 pairwise correlations were higher than 0.75, with the traits of shell almond area, hull area, and perimeter exhibiting the highest number of strong pairwise correlations. High correlations (greater than 0.95) were observed between kernel and shell morphometric traits across different approaches, such as EF-PC1 with PB-PC1. Additionally, strong

correlations were found between these two PC1 components and morphological traits like aspect ratio and ellipse ratio.

Figure 7. Heatmap of the Pearson correlations between all morphological and morphometric traits.

Heritability

Broad sense heritability (H^2) was estimated for all the traits studied here (Figure 8). Fifty-five out of the 73 traits showed heritabilities ≥ 0.25. Weight ratio kernel/shell, shell weight, and shell EF-PC1 showed the highest heritability values (0.9, 0.77 and 0.70 respectively). Between morphometric traits, those related with PC1 showed high heritability values (higher than 0.68), and others such as Kernel PB-PC2, Shell PB-PC2, Shell EF-PC4 and Shell PB-PC3 showed medium values (higher than 0.3).

Figure 8. Broad-sense heritability of all traits studied in the almond populations.

Impact on almond breeding

Nine scientific articles related to almond morphology and breeding were collected to compare the performance of the high-throughput approach implemented in the current study (Supplementary Table 1 and Figure 9). A total of 25,597 kernels were studied (an average of 12,799 per season), along with 21,316 shells (an average of 10,658 per season)

(Figure 9). The entire dataset comprised 660 individuals, and 1,579,443 data points were collected (Figure 9).

Figure 9. Bar plot for comparison among articles related to quantitative almond morphology traits [19,29,54–59].

Discussion

The comprehensive workflow presented here introduces a new high-throughput phenotyping tool using almonds as a case of study for fruits. This approach has enabled the analysis of 665 genotypes—the largest dataset for almond morphological quantitatively measured traits reported to date, to the best of the authors' knowledge. The development of this new phenotyping tool represents significant progress in addressing the phenotyping bottleneck in almond breeding programs~~The development of this new phenotyping tool marks a breakthrough in addressing the phenotyping bottleneck in almonds~~. The developed ~~models~~ methods were adjusted to the target samples used but can be adapted to phenotype other kinds of fruits and other types of organs such as leaves or roots. Furthermore, as it is ~~an open~~open source, it can be used by other plants breeding programs. In fact, the workflow was successfully applied to publicly available image datasets of apples and strawberries [13,15,60–62]. Example results ~~and a video tutorial~~ can be found in the workflow's GitHub repository: https://github.com/jorgemasgomez/almondcv2.

Clearly, recent advancements in AI segmentation models, such as YOLO [47] and SAM [41], enable breeding programs to develop fine-tuned models for specific applications, even without large datasets. Additionally, progress in labeling tools like CVAT [42], which integrate AI-assisted features, accelerates the tedious and time-consuming labeling

process. The new workflow designed here uses Jupyter notebooks for ease of customisation, eliminating the need for high-performance hardware (e.g., GPUs), allowing users to run the tool efficiently in cloud-based environments such as Google Colab. Moreover, the reconstruction approaches employed showed good performance in general terms. A lower performance was noticed only in the shell 2022 using SAHI, which may be improved with SAHI parameter optimisation.

The thickness estimation models demonstrated high accuracy, highlighting the usefulness of combining imaging phenotyping tools with traditional phenotyping processes, such as weighing [63]. This approach enhances the phenotyping process by offering an alternative to capturing transversal pictures and makes phenotyping more accessible without relying on more complex instruments like 3D cameras or approaches such as 3D reconstruction [17,58]. However, the approach employed takes advantage of the similar density among kernels, but it would not be effective for shell nuts due to the variability in shell hardness and density.

Morphometric analyses provided new quantitative traits for almond shape breeding. Both Elliptical Fourier Analysis and Pixel-based PCA showed a large variance explained by the PC1 in kernel and shell datasets. A high correlation between the different PCs1 and aspect ratio traits was identified and can also be observed graphically (Figure 3 and Supplementary Figure 7). Indeed, aspect ratio has been highly correlated with PCs1 in tomato and apple leaves [64,65], in pear fruit [66], walnut [54], and in a shape discriminator almond nut [21]. Although the interpretation of some morphometric traits could be abstract, patterns in different parts of the shape can be observed as in the tip (e.g. kernel PB-PC4), top (e.g. kernel PB-PC5), and side curvature (e.g. kernel EF-PC3). Further interpretation of these traits could be implemented by using visual guidance through text models [67]. In particular, a recent study highlighted the potential role of

almond shape in breakage during processing [28], which indicates that morphology and morphometric analyses could play an important role in future research, with clear and easy interpretation being necessary. Moreover, some morphometric traits (apart from those related to aspect ratio) showed medium heritability values (>0.3) which could place them as breeding targets. In general, all the quantitative traits extracted can be incorporated into genomic selection schemes, achieving higher accuracy than with classical shape descriptors and allowing weighted importance in selection through a selection index [8]. This approach facilitates the development of breeding populations with target kernel size and shape tailored to the requirements of the intended end use [19–21].

Formatted: Font: (Default) Times New Roman, 12 pt

The number of kernels and nuts analyzed in this comparative study is considerably higher than in previous research, and the difference becomes even more pronounced when comparing metrics per season. Data points collected were from 11 to 123 times higher than those reported in other works [19,29,54–59]. More importantly, this progress has been achieved while reducing both time and economic resources, as a single person was able to phenotype the entire data set for one season in just two weeks. Currently, shell cracking remains the primary bottleneck due to the manual process, which is challenging to automate because of variability in shell hardness and size. This extensive dataset will facilitate future studies aimed at dissecting quantitative traits and implementing genomic selection approaches.

Additional Files

Supplementary Table 1. Article metrics studied related to quantitative almond morphological traits.

Supplementary Figure 1. Workflow description outlining the steps involved in developing the segmentation model (green) and deploying it (purple).

Supplementary Figure 2. YOLO metrics obtained during the fine-tuning process for the Kernel-2022 (A), Shell-2022 (B), Kernel-2023 (C), and Shell-2023 (D) datasets. A description of the metrics can be found in (Ultralytics, 2025).

Supplementary Figure 3. Explained variance (%) per EF-PC in the kernel (left) and shell (right) datasets.

Supplementary Figure 4. K-means clustering using EFA-PCA results in the shell dataset, showing the shapes corresponding to each centroid for scenarios ranging from k=1 to k=10.

Supplementary Figure 5. Within group sum of squares decay for K-means clustering using EFA-PCA results in kernel (left) and shell (right) dataset.

Supplementary Figure 6. Explained variance (%) per PB-PC in the kernel (left) and shell (right) datasets.

Supplementary Figure 7. Representation of the influence of the PB-PCs on shape in the kernel (left) and shell (right) datasets, from the mean shape to ±3× standard deviation.

Supplementary Figure 8. K-means clustering using PB-PCA results in the shell dataset, showing the shapes corresponding to each centroid for scenarios ranging from k=1 to k=10.

Supplementary Figure 9. Within group sum of squares decay for K-means clustering using PB-PCA results in kernel (left) and shell (right) dataset.

Abbreviations

EFA: Elliptical Fourier analysis; GPU: Graphics Processing Unit; H2: Broad sense heritability; NGS: Next-generation sequencing; PCA: Principal component analysis; QTL: quantitative trait loci; RMSE: Root mean squared error; ROI: Region of Interest; SAHI: Slicing Aided Hyper Inference; SAM: Segment Anything Model; YOLO: You Only Look Once

Acknowledgements

We would like to thank María del Mar Gómez Abajo, Francisco Gómez Lopez, Lucía Rodríguez Robles, Antonio Moreno Marín, Luis Miguel Serrano Sánchez, and Teresa Cremades Rosado for their assistance with the sampling, as well as Juan Antonio Tudela for lending us his camera and set.

Author contributions

J.M.G. developed the phenotyping workflow, implemented the technical components, and performed image processing and analyses. J.M.G. and P.J.M.G. conceived and designed the study. J.M.G., P.J.M.G., and M.R. carried out field sampling. M.R. and F.D. contributed to the revision and refinement of the manuscript. All authors participated in the writing and revision of the manuscript and approved the final version.

Funding

This work was supported by grant PID2021-127421OB-I00 funded by MICIU/AEI/10.13039/501100011033, by grant CNS2022-135936 funded by MICIU/AEI /10.13039/501100011033 and European Union NextGenerationEU/PRTR and by “ERDF A way of making Europe” and formed part of the AGROALNEXT program and was supported by MCIN with funding from European Union NextGenerationEU (PRTR-C17.I1) and by the Fundación Séneca with funding from

Comunidad Autónoma Región de Murcia (CARM). J.M.-G acknowledges the Spanish MICIU for his predoctoral grant (FPU20/00614).

Availability of source code and requirements

Project name: AlmondCV

Project home page: https://github.com/jorgemasgomez/almondcv2

Operating system(s): Platform independent

Programming language: Python, R

Other requirements: see public environment file released under GNU GPL v3

RRID: SCR_027064

WorkflowHub: https://workflowhub.eu/workflows/1731

Data availability

The data supporting the results of this article are available in the Zenodo repository [68].

Temporal restricted link for reviewers.

Competing interests

The authors declare that they have no competing interests.

References

1. Yang W, Feng H, Zhang X, Zhang J, Doonan JH, Batchelor WD, et al.. Crop Phenomics and High-Throughput Phenotyping: Past Decades, Current Challenges, and Future Perspectives. *Molecular Plant*. 2020; doi: 10.1016/j.molp.2020.01.008.

2. Moore CR, Johnson LS, Kwak I-Y, Livny M, Broman KW, Spalding EP. High-throughput computer vision introduces the time axis to a quantitative trait map of a plant growth response. *Genetics*. 2013; doi: 10.1534/genetics.113.153346.

3. Sheikh M, Iqra F, Ambreen H, Pravin KA, Ikra M, Chung YS. Integrating artificial intelligence and high-throughput phenotyping for crop improvement. *Journal of Integrative Agriculture*. 2024; doi: 10.1016/j.jia.2023.10.019.

4. Condorelli GE, Maccaferri M, Newcomb M, Andrade-Sanchez P, White JW, French AN, et al.. Comparative Aerial and Ground Based High Throughput Phenotyping for the Genetic Dissection of NDVI as a Proxy for Drought Adaptive Traits in Durum Wheat. *Front Plant Sci*. Frontiers; 2018; doi: 10.3389/fpls.2018.00893.

5. Azevedo CF, Ferrão LFV, Benevenuto J, de Resende MDV, Nascimento M, Nascimento ACC, et al.. Using visual scores for genomic prediction of complex traits in breeding programs. *Theor Appl Genet*. 2023; doi: 10.1007/s00122-023-04512-w.

6. Gorjanc G, Cleveland MA, Houston RD, Hickey JM. Potential of genotyping-by-sequencing for genomic selection in livestock populations. *Genet Sel Evol*. 2015; doi: 10.1186/s12711-015-0102-z.

7. Gorjanc G, Dumasy J-F, Gonen S, Gaynor RC, Antolin R, Hickey JM. Potential of Low-Coverage Genotyping-by-Sequencing and Imputation for Cost-Effective Genomic Selection in Biparental Segregating Populations. *Crop Science*. 2017; doi: 10.2135/cropsci2016.08.0675.

8. Kizilkaya K, Fernando RL, Garrick DJ. Reduction in accuracy of genomic prediction for ordered categorical data compared to continuous observations. *Genetics Selection Evolution*. Springer; 46:372014;

9. Singh A, Ganapathysubramanian B, Singh AK, Sarkar S. Machine Learning for High-Throughput Stress Phenotyping in Plants. *Trends Plant Sci*. 2016; doi: 10.1016/j.tplants.2015.10.015.

10. Kim JY. Roadmap to High Throughput Phenotyping for Plant Breeding. *J Biosyst Eng*. 2020; doi: 10.1007/s42853-020-00043-0.

11. Huang Y, Ren Z, Li D, Liu X. Phenotypic techniques and applications in fruit trees: a review. *Plant Methods*. 2020; doi: 10.1186/s13007-020-00649-7.

12. Kirchgessner N, Hodel M, Studer B, Patocchi A, Broggini GAL. FruitPhenoBox – a device for rapid and automated fruit phenotyping of small sample sizes. *Plant Methods*. 2024; doi: 10.1186/s13007-024-01206-2.

13. Keller B, Jung M, Bühlmann-Schütz S, Hodel M, Studer B, Broggini GAL, et al.. The genetic basis of apple shape and size unraveled by digital phenotyping. *G3 (Bethesda)*. 2024; doi: 10.1093/g3journal/jkae045.

14. Jurado-Ruiz F, Rousseau D, Botía JA, Aranzana MJ. GenoDrawing: An Autoencoder Framework for Image Prediction from SNP Markers. *Plant Phenomics*. 2023; doi: 10.34133/plantphenomics.0113.

15. Feldmann MJ, Hardigan MA, Famula RA, López CM, Tabb A, Cole GS, et al.. Multi-dimensional machine learning approaches for fruit shape phenotyping in strawberry. *GigaScience*. 2020; doi: 10.1093/gigascience/giaa030.

16. Zingaretti LM, Monfort A, Pérez-Enciso M. Automatic Fruit Morphology Phenome and Genetic Analysis: An Application in the Octoploid Strawberry. *Plant Phenomics*. 2021; doi: 10.34133/2021/9812910.

17. Feldmann MJ, Tabb A. Cost-effective, high-throughput phenotyping system for 3D reconstruction of fruit form. *The Plant Phenome Journal*. 2022; doi: 10.1002/ppj2.20029.

18. Janick J, Schirra M. Postharvest technology and utilization of almonds. *Horticultural Reviews*. 1997;

19. Martínez-García PJ, Rubio M, Cremades T, Dicenta F. Inheritance of shell and kernel shape in almond (Prunus dulcis). *Scientia Horticulturae*. 2019; doi: 10.1016/j.scienta.2018.09.041.

20. Socias R, Kodad O, Alonso J, Gradziel T. Almond Quality: A Breeding Perspective. *Horticultural Reviews*. 2008;

21. Demir B, Sayinci B, Çetin N, Yaman M, Çömlek R. Shape Discrimination of Almond Cultivars by Elliptic Fourier Descriptors. *Erwerbs-Obstbau*. 2019; doi: 10.1007/s10341-019-00423-7.

22. Verdú A, Izquierdo S, Socias i Company R. Processing and industrialization. *Almonds: botany, production and uses*.

23. Klingenberg CP, Debat V, Roff DA. Quantitative genetics of shape in cricket wings: developmental integration in a functional structure. *Evolution*. 2010; doi: 10.1111/j.1558-5646.2010.01030.x.

24. Claes P, Shriver MD. Establishing a Multidisciplinary Context for Modeling 3D Facial Shape from DNA. *PLOS Genetics*. Public Library of Science; 2014; doi: 10.1371/journal.pgen.1004725.

25. Dryden IL, Mardia KV. Statistical shape analysis. Wiley;

26. Altuntas E, Gercekcioglu R, Kaya C. Selected Mechanical and Geometric Properties of Different Almond Cultivars. *International Journal of Food Properties*. Taylor & Francis; 2010; doi: 10.1080/10942910802331504.

27. Koyuncu MA, Ekinci K, Gun A. The effects of altitude on fruit quality and compression load for cracking of walnuts (Juglans regia L.). *Journal of Food Quality*. John Wiley & Sons, Ltd; 2004; doi: 10.1111/j.1745-4557.2004.00689.x.

28. Lipan L, Miarnau X, Cutrone M, Calle A, Sendra E, Carbonell-Barrachina ÁA, et al.. Impact of industrial shelling and blanching on almond kernel integrity and color. *LWT*. 2025; doi: 10.1016/j.lwt.2025.117472.

29. Pérez de Los Cobos F, Romero A, Lipan L, Miarnau X, Arús P, Eduardo I, et al.. QTL mapping of almond kernel quality traits in the F1 progeny of “Marcona” × “Marinada.” *Front Plant Sci*. 2024; doi: 10.3389/fpls.2024.1504198.

30. Vidyarthi SK, Tiwari R, Singh SK. Stack ensembled model to measure size and mass of almond kernels. *Journal of food process Engineering*. Wiley Online Library; 43:e133742020;

31. Zhu F, Paul P, Hussain W, Wallman K, Dhatt BK, Sandhu J, et al.. SeedExtractor: An Open-Source GUI for Seed Image Analysis. *Frontiers in Plant Science*. Volume 11-20202021;

32. Whan AP, Smith AB, Cavanagh CR, Ral J-PF, Shaw LM, Howitt CA, et al.. GrainScan: a low cost, fast method for grain size and colour measurements. *Plant Methods*. 2014; doi: 10.1186/1746-4811-10-23.

33. Tanabata T, Shibaya T, Hori K, Ebana K, Yano M. SmartGrain: High-Throughput Phenotyping Software for Measuring Seed Shape through Image Analysis. *Plant Physiology*. 2012; doi: 10.1104/pp.112.205120.

34. Gehan MA, Fahlgren N, Abbasi A, Berry JC, Callen ST, Chavez L, et al.. PlantCV v2: Image analysis software for high-throughput plant phenotyping. *PeerJ*. PeerJ Inc.; 2017; doi: 10.7717/peerj.4088.

35. Ngugi LC, Abelwahab M, Abo-Zahhad M. Recent advances in image processing techniques for automated leaf pest and disease recognition – A review. *Information Processing in Agriculture*. 2021; doi: 10.1016/j.inpa.2020.04.004.

36. Sapkota R, Ahmed D, Karkee M. Comparing YOLOv8 and Mask R-CNN for instance segmentation in complex orchard environments. *Artificial Intelligence in Agriculture*. 2024; doi: 10.1016/j.aiia.2024.07.001.

37. Xue W, Ding H, Jin T, Meng J, Wang S, Liu Z, et al.. CucumberAI: Cucumber Fruit Morphology Identification System Based on Artificial Intelligence. *Plant Phenomics*. American Association for the Advancement of Science; 2024; doi: 10.34133/plantphenomics.0193.

38. Katal N, Rzanny M, Mäder P, Wäldchen J. Deep Learning in Plant Phenological Research: A Systematic Literature Review. *Front Plant Sci*. Frontiers; 2022; doi: 10.3389/fpls.2022.805738.

39. Gu W, Bai S, Kong L. A review on 2D instance segmentation based on deep neural networks. *Image and Vision Computing*. 2022; doi: 10.1016/j.imavis.2022.104401.

40. Bommasani R, Hudson DA, Adeli E, Altman R, Arora S, Arx S von, et al.. On the Opportunities and Risks of Foundation Models. arXiv;

41. Kirillov A, Mintun E, Ravi N, Mao H, Rolland C, Gustafson L, et al.. Segment Anything. *2023 IEEE/CVF International Conference on Computer Vision (ICCV)*.

42. Sekachev B, Manovich N, Zhiltsov M, Zhavoronkov A, Kalinin D, Hoff B, et al.. opencv/cvat: v1.1.0. Zenodo;

43. Akyon FC, Onur Altinuc S, Temizel A. Slicing Aided Hyper Inference and Fine-Tuning for Small Object Detection. *2022 IEEE International Conference on Image Processing (ICIP)*.

44. : OpenCV: Camera Calibration. https://docs.opencv.org/4.x/dc/dbb/tutorial_py_calibration.html (2025). Accessed 2025 Jan 31.

45. Pereira A, Santos C, Aguiar M, Welfer D, Dias M, Ribeiro M, et al.. Detection of retinal microlesions through YOLOR-CSP architecture and image slicing with the SAHI algorithm. *2023 International Joint Conference on Neural Networks (IJCNN)*.

46. Saradopoulos I, Potamitis I, Rigakis I, Konstantaras A, Barbounakis IS. Image Augmentation Using Both Background Extraction and the SAHI Approach in the Context of Vision-Based Insect

Localization and Counting. *Information*. Multidisciplinary Digital Publishing Institute; 2025; doi: 10.3390/info16010010.

47. Redmon J, Divvala S, Girshick R, Farhadi A. You Only Look Once: Unified, Real-Time Object Detection. *2016 IEEE Conference on Computer Vision and Pattern Recognition (CVPR)*.

48. : Train - Ultralytics YOLO Docs. https://docs.ultralytics.com/es/modes/train/ (2025). Accessed 2025 Jan 31.

49. Bradski G, Kaehler A. OpenCV. *Dr Dobb's journal of software tools*. 32000;

50. : OpenCV: Image Segmentation with Watershed Algorithm. https://docs.opencv.org/4.x/d3/db4/tutorial_py_watershed.html (2025). Accessed 2025 Jan 31.

51. Bonhomme V, Picq S, Gaucherel C, Claude J. Momocs: Outline Analysis Using R. *Journal of Statistical Software*. 2014; doi: 10.18637/jss.v056.i13.

52. Seabold S, Perktold J. Statsmodels: Econometric and Statistical Modeling with Python. *scipy*. 2010; doi: 10.25080/Majora-92bf1922-011.

53. Ultralytics. YOLO Pre-trained Segmentation Models. https://docs.ultralytics.com/es/tasks/segment/#models;

54. Demir B, Sayıncı B, Çetin N, Yaman M, Çömlek R, Aydın Y, et al.. Elliptic Fourier based analysis and multivariate approaches for size and shape distinctions of walnut (Juglans regia L.) cultivars. *Grasas y Aceites*. 2018; doi: 10.3989/gya.0104181.

55. Fernández i Martí A, Font i Forcada C, Socias i Company R. Genetic analysis for physical nut traits in almond. *Tree Genetics & Genomes*. 2013; doi: 10.1007/s11295-012-0566-8.

56. Forcada CF i, Oraguzie N, Reyes-Chin-Wo S, Espiau MT, Company RS i, Martí AF i. Identification of Genetic Loci Associated with Quality Traits in Almond via Association Mapping. *PLOS ONE*. Public Library of Science; 2015; doi: 10.1371/journal.pone.0127656.

57. Goonetilleke SN, Wirthensohn MG, Mather DE. Genetic analysis of quantitative variation in almond nut traits. *Tree Genetics & Genomes*. 2023; doi: 10.1007/s11295-023-01630-w.

58. Sánchez-Beeckman M, Fornés Comas J, Martorell O, Alonso Segura JM, Buades A. Three-dimensional image analysis for almond endocarp feature extraction and shape description. *Computers and Electronics in Agriculture*. 2024; doi: 10.1016/j.compag.2024.109420.

59. Sorkheh K, Shiran B, Khodambashi M, Moradi H, Gradziel TM, Martínez-Gómez P. Correlations between quantitative tree and fruit almond traits and their implications for breeding. *Scientia Horticulturae*. 2010; doi: 10.1016/j.scienta.2010.04.014.

60. Feldmann M J, Hardigan M A, Famula R A, López C M, Tabb A, Cole G S, et al.. Supporting data for "Multi-Dimensional Machine Learning Approaches for Fruit Shape Phenotyping in Strawberry." GigaScience Database;

61. Feldmann MJ. Classification and Quantification of Strawberry Fruit Shape. 2019; doi: 10.5281/zenodo.3365714.

62. Keller B, Jung M, Bühlmann-Schütz S, Hodel M, Studer B, Broggini G, et al.. Dataset - The genetic basis of apple shape and size unraveled by digital phenotyping. ETH Zurich;

63. Utai K, Nagle M, Hämmerle S, Spreer W, Mahayothee B, Müller J. Mass estimation of mango fruits (Mangifera indica L., cv. 'Nam Dokmai') by linking image processing and artificial neural network. *Engineering in Agriculture, Environment and Food*. 2019; doi: 10.1016/j.eaef.2018.10.003.

64. Chitwood DH, Kumar R, Headland LR, Ranjan A, Covington MF, Ichihashi Y, et al.. A Quantitative Genetic Basis for Leaf Morphology in a Set of Precisely Defined Tomato Introgression Lines. *The Plant Cell*. 2013; doi: 10.1105/tpc.113.112391.

65. Migicovsky Z, Li M, Chitwood DH, Myles S. Morphometrics Reveals Complex and Heritable Apple Leaf Shapes. *Front Plant Sci*. Frontiers; 2018; doi: 10.3389/fpls.2017.02185.

66. Wang H, Yin H, Li H, Wu G, Guo W, Qi K, et al.. Quantitative 2D fruit shape analysis of a wide range of pear genetic resources toward shape design breeding. *Scientia Horticulturae*. 2024; doi: 10.1016/j.scienta.2023.112826.

67. Zhao K, Wu X, Xiao Y, Jiang S, Yu P, Wang Y, et al.. PlanText: Gradually Masked Guidance to Align Image Phenotypes with Trait Descriptions for Plant Disease Texts. *Plant Phenomics*. AAAS; 6:02722024;

68. Mas-Gómez J, Rubio M, Dicenta F, Martínez-García PJ. Dataset AlmondCV Pictures. 2025; doi: 10.5281/zenodo.15423562.

Figure_1

Click here to access/download;Figure;Figure1.png

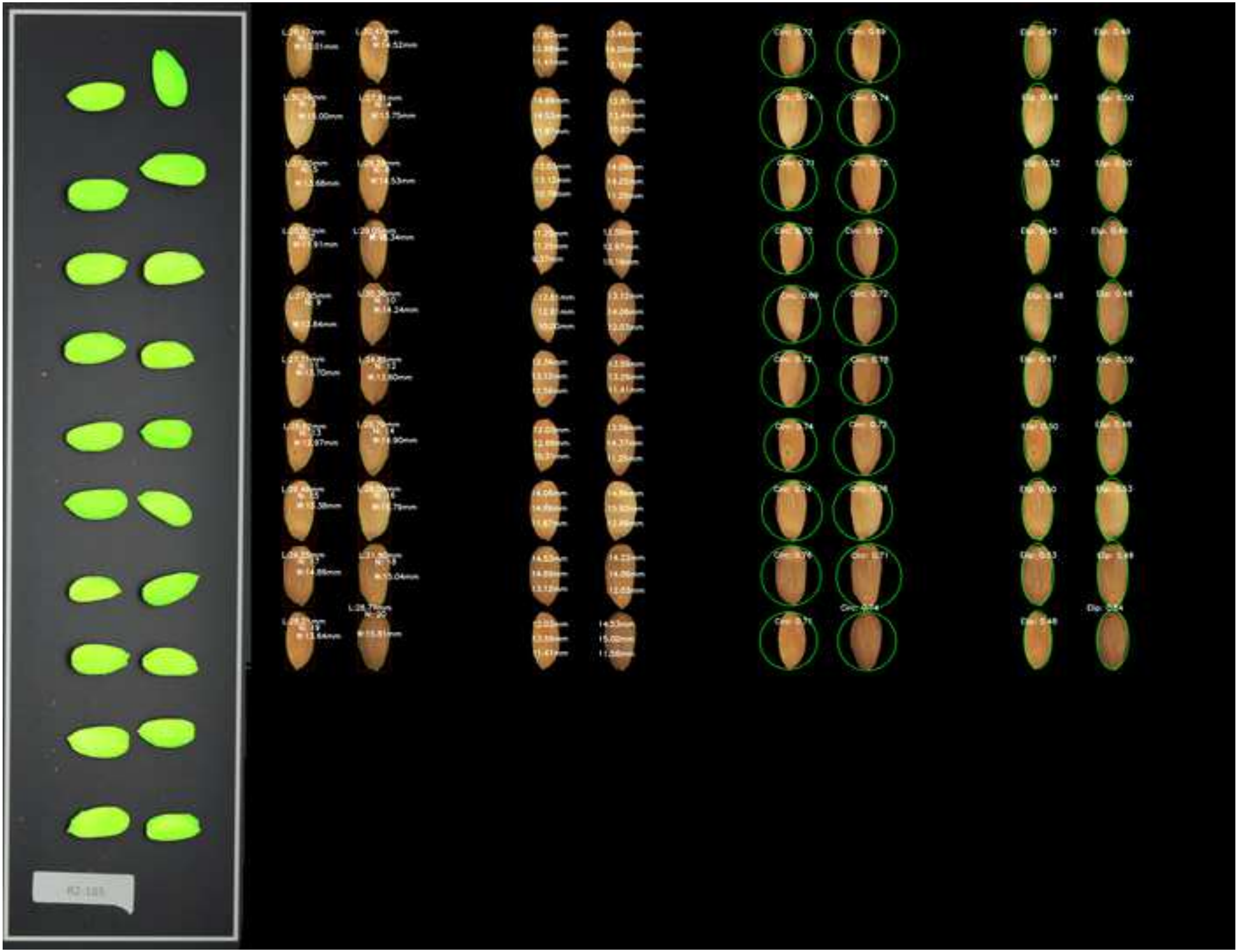

Figure_2A Click here to access/download;Figure;Figure2A.png

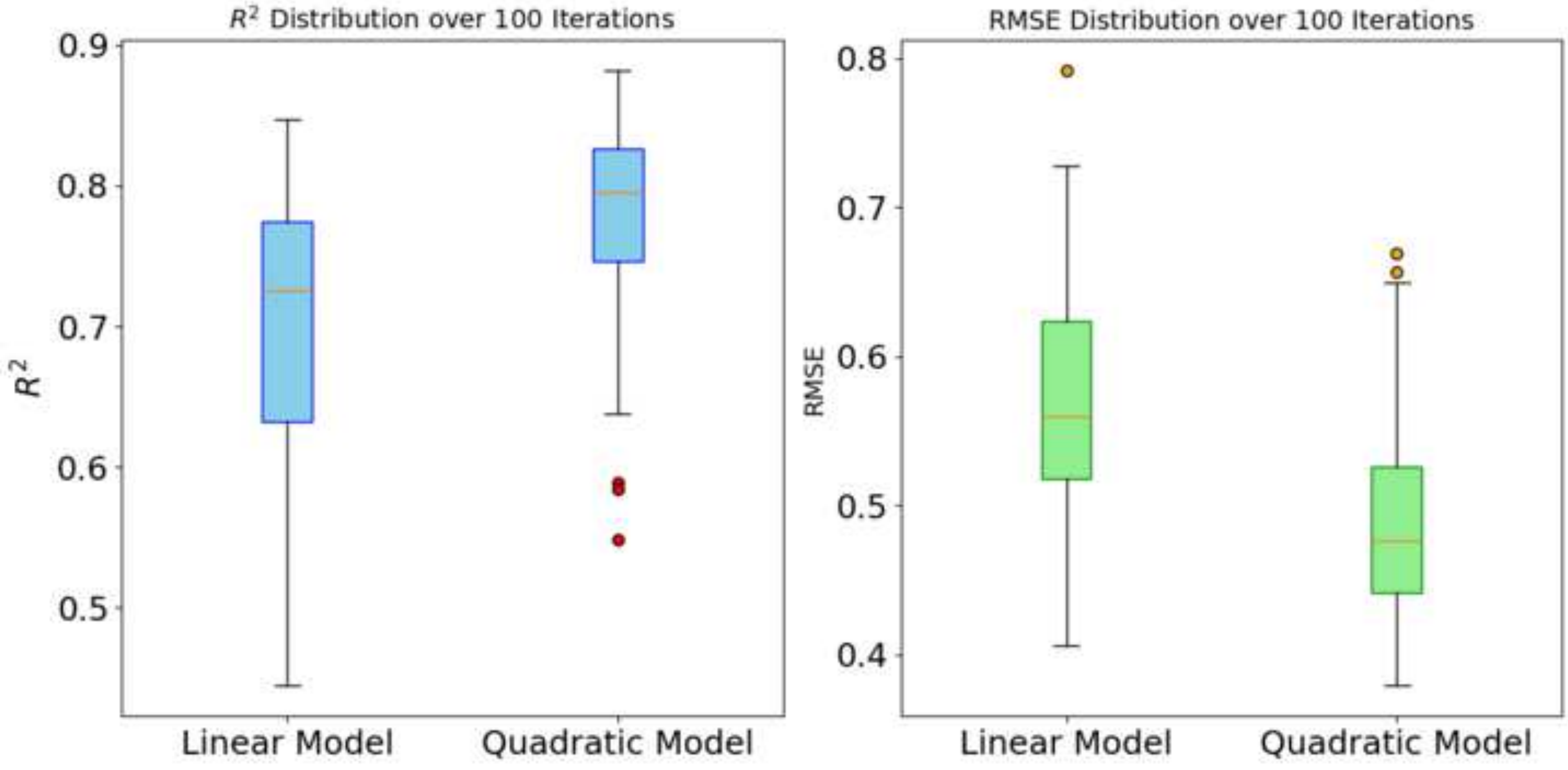

Figure_2B

Click here to access/download;Figure;Figure2B.png

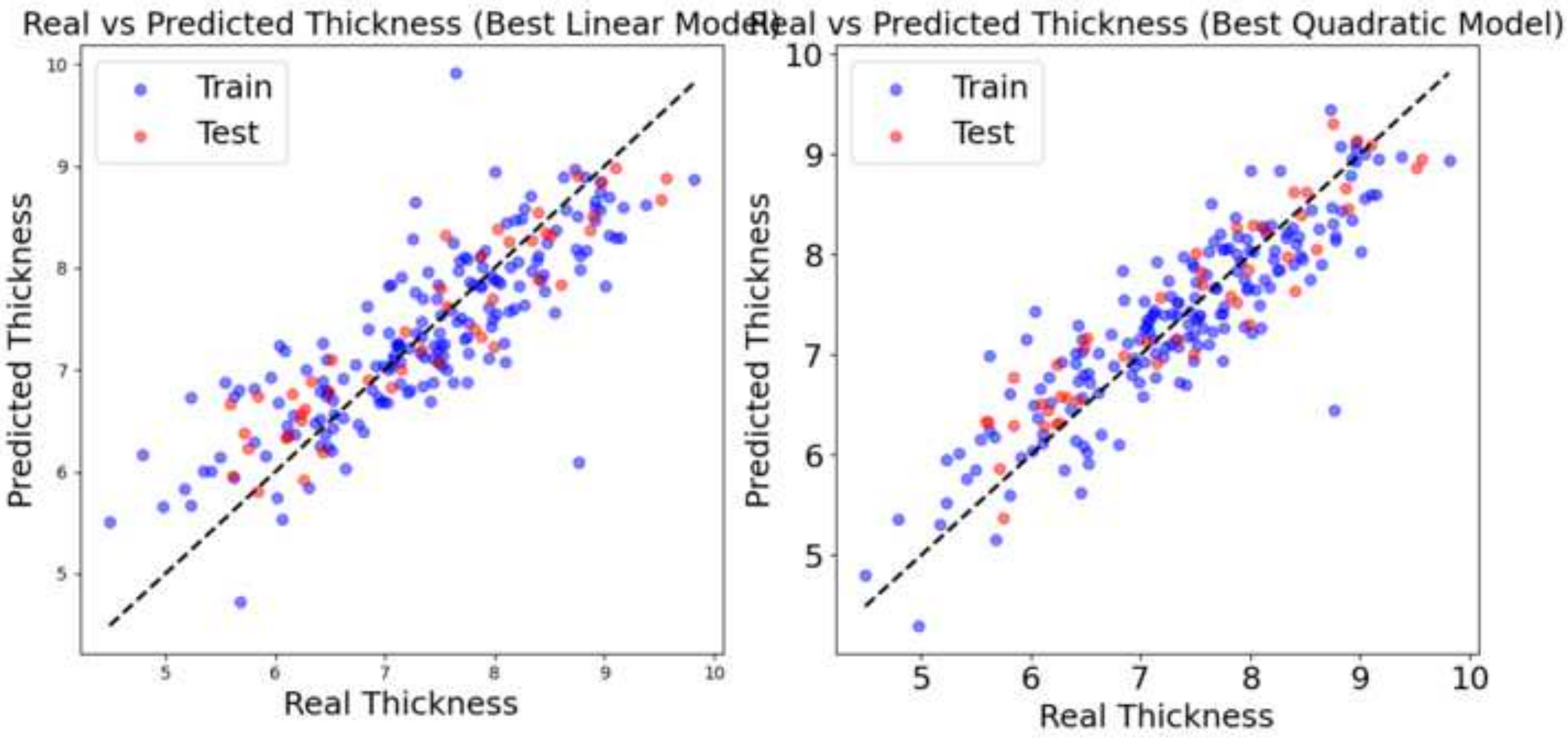

Figure_3

Click here to access/download;Figure;Figure3.jpg

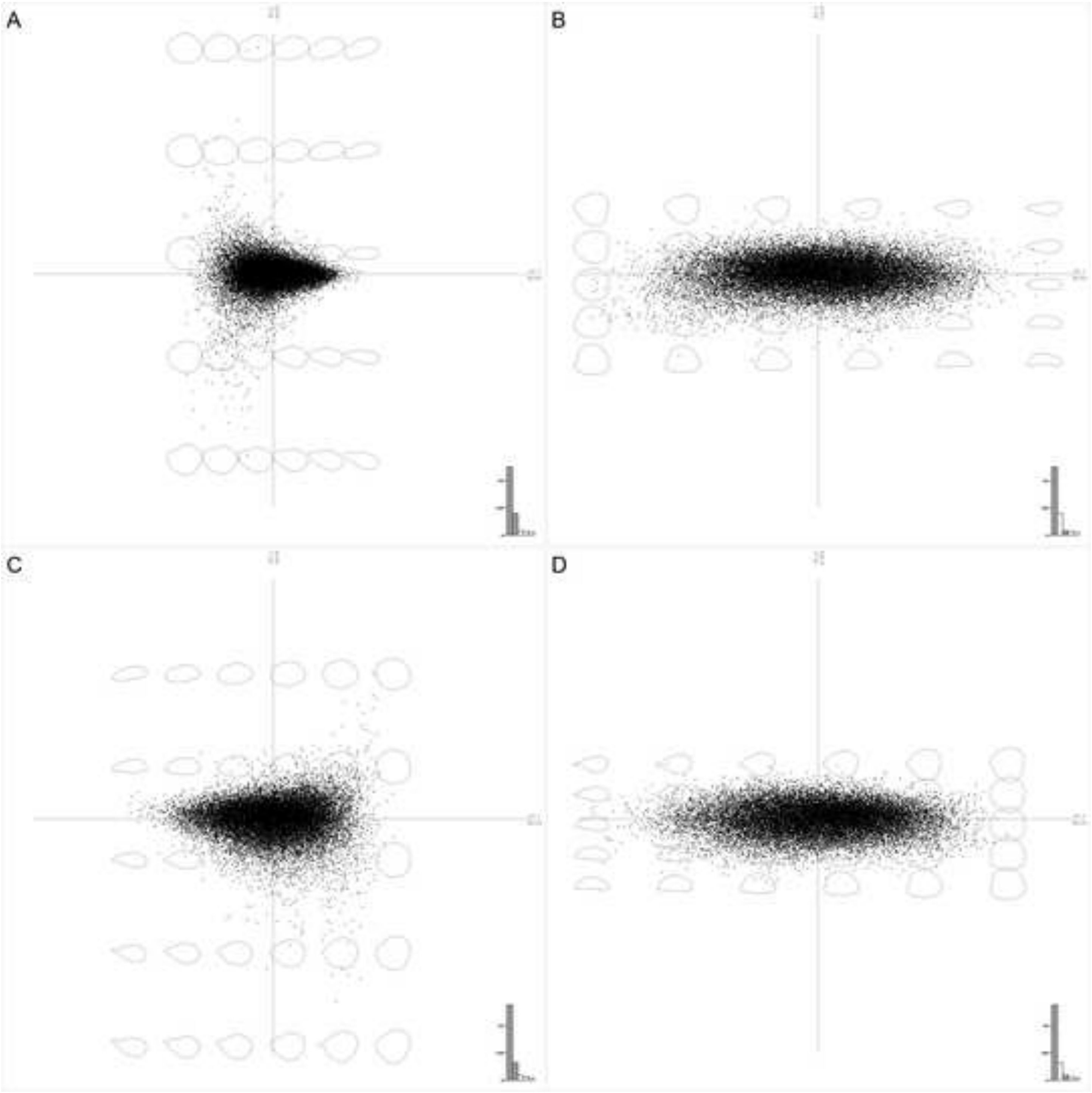

Figure_4

Click here to access/download;Figure;Figure4.jpg

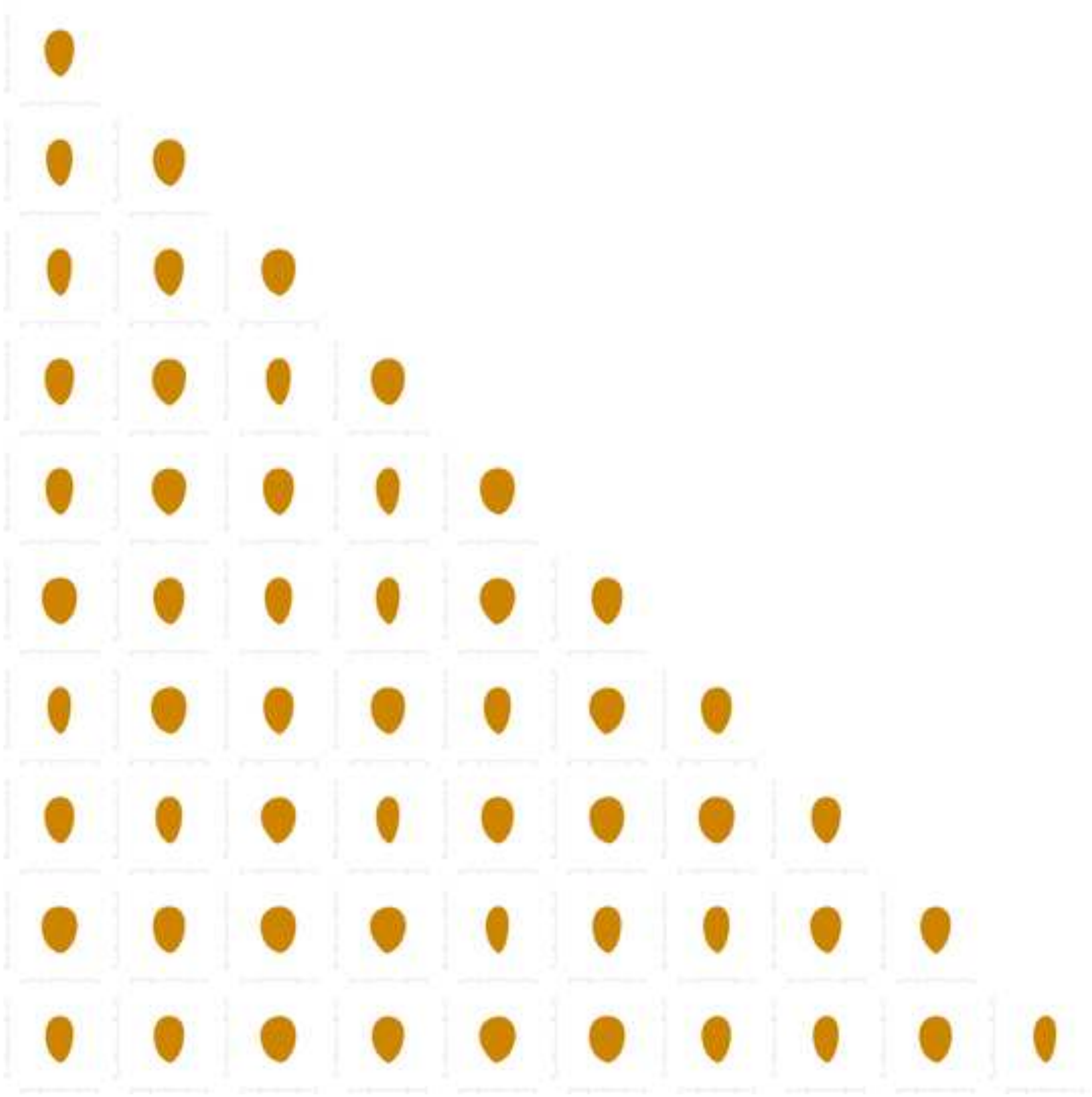

Figure_5

Click here to access/download;Figure;Figure5.png

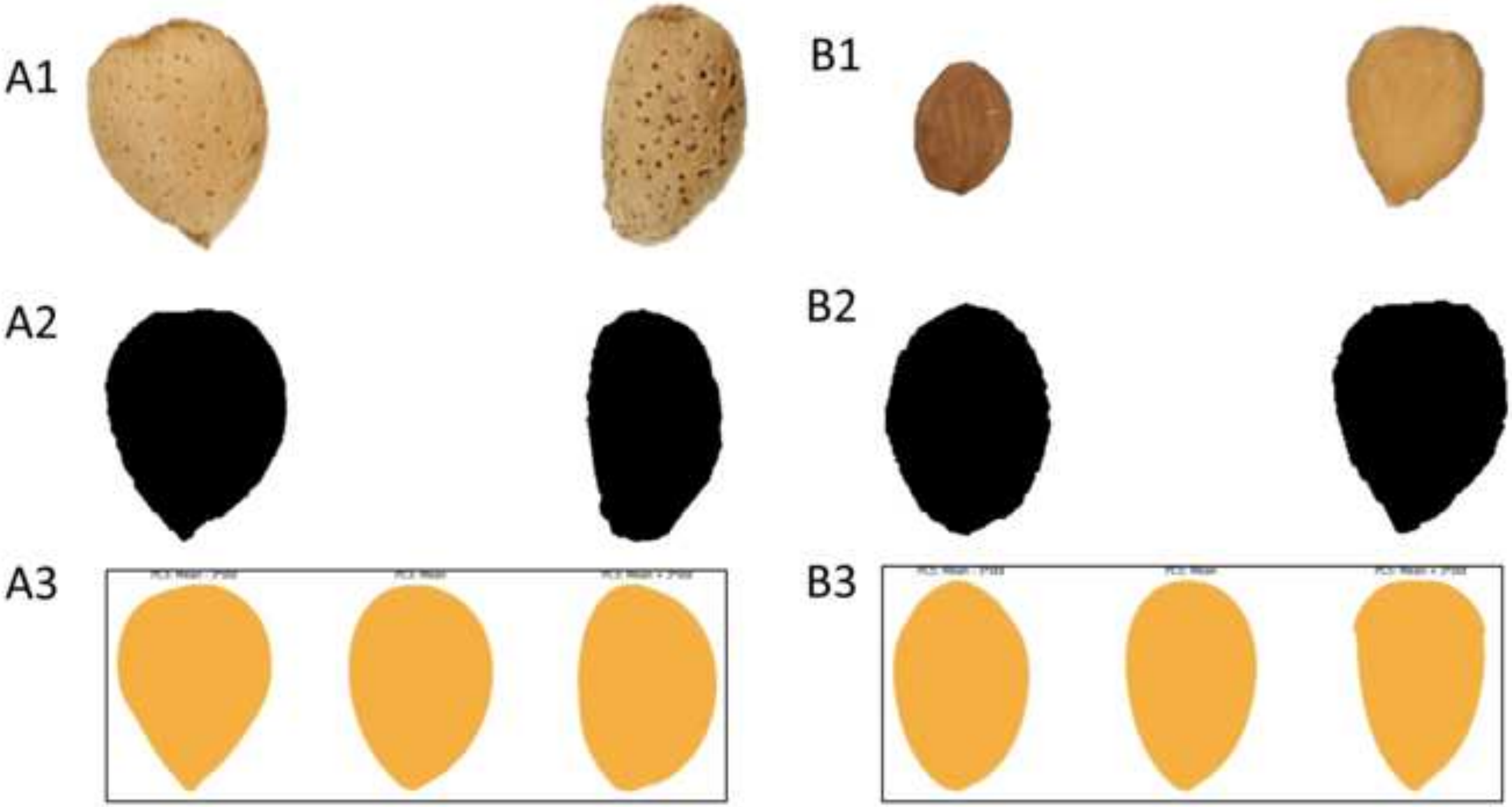

Figure_6

Click here to access/download;Figure;Figure6.jpg

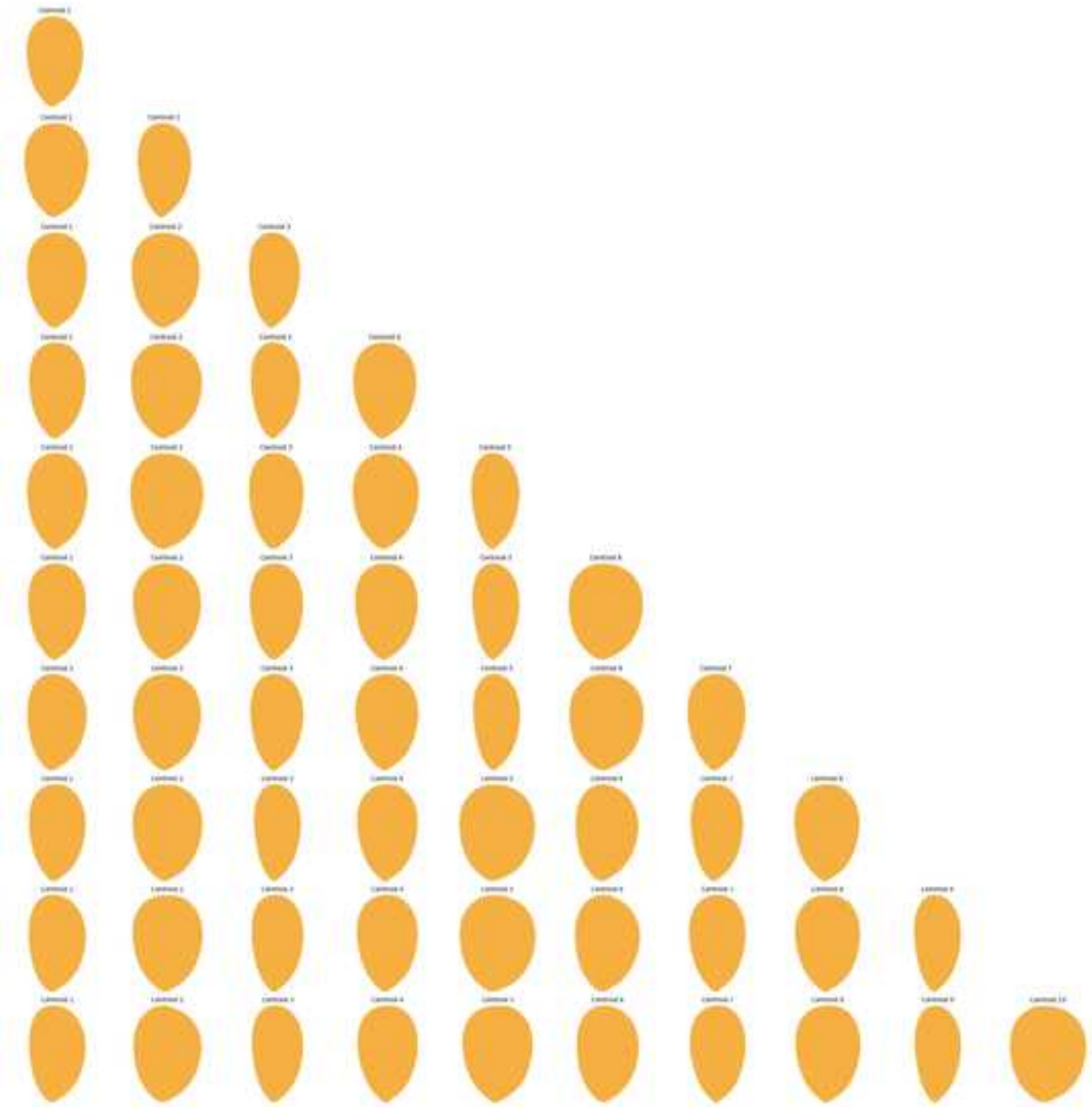

Figure_7

Click here to access/download;Figure;Figure7.png

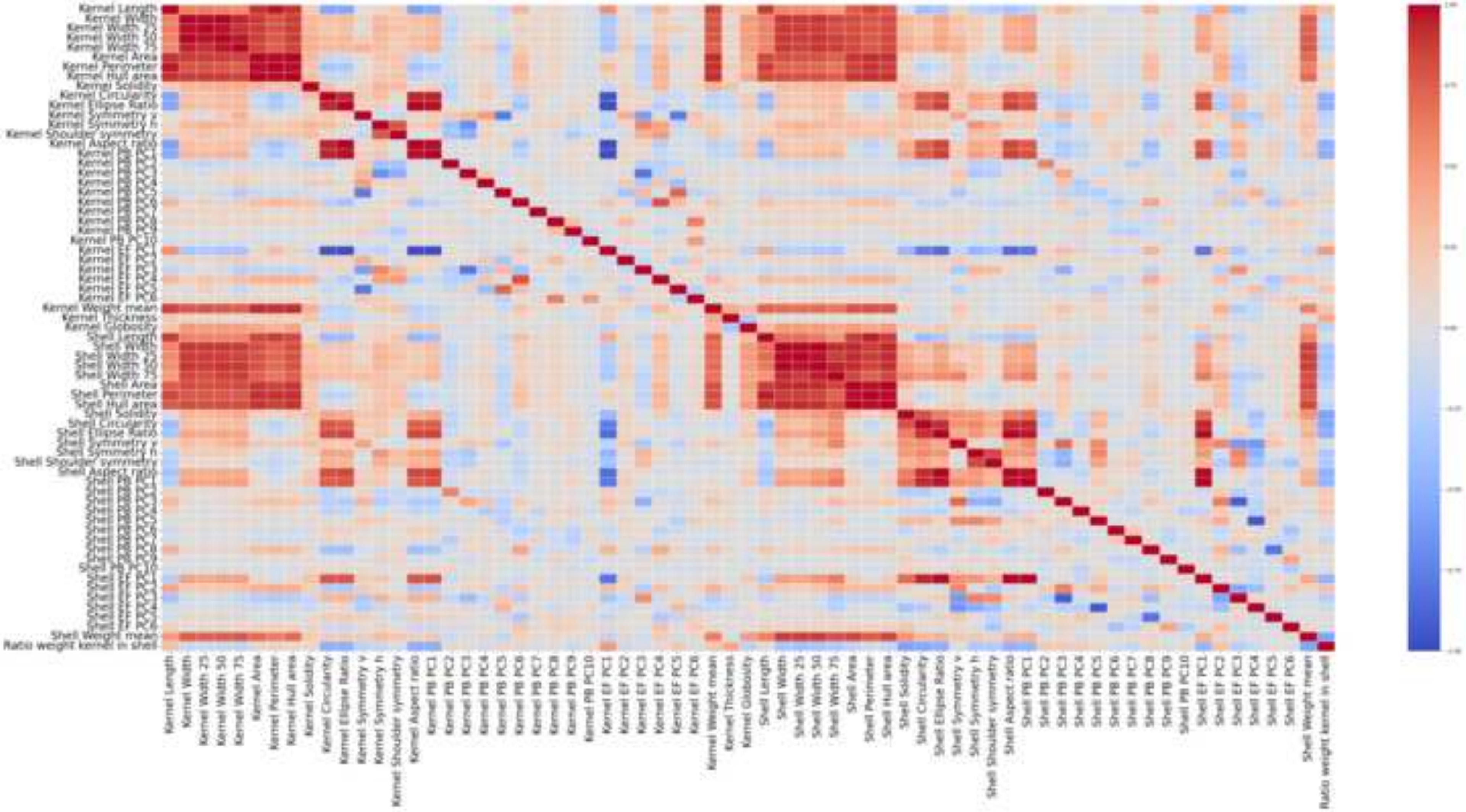

Figure_8

Click here to access/download;Figure;Figure8.png

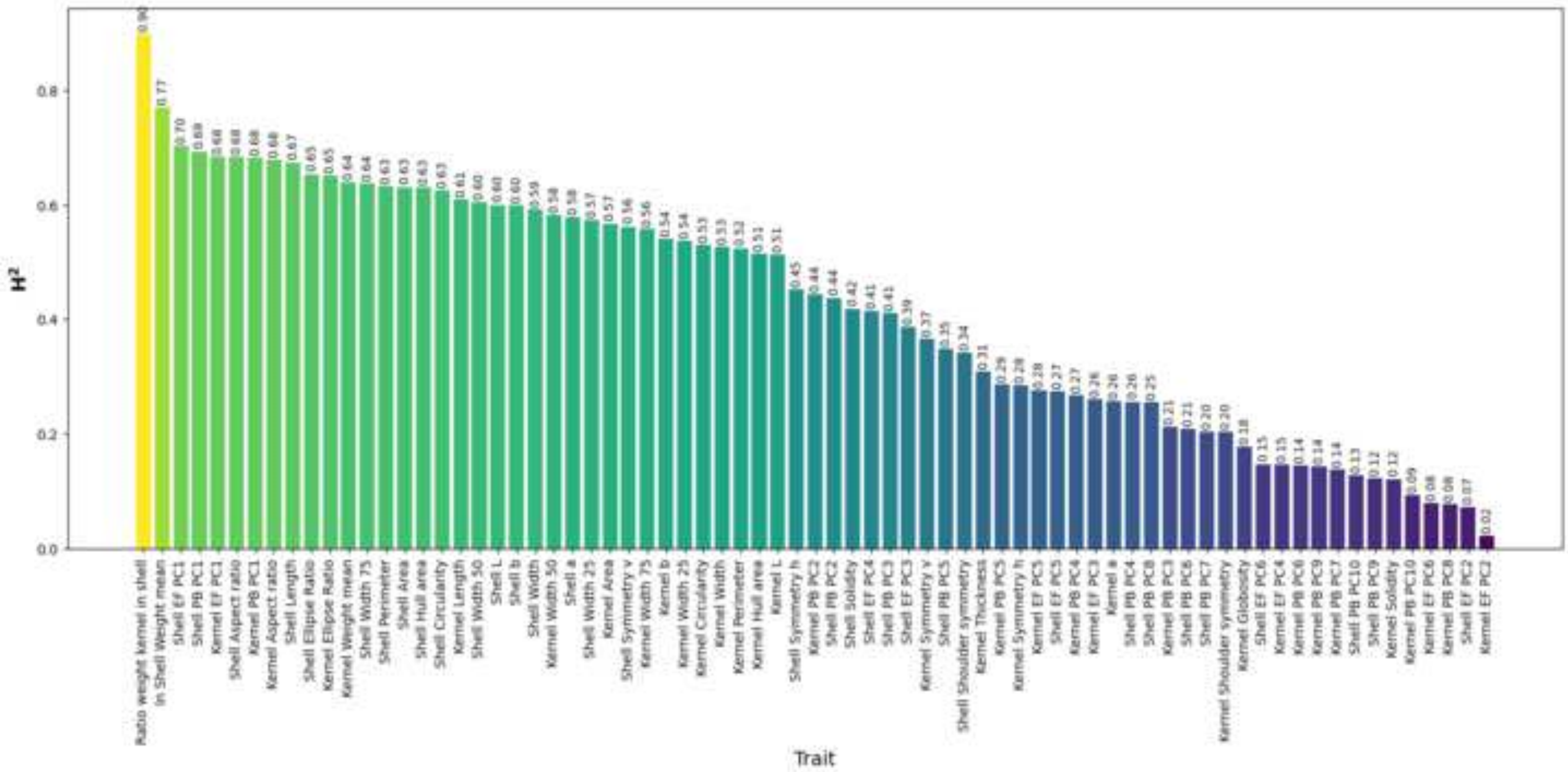

Figure_9

Click here to access/download;Figure;Figure9.png

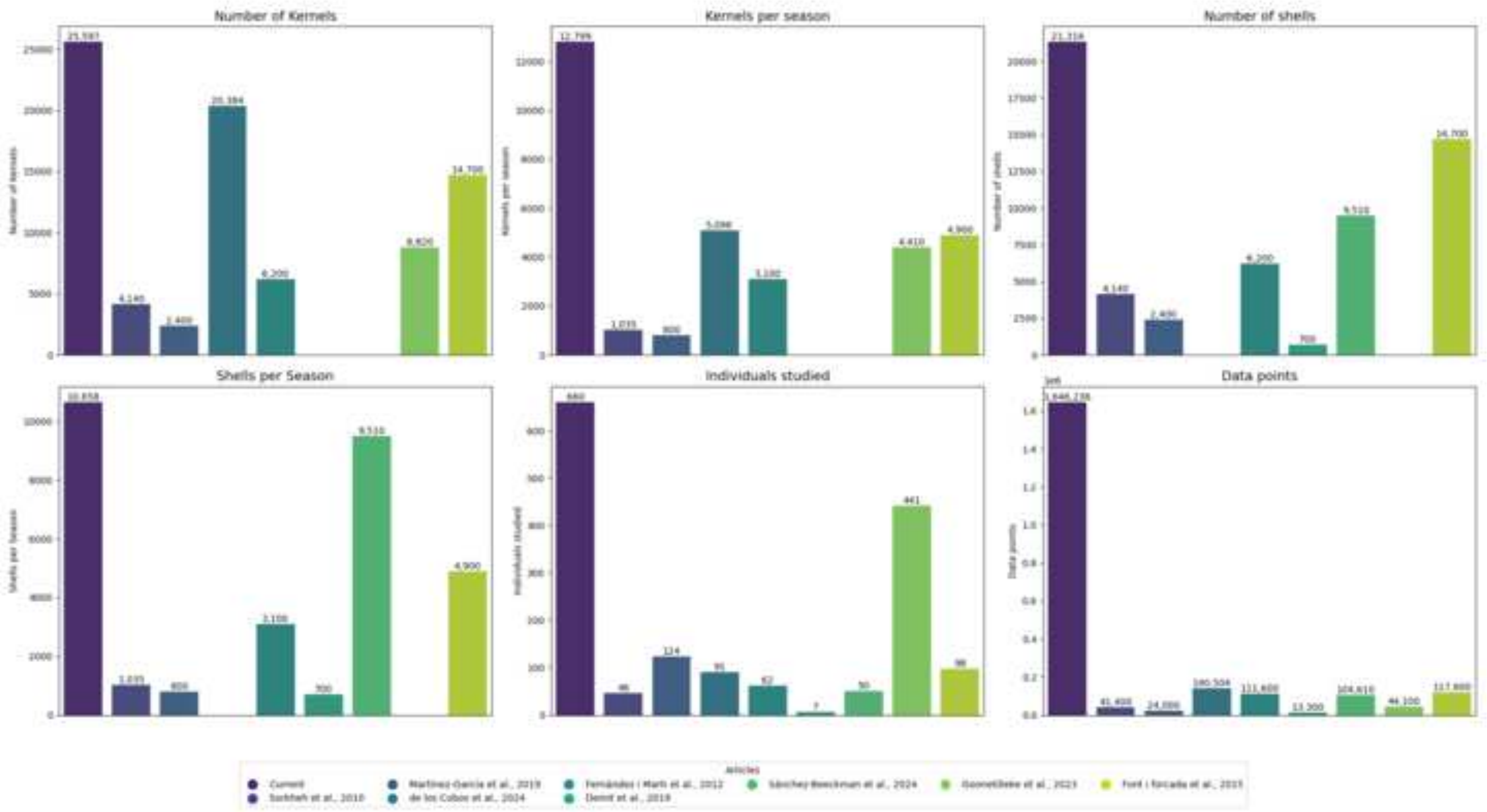

Supplementary Figures

Click here to access/download
Supplementary Material
Supplemmentary_Figures.docx

Supplementary Tables

Click here to access/download
Supplementary Material
Supplementary_tables.xlsx

Personal Cover Click here to access/download;Personal Cover;Cover_letter_Gigascience_R1.docx

20/08/2025

Dear Editor-

Thank you for allowing us to submit a revised version of our manuscript ID GIGA-D-25-00218 entitled now "*Open RGB Imaging Workflow for Morphological and Morphometric Analysis of Fruits using deep learning: A Case Study on Almonds*." We have addressed the reviewer's comments in a revised manuscript.

We are pleased that suggestions from reviewers have contributed to improve our manuscript. A detailed point-by-point response to comments from each reviewer have been submitted.

Thank you for taking the time to review our manuscript and don't hesitate to contact me with further questions.

Yours sincerely,

Pedro José Martínez-García
Tenured Scientist
Department of Plant Breeding
Centro de Edafología y Biología Aplicada del Segura (CEBAS)
Spanish National Research Council (CSIC)

Dear Editor and Reviewers,

We sincerely appreciate your contributions and comments and proceed to address your suggestions point-by-point in this document. Responses are in red color.

Editor reports:

We would like to confirm that the new software has been registered in the bio.tools and SciCrunch.org databases and a RRID (Research Resource Identification Initiative ID) and biotoolsID identifiers were received, and it is now included in the manuscript. Also a new title has been provided according to reviewer#1 comments: "Open RGB Imaging Workflow for Morphological and Morphometric Analysis of Fruits using deep learning: A Case Study on Almonds."

Reviewer reports:

Reviewer #1: The methods described in this article represent a useful tool for fast and reliable morphometric analysis of almonds with potential applications in fruits. The pipeline is technically sound, and publicly available workflow will advance the adoption of this technology. However, there are critical concerns which needs to be addressed before the manuscript could be further proceeded for publication in the journal –

Response: Thank you for your comments.

Major Comments -

1. Authors claim this technique as a new phenotyping tool with breakthrough implications; however, I object to this claim. Numerous studies have utilized this technique in plant phenotyping to the extent that labeling it as a new phenotyping tool may not be ideal. Additionally, for kernel or seed morphometrics, a wide array of user-friendly, open-source tools have already been developed and are readily available, for example,

- SeedExtractor https://www.frontiersin.org/journals/plant-science/articles/10.3389/fpls.2020.581546/full#h3
- SmartGrain https://academic.oup.com/plphys/article/160/4/1871/6109568
- GrainScan https://link.springer.com/article/10.1186/1746-4811-10-23
- PlantCV https://peerj.com/articles/4088/

These tools and a lot of other options can be readily used for almond kernel morphometrics. Authors are requested to discuss/compare advantages/performance of their model with SeedExtractor, SmartGrain, and GrainScan.

Response: Thank you for your comments. It is clear that the mentioned software are valuable and useful tools for high-throughput phenotyping. However, to better highlight the advantages of our phenotyping workflows over these tools, we have summarized them in the following table.

Key point	AlmondCV	SeedExtractor	SmartGrain	GrainScan	PlantCV
Open-Source	Yes	Yes, for standalone version	No	No	Yes
Availability for all operative systems	Yes	Yes	No	No	Yes
Language	Python mainly. Complements in R	Matlab	Visual C++	NA	Python
Online run	Yes	No	No	No	Yes
Color measure	Yes	Yes	NA	Yes	Available functions
Morphology measures	Length, width, area, perimeter, hull-area, solidity, aspect ratio, circularity, ellipse ratio, colour (L*a*b* model), width at three different heights (25, 50 and 75% of the length), vertical and horizontal symmetry and shoulder symmetry, estimation of the thickness, globosity	area, perimeter,length, width), circularity, seed number, color intensity	Area, perimeter length, circularity, length, width, length-to-width ratio, intersection of length and width, center of gravity,	Area, length, width, color	area, convex_hull_area, solidity, perimeter, width, height, longest_path, center_of_mass, convex_hull_vertices, object_in_frame, ellipse_center, ellipse_major_axis, ellipse_minor_axis, ellipse_angle, ellipse_eccentricity, color
Morphometric analyses	Elliptical Fourier and Pixel-Based PCA	NA	No	NA	Landmarks
Camera and color calibration	Yes	NA	NA	Yes	Available functions
Segmentation type	Deep Learning	Thresholding	Thresholding	Thresholdin g	Thresholding, Naive Bayes, Edge detection,k-means
Contact between elements solution	SAHI and Watershed	NA	NA	Watershed	Watershed
Workflow Integration	Yes	Yes, but without calibration, morphometrics.	Yes, but without calibration, morphometr ics.	Yes, without morphometr ics	Independent workflows, integrated in some cases.

Regarding the key points: (1) our workflow is open source, enabling customization and further development for future studies and applications, as already demonstrated in the manuscript

with other fruits; (2) it can run on any operating system with Python installed; (3) together with PlantCV, it is the only one among those mentioned written in Python—a language more widely used than MATLAB, with greater availability of learning resources, generally easier to adopt, and therefore more accessible for a broader range of breeding programs; (4) it can also run online using free and widely adopted resources such as Google Colab; (5) not all of the mentioned tools can analyze color; (6) not all provide the complete set of morphological measurements offered here; (7) none integrate both elliptical Fourier analyses and pixel-based PCA for morphometric analyses; (8) AlmondCV includes functions for camera and color calibration, which are not available in all of them; (9) for segmentation, AlmondCV introduces a novel approach not present in the others, using deep learning models and allowing easy development of a custom segmentation model, whereas the rest mainly rely on thresholding, which, as noted in the SeedExtractor paper and confirmed by our experience, often requires manual parameter adjustment: "Due to a wide range of variation in seed size and color, it is difficult to automatically set optimal size and color ranges (for all the color spaces)," a limitation that can be critical in breeding programs with high phenotypic diversity; additionally, the SeedExtractor paper states (10): "The application requires that the seeds are not touching each other when imaged," an issue we also encountered and addressed through a watershed algorithm similar to GrainScan (but here open source) and a novel method using SAHI that separates elements in their respective functions; and (10) our workflow integrates multiple components—camera calibration, image segmentation, and morphological/morphometric trait extraction—that are not fully combined in any of the other tools mentioned.

This has been explained in the introduction to provide clarity for readers

The term 'breakthrough' refers to the phenotyping bottleneck in almond breeding, which our study surpasses relative to previous research and current breeding practices. We have, however, reformulated the sentence to improve clarity

2. Within horticultural crops, workflows and studies (not acknowledged in this article) are available that can be adapted or modified to do the same thing. For example - publications from as early as 2020 used machine learning models to measure size and mass of almonds, however, this relevant study was not acknowledged by the authors https://onlinelibrary.wiley.com/doi/full/10.1111/jfpe.13374 . Discuss how the presented method is better than the aforementioned article, justify the claim 'breakthrough'.

Response: Thank you for your comments. Regarding the use of the term 'breakthrough,' as mentioned earlier, we refer to the phenotyping bottleneck in almond breeding, which is justified by comparison with previous studies and current phenotyping practices in almond breeding programs. With respect to the advantages over the mentioned article, many of the

points noted in our previous comment also apply (e.g., thresholding instead of deep learning, lack of open-source availability, unspecified programming language, unknown compatibility with different operating systems, measurement limited to length, and no calibration procedures). Moreover, that study analyzed only a single variety (NonPareil), whereas our workflow phenotypes more than 600 genotypes, capturing much greater diversity and providing a broader test. In addition, the previous study focuses primarily on industrial applications, whereas our approach emphasizes the accuracy and diversity required in breeding programs. We have included this context in the introduction to highlight the use of imaging in almonds.

3. The authors claim successfully testing the pipeline for apples and strawberries. Information for fruit size can be extracted from 2D images; however, the example results show only length, width, circularity, and ellipse ratio. How do these parameters assist fruit breeders? Since it is segmentation based classification using a reference scale, the aforementioned tools particularly SeedExtractor can generate similar results. Does it qualify the tool for integration into fruit crops breeding pipeline? Moreover, fruit breeders require on-tree analysis, recent advancements have enabled 3D sensing for significantly better detection particularly using cost effective RGB-D cameras.

Response: Thank you for your comments. In this study, we only test the workflow on the specified species, and the traits measured are all described in the methodology section for the general method (length, width, area, perimeter, hull area, solidity, aspect ratio, circularity, ellipse ratio, and colour using the Lab* model). Additionally, when fruits are placed in an ordered and flat orientation, the same traits as for almonds can be measured (e.g., width at three different heights: 25%, 50%, and 75% of the length, vertical and horizontal symmetry). In the manuscript, we propose that our workflow could be expanded to other fruits and allow the development of crop-specific traits using the existing functions as a base/template, as illustrated with simple examples in apple and strawberry.

In addition, almond shape has been traditionally evaluated subjectively and is time-consuming (Martinez-Garcia et al 2019). By definition, however, morphological traits are highly dimensional (Zingaretii et al 2021). Computing only linear, univariate phenotype leads to a loss of information by extremely simplifying the features of a shape (Klingenberg 2010, Claes 2014). The use of geometric-morphometric approaches for shape analysis is warranted (Dryden 1998). From a breeding point of view, our advancement in high-throughput phenotyping can generate more precise phenotypic datasets that provide valuable information for understanding the genetic basis of our traits, can help to a better estimation of heritability and to improve predictive accuracy in strategies such as Genomic Selection. The integration of cutting-edge technologies like GS and HTP has the potential to accelerate gains in breeding.

Regarding differences with SeedExtractor, as mentioned in our first comment, SeedExtractor does not allow, for instance, the extraction of morphometric traits via elliptical Fourier analyses or pixel-based PCA. As noted in the introduction, these approaches have been applied in apple and strawberry breeding for target traits, providing examples of how such traits can assist fruit breeders (Feldmann et al., 2020; Keller et al., 2024).

Regarding 3D fruit phenotyping in the field, we agree that it represents a promising area for breeding. However, it typically requires more hardware and software resources, which can complicate implementation and limit adoption by breeding programs. A clear example is Li et al. (2025), who developed a sophisticated autonomous rover to phenotype traits such as fruit number, maturity, and yield in blueberry. Moreover, field phenotyping may not be equally effective for all species; for instance, almonds present additional challenges due to their shells, tree architecture, and the dispersed bearing of fruits, which is more complex than in blueberry bushes. Additionally, measurement accuracy for morphological fruit traits would generally be lower in the field than under controlled conditions, which can be critical for breeding schemes.

Minor Comments -

1. Change title - This study uses deep learning models which are a type of AI. AI is a broader term. Additionally, the potential for utility in fruit breeding pipeline appears to be limited. Suggested - Open RGB imaging workflow for morphological and morphometric analysis of almond kernels using deep learning.

Response: Thank you for your comments. We agree that "deep learning" is more precise than the broader term "AI." However, we respectfully disagree with the assessment that the workflow has limited utility in fruit breeding pipelines. Our workflow provides tools that are relevant to breeding programs, as demonstrated in species such as strawberry and apple (Feldmann et al., 2020; Keller et al., 2024), while also incorporating new advances and features that enhance phenotyping capabilities.

2. The video tutorial showed using YOLOv8, mention it in the methods. Add information for all settings used in CVAT.

Response: Thank you for your comments. We have included it in the "Develop your segmentation model" section.

Reviewer #2: We would like to thank you for submitting your manuscript to our journal.The manuscript proposes an AI-powered open RGB imaging workflow for morphological and morphometric analysis of fruits or other plant organs, with almonds as a case study. The workflow, developed in Python, covers the full pipeline from image pre-processing, segmentation model development and deployment, to trait measurement and analysis. It aims to improve the efficiency and accuracy of phenotyping in breeding programs by

addressing the limitations of traditional methods, such as their time-consuming and labor-intensive nature.

Response: Thank you for your comments.

However, there are still the following problems in this paper that need further improvement:

1.Table formatting: Some of the tables in the manuscript do not follow the formatting standards of the journal. The authors are encouraged to revise them accordingly to ensure clarity, consistency, and ease of understanding.

Response: Thank you for your comments. We have followed the table preparation guidelines described in the Instructions to Authors (https://academic.oup.com/gigascience/pages/instructions_to_authors#Preparing%20Tables), including the requirement that all tables are introduced in the text, properly formatted, and have titles with fewer than 15 words. However, we are not certain which specific formatting aspect may not meet the journal's standards. We would greatly appreciate further clarification so we can make the necessary adjustments to ensure full compliance.

2.Formula presentation: Certain mathematical formulas are not clearly formatted and appear disorganized. The authors should re-typeset the equations to improve readability and provide clearer explanations for each formula.

Response: Thank you for your comments. In our original Word document, the formulas appear correctly formatted. We suspect that the conversion process during submission may have altered their layout. We will recheck the typesetting in the submitted version and re-typeset the equations to ensure they are properly aligned and readable.

3.Introduction: The introduction could be strengthened by more thoroughly explaining the relationship between phenotypic data and breeding. The authors may also discuss how phenotyping data supports genomic selection and accelerates breeding via high-throughput workflows.

Response: Thank you for your comments. We have reinforced the introduction to clarify the importance of phenotyping in breeding and its impact in genomic selection.

4.Methods section: While the paper clearly explains how morphological traits and kernel thickness are measured, it does not sufficiently explain how this data contributes to breeding decisions. The authors should elaborate on how the extracted traits are applied in practical breeding or selection strategies.

Response: Thank you for your comments. We have reinforced the discussion to clarify the importance how can be incorporated in breeding approaches.

5.Lack of algorithmic novelty: While the integration of existing tools is commendable, the core methods used (e.g., YOLO, SAHI) are based on publicly available models, without introducing new algorithmic components or comparative ablation studies. The authors are advised to clarify the unique contribution of their workflow, especially in terms of engineering integration or practical usability.

Response: Thank you for your comments. We acknowledge that the core methods employed are publicly available, which we also consider a strength of our work due to the use of pre-trained generalist models (Bommasani et al., 2021). Furthermore, we implemented specific adaptations in the SAHI method, as indicated in the Develop your segmentation model section of the methodology, to enable the use of the retina_mask argument, which improves fine-detail accuracy in segmentation. In any case, we have clarified our contribution in terms of integration and practical usability in the manuscript, specifically at the end of the introduction.

Bommasani R, Hudson DA, Adeli E, Altman R, Arora S, Arx S von, et al.2021. On the Opportunities and Risks of Foundation Models. arXiv;

6.Limited evaluation metrics: The performance of segmentation models is only reported using error percentage. The inclusion of standard metrics such as IoU, Precision, Recall, and F1-score would allow for a more comprehensive evaluation and comparison across models (see LLRL methods).

Response: Thank you for your comments. We agree, and indeed these metrics are presented in Supplementary Figure 2, although they were not discussed in detail in the original manuscript. We have now included a more detailed discussion of these results in the Results section ("YOLO fine-tuned segmentation models performance and reconstruction errors").

7.Figures and captions: Currently, figure images and their descriptions are placed separately, which may reduce readability. It is recommended to place figure captions immediately beneath or alongside the figures to enhance the paper's coherence and user-friendliness.

Response: Thank you for your comments. We agree with your observation; however, according to the journal's guidelines, figure files must be submitted separately and not embedded within the manuscript.
https://academic.oup.com/gigascience/pages/instructions_to_authors#Preparing%20Figures

8.Trait extension suggestions: In order to enhance the expressiveness and resolution of phenotypic trait modeling, authors are advised to refer to the relevant research on extracting fine-grained phenotypic features in plant images in recent years. For example, PlanText proposed a progressive visual guidance strategy to help improve the modeling quality of phenotypic traits in images.

Response: Thank you for your comments. We were not aware of this visual guidance strategy, but we agree that it could be particularly useful for the abstract traits discussed in our manuscript. We have now included a brief mention of this possibility in the discussion.

Therefore, I would like to give a "Major Revision" recommendation.

Reviewer #3: The present study entitled "Open RGB Imaging Workflow for Morphological and Morphometric Analysis of Fruits
using AI: A Case Study on Almonds" reported the development of a Python-based image analysis pipeline that extract morphological traits of almond nut shells and kernels. A case study was conducted to use the developed pipeline to analyze breeding populations of 665 genotypes and extract both general morphology traits such as height, length, area, aspect ratio, etc. and specialized traits for almond such as width at three heights, vertical and horizontal symmetry, etc. Further, each nut shell or kernel was weighed, so models were established to use the weight and morphological traits to predict the thickness of each nut shell or kernel. In addition to morphological traits, morphometric (or shape) was extracted for each nut shell or kernel. Clustering analysis was performed on the morphometric traits to identify variability among genotypes. To further validate the efficacy of the extracted traits, broad-sense heritability was calculated and used as a criterion.

The major contribution of this study is the integration of different components (e.g., camera calibration, image segmentation, and morphological/morphometric trait extraction, etc.) as a user-accessible, open-source Python implementation for the plant breeding community, especially for almond breeders. However, there several aspects that could be further improved.

Response: Thank you for your comments.

First, the present study showed the most number of samples that were phenotyped by the proposed pipeline among recent efforts on almond nut shell and/or kernel phenotyping. However, there was no clear evidence to demonstrate direct benefits to ongoing almond breeding. Certain traits (e.g., aspect ratio, tip/top/side curvatures) could be included in a breeding program, but what's the significance of including these traits in breeding

programs. Are they crucial to either improve the productivity, quality, or other management practices or processing practices for the almond industry, especially given breeding context?

Response: Thank you for your comments. We agree that we should provide more information on the benefits of these traits. For this reason, we have added a discussion on this topic in the Introduction.

"These traits directly influence market value and consumer acceptance [25,26]. Larger kernel sizes are generally more economically valuable [24], while smaller sizes are preferred for specific applications, such as chocolate bars [22]. Shape is also important for industrial processes: elongated almonds are preferred for slicing, whereas round almonds are generally more appreciated by consumers [22,23][23]. Geometric parameters also affect the mechanical properties of almonds and other nuts, which is relevant for processing [27,28]. For example, elongated shapes have been shown to result in lower breakage during blanching, while rounder shapes reduce breakage during shelling in Spanish almond industry machinery [29]."

We have also highlighted in the Discussion the concerns regarding breakage during industrial processing and the role that almond nut and kernel shape can play in this context.

Second, the pipeline uses deep learning-based segmentation which is powerful to handle complex background. Based on the limited figures or example images in the GitHub repo, the background is mostly single colored (e.g., white or black) without appearances that may confuse even conventional segmentation, especially if image color is calibrated. Assuming most of the almond nut shell and kernel analyses would be done in a laboratory condition, it is not convincing why conventional segmentation methods may not be preferred if both illumination and camera configuration can be well controlled. Ultimately, the question is whether it is worthy the effort of labeling hundreds of images to fine-tune a deep learning segmentation model compared to a careful hardware-software design to make operation more efficient. Or with the simplified background, vision foundation models such as SAM will be sufficient.

Response: Thank you for your comments. We have carefully considered these aspects. While we initially explored conventional segmentation methods, our experience indicates that deep learning-based segmentation models offer clear advantages for a high-throughput phenotyping system:

1. We observed that due to the large phenotypic diversity in almonds—including variations in color, shape, texture, and size—thresholding techniques and similar approaches often require manual adjustments. We believe this issue is likely to occur in breeding programs of other species with high phenotypic diversity. Such manual interventions hinder automation, whether processing images in real time or during post-processing. Indeed, similar limitations have been reported in other software,

such as SeedExtractor: “Due to a wide range of variation in seed size and color, it is difficult to automatically set optimal size and color ranges (for all the color spaces).”

2. Another aspect highlighted in SeedExtractor, which we also observed when using conventional segmentation, is that “The application requires that the seeds are not touching each other when imaged.” This challenge has been addressed in software such as GrainScan, which implements a watershed algorithm (although it is not open-source). In our work, we applied an open-source watershed approach, and additionally employed another method using SAHI, which effectively separates individual elements for downstream analysis.
3. Additionally, we captured images of multiple groups of almonds placed inside painted rectangles (see Methodology), for which we fine-tuned simple deep learning models to recognize and separate the groups. Using conventional segmentation, we frequently encountered problems when almonds touched the rectangle boundaries or when the contours of the rectangles were not correctly enclosed during processing.
4. With fine-tuned deep learning models, we can relax the strict requirements on hardware and software design, which becomes particularly important when working with the large sample sizes as here. In such scenarios, processing speed can be just as critical as precision during image acquisition for the overall feasibility of the process.
5. For phenotyping processes, minimizing manual adjustments facilitates the adoption of the task by technicians without a computer vision background, which also helps to accelerate the overall workflow.

In response to whether it is worthwhile to label hundreds of images to fine-tune a deep learning segmentation model, we argue that it is. The labeling process has become significantly faster, taking approximately 30 minutes to annotate all images using a combination of CVAT and SAM, as demonstrated here. Regarding the direct use of foundation models, Bommasani et al. (2021) note that foundation models “are intermediary assets; they are unfinished and generally should not be used directly, instead requiring adaptation for specific downstream tasks.” Given that fine-tuning is not time- or cost-intensive, it improves segmentation performance, accuracy, and consistency, while reducing the need for manual supervision.

Bommasani R, Hudson DA, Adeli E, Altman R, Arora S, Arx S von, et al.2021. On the Opportunities and Risks of Foundation Models. arXiv;

SeedExtractor https://www.frontiersin.org/journals/plant-science/articles/10.3389/fpls.2020.581546/full#h3

GrainScan https://link.springer.com/article/10.1186/1746-4811-10-23

Third, in the Introduction section, some technical statements should be revised to make them accurate. For example, image segmentation is a core computer vision task rather than relying on computer vision algorithms. One-stage and two-stage strategies are used to differentiate models for object detection not image segmentation. Further, Faster RCNN is an object detection model and cannot do image segmentation. It is highly recommended that the authors could find a computer science or engineering colleague to proofread the technical statements to ensure the accuracy.

Response: Thank you for your comments. We have revised the Introduction to improve technical accuracy. Regarding the use of "one-stage" and "two-stage" terminology in instance segmentation, we acknowledge that these terms are more commonly associated with object detection. However, we have retained them in our manuscript because they are used in recent literature on instance segmentation (e.g., Bolya et al., 2019; Gu et al., 2022) to describe architectural strategies. We are open to updating the terminology if more precise references or conventions are suggested.

Bolya, D., Zhou, C., Xiao, F., & Lee, Y. J. (2019). Yolact: Real-time instance segmentation. In *Proceedings of the IEEE/CVF international conference on computer vision* (pp. 9157-9166).

Gu, W., Bai, S., & Kong, L. (2022). A review on 2D instance segmentation based on deep neural networks. *Image and Vision Computing*, *120*, 104401.

Last, it is appreciated the authors effort on making an open-source software for the community. However, the dataset can be equally important to advance the scientific discovery and technology development. Is there any plan to make the dataset publicly available to help facilitate the development of additional computer vision algorithms for almond phenotyping?

Response: Thank you for your comments. The dataset was submitted alongside the manuscript to GigaScience and has also been deposited in Zenodo. It is currently available to reviewers (see Data Availability section) and will be made publicly accessible to the scientific community upon publication.

Please also take a moment to check our website at https://www.editorialmanager.com/giga/l.asp?i=218825&l=T02XNJYZ for any additional comments that were saved as attachments. Please note that as GigaScience has a policy of open peer review, you will be able to see the names of the reviewers.
