# Supplementary material for: Open RGB imaging workflow for morphological and morphometric analysis of fruits using deep learning: a case study on almonds
Source: Gigascience. 2025 Dec 19;15:giaf157. doi: 10.1093/gigascience/giaf157 (PMC12970599; doi:10.1093/gigascience/giaf157)
Supplement: giaf157_Authors_Response_To_Reviewer_Comments_original_submission [file giaf157_authors_response_to_reviewer_comments_original_submission.pdf]

Author's Response To Reviewer Comments

Dear Editor and Reviewers,
We sincerely appreciate your contributions and comments and proceed to address your suggestions point-by-point in this document. Responses are in red color.
Editor reports:
We would like to confirm that the new software has been registered in the bio.tools and SciCrunch.org databases and a RRID (Research Resource Identification Initiative ID) and biotoolsID identifiers were received, and it is now included in the manuscript. Also a new title has been provided according to reviewer#1 comments: “Open RGB Imaging Workflow for Morphological and Morphometric Analysis of Fruits using deep learning: A Case Study on Almonds.”

Reviewer reports:

Reviewer #1: The methods described in this article represent a useful tool for fast and reliable morphometric analysis of almonds with potential applications in fruits. The pipeline is technically sound, and publicly available workflow will advance the adoption of this technology. However, there are critical concerns which needs to be addressed before the manuscript could be further proceeded for publication in the journal –
Response: Thank you for your comments.
Major Comments -
1. Authors claim this technique as a new phenotyping tool with breakthrough implications; however, I object to this claim. Numerous studies have utilized this technique in plant phenotyping to the extent that labeling it as a new phenotyping tool may not be ideal. Additionally, for kernel or seed morphometrics, a wide array of user-friendly, open-source tools have already been developed and are readily available, for example,
- SeedExtractor https://www.frontiersin.org/journals/plant-science/articles/10.3389/fpls.2020.581546/full#h3
- SmartGrain https://academic.oup.com/plphys/article/160/4/1871/6109568
- GrainScan https://link.springer.com/article/10.1186/1746-4811-10-23
- PlantCV https://peerj.com/articles/4088/

These tools and a lot of other options can be readily used for almond kernel morphometrics. Authors are requested to discuss/compare advantages/performance of their model with SeedExtractor, SmartGrain, and GrainScan.
Response: Thank you for your comments. It is clear that the mentioned software are valuable and useful tools for high-throughput phenotyping. However, to better highlight the advantages of our phenotyping workflows over these tools, we have summarized them in the following table.
Key point AlmondCV SeedExtractor SmartGrain GrainScan PlantCV
Open-Source Yes Yes, for standalone version No No Yes
Availability for all operative systems Yes Yes No No Yes
Language Python mainly. Complements in R Matlab Visual C++ NA Python
Online run Yes No No No Yes
Color measure Yes Yes NA Yes Available functions
Morphology measures Length, width, area, perimeter, hull-area, solidity, aspect ratio, circularity, ellipse ratio, colour (L*a*b* model), width at three different heights (25, 50 and 75% of the length), vertical and horizontal symmetry and shoulder symmetry, estimation of the thickness, globosity area, perimeter,length, width), circularity, seed number, color intensity Area, perimeter length, circularity, length, width, length-to-width ratio, intersection of length and width, center of gravity, Area, length, width, color area, convex_hull_area, solidity, perimeter, width, height, longest_path, center_of_mass, convex_hull_vertices, object_in_frame, ellipse_center, ellipse_major_axis, ellipse_minor_axis, ellipse_angle, ellipse_eccentricity, color
Morphometric analyses Elliptical Fourier and Pixel-Based PCA NA No NA Landmarks
Camera and color calibration Yes NA NA Yes Available functions
Segmentation type Deep Learning Thresholding Thresholding Thresholding Thresholding, Naive Bayes, Edge detection,k-means

Contact between elements solution SAHI and Watershed NA NA Watershed Watershed
Workflow Integration Yes Yes, but without calibration, morphometrics. Yes, but without calibration, morphometrics. Yes, without morphometrics Independent workflows, integrated in some cases.

Regarding the key points: (1) our workflow is open source, enabling customization and further development for future studies and applications, as already demonstrated in the manuscript with other fruits; (2) it can run on any operating system with Python installed; (3) together with PlantCV, it is the only one among those mentioned written in Python—a language more widely used than MATLAB, with greater availability of learning resources, generally easier to adopt, and therefore more accessible for a broader range of breeding programs; (4) it can also run online using free and widely adopted resources such as Google Colab; (5) not all of the mentioned tools can analyze color; (6) not all provide the complete set of morphological measurements offered here; (7) none integrate both elliptical Fourier analyses and pixel-based PCA for morphometric analyses; (8) AlmondCV includes functions for camera and color calibration, which are not available in all of them; (9) for segmentation, AlmondCV introduces a novel approach not present in the others, using deep learning models and allowing easy development of a custom segmentation model, whereas the rest mainly rely on thresholding, which, as noted in the SeedExtractor paper and confirmed by our experience, often requires manual parameter adjustment: "Due to a wide range of variation in seed size and color, it is difficult to automatically set optimal size and color ranges (for all the color spaces)," a limitation that can be critical in breeding programs with high phenotypic diversity; additionally, the SeedExtractor paper states (10): "The application requires that the seeds are not touching each other when imaged," an issue we also encountered and addressed through a watershed algorithm similar to GrainScan (but here open source) and a novel method using SAHI that separates elements in their respective functions; and (10) our workflow integrates multiple components—camera calibration, image segmentation, and morphological/morphometric trait extraction—that are not fully combined in any of the other tools mentioned.
This has been explained in the introduction to provide clarity for readers
The term 'breakthrough' refers to the phenotyping bottleneck in almond breeding, which our study surpasses relative to previous research and current breeding practices. We have, however, reformulated the sentence to improve clarity

2. Within horticultural crops, workflows and studies (not acknowledged in this article) are available that can be adapted or modified to do the same thing. For example - publications from as early as 2020 used machine learning models to measure size and mass of almonds, however, this relevant study was not acknowledged by the authors https://onlinelibrary.wiley.com/doi/full/10.1111/jfpe.13374 . Discuss how the presented method is better than the aforementioned article, justify the claim 'breakthrough'.

Response: Thank you for your comments. Regarding the use of the term 'breakthrough,' as mentioned earlier, we refer to the phenotyping bottleneck in almond breeding, which is justified by comparison with previous studies and current phenotyping practices in almond breeding programs. With respect to the advantages over the mentioned article, many of the points noted in our previous comment also apply (e.g., thresholding instead of deep learning, lack of open-source availability, unspecified programming language, unknown compatibility with different operating systems, measurement limited to length, and no calibration procedures). Moreover, that study analyzed only a single variety (NonPareil), whereas our workflow phenotypes more than 600 genotypes, capturing much greater diversity and providing a broader test. In addition, the previous study focuses primarily on industrial applications, whereas our approach emphasizes the accuracy and diversity required in breeding programs. We have included this context in the introduction to highlight the use of imaging in almonds.

3. The authors claim successfully testing the pipeline for apples and strawberries. Information for fruit size can be extracted from 2D images; however, the example results show only length, width, circularity, and ellipse ratio. How do these parameters assist fruit breeders? Since it is segmentation based classification using a reference scale, the aforementioned tools particularly SeedExtractor can generate similar results. Does it qualify the tool for integration into fruit crops breeding pipeline? Moreover, fruit breeders require on-tree analysis, recent advancements have enabled 3D sensing for significantly better detection particularly using cost effective RGB-D cameras.

Response: Thank you for your comments. In this study, we only test the workflow on the specified species,

and the traits measured are all described in the methodology section for the general method (length, width, area, perimeter, hull area, solidity, aspect ratio, circularity, ellipse ratio, and colour using the Lab* model). Additionally, when fruits are placed in an ordered and flat orientation, the same traits as for almonds can be measured (e.g., width at three different heights: 25%, 50%, and 75% of the length, vertical and horizontal symmetry). In the manuscript, we propose that our workflow could be expanded to other fruits and allow the development of crop-specific traits using the existing functions as a base/template, as illustrated with simple examples in apple and strawberry.
In addition, almond shape has been traditionally evaluated subjectively and is time-consuming (Martinez-Garcia et al 2019). By definition, however, morphological traits are highly dimensional (Zingaretii et al 2021). Computing only linear, univariate phenotype leads to a loss of information by extremely simplifying the features of a shape (Klingenberg 2010, Claes 2014). The use of geometric-morphometric approaches for shape analysis is warranted (Dryden 1998). From a breeding point of view, our advancement in high-throughput phenotyping can generate more precise phenotypic datasets that provide valuable information for understanding the genetic basis of our traits, can help to a better estimation of heritability and to improve predictive accuracy in strategies such as Genomic Selection. The integration of cutting-edge technologies like GS and HTP has the potential to accelerate gains in breeding.
Regarding differences with SeedExtractor, as mentioned in our first comment, SeedExtractor does not allow, for instance, the extraction of morphometric traits via elliptical Fourier analyses or pixel-based PCA. As noted in the introduction, these approaches have been applied in apple and strawberry breeding for target traits, providing examples of how such traits can assist fruit breeders (Feldmann et al., 2020; Keller et al., 2024).
Regarding 3D fruit phenotyping in the field, we agree that it represents a promising area for breeding. However, it typically requires more hardware and software resources, which can complicate implementation and limit adoption by breeding programs. A clear example is Li et al. (2025), who developed a sophisticated autonomous rover to phenotype traits such as fruit number, maturity, and yield in blueberry. Moreover, field phenotyping may not be equally effective for all species; for instance, almonds present additional challenges due to their shells, tree architecture, and the dispersed bearing of fruits, which is more complex than in blueberry bushes. Additionally, measurement accuracy for morphological fruit traits would generally be lower in the field than under controlled conditions, which can be critical for breeding schemes.

Minor Comments -
1. Change title - This study uses deep learning models which are a type of AI. AI is a broader term. Additionally, the potential for utility in fruit breeding pipeline appears to be limited. Suggested - Open RGB imaging workflow for morphological and morphometric analysis of almond kernels using deep learning.
Response: Thank you for your comments. We agree that “deep learning” is more precise than the broader term “AI.” However, we respectfully disagree with the assessment that the workflow has limited utility in fruit breeding pipelines. Our workflow provides tools that are relevant to breeding programs, as demonstrated in species such as strawberry and apple (Feldmann et al., 2020; Keller et al., 2024), while also incorporating new advances and features that enhance phenotyping capabilities.
2. The video tutorial showed using YOLOv8, mention it in the methods. Add information for all settings used in CVAT.
Response: Thank you for your comments. We have included it in the “Develop your segmentation model” section.

Reviewer #2: We would like to thank you for submitting your manuscript to our journal.The manuscript proposes an AI-powered open RGB imaging workflow for morphological and morphometric analysis of fruits or other plant organs, with almonds as a case study. The workflow, developed in Python, covers the full pipeline from image pre-processing, segmentation model development and deployment, to trait measurement and analysis. It aims to improve the efficiency and accuracy of phenotyping in breeding programs by addressing the limitations of traditional methods, such as their time-consuming and labor-intensive nature.
Response: Thank you for your comments.

However, there are still the following problems in this paper that need further improvement:

1.Table formatting: Some of the tables in the manuscript do not follow the formatting standards of the journal. The authors are encouraged to revise them accordingly to ensure clarity, consistency, and ease of understanding.
Response: Thank you for your comments. We have followed the table preparation guidelines described in the Instructions to Authors (https://academic.oup.com/gigascience/pages/instructions_to_authors#Preparing%20Tables), including the requirement that all tables are introduced in the text, properly formatted, and have titles with fewer than 15 words. However, we are not certain which specific formatting aspect may not meet the journal's standards. We would greatly appreciate further clarification so we can make the necessary adjustments to ensure full compliance.

2.Formula presentation: Certain mathematical formulas are not clearly formatted and appear disorganized. The authors should re-typeset the equations to improve readability and provide clearer explanations for each formula.
Response: Thank you for your comments. In our original Word document, the formulas appear correctly formatted. We suspect that the conversion process during submission may have altered their layout. We will recheck the typesetting in the submitted version and re-typeset the equations to ensure they are properly aligned and readable.

3.Introduction: The introduction could be strengthened by more thoroughly explaining the relationship between phenotypic data and breeding. The authors may also discuss how phenotyping data supports genomic selection and accelerates breeding via high-throughput workflows.
Response: Thank you for your comments. We have reinforced the introduction to clarify the importance of phenotyping in breeding and its impact in genomic selection.
4.Methods section: While the paper clearly explains how morphological traits and kernel thickness are measured, it does not sufficiently explain how this data contributes to breeding decisions. The authors should elaborate on how the extracted traits are applied in practical breeding or selection strategies.
Response: Thank you for your comments. We have reinforced the discussion to clarify the importance how can be incorporated in breeding approaches.

5.Lack of algorithmic novelty: While the integration of existing tools is commendable, the core methods used (e.g., YOLO, SAHI) are based on publicly available models, without introducing new algorithmic components or comparative ablation studies. The authors are advised to clarify the unique contribution of their workflow, especially in terms of engineering integration or practical usability.
Response: Thank you for your comments. We acknowledge that the core methods employed are publicly available, which we also consider a strength of our work due to the use of pre-trained generalist models (Bommasani et al., 2021). Furthermore, we implemented specific adaptations in the SAHI method, as indicated in the Develop your segmentation model section of the methodology, to enable the use of the retina_mask argument, which improves fine-detail accuracy in segmentation. In any case, we have clarified our contribution in terms of integration and practical usability in the manuscript, specifically at the end of the introduction.
Bommasani R, Hudson DA, Adeli E, Altman R, Arora S, Arx S von, et al.2021. On the Opportunities and Risks of Foundation Models. arXiv;

6.Limited evaluation metrics: The performance of segmentation models is only reported using error percentage. The inclusion of standard metrics such as IoU, Precision, Recall, and F1-score would allow for a more comprehensive evaluation and comparison across models (see LLRL methods).
Response: Thank you for your comments. We agree, and indeed these metrics are presented in Supplementary Figure 2, although they were not discussed in detail in the original manuscript. We have now included a more detailed discussion of these results in the Results section ("YOLO fine-tuned segmentation models performance and reconstruction errors").

7.Figures and captions: Currently, figure images and their descriptions are placed separately, which may reduce readability. It is recommended to place figure captions immediately beneath or alongside the figures to enhance the paper's coherence and user-friendliness.
Response: Thank you for your comments. We agree with your observation; however, according to the journal's guidelines, figure files must be submitted separately and not embedded within the manuscript.

https://academic.oup.com/gigascience/pages/instructions_to_authors#Preparing%20Figures

8.Trait extension suggestions: In order to enhance the expressiveness and resolution of phenotypic trait modeling, authors are advised to refer to the relevant research on extracting fine-grained phenotypic features in plant images in recent years. For example, PlanText proposed a progressive visual guidance strategy to help improve the modeling quality of phenotypic traits in images.
Response: Thank you for your comments. We were not aware of this visual guidance strategy, but we agree that it could be particularly useful for the abstract traits discussed in our manuscript. We have now included a brief mention of this possibility in the discussion.

Therefore, I would like to give a "Major Revision" recommendation.

Reviewer #3: The present study entitled "Open RGB Imaging Workflow for Morphological and Morphometric Analysis of Fruits
using AI: A Case Study on Almonds" reported the development of a Python-based image analysis pipeline that extract morphological traits of almond nut shells and kernels. A case study was conducted to use the developed pipeline to analyze breeding populations of 665 genotypes and extract both general morphology traits such as height, length, area, aspect ratio, etc. and specialized traits for almond such as width at three heights, vertical and horizontal symmetry, etc. Further, each nut shell or kernel was weighed, so models were established to use the weight and morphological traits to predict the thickness of each nut shell or kernel. In addition to morphological traits, morphometric (or shape) was extracted for each nut shell or kernel. Clustering analysis was performed on the morphometric traits to identify variability among genotypes. To further validate the efficacy of the extracted traits, broad-sense heritability was calculated and used as a criterion.

The major contribution of this study is the integration of different components (e.g., camera calibration, image segmentation, and morphological/morphometric trait extraction, etc.) as a user-accessible, open-source Python implementation for the plant breeding community, especially for almond breeders. However, there several aspects that could be further improved.
Response: Thank you for your comments.

First, the present study showed the most number of samples that were phenotyped by the proposed pipeline among recent efforts on almond nut shell and/or kernel phenotyping. However, there was no clear evidence to demonstrate direct benefits to ongoing almond breeding. Certain traits (e.g., aspect ratio, tip/top/side curvatures) could be included in a breeding program, but what's the significance of including these traits in breeding programs. Are they crucial to either improve the productivity, quality, or other management practices or processing practices for the almond industry, especially given breeding context?
Response: Thank you for your comments. We agree that we should provide more information on the benefits of these traits. For this reason, we have added a discussion on this topic in the Introduction.
“These traits directly influence market value and consumer acceptance [25,26]. Larger kernel sizes are generally more economically valuable [24], while smaller sizes are preferred for specific applications, such as chocolate bars [22]. Shape is also important for industrial processes: elongated almonds are preferred for slicing, whereas round almonds are generally more appreciated by consumers [22,23][23]. Geometric parameters also affect the mechanical properties of almonds and other nuts, which is relevant for processing [27,28]. For example, elongated shapes have been shown to result in lower breakage during blanching, while rounder shapes reduce breakage during shelling in Spanish almond industry machinery [29].”
We have also highlighted in the Discussion the concerns regarding breakage during industrial processing and the role that almond nut and kernel shape can play in this context.

Second, the pipeline uses deep learning-based segmentation which is powerful to handle complex background. Based on the limited figures or example images in the GitHub repo, the background is mostly single colored (e.g., white or black) without appearances that may confuse even conventional segmentation, especially if image color is calibrated. Assuming most of the almond nut shell and kernel analyses would be done in a laboratory condition, it is not convincing why conventional segmentation methods may not be preferred if both illumination and camera configuration can be well controlled.

Ultimately, the question is whether it is worthy the effort of labeling hundreds of images to fine-tune a deep learning segmentation model compared to a careful hardware-software design to make operation more efficient. Or with the simplified background, vision foundation models such as SAM will be sufficient.
Response: Thank you for your comments. We have carefully considered these aspects. While we initially explored conventional segmentation methods, our experience indicates that deep learning-based segmentation models offer clear advantages for a high-throughput phenotyping system:
1. We observed that due to the large phenotypic diversity in almonds—including variations in color, shape, texture, and size—thresholding techniques and similar approaches often require manual adjustments. We believe this issue is likely to occur in breeding programs of other species with high phenotypic diversity. Such manual interventions hinder automation, whether processing images in real time or during post-processing. Indeed, similar limitations have been reported in other software, such as SeedExtractor: “Due to a wide range of variation in seed size and color, it is difficult to automatically set optimal size and color ranges (for all the color spaces).”
2. Another aspect highlighted in SeedExtractor, which we also observed when using conventional segmentation, is that “The application requires that the seeds are not touching each other when imaged.” This challenge has been addressed in software such as GrainScan, which implements a watershed algorithm (although it is not open-source). In our work, we applied an open-source watershed approach, and additionally employed another method using SAHI, which effectively separates individual elements for downstream analysis.
3. Additionally, we captured images of multiple groups of almonds placed inside painted rectangles (see Methodology), for which we fine-tuned simple deep learning models to recognize and separate the groups. Using conventional segmentation, we frequently encountered problems when almonds touched the rectangle boundaries or when the contours of the rectangles were not correctly enclosed during processing.
4. With fine-tuned deep learning models, we can relax the strict requirements on hardware and software design, which becomes particularly important when working with the large sample sizes as here. In such scenarios, processing speed can be just as critical as precision during image acquisition for the overall feasibility of the process.
5. For phenotyping processes, minimizing manual adjustments facilitates the adoption of the task by technicians without a computer vision background, which also helps to accelerate the overall workflow.
In response to whether it is worthwhile to label hundreds of images to fine-tune a deep learning segmentation model, we argue that it is. The labeling process has become significantly faster, taking approximately 30 minutes to annotate all images using a combination of CVAT and SAM, as demonstrated here. Regarding the direct use of foundation models, Bommasani et al. (2021) note that foundation models “are intermediary assets; they are unfinished and generally should not be used directly, instead requiring adaptation for specific downstream tasks.” Given that fine-tuning is not time- or cost-intensive, it improves segmentation performance, accuracy, and consistency, while reducing the need for manual supervision.
Bommasani R, Hudson DA, Adeli E, Altman R, Arora S, Arx S von, et al.2021. On the Opportunities and Risks of Foundation Models. arXiv;

SeedExtractor https://www.frontiersin.org/journals/plant-science/articles/10.3389/fpls.2020.581546/full#h3

GrainScan https://link.springer.com/article/10.1186/1746-4811-10-23

Third, in the Introduction section, some technical statements should be revised to make them accurate. For example, image segmentation is a core computer vision task rather than relying on computer vision algorithms. One-stage and two-stage strategies are used to differentiate models for object detection not image segmentation. Further, Faster RCNN is an object detection model and cannot do image segmentation. It is highly recommended that the authors could find a computer science or engineering colleague to proofread the technical statements to ensure the accuracy.
Response: Thank you for your comments. We have revised the Introduction to improve technical accuracy. Regarding the use of “one-stage” and “two-stage” terminology in instance segmentation, we acknowledge that these terms are more commonly associated with object detection. However, we have retained them in our manuscript because they are used in recent literature on instance segmentation (e.g., Bolya et al., 2019; Gu et al., 2022) to describe architectural strategies. We are open to updating the terminology if more precise references or conventions are suggested.

Bolya, D., Zhou, C., Xiao, F., & Lee, Y. J. (2019). Yolact: Real-time instance segmentation. In Proceedings of the IEEE/CVF international conference on computer vision (pp. 9157-9166).
Gu, W., Bai, S., & Kong, L. (2022). A review on 2D instance segmentation based on deep neural networks. Image and Vision Computing, 120, 104401.

Last, it is appreciated the authors effort on making an open-source software for the community. However, the dataset can be equally important to advance the scientific discovery and technology development. Is there any plan to make the dataset publicly available to help facilitate the development of additional computer vision algorithms for almond phenotyping?
Response: Thank you for your comments. The dataset was submitted alongside the manuscript to GigaScience and has also been deposited in Zenodo. It is currently available to reviewers (see Data Availability section) and will be made publicly accessible to the scientific community upon publication.

Please also take a moment to check our website at https://www.editorialmanager.com/giga/l.asp?i=218825&l=T02XNJYZ for any additional comments that were saved as attachments. Please note that as GigaScience has a policy of open peer review, you will be able to see the names of the reviewers.

Editorial Manager®

Cookie Preference Center

We use cookies which are necessary to make our site work. We may also use additional cookies to analyze, improve and personalize our content and your digital experience. For more information, see our Cookie Policy.

You may choose not to allow some types of cookies. However, blocking some types may impact your experience of our site and the services we are able to offer. See the different category headings below to find out more or change your settings.

Allow all

Manage Consent Preferences

Strictly Necessary Cookies

Always active

These cookies are necessary for the website to function and cannot be switched off in our systems. They are usually only set in response to actions made by you which

amount to a request for services, such as setting your privacy preferences, logging in or filling in forms. You can set your browser to block or alert you about these cookies, but some parts of the site will not then work.

Functional Cookies

These cookies enable the website to provide enhanced functionality and personalisation. They may be set by us or by third party providers whose services we have added to our pages. If you do not allow these cookies then some or all of these services may not function properly.

Performance Cookies

These cookies allow us to count visits and traffic sources so we can measure and improve the performance of our site. They help us to know which pages are the most and least popular and see how visitors move around the site.

Targeting Cookies

These cookies may be set through our site by our advertising partners. They may be used by those companies to build a profile of your interests and show you relevant adverts on other sites. If you do not allow these cookies, you will experience less targeted advertising.

Cookie List

Clear

Apply Cancel

Consent Leg.Interest

Confirm my choices

Powered by onetrust
