# Supplementary material for: Open RGB imaging workflow for morphological and morphometric analysis of fruits using deep learning: a case study on almonds
Source: Gigascience. 2025 Dec 19;15:giaf157. doi: 10.1093/gigascience/giaf157 (PMC12970599; doi:10.1093/gigascience/giaf157)
Supplement: giaf157_Authors_Response_To_Reviewer_Comments_revision_1 [file giaf157_authors_response_to_reviewer_comments_revision_1.pdf]

Author's Response To Reviewer Comments

Dear Editor and Reviewer,
We sincerely appreciate your contributions and comments and proceed to address your suggestions point-by-point in this document. Responses are in red color.

Editor report:

Response: Thank you very much for your comments and for all the information provided. The workflow is registered in bio.tools (https://bio.tools/almondcv2), SciCrunch.org (SCR_027064), and WorkflowHub (https://workflowhub.eu/workflows/1731), and all of these resources are also referenced in the paper.

Reviewer report:
Reviewer #2: Comments to the Author:
The manuscript has been substantially improved in response to the previous review, with clearer presentation and stronger methodological description. But there are still unresolved issues:
(1)The paper should provide a more detailed description and discussion of evaluation metrics such as IoU, Precision, Recall, and F1-score to fully reflect the model's performance on manuscript and enhance its persuasiveness by comparing it with other models. For more details, see Yu et al., 2025, Location-guided Lesions Representation Learning, which provides a systematic analysis and discussion of these metrics.
With these minor revisions, the manuscript will be ready for publication.

Response: Thank you for your comments. We have expanded the methodology section to include more details on the model's performance metrics, and we have added sentences to the results and discussion to highlight the strong performance of the models developed through the workflow.

Editorial Manager®

Cookie Preference Center

We use cookies which are necessary to make our site work. We may also use additional cookies to analyze, improve and personalize our content and your digital experience. For more information, see our Cookie Policy.

You may choose not to allow some types of cookies. However, blocking some types may impact your experience of our site and the services we are able to offer. See the different category headings below to find out more or change your settings.

Allow all

Manage Consent Preferences

Strictly Necessary Cookies

Always active

These cookies are necessary for the website to function and cannot be switched off in our systems. They are usually only set in response to actions made by you which amount to a request for services, such as setting your privacy preferences, logging in or filling in forms. You can set your browser to block or alert you about these cookies, but some parts of the site will not then work.

Functional Cookies

These cookies enable the website to provide enhanced functionality and personalisation. They may be set by us or by third party providers whose services we have added to our pages. If you do not allow these cookies then some or all of these services may not function properly.

Performance Cookies

These cookies allow us to count visits and traffic sources so we can measure and improve the performance of our site. They help us to know which pages are the most and least popular and see how visitors move around the site.

Targeting Cookies

These cookies may be set through our site by our advertising partners. They may be used by those companies to build a profile of your interests and show you relevant adverts on other sites. If you do not allow these cookies, you will experience less targeted advertising.

Cookie List

Clear

Apply Cancel

Consent Leg.Interest

Confirm my choices

Powered by onetrust
