# Supplementary material for: Open RGB imaging workflow for morphological and morphometric analysis of fruits using deep learning: a case study on almonds
Source: Gigascience. 2025 Dec 19;15:giaf157. doi: 10.1093/gigascience/giaf157 (PMC12970599; doi:10.1093/gigascience/giaf157)
Supplement: giaf157_Reviewer_1_Report_original_submission — Yuvraj Chopra -- 7/1/2025 [file giaf157_reviewer_1_report_original_submission.pdf]

Reviewer Report

Title: Open RGB Imaging Workflow for Morphological and Morphometric Analysis of Fruits using Deep Learning: A Case Study on Almonds.

Version: Original Submission **Date:** 7/1/2025

Reviewer name: Yuvraj Chopra

Reviewer Comments to Author:

The methods described in this article represent a useful tool for fast and reliable morphometric analysis of almonds with potential applications in fruits. The pipeline is technically sound, and publicly available workflow will advance the adoption of this technology. However, there are critical concerns which needs to be addressed before the manuscript could be further proceeded for publication in the journal -
Major Comments -
1. Authors claim this technique as a new phenotyping tool with breakthrough implications; however, I object to this claim. Numerous studies have utilized this technique in plant phenotyping to the extent that labeling it as a new phenotyping tool may not be ideal. Additionally, for kernel or seed morphometrics, a wide array of user-friendly, open-source tools have already been developed and are readily available, for example,
- SeedExtractor https://www.frontiersin.org/journals/plant-science/articles/10.3389/fpls.2020.581546/full#h3
- SmartGrain https://academic.oup.com/plphys/article/160/4/1871/6109568
- GrainScan https://link.springer.com/article/10.1186/1746-4811-10-23
- PlantCV https://peerj.com/articles/4088/

These tools and a lot of other options can be readily used for almond kernel morphometrics. Authors are requested to discuss/compare advantages/performance of their model with SeedExtractor, SmartGrain, and GrainScan.
2. Within horticultural crops, workflows and studies (not acknowledged in this article) are available that can be adapted or modified to do the same thing. For example - publications from as early as 2020 used machine learning models to measure size and mass of almonds, however, this relevant study was not acknowledged by the authors https://onlinelibrary.wiley.com/doi/full/10.1111/jfpe.13374 . Discuss how the presented method is better than the aforementioned article, justify the claim 'breakthrough'.
3. The authors claim successfully testing the pipeline for apples and strawberries. Information for fruit size can be extracted from 2D images; however, the example results show only length, width, circularity, and ellipse ratio. How do these parameters assist fruit breeders? Since it is segmentation based classification using a reference scale, the aforementioned tools particularly SeedExtractor can generate similar results. Does it qualify the tool for integration into fruit crops breeding pipeline? Moreover, fruit breeders require on-tree analysis, recent advancements have enabled 3D sensing for significantly better detection particularly using cost effective RGB-D cameras.
Minor Comments -
1. Change title - This study uses deep learning models which are a type of AI. AI is a broader term.

Additionally, the potential for utility in fruit breeding pipeline appears to be limited. Suggested - Open RGB imaging workflow for morphological and morphometric analysis of almond kernels using deep learning.
2. The video tutorial showed using YOLOv8, mention it in the methods. Add information for all settings used in CVAT.

Methods

Are the methods appropriate to the aims of the study, are they well described, and are necessary controls included? Choose an item.

Conclusions

Are the conclusions adequately supported by the data shown? Choose an item.

Reporting Standards

Does the manuscript adhere to the journal's guidelines on minimum standards of reporting? Choose an item.

Choose an item.

Statistics

Are you able to assess all statistics in the manuscript, including the appropriateness of statistical tests used? Choose an item.

Quality of Written English

Please indicate the quality of language in the manuscript: Choose an item.

Declaration of Competing Interests

Please complete a declaration of competing interests, considering the following questions:

- Have you in the past five years received reimbursements, fees, funding, or salary from an organisation that may in any way gain or lose financially from the publication of this manuscript, either now or in the future?
- Do you hold any stocks or shares in an organisation that may in any way gain or lose financially from the publication of this manuscript, either now or in the future?
- Do you hold or are you currently applying for any patents relating to the content of the manuscript?
- Have you received reimbursements, fees, funding, or salary from an organization that holds or has applied for patents relating to the content of the manuscript?

- Do you have any other financial competing interests?
- Do you have any non-financial competing interests in relation to this paper?

If you can answer no to all of the above, write 'I declare that I have no competing interests' below. If your reply is yes to any, please give details below.

I declare that I have no competing interests

I agree to the open peer review policy of the journal. I understand that my name will be included on my report to the authors and, if the manuscript is accepted for publication, my named report including any attachments I upload will be posted on the website along with the authors' responses. I agree for my report to be made available under an Open Access Creative Commons CC-BY license (http://creativecommons.org/licenses/by/4.0/). I understand that any comments which I do not wish to be included in my named report can be included as confidential comments to the editors, which will not be published.

Choose an item.

To further support our reviewers, we have joined with Publons, where you can gain additional credit to further highlight your hard work (see: https://publons.com/journal/530/gigascience). On publication of this paper, your review will be automatically added to Publons, you can then choose whether or not to claim your Publons credit. I understand this statement.

Yes Choose an item.
