# Supplementary material for: Open RGB imaging workflow for morphological and morphometric analysis of fruits using deep learning: a case study on almonds
Source: Gigascience. 2025 Dec 19;15:giaf157. doi: 10.1093/gigascience/giaf157 (PMC12970599; doi:10.1093/gigascience/giaf157)
Supplement: giaf157_Reviewer_2_Report_original_submission — Qi Wang -- 7/1/2025 [file giaf157_reviewer_2_report_original_submission.pdf]

**Reviewer Report**

**Title: Open RGB Imaging Workflow for Morphological and Morphometric Analysis of Fruits using Deep Learning: A Case Study on Almonds.**

**Version: Original Submission** **Date:** 7/1/2025

**Reviewer name: Qi Wang**

**Reviewer Comments to Author:**

We would like to thank you for submitting your manuscript to our journal.The manuscript proposes an AI-powered open RGB imaging workflow for morphological and morphometric analysis of fruits or other plant organs, with almonds as a case study. The workflow, developed in Python, covers the full pipeline from image pre-processing, segmentation model development and deployment, to trait measurement and analysis. It aims to improve the efficiency and accuracy of phenotyping in breeding programs by addressing the limitations of traditional methods, such as their time-consuming and labor-intensive nature.

However, there are still the following problems in this paper that need further improvement:

1.Table formatting: Some of the tables in the manuscript do not follow the formatting standards of the journal. The authors are encouraged to revise them accordingly to ensure clarity, consistency, and ease of understanding.

2.Formula presentation: Certain mathematical formulas are not clearly formatted and appear disorganized. The authors should re-typeset the equations to improve readability and provide clearer explanations for each formula.

3.Introduction: The introduction could be strengthened by more thoroughly explaining the relationship between phenotypic data and breeding. The authors may also discuss how phenotyping data supports genomic selection and accelerates breeding via high-throughput workflows.

4.Methods section: While the paper clearly explains how morphological traits and kernel thickness are measured, it does not sufficiently explain how this data contributes to breeding decisions. The authors should elaborate on how the extracted traits are applied in practical breeding or selection strategies.

5.Lack of algorithmic novelty: While the integration of existing tools is commendable, the core methods used (e.g., YOLO, SAHI) are based on publicly available models, without introducing new algorithmic components or comparative ablation studies. The authors are advised to clarify the unique contribution of their workflow, especially in terms of engineering integration or practical usability.

6.Limited evaluation metrics: The performance of segmentation models is only reported using error percentage. The inclusion of standard metrics such as IoU, Precision, Recall, and F1-score would allow for a more comprehensive evaluation and comparison across models (see LLRL methods).

7.Figures and captions: Currently, figure images and their descriptions are placed separately, which may reduce readability. It is recommended to place figure captions immediately beneath or alongside the figures to enhance the paper's coherence and user-friendliness.

8.Trait extension suggestions: In order to enhance the expressiveness and resolution of phenotypic trait modeling, authors are advised to refer to the relevant research on extracting fine-grained phenotypic

features in plant images in recent years. For example, PlanText proposed a progressive visual guidance strategy to help improve the modeling quality of phenotypic traits in images.
Therefore, I would like to give a "Major Revision" recommendation.

**Methods**

Are the methods appropriate to the aims of the study, are they well described, and are necessary controls included? Choose an item.

**Conclusions**

Are the conclusions adequately supported by the data shown? Choose an item.

**Reporting Standards**

Does the manuscript adhere to the journal's guidelines on minimum standards of reporting? Choose an item.

Choose an item.

**Statistics**

Are you able to assess all statistics in the manuscript, including the appropriateness of statistical tests used? Choose an item.

**Quality of Written English**

Please indicate the quality of language in the manuscript: Choose an item.

**Declaration of Competing Interests**

Please complete a declaration of competing interests, considering the following questions:

- Have you in the past five years received reimbursements, fees, funding, or salary from an organisation that may in any way gain or lose financially from the publication of this manuscript, either now or in the future?
- Do you hold any stocks or shares in an organisation that may in any way gain or lose financially from the publication of this manuscript, either now or in the future?
- Do you hold or are you currently applying for any patents relating to the content of the manuscript?
- Have you received reimbursements, fees, funding, or salary from an organization that holds or has applied for patents relating to the content of the manuscript?
- Do you have any other financial competing interests?
- Do you have any non-financial competing interests in relation to this paper?

If you can answer no to all of the above, write 'I declare that I have no competing interests' below. If your reply is yes to any, please give details below.

I declare that I have no competing interests.

I agree to the open peer review policy of the journal. I understand that my name will be included on my report to the authors and, if the manuscript is accepted for publication, my named report including any attachments I upload will be posted on the website along with the authors' responses. I agree for my report to be made available under an Open Access Creative Commons CC-BY license (http://creativecommons.org/licenses/by/4.0/). I understand that any comments which I do not wish to be included in my named report can be included as confidential comments to the editors, which will not be published.

Choose an item.

To further support our reviewers, we have joined with Publons, where you can gain additional credit to further highlight your hard work (see: https://publons.com/journal/530/gigascience). On publication of this paper, your review will be automatically added to Publons, you can then choose whether or not to claim your Publons credit. I understand this statement.

Yes Choose an item.
