# Supplementary material for: Open RGB imaging workflow for morphological and morphometric analysis of fruits using deep learning: a case study on almonds
Source: Gigascience. 2025 Dec 19;15:giaf157. doi: 10.1093/gigascience/giaf157 (PMC12970599; doi:10.1093/gigascience/giaf157)
Supplement: giaf157_Reviewer_3_Report_original_submission — Yu Jiang -- 7/29/2025 [file giaf157_reviewer_3_report_original_submission.pdf]

Reviewer Report

Title: Open RGB Imaging Workflow for Morphological and Morphometric Analysis of Fruits using Deep Learning: A Case Study on Almonds.

Version: Original Submission **Date:** 7/29/2025

Reviewer name: Yu Jiang

Reviewer Comments to Author:

The present study entitled "Open RGB Imaging Workflow for Morphological and Morphometric Analysis of Fruits
using AI: A Case Study on Almonds" reported the development of a Python-based image analysis pipeline that extract morphological traits of almond nut shells and kernels. A case study was conducted to use the developed pipeline to analyze breeding populations of 665 genotypes and extract both general morphology traits such as height, length, area, aspect ratio, etc. and specialized traits for almond such as width at three heights, vertical and horizontal symmetry, etc. Further, each nut shell or kernel was weighed, so models were established to use the weight and morphological traits to predict the thickness of each nut shell or kernel. In addition to morphological traits, morphometric (or shape) was extracted for each nut shell or kernel. Clustering analysis was performed on the morphometric traits to identify variability among genotypes. To further validate the efficacy of the extracted traits, broad-sense heritability was calculated and used as a criterion.

The major contribution of this study is the integration of different components (e.g., camera calibration, image segmentation, and morphological/morphometric trait extraction, etc.) as a user-accessible, open-source Python implementation for the plant breeding community, especially for almond breeders. However, there several aspects that could be further improved.

First, the present study showed the most number of samples that were phenotyped by the proposed pipeline among recent efforts on almond nut shell and/or kernel phenotyping. However, there was no clear evidence to demonstrate direct benefits to ongoing almond breeding. Certain traits (e.g., aspect ratio, tip/top/side curvatures) could be included in a breeding program, but what's the significance of including these traits in breeding programs. Are they crucial to either improve the productivity, quality, or other management practices or processing practices for the almond industry, especially given breeding context?

Second, the pipeline uses deep learning-based segmentation which is powerful to handle complex background. Based on the limited figures or example images in the GitHub repo, the background is mostly single colored (e.g., white or black) without appearances that may confuse even conventional segmentation, especially if image color is calibrated. Assuming most of the almond nut shell and kernel analyses would be done in a laboratory condition, it is not convincing why conventional segmentation methods may not be preferred if both illumination and camera configuration can be well controlled. Ultimately, the question is whether it is worthy the effort of labeling hundreds of images to fine-tune a deep learning segmentation model compared to a careful hardware-software design to make operation more efficient. Or with the simplified background, vision foundation models such as SAM will be

sufficient.
Third, in the Introduction section, some technical statements should be revised to make them accurate. For example, image segmentation is a core computer vision task rather than relying on computer vision algorithms. One-stage and two-stage strategies are used to differentiate models for object detection not image segmentation. Further, Faster RCNN is an object detection model and cannot do image segmentation. It is highly recommended that the authors could find a computer science or engineering colleague to proofread the technical statements to ensure the accuracy.
Last, it is appreciated the authors effort on making an open-source software for the community. However, the dataset can be equally important to advance the scientific discovery and technology development. Is there any plan to make the dataset publicly available to help facilitate the development of additional computer vision algorithms for almond phenotyping?

Methods

Are the methods appropriate to the aims of the study, are they well described, and are necessary controls included? Choose an item.

Conclusions

Are the conclusions adequately supported by the data shown? Choose an item.

Reporting Standards

Does the manuscript adhere to the journal's guidelines on minimum standards of reporting? Choose an item.

Choose an item.

Statistics

Are you able to assess all statistics in the manuscript, including the appropriateness of statistical tests used? Choose an item.

Quality of Written English

Please indicate the quality of language in the manuscript: Choose an item.

Declaration of Competing Interests

Please complete a declaration of competing interests, considering the following questions:

- Have you in the past five years received reimbursements, fees, funding, or salary from an organisation that may in any way gain or lose financially from the publication of this manuscript, either now or in the future?

- Do you hold any stocks or shares in an organisation that may in any way gain or lose financially from the publication of this manuscript, either now or in the future?
- Do you hold or are you currently applying for any patents relating to the content of the manuscript?
- Have you received reimbursements, fees, funding, or salary from an organization that holds or has applied for patents relating to the content of the manuscript?
- Do you have any other financial competing interests?
- Do you have any non-financial competing interests in relation to this paper?

If you can answer no to all of the above, write 'I declare that I have no competing interests' below. If your reply is yes to any, please give details below.

'I declare that I have no competing interests

I agree to the open peer review policy of the journal. I understand that my name will be included on my report to the authors and, if the manuscript is accepted for publication, my named report including any attachments I upload will be posted on the website along with the authors' responses. I agree for my report to be made available under an Open Access Creative Commons CC-BY license (http://creativecommons.org/licenses/by/4.0/). I understand that any comments which I do not wish to be included in my named report can be included as confidential comments to the editors, which will not be published.

Choose an item.

To further support our reviewers, we have joined with Publons, where you can gain additional credit to further highlight your hard work (see: https://publons.com/journal/530/gigascience). On publication of this paper, your review will be automatically added to Publons, you can then choose whether or not to claim your Publons credit. I understand this statement.

Yes Choose an item.
